# Direct introduction of nitrogen and oxygen functionality with spatial control using copper catalysis†
†Electronic supplementary information (ESI) available. See DOI: 10.1039/c8sc03288b


**DOI:** 10.1039/c8sc03288b

**Published:** 2018-09-17

**Authors:** James B. Shaum, David J. Fisher, Miranda M. Sroda, Luis Limon, Javier Read de Alaniz

**Affiliations:** a Department of Chemistry and Biochemistry , University of California , Santa Barbara , California 93106 , USA . Email: javier@chem.ucsb.edu

## Abstract

Synthetic chemists have spent considerable effort optimizing the synthesis of nitrogen and oxygen containing compounds through a number of methods; however, direct introduction of N- and O-functionality remains challenging. Presented herein is a general method to allow for the simultaneous installation of N- and O-functionality to construct unexplored N–O heterocyclic and amino-alcohol scaffolds. This transformation uses earth abundant copper salts to facilitate the formation of a carbon-centered radical and subsequent carbon–nitrogen bond formation. The intermediate aminoxyl radical is terminated by an intramolecularly appended carbon-centered radical. We have exploited this methodology to also access amino-alcohols with a range of aliphatic and aromatic linkers.

## Abstract 2

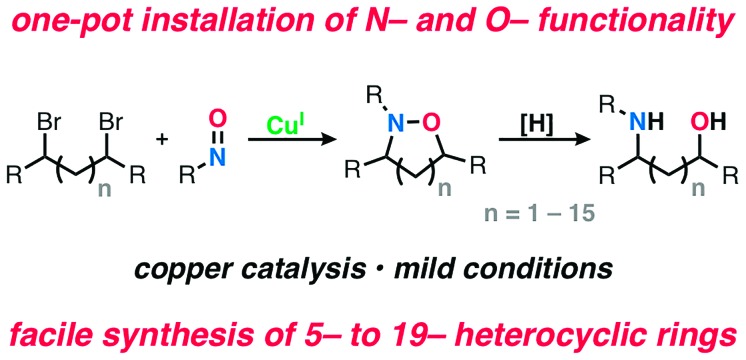
Synthetic chemists have spent considerable effort optimizing the synthesis of nitrogen and oxygen containing compounds through a number of methods; however, direct introduction of N- and O-functionality remains challenging.

## 

Historically, cycloaddition reactions have provided an efficient strategy to build molecular complexity into heterocycles by taking advantage of multiple bond formations in a single step. Given the ubiquity of C–N and C–O bonds in biologically active molecules, the ability of nitrones and nitroso compounds to directly install nitrogen and oxygen heteroatoms is of particular importance.^1^ Moreover, due to the labile nature of the N–O bond, these transformations have also served as strategic approaches for the synthesis of amino alcohols bearing a 1,3 or 1,4-relationship.^2^ Despite the prevalence of these transformations in organic synthesis, most methods have been restricted to the construction of isoxazoline (Scheme 1A)^3^ and 1,2-oxazine (Scheme 1B)^4^ heterocyclic scaffolds and the corresponding amino alcohols upon reduction. To date, there is no unified method for the synthesis of N–O heterocycles with varying ring sizes (small to large) or a direct approach to construct amino-alcohols with spatial control without independently installing the N- and O-functionality in sequential steps.

**Scheme 1:**
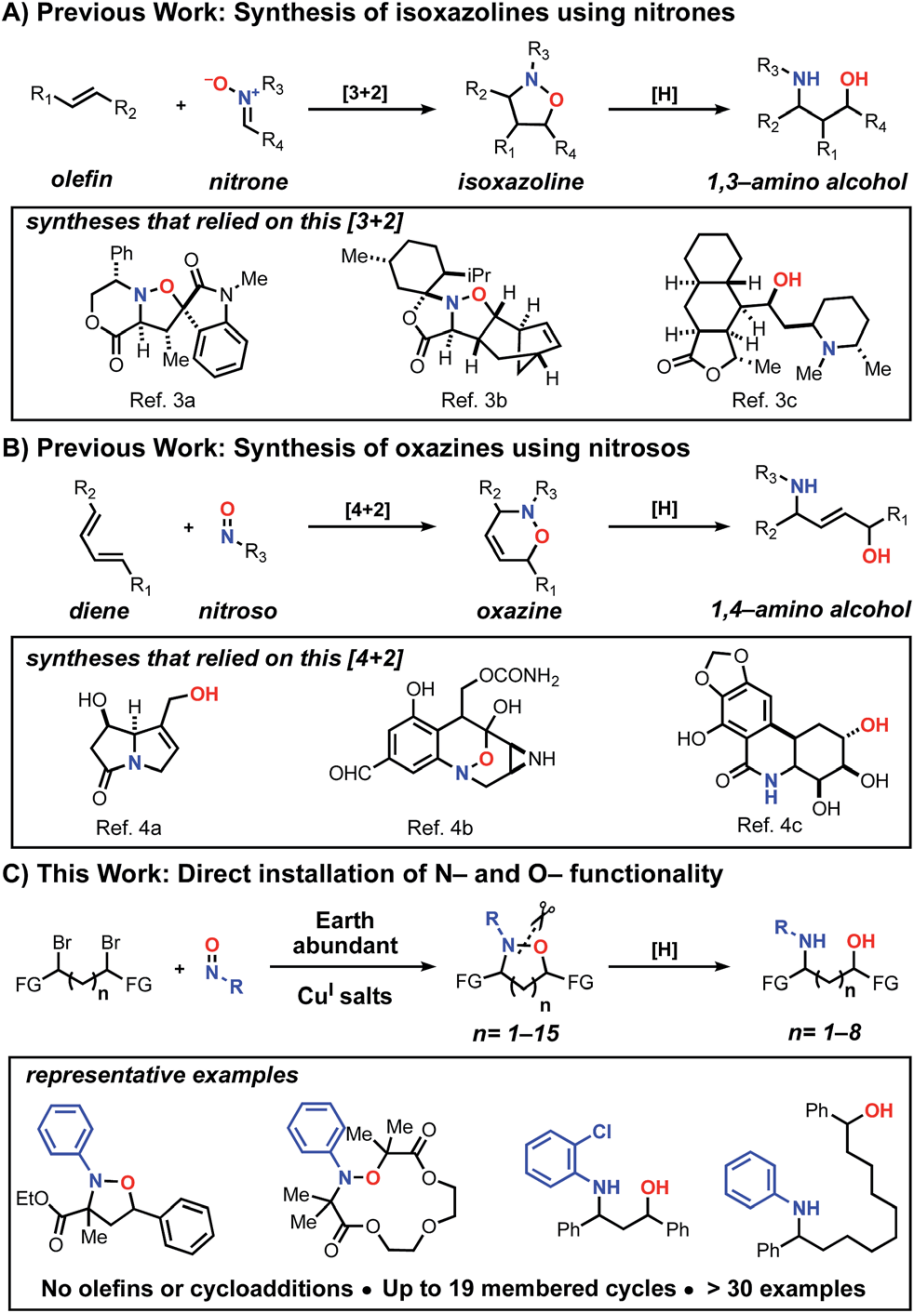
Examples of strategies that enable the direct installation of nitrogen and oxygen heteroatoms and examples of biologically active products that relied on these methods.

Previously, in an effort to provide alternatives to cycloaddition reactions or electrophilic functionalization of carbonyls using nitroso compounds, we^5^ and others^6^ described the use of radical transformations with nitroso compounds to construct sterically hindered amines. This process employs earth abundant copper salts, tolerates a range of functional groups and employs widely available radical precursors. Here we report the development of a generalized method to construct N–O heterocycles and amino-alcohols of any size and distribution. These studies demonstrate that bi-molecular reactions are not necessary to trap the *in situ* generated aminoxyl radical, despite the well-known challenges of forming larger macrocyclic rings.^7^ In addition and in contrast to our previous work, this method also increases the atom economy of the nitroso additions, accessing products that incorporate both heteroatoms. Previously, the C–O bond constructed during the radical transformation was treated as part of the waste stream and discarded upon N–O bond cleavage. Combined, this radical-based process provides efficient entry into many unexplored scaffolds (Scheme 1C).

To begin our investigations, we examined the intramolecular reaction of the 1,3-dibromide scaffold. Initially, we were able to identify conditions inspired by our previous work and others^8^ (5 equiv. of both Cu^I^ and Cu^0^, 2.5 equiv. of PMDTA, 2 equiv. of nitrosobenzene, THF, 40 °C) that afforded the desired N–O heterocycle **1** in 70% yield. We were further encouraged to find that, in a two-step-one-pot approach, the heterocycle could be reduced to the corresponding amino-alcohol **15** (65% overall yield) by simply adding additional Cu^I^ and ascorbic acid. Furthermore, the heterocycle was formed in a 2 : 1 ratio of diastereomers (dr), favouring the *cis* isomer over the *trans*, and the N–O bond reduction did not erode the selectivity. Through optimization of the reaction parameters, we found that Cu^0^ could be removed entirely, Cu^I^ loading could be reduced to 2 equivalents, and nitrosobenzene loading could be lowered to 1.5 equivalents (see ESI, Table S2†). These modifications increased the yield of the desired product (**1**) to 85%. Unfortunately, we discovered that reduction of the N–O heterocycle with Cu^I^ and ascorbic acid was only useful for five-membered ring heterocycles, with incomplete reduction occurring when larger rings were investigated. A screen of various reducing conditions revealed that stronger reducing agents such as zinc in HCl and sodium-naphthalenide afforded the desired amino alcohol **15** in higher yield (67% isolated yield over two steps using Zn/HCl conditions) and these methods proved general. Notably during optimization studies, we discovered that increasing the reaction temperature to 50 °C increased the dr of this transformation to 5 : 1 favoring the *cis*-isomer. Finally, a copper ligand screen was investigated; reactions run with the more activating ligands such as Me_6_TREN provided yields very similar to those run with PMDTA. However, using a less activating ligand, such as 2,2′-bipyridyl, resulted in limited or no conversion of the starting material.

With optimized conditions established, we initially explored the generality of this method to construct N–O heterocycles with varying ring sizes (Fig. 1A). Five (**1**) and six (**2**) membered rings were synthesized in good yields with the optimized conditions. The seven-membered ring (**3**) required more dilute reaction conditions, as increasing amounts of oligomers were observed by ^1^H-NMR spectroscopy, presumably formed *via* a competitive intermolecular radical termination. The larger 8–12 membered heterocycles (**4–7**) required the same dilute reaction conditions, as well as the addition of 5 mol% copper(ii) bromide relative to the copper(i) bromide. Cu^II^ is known to have a strong effect on the kinetics of atom transfer radical polymerization (ATRP) systems^9^ and we hypothesize that the addition of Cu^II^ decreases the concentration of carbon-centered radicals, leading to a more controlled reaction. As expected when forming larger macrocyclic ring systems, the stereoselectivity of the transformation decreases as the spacer length increases. The five-membered ring **1** demonstrated a relatively high dr of 5 : 1 *cis*:*trans*, while the six- and seven-membered rings **2** and **3** demonstrated dr's of 1.8 : 1 and 1.5 : 1, respectively.^10^ Rings eight-membered and greater demonstrated no selectivity. Alkyl-nitroso compounds were used to create heterocycles with yields similar to their aromatic counterparts; compound **8** was synthesized using the commercially available 2-methyl-2-nitrosopropane dimer. We were pleased to find that the intramolecular reaction could be extended to readily available α-bromo carbonyl-based scaffolds. Impressively, as shown in Fig. 1B, these scaffolds were found to cyclize very efficiently, creating up to 19-membered heterocycles in great yield (**9–14**). Overall, these results open the door for efficient access to a series of unexplored N–O based heterocyclic scaffolds.

**Fig. 1:**
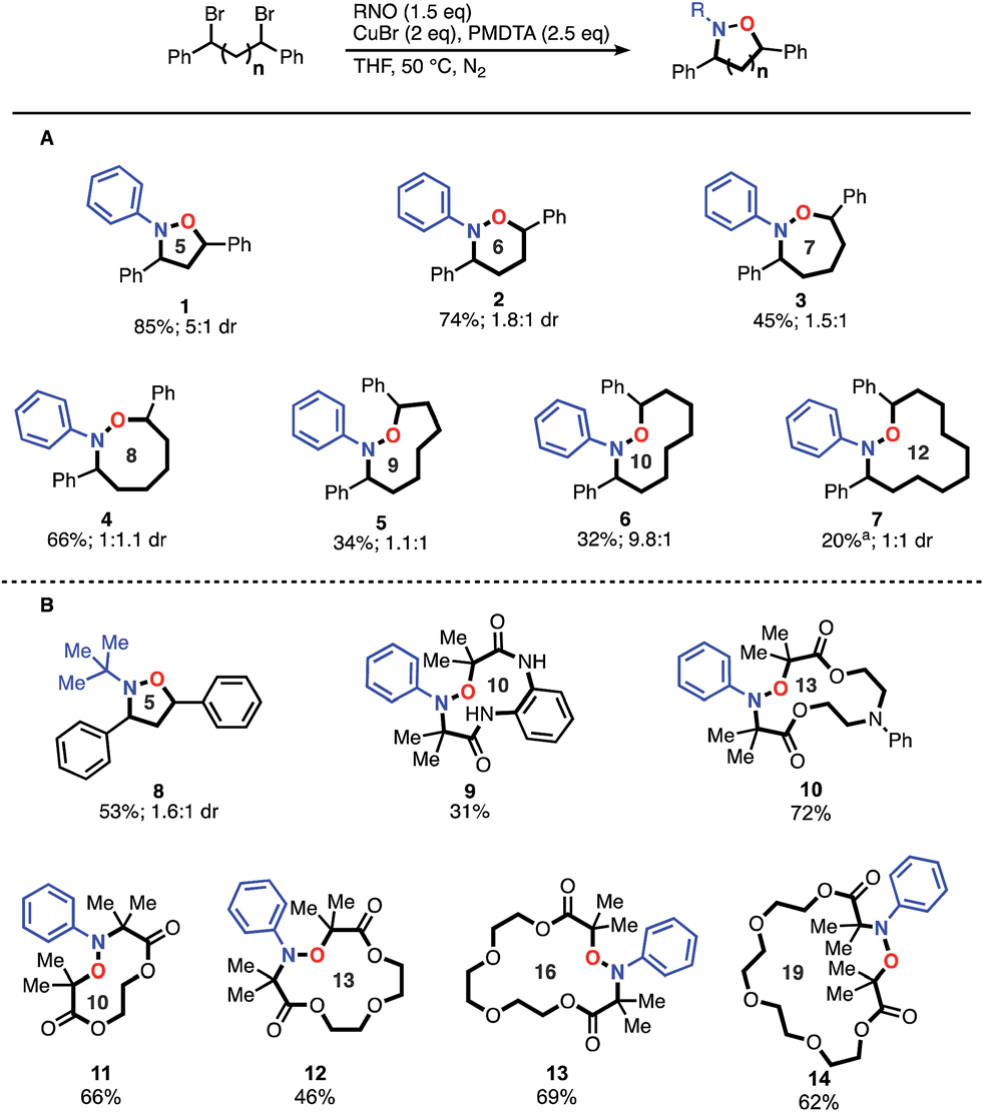
Scope of N–O heterocycle synthesis. (A) Products derived from benzyl dibromide scaffolds and nitrosobenzene. (B) Products derived from α-bromocarbonyl compounds and nitroso benzene or the 2-methyl-2-nitrosopropane dimer. ^a^Yield established through an internal standard. Isolated yield of **7** is 10%.

We were intrigued by the large discrepancy in yields between the glycol-linked **10–14** and the alkyl-linked **1–5** substrates, and considered that a Cu^II^ templating effect was responsible. Cu^II^ has been employed advantageously in a number of similar cyclizations.^11^ To test this hypothesis, we synthesized an alkyl-linked 18-membered heterocycle that cannot benefit from templating and subjected it to optimized conditions (see ESI, S25†). Compared to the closest derivatives, compounds **13** and **14**, the yield dropped from greater than 60% to 32%. This direct comparison indicates that a templating effect might be responsible for the increased yields observed with substrates **10–14**.

After demonstrating the construction of N–O heterocycles with spatial control, we were now set to examine the scope of our two-step-one-pot approach to construct amino-alcohols of various distributions (Fig. 2). We were pleased to find that many of the yields are actually higher for the amino-alcohols using the two-step-one-pot approach than those of the corresponding N–O heterocycle. For example, synthesis of an amino-alcohol (**21**) bearing a 1–10 relationship, which represents the direct installation of both N- and O-functionality over 12 angstroms of space, afforded the desired product in 48% overall yield. Notably, this is 20% higher than that of the corresponding N–O heterocycle (**7**, 20% yield). We speculate this is due to the *in situ* reduction of oligomers that also afford the desired amino-alcohol product **21**. Previously, the oligomers were removed during the heterocycle purification and isolation. For the *in situ* reduction, the five-membered heterocycle affording amino-alcohol **15** and **22** was reduced using Zn/HCl conditions, but all others were reduced using sodium-naphthalenide.

**Fig. 2:**
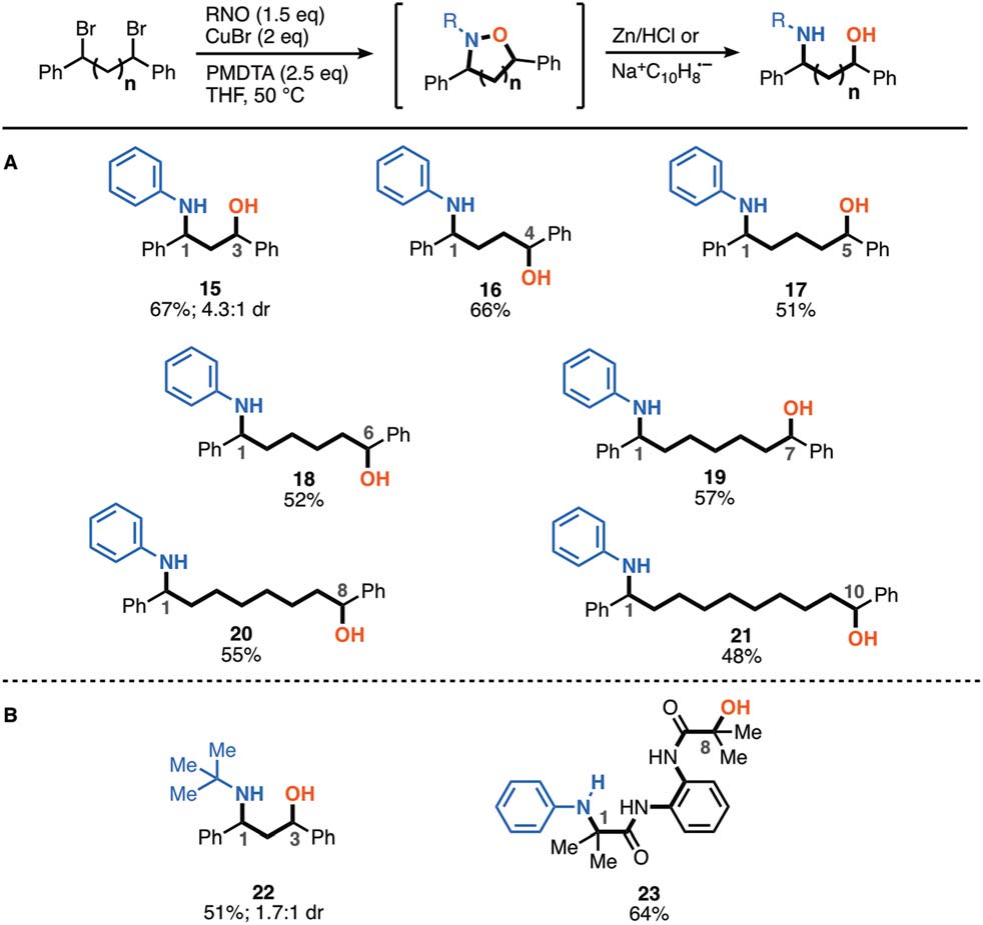
Scope of amino-alcohols synthesized with a one-pot-two-step approach. (A) Products derived from benzyl dibromide scaffolds and nitrosobenzene. (B) Products derived from benzyl dibromide and the 2-methyl-2-nitrosopropane dimer or α-bromocarbonyl compounds and nitroso benzene.

Next, we explored how structural modifications to the nitrosoarene and the dibromide architecture were tolerated. Given the higher yields of amino-alcohol synthesis, the two-step-one-pot approach was used for these studies. A small library of both electron-rich and deficient nitrosoarenes was synthesized and subjected to the optimized conditions (Fig. 3A). With respect to the nitrosoarene coupling partner, the reaction was tolerant of electronic changes. Not surprisingly, the reaction tolerates halogenated compounds **26** and **28** that allow for facile downstream functionalization. Of note, the amine functional group (NH_2_) group in substrate **27** is derived from the corresponding nitro group and was generated *in situ* upon treatment with zinc and HCl conditions. Moreover, structural changes can be made to the dibromide scaffold, either the methylene linker or the aromatic rings, affording the anticipated product in moderate to good yields (40% to 66%) (Fig. 3B). Notably, the reaction efficiency decreased slightly when *gem*-dimethyl groups are introduced alpha to the dibromide (**30**). This is not surprising considering the costly steric interactions of forming C–N and C–O bonds adjacent to a quaternary center. Interestingly, while most of the modifications to the scaffold had limited effect on the diastereomeric ratio of the products (∼3 : 1 dr ratio was observed for **29–31**, **33**), compound **32** was formed in a 10 : 1 dr, suggesting that diastereoselectivity can be enhanced using substitution at the *meta*-position.

**Fig. 3:**
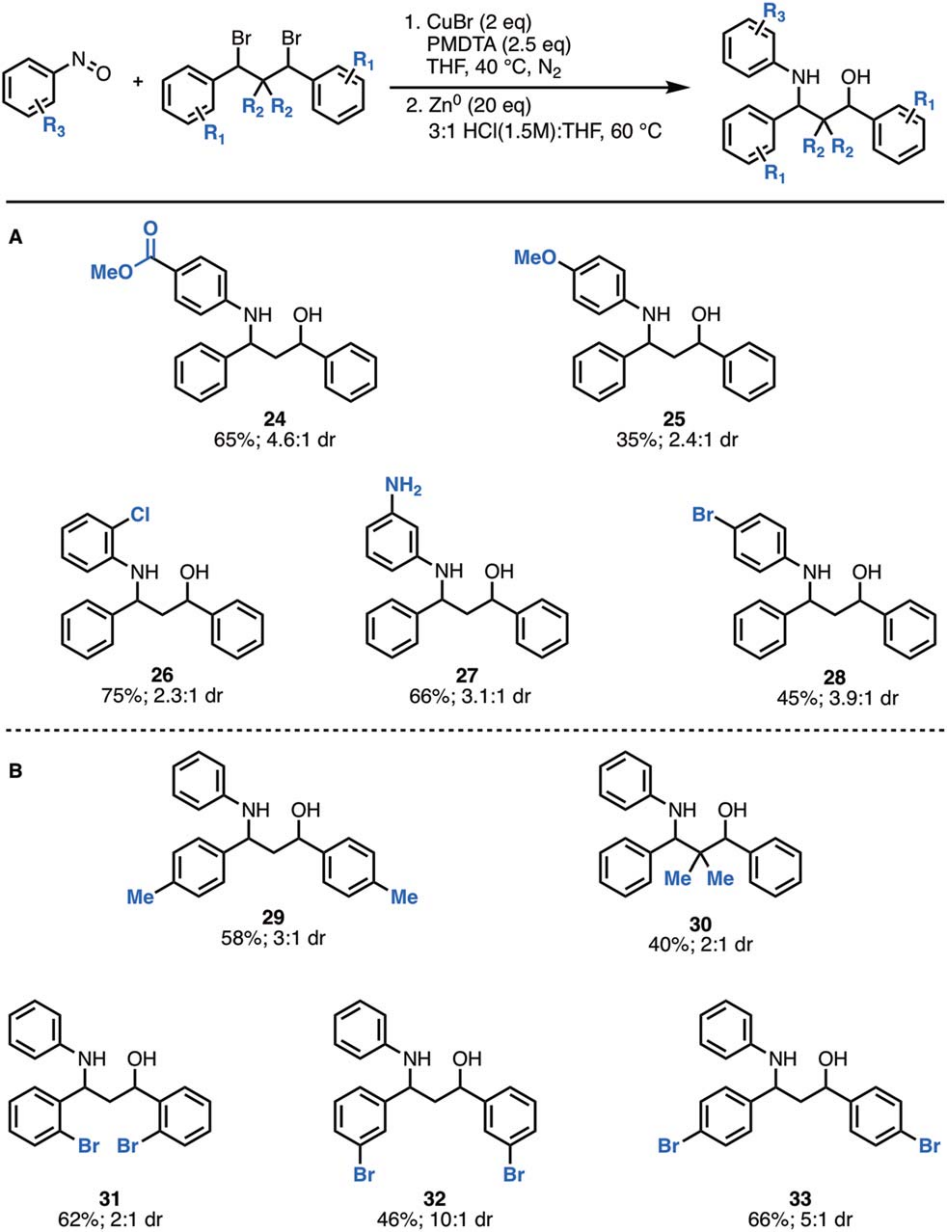
Scope of scaffold modifications. (A) Substrates derived from functionalized nitrosoarenes. (B) Substrates derived from modified dibromide scaffolds.

To demonstrate the synthetic utility of this methodology beyond symmetrical substrates, we investigated strategies to construct N- and O-bonds on unsymmetrical scaffolds with regioselective control. A common feature of radical reactions with nitroso compounds is that the initial carbon centered radical reacts with nitrogen. Consequently, we hypothesized that radical initiation rates could be leveraged to control the regioselectivity. The success of this approach would also require a second intramolecular radical reaction with the intermediate aminoxyl radical to outcompete the intermolecular reaction. Despite the challenges of balancing the reaction rates of these highly reactive radical intermediates, we were encouraged by the wealth of literature on activation rates for various initiators used for ATRP.^12^ Guided by these activation studies, we designed a mixed-initiator scaffold containing both an α-bromoester and a benzyl-bromide radical precursor which could be synthesized in one step from styrene and ethyl dibromopropanoate (Fig. 4). The *k*_act_ of the α-bromoester moiety is roughly an order of magnitude greater than that of the benzyl bromide under standard ATRP conditions.^13^ Given this difference, we predicted that the initial radical would predominately form at the α-bromoester, leading to carbon–nitrogen bond formation α to the ester and carbon–oxygen bond formation at the less active benzyl site. To our gratification, subjection of the unsymmetrical scaffold to the optimized reaction conditions resulted in the N–O heterocycle with a 10 : 1 ratio of products **35** to **36** favouring the predicted major isomer. This result indicates that the major regioselectivity can be predicted through the relative *k*_act_ of each radical precursor; moreover, the approximate ratio of the regioisomers can be predicted from the ratio of the *k*_act_ of the initiators. Further studies are underway to elucidate these factors in more detail and explore the scope of unsymmetrical scaffolds.

**Fig. 4:**
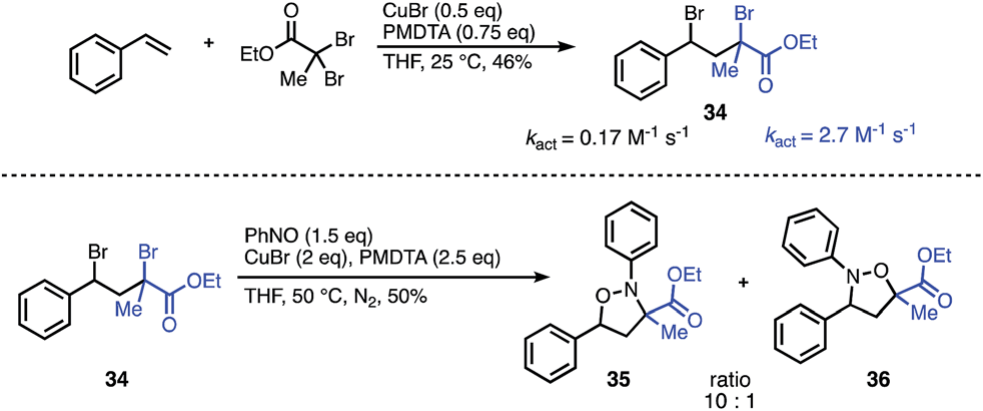
Regioselectivity of the nitroso addition onto an unsymmetrical scaffold can be predicted from relative *k*_act_.

## Conclusions

In summary, we have developed a new method for the direct installation of nitrogen and oxygen functionality where N–O heterocycles and amino-alcohol scaffold size are unencumbered by traditional olefin coupling reactions. The described method is general in terms of scope and provides an efficient method capable of construction macrocycles up to 19-members in size and amino-alcohols with up to 12 Å separating the N- and O-heteroatoms. The reaction is catalysed by copper salts and leverages readily available radical precursors and nitroso compounds to generate a new C–N bond and an intermediate aminoxyl radical, which is subsequently terminated with a second intramolecularly appended radical. Moreover, we have shown that the regioselectivity of the installation of nitrogen and oxygen functionality can be predicted using well-documented ATRP rate constants for radical formation. The method reported herein provides a new versatile platform for the development of N–O heterocycles and the corresponding amino-alcohols, all with high atom economy and earth-abundant catalysts.

## Conflicts of interest

There are no conflicts to declare.

## Supplementary Material

Supplementary information
