# Supplementary material for: Direct introduction of nitrogen and oxygen functionality with spatial control using copper catalysis
Source: Chem Sci. 2018 Sep 17;9(46):8748–52. doi: 10.1039/c8sc03288b (PMC6289103; doi:10.1039/c8sc03288b)
Supplement: Supplementary file 1 [file SC-009-C8SC03288B-s001.pdf]

Electronic Supplementary Material (ESI) for Chemical Science.
This journal is © The Royal Society of Chemistry 2018

Direct Introduction of Nitrogen and Oxygen Functionality with Spatial Control Using Copper Catalysis

James B. Shaum, David J. Fisher, Miranda M. Sroda, Luis Limon, Javier G. Read de Alaniz*
Department of Chemistry and Biochemistry, University of California, Santa Barbara, California 93106, USA

Supporting Information – Table of Contents

Materials and Methods……………………………………………………………………………2
General Experimental Procedures…………………………………………………………3
Relevant Optimization Data……………………………………………………………………6
Experimental Procedures and Data……………………………………………………….8
References……………………………………………………………………………………………36
^{1}H and ^{13}C NMR Spectra…………………………………………………………………37

Materials and Methods Unless stated otherwise, reactions were conducted in flame dried glassware under an atmosphere of N_2 using reagent grade solvents. All commercially obtained reagents were used as received. Reaction temperatures were controlled using a Heidolph temperature modulator, and unless stated otherwise, reactions were performed at room temperature (rt, approximately 23 °C). Thin-layer chromatography (TLC) was conducted with E. Merck silica gel 60 F254 pre-coated plates, (0.25 mm) and visualized by exposure to UV light (254 nm) or stained with potassium permanganate or anisaldehyde. Flash column chromatography was performed using normal phase silica gel (60 Å, 230-240 mesh, Geduran®). ^1H NMR spectra were recorded on Varian Spectrometers (at 400, 500 and 600 MHz) and are reported relative to deuterated solvent signals. Data for ^1H NMR spectra are reported as follows: chemical shift (δ ppm), multiplicity, coupling constant (Hz) and integration. ^{13}C NMR spectra were recorded on Varian Spectrometers (at 100, 125 and 150 MHz). Data for ^{13}C NMR spectra are reported in terms of chemical shift. IR spectra were recorded on a Perkin Elmer Spectrum 100 FT/IR and a Bruker Alpha FT/IR and are reported in terms of frequency of absorption (cm^{-1}). High resolution mass spectra were obtained from the UC Santa Barbara Mass Spectrometry Facility and the Materials Research Laboratory Polymer Characterization Facility at UC Santa Barbara.

Starting materials **S1** was prepared according to literature procedure and **S14–S18** using a modified procedure.[1] Starting materials **S2–S7** were prepared according to a literature procedure using a modified procedure.[2] Nitrosoarenes **S19–S22** were prepared according to modified literature procedures.[3,4]

General Procedure A for N–O Heterocycles (5-6 membered rings)
To a 25 mL oven dried round bottom flask is added CuBr (28 mg, 0.2 mmol, 2 eq) and PMDTA (52 µL, 0.25 mmol, 2.5 eq) in THF (anhydrous, 7 mL). This suspension is sonicated for one minutes and sparged with nitrogen for seven minutes. A second solution of nitrosobenzene (16 mg, 0.15 mmol, 1.5 eq) and dibromide (0.1 mmol, 1 eq) is prepared in THF (anhydrous, 3 mL) in a 10 mL conical flask. This solution is sparged with nitrogen for three minutes. The copper suspension is stirred at 50 °C under nitrogen while the nitroso solution is added dropwise to the copper suspension via syringe pump over ten hours. Upon completion of addition, the reaction is allowed to continue stirring under nitrogen for an additional six hours.

The solution is concentrated *in vacuo* and reconstituted in DCM (10 mL). The DCM solution is washed with EDTA (0.5 M, pH 8, 20 mL) and extracted with DCM (3 x 15 mL). The organic layers are combined and sequentially dried with brine (20 mL) and sodium sulfate. The crude product was dry loaded onto Celite and purified via flash chromatography with an increasing gradient of DCM in hexane (0 —> 35%). Diastereomeric ratio was determined from the crude ^{1}H NMR.

General Procedure B for N–O Heterocycles (7 membered rings)
To a 100 mL oven dried round bottom flask is added CuBr (28 mg, 0.2 mmol, 2 eq) and PMDTA (52 µL, 0.25 mmol, 2.5 eq) in THF (anhydrous, 30 mL). This suspension is sonicated for one minutes and sparged with nitrogen for thirty minutes. A second solution of nitrosobenzene (16 mg, 0.15 mmol, 1.5 eq) and dibromide (0.1 mmol, 1 eq) is prepared in THF (anhydrous, 10 mL) in a 10 mL conical flask. This solution is sparged with nitrogen for ten minutes. The copper suspension is stirred at 50 °C under nitrogen while the nitroso solution is added dropwise to the copper suspension via syringe pump over ten hours. Upon completion of addition, the reaction is allowed to continue stirring under nitrogen for an additional six hours.

The solution is concentrated *in vacuo* and reconstituted in DCM (10 mL). The DCM solution is washed with EDTA (0.5 M, pH 8, 20 mL) and extracted with DCM (3 x 15 mL). The organic layers are combined and sequentially dried with brine (20 mL) and sodium sulfate. The crude product was dry loaded onto Celite and purified via flash chromatography with an increasing gradient of DCM in hexane (0 —> 35%). Diastereomeric ratio was determined from the crude ^{1}H NMR.

General Procedure C for N–O Heterocycles (8 membered ring)
To a 100 mL oven dried round bottom flask is added CuBr (28 mg, 0.2 mmol, 2 eq), $CuBr_2$ (4 mg, 0.02 mmol, 10 mol% relative to CuBr) and PMDTA (52 µL, 0.25 mmol, 2.5 eq) in THF (anhydrous, 30 mL). This suspension is sonicated for one minutes and sparged with nitrogen for thirty minutes. A second solution of nitrosobenzene (16 mg, 0.15 mmol, 1.5 eq) and dibromide (0.1 mmol, 1 eq) is prepared in THF (anhydrous, 10 mL) in a 10 mL conical flask. This solution is sparged with nitrogen for ten minutes. The copper suspension is stirred at

50 °C under nitrogen while the nitroso solution is added dropwise to the copper suspension via syringe pump over ten hours. Upon completion of addition, the reaction is allowed to continue stirring under nitrogen for an additional six hours.

The solution is concentrated *in vacuo* and reconstituted in DCM (10 mL). The DCM solution is washed with EDTA (0.5 M, pH 8, 20 mL) and extracted with DCM (3 x 15 mL). The organic layers are combined and sequentially dried with brine (20 mL) and sodium sulfate. The crude product was dry loaded onto Celite and purified via flash chromatography with an increasing gradient of DCM in hexane (0 —> 35%). Diastereomeric ratio was determined from the crude ^1H NMR.

General Procedure D for N–O Heterocycles (9+ membered rings)
To a 100 mL oven dried round bottom flask is added CuBr (28 mg, 0.2 mmol, 2 eq), $CuBr_2$ (4 mg, 0.02 mmol, 10 mol% relative to CuBr) and PMDTA (52 µL, 0.25 mmol, 2.5 eq) in THF (anhydrous, 30 mL). This suspension is sonicated for one minutes and sparged with nitrogen for thirty minutes. A second solution of nitrosobenzene (16 mg, 0.15 mmol, 1.5 eq) and dibromide (0.1 mmol, 1 eq) is prepared in THF (anhydrous, 10 mL) in a 10 mL conical flask. This solution is sparged with nitrogen for ten minutes. The copper suspension is stirred at 50 °C under nitrogen while the nitroso solution is added dropwise to the copper suspension via syringe pump over ten hours. Upon completion of addition, the reaction is allowed to continue stirring under nitrogen for an additional eight hours.

The solution is concentrated *in vacuo* and reconstituted in DCM (10 mL). The DCM solution is washed with EDTA (0.5 M, pH 8, 20 mL) and extracted with DCM (3 x 15 mL). The organic layers are combined and sequentially dried with brine (20 mL) and sodium sulfate. The crude solution is loaded onto a prep TLC plate and purified with an isocratic mobile phase of 3:7 DCM:Hexane. Diastereomeric ratio was determined from the crude ^1H NMR.

General Procedure for N–O Bond Reduction *via* Zn/HCl
A 1-dram vial containing the resulting crude N–O alkylated adduct and a stir bar was charged with activated Zn^0 (130 mg, 2 mmol, 20 equiv) and 4:1 solution of 1.5 M HCl and THF (1.5 mL) and heated to 60 °C. The solution was stirred until consumption of the starting material was observed by TLC, upon which the crude reaction mixture was quenched with an aqueous solution of saturated $NaHCO_3$ (20 mL). The resulting precipitate was removed through vacuum filtration and the filtrate was extracted with EtOAc (3x 15 mL). The combined organic layers were washed with water (1x 15 mL) and once with brine (1x 15 mL) and then dried over sodium sulfate. The crude product was dry loaded onto Celite and purified via flash chromatography with an increasing gradient of DCM in hexane (0 —> 50%)

General Procedure for N–O Bond Reduction *via* Sodium Naphthalenide
A 500 mM solution of Sodium Naphthalenide was freshly prepared in THF while the substrate to be reduced was dissolved in 1 mL of THF in a 1-dram vial and briefly degassed with N_2. The napthalenide was titrated slowly until the substrate solution remained dark green. After three minutes, the reaction was quenched with isopropanol (~5 mL), washed with saturated sodium bicarbonate (10 mL) and extracted with ethyl acetate (2 x 10 mL).

Organic layers were combined and dried sequentially with brine (10 mL) and sodium sulfate. The crude product was dry loaded onto Celite and purified via flash chromatography with an increasing gradient of DCM in hexane (0 —> 50%).

Relevant Optimization Data

Table S1: Relevant heterocycle synthesis optimization data

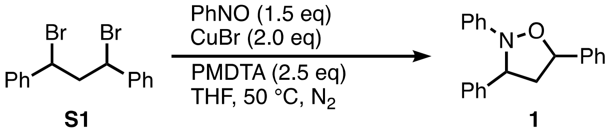

entry	deviation from conditions	temp (°C)	result
1	5 eq CuBr, 5 eq Cu^0, 2 eq PhNO	40	70%
2	5 eq Cu^0, 5 eq PhNO	40	46%
3	5 eq CuBr, 1 eq PhNO	40	66%
4	5 eq CuBr, 2 eq PhNO	40	80%
5	5 eq CuBr, 1.5 eq PhNO	40	85%
6	None	40	84%
7	1.5 eq CuBr, 1.5 eq PhNO	40	75%
**8**	**None**	**50**	**85%**
9	MeCN as solvent	40	66%
10	DMF as solvent	40	61%
11	2.5 eq Me_6TREN	40	82%
12	2.5 eq bpy	40	>1%
13	2.5 eq ^{tBu}bpy	40	>1%

ATRP Ligands used in this screen

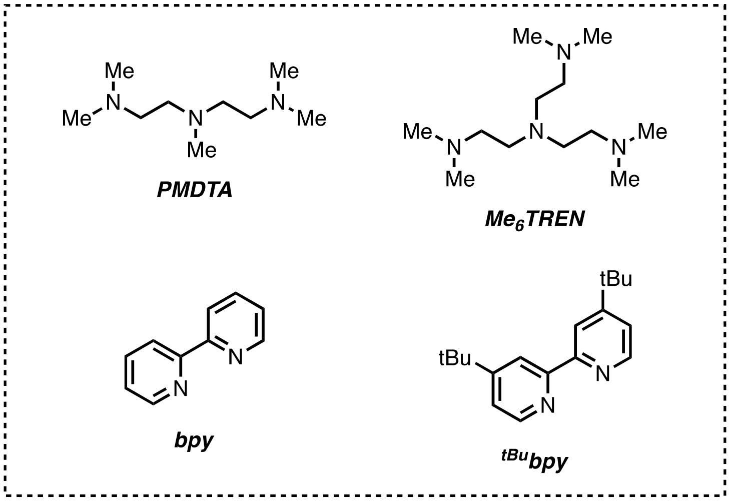

Table S2: Relevant data from the two-step-one-pot optimization

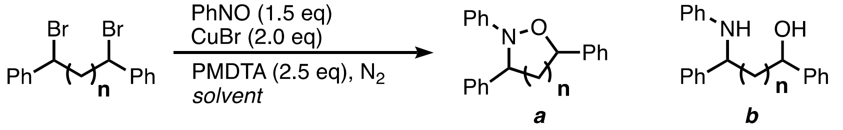

entry	deviation from conditions	n	a	b
1	THF, 50 °C, 18 hr	1	85%	0%
2	EtOH, 50 °C, 18 hr	1	42%	36%
3	EtOH, 50 °C, 48 hr	1	12%	68%
4	EtOH, 50 °C, 4 eq CuBr, 38 hr	1	0%	71%
5	EtOH, reflux, 2 eq CuBr, 14 hr	1	0%	73%
6	EtOH, reflux, 1 eq CuBr, 14 hr	1	0%	68%
7	EtOH, reflux, 2 eq CuBr, 14 hr	2	56%	0%
8	EtOH, reflux, 2 eq CuBr, 14 hr	3	43%	0%
9	THF, 50 °C, 16 hr; then 2 eq AscOH, excess Cu^0 in 1.5 mL MeCN, 80 °C, 14 hr	1	0%	65%
10	DMF, 40 °C, excess Cu^0, 16 hr; then 5 eq AscOH, DMF, 80 °C, 24 hr	1	6%	61%
11	MeCN, 40 °C, 16 hr; then 5 eq AscOH, 2 eq Cu^0, 80°C, 8hr	1	0%	64%
12	MeCN, 40 °C, 16 hr; then 5 eq AscOH, 2 eq Cu^0, 80°C, 8hr	2	35%	0%
13	MeCN, 40 °C, 16 hr; then 5 eq AscOH, 2 eq Cu^0, 80°C, 8hr	3	51%	0%
13	MeCN, 40 °C, 16 hr; then 5 eq AscOH, 2 eq Cu^0, 80°C, 8hr	4	24%	0%

Table S3: Temperature and solvent effects on diastereoselectivity

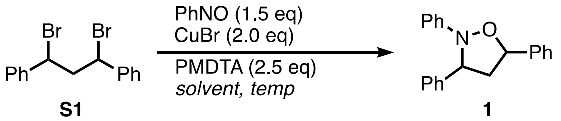

S1 → **1**

entry	solvent	temp (°C)	dr of product
1	MeCN	40	1.5:1
2	THF	40	4:1
3	THF	50	5:1

Experimental Procedures and Data:

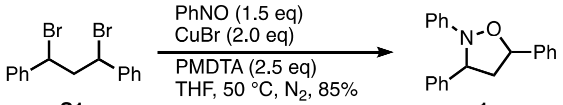

S1 1

2,3,5-triphenylisoxazolidine (1): To a 25 mL oven dried round bottom flask is added CuBr (28 mg, 0.2 mmol, 2 eq) and PMDTA (52 µL, 0.25 mmol, 2.5 eq) in THF (anhydrous, 7 mL). This suspension is sonicated for one minutes and sparged with nitrogen for seven minutes. A second solution of nitrosobenzene (16 mg, 0.15 mmol, 1.5 eq) and dibromide **S1** (35 mg, 0.1 mmol, 1 eq, 1.1:1 mix of diastereomers) is prepared in THF (anhydrous, 3 mL) in a 10 mL conical flask. This solution is sparged with nitrogen for three minutes. The copper suspension is stirred at 50 °C under nitrogen while the nitroso solution is added dropwise to the copper suspension via syringe pump over ten hours. Upon completion of addition, the reaction is allowed to continue stirring under nitrogen for an additional six hours.

The solution is concentrated *in vacuo* and reconstituted in DCM (10 mL). The DCM solution is washed with EDTA (0.5 M, pH 8, 20 mL) and extracted with DCM (3 x 15 mL). The organic layers are combined and sequentially dried with brine (20 mL) and sodium sulfate. The crude product was dry loaded onto Celite and purified via flash chromatography with an increasing gradient of DCM in hexane (0 —> 35%) to afford **1** as a clear oil and a 5:1 mix of diastereomers (26 mg, 85%). ^{1}H NMR (500 MHz, Chloroform-*d*) δ 7.62 – 7.53 (m, 2H), 7.45 (d, *J* = 8.1 Hz, 2H), 7.43 – 7.17 (m, 8H), 7.13 – 6.91 (m, 3H), 5.38 (dd, *J* = 9.0, 6.5 Hz, 1H minor), 5.20 (dd, *J* = 10.1, 5.7 Hz, 1H major), 4.94 (t, *J* = 7.9 Hz, 1H major), 4.70 (dd, *J* = 9.1, 4.6 Hz, 1H minor), 3.20 (ddd, *J* = 12.2, 8.0, 5.7 Hz, 1H major), 2.81 (dt, *J* = 12.2, 9.0 Hz, 1H minor), 2.69 (ddd, *J* = 12.3, 6.6, 4.6 Hz, 1H minor), 2.50 (ddd, *J* = 12.2, 10.2, 7.8 Hz, 1H major); ^{13}C NMR (125 MHz, Chloroform-*d*) δ 152.6, 142.9, 137.9, 129.0, 128.9, 128.8, 128.6, 128.6, 128.3, 128.2, 127.6, 127.4, 126.9, 126.8, 126.8, 126.3, 121.9, 121.4, 115.9, 114.0, 80.6, 78.8, 71.6, 69.8, 48.8, 47.3; IR (thin film) 3061, 3029, 2940, 2878, 1596, 1487, 1451, 1248, 1026, 753, 696 cm^{-1}; HRMS (ESI), calculated for $C_{21}H_{19}NO$: (M+Na$^+$) 324.1364, observed 324.1369. [56]

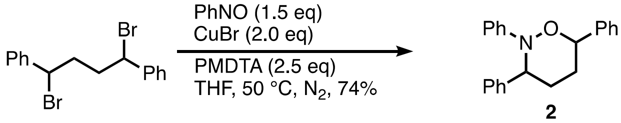

S2 2

2,3,6-triphenyl-1,2-oxazinane (**2**): To a 25 mL oven dried round bottom flask is added CuBr (28 mg, 0.2 mmol, 2 eq) and PMDTA (52 µL, 0.25 mmol, 2.5 eq) in THF (anhydrous, 7 mL). This suspension is sonicated for one minutes and sparged with nitrogen for seven minutes. A second solution of nitrosobenzene (16 mg, 0.15 mmol, 1.5 eq) and dibromide **S2** (37 mg, 0.1 mmol, 1 eq) is prepared in THF (anhydrous, 3 mL) in a 10 mL conical flask. This solution is sparged with nitrogen for three minutes. The copper suspension is stirred at 50 °C under nitrogen while the nitroso solution is added dropwise to the copper suspension via syringe pump over ten hours. Upon completion of addition, the reaction is allowed to continue stirring under nitrogen for an additional six hours.

The solution is concentrated *in vacuo* and reconstituted in DCM (10 mL). The DCM solution is washed with EDTA (0.5 M, pH 8, 20 mL) and extracted with DCM (3 x 15 mL). The organic layers are combined and sequentially dried with brine (20 mL) and sodium sulfate. The crude product was dry loaded onto Celite and purified via flash chromatography with an increasing gradient of DCM in hexane (0 —> 50%) to afford **2** as a clear oil with a 1.8:1 mix of diastereomers (23 mg, 74%). *trans (minor) isomer:* ^{1}H NMR (400 MHz, Chloroform-*d*) δ 7.44 (d, *J* = 7.0 Hz, 2H), 7.35 (t, *J* = 7.4 Hz, 2H), 7.33 – 7.04 (m, 10H), 6.98 (d, *J* = 9.2 Hz, 1H), 5.19 (dd, *J* = 11.2, 2.3 Hz, 1H), 4.17 (dd, *J* = 11.2, 2.8 Hz, 1H), 2.45 – 2.30 (m, 1H), 2.23 – 2.10 (m, 2H), 2.04 – 1.92 (m, 1H); ^{13}C NMR (125 MHz, Chloroform-*d*) δ 149.8, 141.3, 141.1, 128.3, 128.3, 128.2, 128.0, 127.7, 127.4, 126.4, 125.6, 124.0, 81.4, 77.4, 77.1, 76.9, 76.8, 69.9, 33.8, 32.5; IR (thin film) 3052, 3020, 2909, 2840, 1529, 1484, 1447, 1059, 745, 692; HRMS (ESI), calculated for $C_{22}H_{21}NO$: (M+H$^+$) 316.1701, observed 316.1723. *cis (major) isomer*: ^{1}H NMR (500 MHz, Chloroform-*d*) δ 7.56 (d, *J* = 7.4 Hz, 2H), 7.47 – 7.24 (m, 7H), 7.20 (t, *J* = 7.4 Hz, 3H), 7.03 (d, *J* = 8.2 Hz, 2H), 6.82 (t, *J* = 7.3, 1.1 Hz, 1H), 5.15 (dd, *J* = 5.5, 2.5 Hz, 1H), 5.05 (dd, *J* = 11.2, 2.7 Hz, 1H), 2.54 (tt, *J* = 13.1, 5.1 Hz, 1H), 2.31 – 2.23 (m, 1H), 2.01 – 1.91 (m, 1H), 1.83 (ddt, *J* = 13.5, 4.3, 3.0 Hz, 1H); ^{13}C NMR (125 MHz, Chloroform-*d*) δ 148.9, 140.7, 140.3, 128.9, 128.4, 128.3, 128.1, 128.0, 126.6, 126.4, 120.2, 114.2, 80.5, 58.7, 30.0, 29.7, 27.3; IR (thin film) 3019, 2913, 1591, 1486, 1445, 1215, 1028, 936, 747; HRMS (ESI), calculated for $C_{22}H_{21}NO$: (M+H$^+$) 316.1701, observed 316.1723.

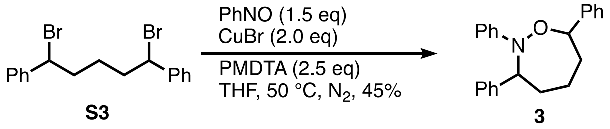

2,3,7-triphenyl-1,2-oxazepane (**3**): To a 100 mL oven dried round bottom flask is added CuBr (28 mg, 0.2 mmol, 2 eq) and PMDTA (52 µL, 0.25 mmol, 2.5 eq) in THF (anhydrous, 30 mL). This suspension is sonicated for one minutes and sparged with nitrogen for thirty minutes. A second solution of nitrosobenzene (16 mg, 0.15 mmol, 1.5 eq) and dibromide **S3** (38 mg, 0.1 mmol, 1 eq) is prepared in THF (anhydrous, 10 mL) in a 10 mL conical flask. This solution is sparged with nitrogen for ten minutes. The copper suspension is stirred at 50 °C under nitrogen while the nitroso solution is added dropwise to the copper suspension via syringe pump over ten hours. Upon completion of addition, the reaction is allowed to continue stirring under nitrogen for an additional six hours.

The solution is concentrated *in vacuo* and reconstituted in DCM (10 mL). The DCM solution is washed with EDTA (0.5 M, pH 8, 20 mL) and extracted with DCM (3 x 15 mL). The organic layers are combined and sequentially dried with brine (20 mL) and sodium sulfate. The crude product was dry loaded onto Celite and purified via flash chromatography with an increasing gradient of DCM in hexane (0 —> 50%) to afford **3** as a clear oil with a 1.5:1 mix of diastereomers (14 mg, 45%) ^{1}H NMR (500 MHz, Chloroform-*d*) δ 7.53 – 7.30 (m, 8H), 7.25 – 7.00 (m, 4H), 6.84 – 6.62 (m, 3H), 5.27 – 4.91 (m, 1H), 4.86 – 4.73 (m, 1H), 2.73 – 2.61 (m, 1H), 2.51 – 2.39 (m, 1H), 2.21 – 1.82 (m, 3H), 1.76 – 1.64 (m, 1H); ^{13}C NMR (125 MHz, Chloroform-*d*) δ 148.8, 142.8, 141.5, 128.7, 128.5, 128.4, 128.4, 128.3, 128.3, 127.6, 127.5, 127.4, 127.0, 126.9, 126.6, 126.3, 121.3, 119.1, 116.9, 113.5, 90.7, 88.6, 69.8, 66.4, 40.1, 38.2,

37.5, 36.3, 24.7, 20.9; IR (thin film) 3060, 3072, 2931, 2858, 1597, 1490, 1449, 748, 697 cm $^{-1}$; HRMS (ESI), calculated for $C_{23}H_{23}NO$: $(M+H^+)$ 330.1858, observed 330.1859.

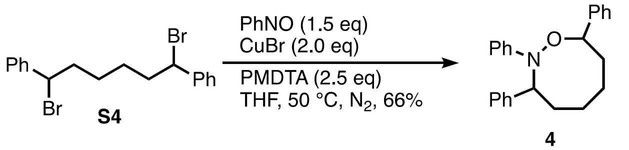

2,3,8-triphenyl-1,2-oxazocane (**4**): To a 100 mL oven dried round bottom flask is added CuBr (28 mg, 0.2 mmol, 2 eq), $CuBr_2$ (4 mg, 0.02 mmol, 10 mol% relative to CuBr) and PMDTA (52 μL, 0.25 mmol, 2.5 eq) in THF (anhydrous, 30 mL). This suspension is sonicated for one minutes and sparged with nitrogen for thirty minutes. A second solution of nitrosobenzene (16 mg, 0.15 mmol, 1.5 eq) and dibromide **S4** (40 mg, 0.1 mmol, 1 eq) is prepared in THF (anhydrous, 10 mL) in a 10 mL conical flask. This solution is sparged with nitrogen for ten minutes. The copper suspension is stirred at 50 °C under nitrogen while the nitroso solution is added dropwise to the copper suspension via syringe pump over ten hours. Upon completion of addition, the reaction is allowed to continue stirring under nitrogen for an additional six hours.

The solution is concentrated *in vacuo* and reconstituted in DCM (10 mL). The DCM solution is washed with EDTA (0.5 M, pH 8, 20 mL) and extracted with DCM (3 x 15 mL). The organic layers are combined and sequentially dried with brine (20 mL) and sodium sulfate. The crude product was dry loaded onto Celite and purified via flash chromatography with an increasing gradient of DCM in hexane (0 —> 35%) to afford **4** as a clear oil with a 1.1:1 mix of diastereomers (22.5 mg, 66%). ^{1}H NMR (500 MHz, Chloroform-*d*) δ 7.49 (d, *J* = 8.7 Hz, 2H), 7.42 (t, *J* = 7.7 Hz, 2H), 7.33 (d, *J* = 11.4 Hz, 3H), 7.15 (t, *J* = 7.0 Hz, 2H), 7.12 – 7.05 (m, 3H), 6.90 (d, *J* = 7.7 Hz, 2H), 6.73 (t, *J* = 7.0 Hz, 1H), 4.88 (dd, *J* = 6.7, 3.9 Hz, 1H), 4.71 (dd, *J* = 11.7, 3.8 Hz, 1H), 2.63 (q, *J* = 12.8 Hz, 1H), 2.29 (ddd, *J* = 14.8, 10.3, 3.8 Hz, 1H), 2.06 – 1.89 (m, 4H), 1.83 – 1.66 (m, 2H); ^{13}C NMR (125 MHz, Chloroform-*d*) δ 149.8, 141.9, 141.7, 128.6, 128.2, 127.9, 127.9, 127.3, 126.6, 126.5, 120.4, 116.0, 82.0, 70.2, 35.5, 33.4, 27.8, 23.6; IR (thin film) 3085, 3027, 2922, 2853, 1597, 1489, 1449, 1359, 755, 696 cm $^{-1}$; HRMS (ESI), calculated for $C_{24}H_{25}NO$: $(M+H^+)$ 344.2014, observed 344.2022.

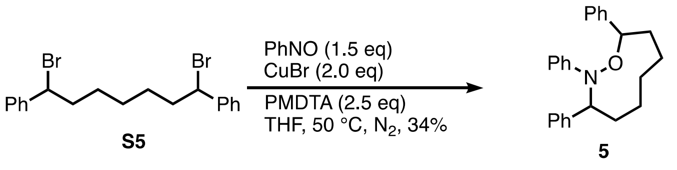

2,3,9-triphenyl-1,2-oxazonane (**5**): To a 100 mL oven dried round bottom flask is added CuBr (28 mg, 0.2 mmol, 2 eq) and PMDTA (52 μL, 0.25 mmol, 2.5 eq) in THF (anhydrous, 7 mL). This suspension is sonicated for one minutes and sparged with nitrogen for seven minutes. A second solution of nitrosobenzene (16 mg, 0.15 mmol, 1.5 eq) and dibromide **S5** (41 mg, 0.1 mmol, 1 eq) is prepared in THF (anhydrous, 3 mL) in a 10 mL conical flask. This solution is sparged with nitrogen for three minutes. The copper suspension is stirred at 50 °C under nitrogen while the nitroso solution is added dropwise to the copper suspension via

syringe pump over ten hours. Upon completion of addition, the reaction is allowed to continue stirring under nitrogen for an additional eight hours.

The solution is concentrated *in vacuo* and reconstituted in DCM (10 mL). The DCM solution is washed with EDTA (0.5 M, pH 8, 20 mL) and extracted with DCM (3 x 15 mL). The organic layers are combined and sequentially dried with brine (20 mL) and sodium sulfate. The crude solution is loaded onto a prep TLC plate and purified with an isocratic mobile phase of 3:7 DCM:Hexane to afford **5** as a clear oil with a 1.1:1 mix of diasteroemers (12 mg, 34%). *Major Diastereomer* **5a** ^{1}H NMR (500 MHz, Chloroform-*d*, 50 °C) δ 7.41 (d, *J* = 7.6 Hz, 2H), 7.36 (t, *J* = 7.9, 7.4 Hz, 2H), 7.31 – 7.23 (m, 1H), 7.22 – 7.18 (m, 2H), 7.17 – 7.06 (m, 6H), 6.89 (d, *J* = 7.4 Hz, 2H), 6.83 (tt, *J* = 7.5, 1.2 Hz, 1H), 4.77 (t, *J* = 3.8 Hz, 1H), 4.38 (dd, *J* = 12.3, 3.9 Hz, 1H), 2.66 – 2.55 (m, 1H), 2.47 (dq, *J* = 15.1, 4.2 Hz, 1H), 2.25 – 2.15 (m, 1H), 1.99 – 1.74 (m, 6H), 1.62 – 1.45 (m, 5H); ^{13}C NMR (125 MHz, Chloroform-*d,* 50 °C) δ 150.5, 142.1, 139.8, 128.6, 128.4, 128.0, 127.4, 126.8, 126.7, 126.1, 122.0, 118.9, 80.2, 73.5, 30.5, 28.4, 27.1, 22.3, 18.7; IR (thin film) 3018, 2917, 2839, 1590, 1481, 1446, 1256, 1026, 946, 805, 749; HRMS (ESI), calculated for $C_{25}H_{27}NO$: (M+H^{+}) 358.2171, observed 358.2219. *Minor Diastereomer* **5b** ^{1}H NMR (500 MHz, Chloroform-*d,* 50 °C) δ 7.22 – 7.05 (m, 10H), 6.94 (d, *J* = 4.5 Hz, 4H), 6.90 – 6.78 (m, 1H), 5.03 (dd, *J* = 7.7, 3.6 Hz, 1H), 4.31 (t, *J* = 5.1 Hz, 1H), 2.44 – 1.69 (m, 10H); ^{13}C NMR (125 MHz, Chloroform-*d,* 50 °C) δ 128.4, 127.7, 127.6, 127.5, 126.8, 126.7, 85.7, 74.4, 29.6, 27.5, 24.0, 22.8; IR (thin film) 3018, 2914, 2841, 1590, 1483, 1446, 1067, 1025, 761; HRMS (ESI), calculated for $C_{25}H_{27}NO$: (M+H^{+}) 358.2171, observed 358.2219.

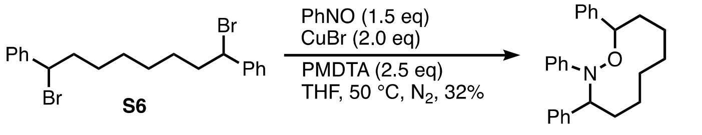

2,3,10-triphenyl-1,2-oxazecane (**6**): To a 100 mL oven dried round bottom flask is added CuBr (28 mg, 0.2 mmol, 2 eq), $CuBr_2$ (4 mg, 0.02 mmol, 10 mol% relative to CuBr) and PMDTA (52 µL, 0.25 mmol, 2.5 eq) in THF (anhydrous, 30 mL). This suspension is sonicated for one minutes and sparged with nitrogen for thirty minutes. A second solution of nitrosobenzene (16 mg, 0.15 mmol, 1.5 eq) and dibromide **S6** (42 mg, 0.1 mmol, 1 eq) is prepared in THF (anhydrous, 10 mL) in a 10 mL conical flask. This solution is sparged with nitrogen for ten minutes. The copper suspension is stirred at 50 °C under nitrogen while the nitroso solution is added dropwise to the copper suspension via syringe pump over ten hours. Upon completion of addition, the reaction is allowed to continue stirring under nitrogen for an additional eight hours.

The solution is concentrated *in vacuo* and reconstituted in DCM (10 mL). The DCM solution is washed with EDTA (0.5 M, pH 8, 20 mL) and extracted with DCM (3 x 15 mL). The organic layers are combined and sequentially dried with brine (20 mL) and sodium sulfate. The crude solution is loaded onto a prep TLC plate and purified with an isocratic mobile phase of 3:7 DCM:Hexane to afford **6** as a clear oil, isolated as a 9.8:1 mixture of diastereomers (12 mg, 32%). ^{1}H NMR (500 MHz, Chloroform-*d*) δ 7.17 – 7.04 (m, 1H), 6.93 – 6.74 (m, 9H), 6.69 – 6.52 (m, 4H), 4.71 (dd, *J* = 8.8, 3.2 Hz, 1H), 4.02 (dd, *J* = 6.5, 2.7 Hz, 1H), 2.00 (dtd, *J* = 15.2, 7.8, 2.7 Hz, 1H), 1.90 – 1.45 (m, 8H), 1.36 (dddt, *J* = 28.2, 14.4, 9.7, 5.2 Hz, 3H); ^{13}C NMR (125

MHz, Chloroform-*d*) δ 152.0, 142.7, 142.3, 128.7, 128.5, 128.2, 127.9, 127.6, 127.5, 127.4, 127.3, 126.9, 126.6, 126.4, 126.0, 125.0, 124.5, 120.5, 83.8, 78.9, 74.9, 74.4, 34.6, 32.3, 31.1, 30.9, 29.6, 26.7, 26.4, 26.1, 25.8, 23.5, 23.2, 22.4; IR (thin film) 3020, 2917, 2844, 1451, 1449, 1069, 1027, 753 cm $^{-1}$; HRMS (ESI), calculated for $C_{26}H_{29}NO$: $(M+H^+)$ 372.2327, observed 372.2343.

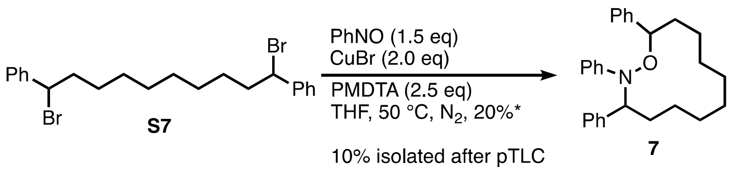

2,3,12-triphenyl-1-oxa-2-azacyclododecane (**7**): To a 100 mL oven dried round bottom flask is added CuBr (28 mg, 0.2 mmol, 2 eq), $CuBr_2$ (4 mg, 0.02 mmol, 10 mol% relative to CuBr) and PMDTA (52 µL, 0.25 mmol, 2.5 eq) in THF (anhydrous, 30 mL). This suspension is sonicated for one minutes and sparged with nitrogen for thirty minutes. A second solution of nitrosobenzene (16 mg, 0.15 mmol, 1.5 eq) and dibromide **S7** (45 mg, 0.1 mmol, 1 eq) is prepared in THF (anhydrous, 10 mL) in a 10 mL conical flask. This solution is sparged with nitrogen for ten minutes. The copper suspension is stirred at 50 °C under nitrogen while the nitroso solution is added dropwise to the copper suspension via syringe pump over ten hours. Upon completion of addition, the reaction is allowed to continue stirring under nitrogen for an additional eight hours.

The solution is concentrated *in vacuo* and reconstituted in DCM (10 mL). The DCM solution is washed with EDTA (0.5 M, pH 8, 20 mL) and extracted with DCM (3 x 15 mL). The organic layers are combined and sequentially dried with brine (20 mL) and sodium sulfate. NMR analysis of the crude sample versus an internal standard (0.1 mmol of dimethylterephthalate) indicated 20% conversion to the heterocycle. The crude solution is loaded onto a prep TLC plate and purified with an isocratic mobile phase of 3:7 DCM:Hexane to afford **7** as a clear oil with a 1:1 mix of diastereomers (4mg, 10%). ^{1}H NMR (500 MHz, Chloroform-*d*) δ 7.18 – 6.95 (m, 10H), 6.90 (t, *J* = 7.6 Hz, 2H), 6.83 – 6.74 (m, 3H), 4.85 (dd, *J* = 6.5, 4.2 Hz, 1H), 4.45 (dd, *J* = 7.6, 5.6 Hz, 1H), 2.27 (dq, *J* = 13.6, 6.6 Hz, 1H), 2.06 (dd, *J* = 13.2, 6.6 Hz, 1H), 1.94 (dq, *J* = 13.5, 6.7 Hz, 1H), 1.88 – 1.43 (m, 13H); ^{13}C NMR (125 MHz, Chloroform-*d*) δ 150.4, 142.3, 140.3, 129.0, 127.5, 127.5, 127.4, 126.8, 126.7, 126.6, 124.2, 123.4, 84.0, 72.6, 33.3, 32.7, 29.6, 26.4, 25.6, 24.7, 23.3, 22.9; IR (thin film) 3019, 2914, 2847, 1591, 1483, 1448, 1025, 752; HRMS (ESI), calculated for $C_{28}H_{33}NO$: $(M+H^+)$ 400.2640, observed 400.2672.

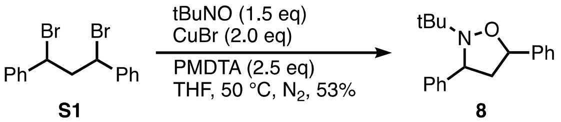

2-(*tert*-butyl)-3,5-diphenylisoxazolidine (**8**): To a 25 mL oven dried round bottom flask is added CuBr (28 mg, 0.2 mmol, 2 eq) and PMDTA (52 µL, 0.25 mmol, 2.5 eq) in THF (anhydrous, 7 mL). This suspension is sonicated for one minutes and sparged with nitrogen for seven minutes. A second solution of 2-methyl-2-nitrosopropane dimer (13 mg, 0.075

mmol, 1.5 eq) and dibromide **S1** (35 mg, 0.1 mmol, 1 eq, 1.1:1 mix of diastereomers) is prepared in THF (anhydrous, 3 mL) in a 10 mL conical flask. This solution is sparged with nitrogen for three minutes. The copper suspension is stirred at 50 °C under nitrogen while the nitroso solution is added dropwise to the copper suspension via syringe pump over ten hours. Upon completion of addition, the reaction is allowed to continue stirring under nitrogen for an additional fourteen hours.

The solution is concentrated *in vacuo* and reconstituted in DCM (10 mL). The DCM solution is washed with EDTA (0.5 M, pH 10, 20 mL) and extracted with DCM (3 x 15 mL). The organic layers are combined and sequentially dried with brine (20 mL) and sodium sulfate. The crude product was dry loaded onto Celite and purified via flash chromatography on a Yamazen brand amino-column with an increasing gradient of DCM in hexane (0 —> 35%) to afford **8** as a 1.6:1 mix of diastereomers (15 mg, 53%). ^{1}H NMR (500 MHz, Chloroform-*d*) δ 7.58 – 7.17 (m, 10H), 5.13 (ddd, *J* = 14.4, 9.9, 5.7 Hz, 1H), 4.53 – 4.33 (m, 1H), 2.96 (ddd, *J* = 12.2, 6.9, 5.3 Hz, 1H), 2.52 (dt, *J* = 12.1, 9.8 Hz, 1H), 2.44 (ddd, *J* = 12.1, 6.2, 3.9 Hz, 1H), 2.36 (dt, *J* = 12.2, 10.0 Hz, 1H), 1.15 (s, 4H), 1.13 (s, 5H); ^{13}C NMR (125 MHz, Chloroform-*d*) δ 145.4, 143.9, 139.9, 128.4, 128.3, 128.3, 128.3, 127.7, 127.7, 127.2, 127.0, 126.8, 126.8, 126.5, 126.4, 80.2, 64.5, 62.9, 60.3, 58.7, 51.2, 49.2, 26.7, 26.2; IR (thin film) 3061, 3028, 2968, 2925, 2870, 1451, 1214, 754, 698 ; HRMS (ESI), calculated for $C_{19}H_{23}NO$: (M+H$^+$) 282.1858, observed 282.1885.[7]

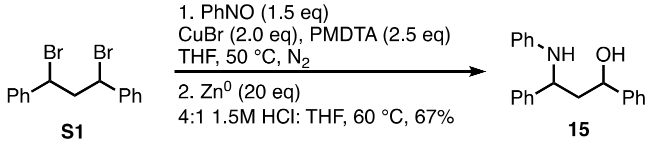

1,3-diphenyl-3-(phenylamino)propan-1-ol (**16**): To a 25 mL oven dried round bottom flask is added CuBr (28 mg, 0.2 mmol, 2 eq) and PMDTA (52 µL, 0.25 mmol, 2.5 eq) in THF (anhydrous, 7 mL). This suspension is sonicated for one minutes and sparged with nitrogen for seven minutes. A second solution of nitrosobenzene (16 mg, 0.15 mmol, 1.5 eq) and dibromide **S1** (35 mg, 0.1 mmol, 1 eq, 1.1:1 mix of diastereomers) is prepared in THF (anhydrous, 3 mL) in a 10 mL conical flask. This solution is sparged with nitrogen for three minutes. The copper suspension is stirred at 50 °C under nitrogen while the nitroso solution is added dropwise to the copper suspension via syringe pump over ten hours. Upon completion of addition, the reaction is allowed to continue stirring under nitrogen for an additional six hours.

The solution is concentrated *in vacuo* and reconstituted in DCM (10 mL). The DCM solution is washed with EDTA (0.5 M, pH 8, 20 mL) and extracted with DCM (3 x 15 mL). The organic layers are combined and sequentially dried with brine (20 mL) and sodium sulfate.

A 1-dram vial containing the resulting crude N–O alkylated adduct and a stir bar was charged with activated Zn^0 (130 mg, 2 mmol, 20 equiv) and 4:1 solution of 1.5 M HCl and THF (1.5 mL) and heated to 60 °C. The solution was stirred until consumption of the starting material was observed by TLC, upon which the crude reaction mixture was quenched with an aqueous solution of saturated $NaHCO_3$ (20 mL). The resulting precipitate was removed through

vacuum filtration and the filtrate was extracted with EtOAc (3x 15 mL). The combined organic layers were washed with water (1x 15 mL) and once with brine (1x 15 mL) and then dried over sodium sulfate. The crude product was dry loaded onto Celite and purified via flash chromatography with an increasing gradient of DCM in hexane (0 —> 50%) to afford **15** as a 4.3:1 mix of diastereomers (20 mg, 67%). ^{1}H NMR (600 MHz, CDCl3) δ 7.43 – 7.25 (m, 9H), 7.23 – 7.18 (m, 1H), 7.08 (q, J = 8.1 Hz, 2H), 6.72 – 6.61 (m, 1.36H major), 6.57 (d, J = 8.0 Hz, 0.66H minor), 6.52 (d, J = 8.0 Hz, 1H), 4.86 (dd, J = 9.2, 3.3 Hz, 1H), 4.65 – 4.53 (m, 1H), 2.28 (ddt, J = 18.4, 14.6, 9.7 Hz, 1H), 2.16 (ddd, J = 14.1, 8.4, 3.5 Hz, 0.36H minor), 2.05 (dt, J = 14.5, 4.2 Hz, 0.78H major); ^{13}C NMR (100 MHz, CDCl3) δ 129.1, 128.7, 128.6, 128.6, 127.8, 127.7, 127.2, 127.0, 126.3, 126.2, 125.7, 118.2, 114.5, 113.9, 73.8, 71.8, 58.2, 47.3, 46.6; IR (thin film) 3533, 3390, 3057, 3026, 2922, 2853, 1601, 1502, 749, 699 cm^{-1}; HRMS (ESI), calculated for $C_{21}H_{21}NO$: (M+H^{+}) 304.1701, observed 304.1706.[8]

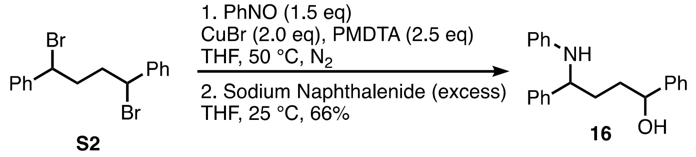

1,4-diphenyl-4-(phenylamino)butan-1-ol (**16**): To a 25 mL oven dried round bottom flask is added CuBr (28 mg, 0.2 mmol, 2 eq) and PMDTA (52 µL, 0.25 mmol, 2.5 eq) in THF (anhydrous, 7 mL). This suspension is sonicated for one minutes and sparged with nitrogen for seven minutes. A second solution of nitrosobenzene (16 mg, 0.15 mmol, 1.5 eq) and dibromide **S2** (37 mg, 0.1 mmol, 1 eq) is prepared in THF (anhydrous, 3 mL) in a 10 mL conical flask. This solution is sparged with nitrogen for three minutes. The copper suspension is stirred at 50 °C under nitrogen while the nitroso solution is added dropwise to the copper suspension via syringe pump over ten hours. Upon completion of addition, the reaction is allowed to continue stirring under nitrogen for an additional six hours.

The solution is concentrated *in vacuo* and reconstituted in DCM (10 mL). The DCM solution is washed with EDTA (0.5 M, pH 8, 20 mL) and extracted with DCM (3 x 15 mL). The organic layers are combined and sequentially dried with brine (20 mL) and sodium sulfate.

A 500 mM solution of Sodium Naphthalenide was freshly prepared in THF while the substrate to be reduced was dissolved in 1 mL of THF in a 1-dram vial and briefly degassed with N_2. The napthalenide was titrated slowly until the substrate solution remained dark green. After three minutes, the reaction was quenched with isopropanol (~5 mL), washed with saturated sodium bicarbonate (10 mL) and extracted with ethyl acetate (2 x 10 mL). Organic layers were combined and dried sequentially with brine (10 mL) and sodium sulfate. The crude product was dry loaded onto Celite and purified via flash chromatography with an increasing gradient of DCM in hexane (0 —> 50%) to afford **16** as a 2:1 mix of diastereomers (21 mg, 66%). ^{1}H NMR (500 MHz, Chloroform-*d*) δ 7.40 – 7.28 (m, 9H), 7.26 – 7.19 (m, 1H), 7.08 (ddt, *J* = 9.6, 6.7, 2.4 Hz, 2H), 6.64 (tdd, *J* = 7.3, 2.6, 1.3 Hz, 1H), 6.58 – 6.45 (m, 2H), 4.73 – 4.66 (m, 1H), 4.37 – 4.30 (m, 1H), 2.05 – 1.72 (m, 4H); ^{13}C NMR (125 MHz, Chloroform-*d*) δ 147.2, 144.3, 143.8, 143.7, 129.1, 128.6, 128.6, 128.5, 127.7, 127.7, 127.0, 126.4, 126.4, 125.9, 125.8, 117.3, 117.3, 113.4, 113.4, 74.3, 74.3, 58.3, 58.2, 35.7, 35.6, 34.9,

34.7, 29.7; IR (thin film) 3423, 3028, 2929, 2855, 1601, 1504, 1490, 1317, 750, 702; HRMS (ESI), calculated for $C_{22}H_{23}NO$: (M+H$^+$) 318.1858 observed 318.1870.

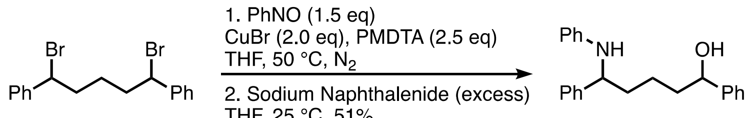

S3 **17**

1,5-diphenyl-5-(phenylamino)pentan-1-ol (**17**): To a 100 mL oven dried round bottom flask is added CuBr (28 mg, 0.2 mmol, 2 eq) and PMDTA (52 µL, 0.25 mmol, 2.5 eq) in THF (anhydrous, 30 mL). This suspension is sonicated for one minutes and sparged with nitrogen for thirty minutes. A second solution of nitrosobenzene (16 mg, 0.15 mmol, 1.5 eq) and dibromide **S3** (38 mg, 0.1 mmol, 1 eq) is prepared in THF (anhydrous, 10 mL) in a 10 mL conical flask. This solution is sparged with nitrogen for ten minutes. The copper suspension is stirred at 50 °C under nitrogen while the nitroso solution is added dropwise to the copper suspension via syringe pump over ten hours. Upon completion of addition, the reaction is allowed to continue stirring under nitrogen for an additional six hours.

The solution is concentrated *in vacuo* and reconstituted in DCM (10 mL). The DCM solution is washed with EDTA (0.5 M, pH 8, 20 mL) and extracted with DCM (3 x 15 mL). The organic layers are combined and sequentially dried with brine (20 mL) and sodium sulfate.

A 500 mM solution of Sodium Naphthalenide was freshly prepared in THF while the substrate to be reduced was dissolved in 1 mL of THF in a 1-dram vial and briefly degassed with N_2. The napthalenide was titrated slowly until the substrate solution remained dark green. After three minutes, the reaction was quenched with isopropanol (~5 mL), washed with saturated sodium bicarbonate (10 mL) and extracted with ethyl acetate (2 x 10 mL). Organic layers were combined and dried sequentially with brine (10 mL) and sodium sulfate. The crude product was dry loaded onto Celite and purified via flash chromatography with an increasing gradient of DCM in hexane (0 —> 50%) to afford **17** as a 1.5:1 mix of diastereomers (17 mg, 51%). ^{1}H NMR (400 MHz, Chloroform-*d*) δ 7.41 – 7.15 (m, 10H), 7.07 (t, *J* = 7.7 Hz, 2H), 6.62 (t, *J* = 7.3 Hz, 1H), 6.49 (d, *J* = 8.0 Hz, 2H), 4.65 (dd, *J* = 7.7, 5.6 Hz, 1H), 4.29 (t, *J* = 6.8 Hz, 1H), 1.93 – 1.29 (m, 6H); ^{13}C NMR (100 MHz, Chloroform-*d*) δ 147.3, 144.6, 144.1, 144.0, 129.0, 128.5, 128.5, 127.6, 126.9, 126.3, 125.8, 117.1, 113.2, 74.4, 74.4, 58.1, 58.0, 38.7, 38.7, 22.8, 22.6; IR (thin film) 3404, 2913, 2856, 1599, 1501, 1451, 1315, 1027, 748, 696 cm $^{-1}$; HRMS (ESI), calculated for $C_{23}H_{25}NO$: (M+H$^+$) 322.2014 observed 332.2321.

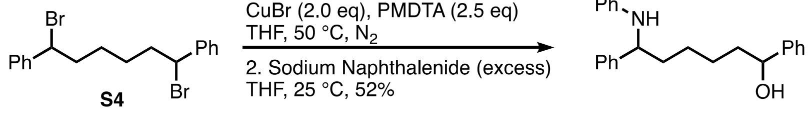

18

1,6-diphenyl-6-(phenylamino)hexan-1-ol (**18**): To a 100 mL oven dried round bottom flask is added CuBr (28 mg, 0.2 mmol, 2 eq), $CuBr_2$ (4 mg, 0.02 mmol, 10 mol% relative to CuBr) and PMDTA (52 µL, 0.25 mmol, 2.5 eq) in THF (anhydrous, 30 mL). This suspension is sonicated for one minutes and sparged with nitrogen for thirty minutes. A second solution of nitrosobenzene (16 mg, 0.15 mmol, 1.5 eq) and dibromide **S4** (40 mg, 0.1 mmol, 1 eq) is

prepared in THF (anhydrous, 10 mL) in a 10 mL conical flask. This solution is sparged with nitrogen for ten minutes. The copper suspension is stirred at 50 °C under nitrogen while the nitroso solution is added dropwise to the copper suspension via syringe pump over ten hours. Upon completion of addition, the reaction is allowed to continue stirring under nitrogen for an additional six hours.

The solution is concentrated *in vacuo* and reconstituted in DCM (10 mL). The DCM solution is washed with EDTA (0.5 M, pH 8, 20 mL) and extracted with DCM (3 x 15 mL). The organic layers are combined and sequentially dried with brine (20 mL) and sodium sulfate.

A 500 mM solution of Sodium Naphthalenide was freshly prepared in THF while the substrate to be reduced was dissolved in 1 mL of THF in a 1-dram vial and briefly degassed with N_2. The napthalenide was titrated slowly until the substrate solution remained dark green. After three minutes, the reaction was quenched with isopropanol (~5 mL), washed with saturated sodium bicarbonate (10 mL) and extracted with ethyl acetate (2 x 10 mL). Organic layers were combined and dried sequentially with brine (10 mL) and sodium sulfate. The crude product was dry loaded onto Celite and purified via flash chromatography with an increasing gradient of DCM in hexane (0 —> 50%) to afford **18** as a 1:1 mix of diasteromers (18 mg, 52%). ^{1}H NMR (400 MHz, Chloroform-*d*) δ 7.42 – 7.27 (m, 8H), 7.25 – 7.18 (m, 1H), 7.07 (t, *J* = 8.5 Hz, 2H), 6.63 (t, *J* = 7.3 Hz, 1H), 6.49 (d, *J* = 7.7 Hz, 2H), 4.64 (dd, *J* = 7.4, 5.8 Hz, 1H), 4.37 – 4.19 (m, 1H), 4.04 (bs, 1H), 1.89 – 1.63 (m, 5H), 1.59 (bs, 1H), 1.53 – 1.23 (m, 4H); ^{13}C NMR (100 MHz, Chloroform-*d*) δ 147.4, 144.7, 144.1, 129.0, 128.6, 128.5, 128.5, 127.6, 126.9, 126.3, 125.8, 125.8, 117.1, 113.2, 74.5, 74.5, 58.1, 38.8, 38.8, 38.8, 26.2, 25.7, 25.6; IR (thin film) 3353, 2980, 2931, 2857, 1655, 1529, 1156, 702 cm $^{-1}$; HRMS (ESI), calculated for $C_{24}H_{27}NO$: (M+Na$^+$) 346.2171 observed 346.2175.

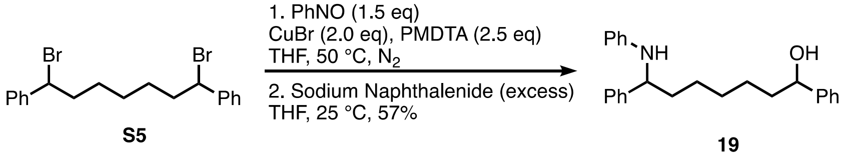

1,7-diphenyl-7-(phenylamino)heptan-1-ol (**19**): To a 100 mL oven dried round bottom flask is added CuBr (28 mg, 0.2 mmol, 2 eq) and PMDTA (52 µL, 0.25 mmol, 2.5 eq) in THF (anhydrous, 7 mL). This suspension is sonicated for one minutes and sparged with nitrogen for seven minutes. A second solution of nitrosobenzene (16 mg, 0.15 mmol, 1.5 eq) and dibromide **S5** (41 mg, 0.1 mmol, 1 eq) is prepared in THF (anhydrous, 3 mL) in a 10 mL conical flask. This solution is sparged with nitrogen for three minutes. The copper suspension is stirred at 50 °C under nitrogen while the nitroso solution is added dropwise to the copper suspension via syringe pump over ten hours. Upon completion of addition, the reaction is allowed to continue stirring under nitrogen for an additional eight hours.

The solution is concentrated *in vacuo* and reconstituted in DCM (10 mL). The DCM solution is washed with EDTA (0.5 M, pH 8, 20 mL) and extracted with DCM (3 x 15 mL). The organic layers are combined and sequentially dried with brine (20 mL) and sodium sulfate.

A 500 mM solution of Sodium Naphthalenide was freshly prepared in THF while the substrate to be reduced was dissolved in 1 mL of THF in a 1-dram vial and briefly degassed with N_2. The napthalenide was titrated slowly until the substrate solution remained dark green. After three minutes, the reaction was quenched with isopropanol (~5 mL), washed with saturated sodium bicarbonate (10 mL) and extracted with ethyl acetate (2 x 10 mL). Organic layers were combined and dried sequentially with brine (10 mL) and sodium sulfate. The crude product was dry loaded onto Celite and purified via flash chromatography with an increasing gradient of DCM in hexane (0 —> 50%) to afford **19** as a mix of diastereomers (20 mg, 57%). ^{1}H NMR (500 MHz, Chloroform-*d*) δ 7.40 – 7.25 (m, 10H), 7.22 (ddd, *J* = 8.5, 5.7, 2.3 Hz, 1H), 7.12 – 7.04 (m, 2H), 6.67 – 6.60 (m, 1H), 6.56 – 6.46 (m, 2H), 4.65 (dd, *J* = 7.5, 5.8 Hz, 1H), 4.28 (t, *J* = 6.8 Hz, 1H), 4.06 (bs, 1H), 1.90 – 1.63 (m, 3H), 1.57 (bs, 1H), 1.36 (tdd, *J* = 30.4, 16.6, 7.4 Hz, 7H); ^{13}C NMR (125 MHz, Chloroform-*d*) δ 144.8, 144.8, 129.1, 128.5, 128.4, 127.5, 126.9, 126.4, 125.9, 117.2, 113.3, 74.6, 74.6, 58.3, 38.9, 38.8, 29.3, 26.2, 26.2, 25.6, 25.6; IR (thin film) 3399, 3028, 2929, 2845, 1600, 1502, 1451, 1317, 1027, 749, 699; HRMS (ESI), calculated for $C_{25}H_{29}NO$: (M+H$^+$) 360.2372 observed 360.2290.

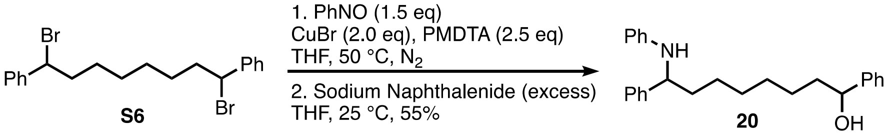

1,8-diphenyl-8-(phenylamino)octan-1-ol (**20**): To a 100 mL oven dried round bottom flask is added CuBr (28 mg, 0.2 mmol, 2 eq), $CuBr_2$ (4 mg, 0.02 mmol, 10 mol% relative to CuBr) and PMDTA (52 µL, 0.25 mmol, 2.5 eq) in THF (anhydrous, 30 mL). This suspension is sonicated for one minutes and sparged with nitrogen for thirty minutes. A second solution of nitrosobenzene (16 mg, 0.15 mmol, 1.5 eq) and dibromide **S6** (42 mg, 0.1 mmol, 1 eq) is prepared in THF (anhydrous, 10 mL) in a 10 mL conical flask. This solution is sparged with nitrogen for ten minutes. The copper suspension is stirred at 50 °C under nitrogen while the nitroso solution is added dropwise to the copper suspension via syringe pump over ten hours. Upon completion of addition, the reaction is allowed to continue stirring under nitrogen for an additional eight hours.

The solution is concentrated *in vacuo* and reconstituted in DCM (10 mL). The DCM solution is washed with EDTA (0.5 M, pH 8, 20 mL) and extracted with DCM (3 x 15 mL). The organic layers are combined and sequentially dried with brine (20 mL) and sodium sulfate.

A 500 mM solution of Sodium Naphthalenide was freshly prepared in THF while the substrate to be reduced was dissolved in 1 mL of THF in a 1-dram vial and briefly degassed with N_2. The napthalenide was titrated slowly until the substrate solution remained dark green. After three minutes, the reaction was quenched with isopropanol (~5 mL), washed with saturated sodium bicarbonate (10 mL) and extracted with ethyl acetate (2 x 10 mL). Organic layers were combined and dried sequentially with brine (10 mL) and sodium sulfate. The crude product was dry loaded onto Celite and purified via flash chromatography with an increasing gradient of DCM in hexane (0 —> 50%) to afford **20** (20 mg, 55%). ^{1}H NMR (500 MHz, Chloroform-*d*) δ 7.38 – 7.27 (m, 10H), 7.24 – 7.19 (m, 1H), 7.11 – 7.02 (m, 2H), 6.62 (t, *J* = 7.4 Hz, 1H), 6.54 – 6.45 (m, 2H), 4.68 – 4.61 (m, 1H), 4.27 (t, *J* = 6.8 Hz, 1H), 4.03

(bs, 1H), 1.84 – 1.63 (m, 5H), 1.45 – 1.24 (m, 7H); ^{13}C NMR (100 MHz, Chloroform-*d*) δ 147.4, 144.8, 144.2, 129.1, 128.5, 128.4, 127.5, 126.8, 126.3, 125.9, 117.1, 113.2, 74.6, 58.2, 39.0, 38.9, 29.3, 29.3, 26.2, 25.7; IR (thin film) 3402, 3026, 2927, 2853, 1600, 1503, 1452, 1316, 1179, 1027, 749, 700; HRMS (ESI), calculated for $C_{26}H_{31}NO$: (M+H$^+$) 374.2484 observed 374.2978.

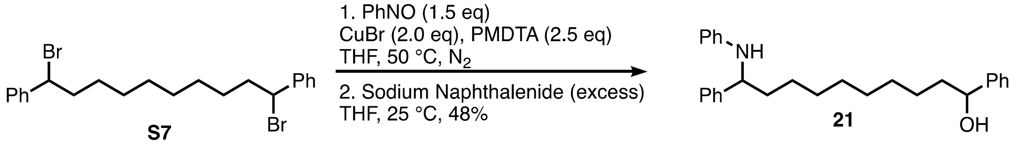

1,10-diphenyl-10-(phenylamino)decan-1-ol (**21**): To a 100 mL oven dried round bottom flask is added CuBr (28 mg, 0.2 mmol, 2 eq), $CuBr_2$ (4 mg, 0.02 mmol, 10 mol% relative to CuBr) and PMDTA (52 µL, 0.25 mmol, 2.5 eq) in THF (anhydrous, 30 mL). This suspension is sonicated for one minutes and sparged with nitrogen for thirty minutes. A second solution of nitrosobenzene (16 mg, 0.15 mmol, 1.5 eq) and dibromide **S7** (45 mg, 0.1 mmol, 1 eq) is prepared in THF (anhydrous, 10 mL) in a 10 mL conical flask. This solution is sparged with nitrogen for ten minutes. The copper suspension is stirred at 50 °C under nitrogen while the nitroso solution is added dropwise to the copper suspension via syringe pump over ten hours. Upon completion of addition, the reaction is allowed to continue stirring under nitrogen for an additional eight hours.

The solution is concentrated *in vacuo* and reconstituted in DCM (10 mL). The DCM solution is washed with EDTA (0.5 M, pH 8, 20 mL) and extracted with DCM (3 x 15 mL). The organic layers are combined and sequentially dried with brine (20 mL) and sodium sulfate.

A 500 mM solution of Sodium Naphthalenide was freshly prepared in THF while the substrate to be reduced was dissolved in 1 mL of THF in a 1-dram vial and briefly degassed with N_2. The napthalenide was titrated slowly until the substrate solution remained dark green. After three minutes, the reaction was quenched with isopropanol (~5 mL), washed with saturated sodium bicarbonate (10 mL) and extracted with ethyl acetate (2 x 10 mL). Organic layers were combined and dried sequentially with brine (10 mL) and sodium sulfate. The crude product was dry loaded onto Celite and purified via flash chromatography with an increasing gradient of DCM in hexane (0 —> 50%) to afford **21** (19 mg, 48%). ^{1}H NMR (400 MHz, Chloroform-*d*) δ 7.42 – 7.17 (m, 10H), 7.13 – 7.04 (m, 2H), 6.63 (t, *J* = 7.3 Hz, 1H), 6.51 (d, *J* = 7.9 Hz, 2H), 4.66 (dd, *J* = 7.5, 5.7 Hz, 1H), 4.28 (t, *J* = 6.8 Hz, 1H), 4.07 (s, 1H), 1.88 – 1.63 (m, 4H), 1.57 (bs, 1H), 1.48 – 1.17 (m, 12H); ^{13}C NMR (125 MHz, Chloroform-*d*) δ 147.5, 144.9, 144.3, 129.1, 128.5, 128.4, 127.5, 126.8, 126.4, 125.9, 125.9, 117.1, 113.2, 74.7, 58.2, 39.1, 39.0, 29.5, 29.5, 29.4, 29.4, 26.3, 25.8; IR (thin film) 3421, 3028, 2927, 2851, 1600, 1501, 1451, 1027, 748, 697 cm^{-1}; HRMS (ESI), calculated for $C_{28}H_{35}NO$: (M+H$^+$) 402.2797 observed 402.2787.

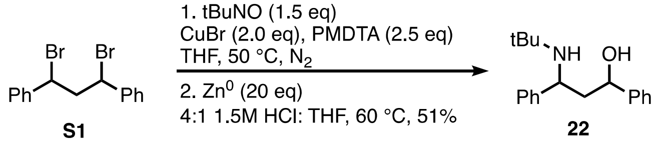

3-(*tert*-butylamino)-1,3-diphenylpropan-1-ol (**22**): To a 25 mL oven dried round bottom flask is added CuBr (28 mg, 0.2 mmol, 2 eq) and PMDTA (52 µL, 0.25 mmol, 2.5 eq) in THF (anhydrous, 7 mL). This suspension is sonicated for one minutes and sparged with nitrogen for seven minutes. A second solution of 2-methyl-2-nitrosopropane dimer (13 mg, 0.075 mmol, 1.5 eq) and dibromide **S1** (35 mg, 0.1 mmol, 1 eq, 1.1:1 mix of diastereomers) is prepared in THF (anhydrous, 3 mL) in a 10 mL conical flask. This solution is sparged with nitrogen for three minutes. The copper suspension is stirred at 50 °C under nitrogen while the nitroso solution is added dropwise to the copper suspension via syringe pump over ten hours. Upon completion of addition, the reaction is allowed to continue stirring under nitrogen for an additional fourteen hours.

The solution is concentrated *in vacuo* and reconstituted in DCM (10 mL). The DCM solution is washed with EDTA (0.5 M, pH 10, 20 mL) and extracted with DCM (3 x 15 mL). The organic layers are combined and sequentially dried with brine (20 mL) and sodium sulfate.

A 1-dram vial containing the resulting crude N–O alkylated adduct and a stir bar was charged with activated Zn^0 (130 mg, 2 mmol, 20 equiv) and 4:1 solution of 1.5 M HCl and THF (1.5 mL) and heated to 60 °C. The solution was stirred until consumption of the starting material was observed by TLC, upon which the crude reaction mixture was quenched with an aqueous solution of saturated $NaHCO_3$ (20 mL). The resulting precipitate was removed through vacuum filtration and the filtrate was extracted with EtOAc (3 x 15 mL). The combined organic layers were washed with water (1 x 15 mL) and once with brine (1 x 15 mL) and then dried over sodium sulfate. The crude product was dry loaded onto Celite and purified via flash chromatography on a Yamazen brand amino-column with an increasing gradient of EtOAc in hexane (0 —> 50%) to afford **22** as a 1.7:1 mix of diasteromers (14 mg, 51%). *Major Diastereomer **22-a***: ^{1}H NMR (500 MHz, Chloroform-*d*) δ 7.37 (d, *J* = 7.3 Hz, 2H), 7.34 – 7.26 (m, 4H), 7.25 – 7.17 (m, 4H), 5.00 (dd, *J* = 9.6, 2.9 Hz, 1H), 4.16 (dd, *J* = 10.4, 4.0 Hz, 1H), 1.89 – 1.73 (m, 2H), 1.07 (s, 9H); ^{13}C NMR (125 MHz, Chloroform-*d*) δ 146.7, 145.1, 128.7, 128.2, 127.0, 126.9, 125.8, 125.5, 75.2, 58.9, 52.0, 48.1, 30.3; IR (thin film) 3287, 3026, 2963, 2805, 1453, 1206, 1063, 751, 699 cm $^{-1}$; HRMS (ESI), calculated for $C_{19}H_{25}NO$: $(M+H^+)$ 284.2014 observed 284.2027. *Minor Diastereomer **22-b***: ^{1}H NMR (500 MHz, Chloroform-*d*) δ 7.42 – 7.39 (m, 2H), 7.38 – 7.28 (m, 4H), 7.25 – 7.18 (m, 2H), 7.17 – 7.13 (m, 2H), 5.02 (t, *J* = 4.7 Hz, 1H), 3.93 (dd, *J* = 8.9, 3.2 Hz, 1H), 2.14 (ddd, *J* = 14.5, 8.9, 3.9 Hz, 1H), 2.00 (ddd, *J* = 14.5, 5.5, 3.2 Hz, 1H), 0.98 (s, 9H); ^{13}C NMR (125 MHz, Chloroform-*d*) δ 146.2, 145.2, 128.7, 128.1, 126.9, 126.6, 126.0, 125.7, 72.1, 54.9, 51.9, 45.3, 30.2; IR (thin film) 3260, 3057, 3026, 2961, 2924, 2854, 1601, 1453, 1365, 1210, 1064, 1029, 759, 700, 668 cm $^{-1}$; HRMS (ESI), calculated for $C_{19}H_{25}NO$: $(M+H^+)$ 284.2014 observed 284.2024.

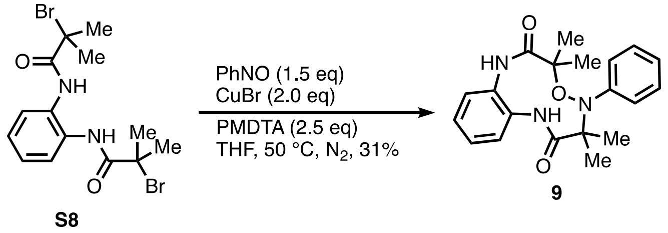

3,3,6,6-tetramethyl-5-phenyl-1,5,6,8-tetrahydro-7*H*-benzo[*f*][1,2,5,8]oxatriazecine-2,7(3*H*)-dione (**9**): To a 100 mL oven dried round bottom flask is added CuBr (28 mg, 0.2 mmol, 2 eq) and PMDTA (52 µL, 0.25 mmol, 2.5 eq) in THF (anhydrous, 30 mL). This suspension is sonicated for one minutes and sparged with nitrogen for thirty minutes. A second solution of nitrosobenzene (16 mg, 0.15 mmol, 1.5 eq) and dibromide **S8** (40 mg, 0.1 mmol, 1 eq) is prepared in THF (anhydrous, 10 mL) in a 10 mL conical flask. This solution is sparged with nitrogen for ten minutes. The copper suspension is stirred at 50 °C under nitrogen while the nitroso solution is added dropwise to the copper suspension via syringe pump over ten hours. Upon completion of addition, the reaction is allowed to continue stirring under nitrogen for an additional six hours.

The solution is concentrated *in vacuo* and reconstituted in DCM (10 mL). The DCM solution is washed with EDTA (0.5 M, pH 8, 20 mL) and extracted with DCM (3 x 15 mL). The organic layers are combined and sequentially dried with brine (20 mL) and sodium sulfate. The crude product was dry loaded onto Celite and purified via flash chromatography on a Yamazen brand amino-column with an increasing gradient of DCM in hexane (0 —> 35%) to afford **9** as a white crystalline solid (11 mg, 31%). ^{1}H NMR (500 MHz, Chloroform-*d*) δ 7.72 (s, 1H), 7.58 (s, 1H), 7.44 – 7.32 (m, 5H), 7.28 (s, 1H), 7.21 (tt, *J* = 7.3, 1.3 Hz, 1H), 7.09 (s, 1H), 1.45 (s, 3H), 1.36 (s, 3H), 1.18 (s, 3H), 1.05 (s, 3H); ^{13}C NMR (125 MHz, Chloroform-*d*) δ 177.5, 176.5, 148.6, 136.1, 135.5, 129.2, 129.2, 128.9, 128.8, 128.3, 127.7, 126.3, 126.1, 124.7, 82.4, 68.5, 26.6, 25.1, 22.4, 14.9; IR (thin film) 3333, 3274, 2922, 2853, 1669, 1501, 1164, 750, 706 cm^{-1}; HRMS (ESI), calculated for $C_{20}H_{23}N_3O_3$: (M+Na$^+$) 376.1637 observed 376.1637.

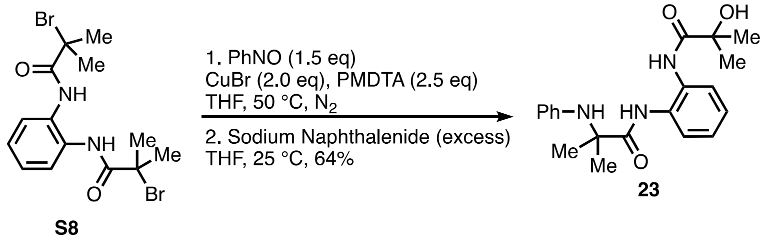

2-hydroxy-2-methyl-*N*-(2-(2-methyl-2-(phenylamino)propanamido)phenyl)propanamide (**23**): To a 100 mL oven dried round bottom flask is added CuBr (28 mg, 0.2 mmol, 2 eq) and PMDTA (52 µL, 0.25 mmol, 2.5 eq) in THF (anhydrous, 30 mL). This suspension is sonicated for one minutes and sparged with nitrogen for thirty minutes. A second solution of nitrosobenzene (16 mg, 0.15 mmol, 1.5 eq) and dibromide **S8** (40 mg, 0.1 mmol, 1 eq) is prepared in THF (anhydrous, 10 mL) in a 10 mL conical flask. This solution is sparged with nitrogen for ten minutes. The copper suspension is stirred at 50 °C under nitrogen while the nitroso solution is added dropwise to the copper

suspension via syringe pump over ten hours. Upon completion of addition, the reaction is allowed to continue stirring under nitrogen for an additional six hours.

The solution is concentrated *in vacuo* and reconstituted in DCM (10 mL). The DCM solution is washed with EDTA (0.5 M, pH 8, 20 mL) and extracted with DCM (3 x 15 mL). The organic layers are combined and sequentially dried with brine (20 mL) and sodium sulfate.

A 500 mM solution of Sodium Naphthalenide was freshly prepared in THF while the substrate to be reduced was dissolved in 1 mL of THF in a 1-dram vial and briefly degassed with N_2. The napthalenide was titrated slowly until the substrate solution remained dark green. After three minutes, the reaction was quenched with isopropanol (~5 mL), washed with saturated sodium bicarbonate (10 mL) and extracted with ethyl acetate (2 x 10 mL). Organic layers were combined and dried sequentially with brine (10 mL) and sodium sulfate. The crude product was dry loaded onto Celite and purified via flash chromatography on a Yamazen brand amino-column with an increasing gradient of DCM in hexane (0 —> 50%) to afford **23** (27 mg, 64%). ^{1}H NMR (500 MHz, Chloroform-*d*) δ 9.17 (s, 1H), 8.83 (s, 1H), 7.59 (dd, *J* = 7.7, 1.8 Hz, 1H), 7.37 (dd, *J* = 7.7, 1.9 Hz, 1H), 7.24 – 7.13 (m, 4H), 6.84 (tt, *J* = 7.3, 1.0 Hz, 1H), 6.64 (d, *J* = 8.5 Hz, 2H), 3.94 (bs, 1H), 2.30 (bs, 1H), 1.59 (s, 6H), 1.39 (s, 6H); ^{13}C NMR (125 MHz, Chloroform-*d*) δ 175.6, 175.4, 144.7, 130.3, 130.1, 129.5 126.4, 126.4, 126.2, 125.1, 125.1, 124.8, 119.8, 116.5, 73.7, 58.9, 27.7, 26.0; IR (thin film) 3337, 3054, 2976, 2930, 2866, 1661, 1597, 1499, 1445, 1364, 1298, 1258, 1184, 1148, 749, 734, 695 cm $^{-1}$; HRMS (ESI), calculated for $C_{20}H_{25}N_3O_3$: $(M+H^+)$ 356.1974 observed 356.1968.

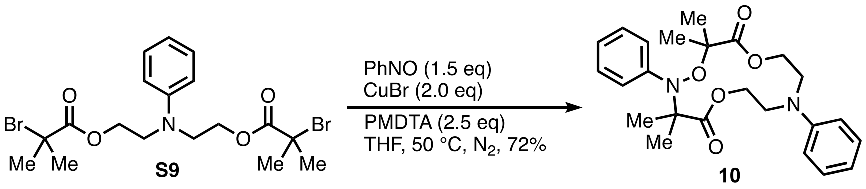

3,3,13,13-tetramethyl-2,8-diphenyl-1,5,11-trioxa-2,8-diazacyclotridecane-4,12-dione (10): To a 100 mL oven dried round bottom flask is added CuBr (28 mg, 0.2 mmol, 2 eq) and PMDTA (52 µL, 0.25 mmol, 2.5 eq) in THF (anhydrous, 30 mL). This suspension is sonicated for one minutes and sparged with nitrogen for thirty minutes. A second solution of nitrosobenzene (16 mg, 0.15 mmol, 1.5 eq) and dibromide **S9** (48 mg, 0.1 mmol, 1 eq) is prepared in THF (anhydrous, 10 mL) in a 10 mL conical flask. This solution is sparged with nitrogen for ten minutes. The copper suspension is stirred at 50 °C under nitrogen while the nitroso solution is added dropwise to the copper suspension via syringe pump over ten hours. Upon completion of addition, the reaction is allowed to continue stirring under nitrogen for an additional six hours.

The solution is concentrated *in vacuo* and reconstituted in DCM (10 mL). The DCM solution is washed with EDTA (0.5 M, pH 8, 20 mL) and extracted with DCM (3 x 15 mL). The organic layers are combined and sequentially dried with brine (20 mL) and sodium sulfate. The crude product was dry loaded onto Celite and purified via flash chromatography with an increasing gradient of DCM in hexane (0 —> 35%) to afford **10** (30 mg, 72%). ^{1}H NMR (500

MHz, Chloroform-*d*) δ 7.32 – 7.23 (m, 4H), 7.22 – 7.15 (m, 2H), 7.15 – 7.07 (m, 3H), 6.85 – 6.76 (m, 3H), 4.53 – 4.38 (m, 2H), 4.27 (tt, *J* = 11.6, 4.0 Hz, 2H), 3.74 (tq, *J* = 3.9, 2.3, 1.5 Hz, 4H), 1.31 (s, 3H), 1.28 (s, 3H), 1.14 (s, 3H), 1.10 (s, 3H); ^{13}C NMR (125 MHz, Chloroform-*d*) δ 174.3, 173.5, 148.9, 148.2, 129.2, 127.7, 125.8, 125.3, 117.7, 114.0, 83.1, 68.0, 63.8, 62.9, 51.5, 50.0, 25.8, 25.0, 23.1, 19.3; IR (thin film) 3060, 2982, 2927, 2852, 1732, 1598, 1504, 1286, 1173, 1154, 749, 700 cm^{-1}; HRMS (ESI), calculated for $C_{24}H_{30}N_2O_5$: (M+Na$^+$) 449.2052 observed 449.2067.

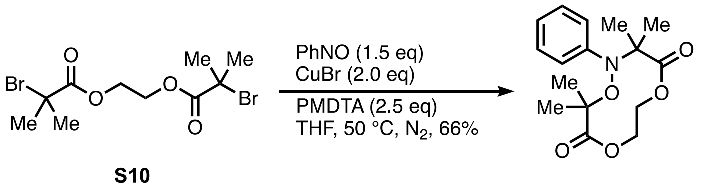

S10 **11**

3,3,10,10-tetramethyl-2-phenyl-1,5,8,2-trioxazecane-4,9-dione (**11**): To a 100 mL oven dried round bottom flask is added CuBr (28 mg, 0.2 mmol, 2 eq) and PMDTA (52 µL, 0.25 mmol, 2.5 eq) in THF (anhydrous, 30 mL). This suspension is sonicated for one minutes and sparged with nitrogen for thirty minutes. A second solution of nitrosobenzene (16 mg, 0.15 mmol, 1.5 eq) and dibromide **S10** (36 mg, 0.1 mmol, 1 eq) is prepared in THF (anhydrous, 10 mL) in a 10 mL conical flask. This solution is sparged with nitrogen for ten minutes. The copper suspension is stirred at 50 °C under nitrogen while the nitroso solution is added dropwise to the copper suspension via syringe pump over ten hours. Upon completion of addition, the reaction is allowed to continue stirring under nitrogen for an additional six hours.

The solution is concentrated *in vacuo* and reconstituted in DCM (10 mL). The DCM solution is washed with EDTA (0.5 M, pH 8, 20 mL) and extracted with DCM (3 x 15 mL). The organic layers are combined and sequentially dried with brine (20 mL) and sodium sulfate. The crude product was dry loaded onto Celite and purified via flash chromatography with an increasing gradient of EtOAc in hexane (0 —> 50%) to afford **11** (20 mg, 66%). ^{1}H NMR (500 MHz, Chloroform-*d*) δ 7.49 – 7.00 (m, 5H), 5.25 (tdd, *J* = 10.6, 5.3, 0.7 Hz, 1H), 5.16 (tdd, *J* = 10.9, 5.3, 0.7 Hz, 1H), 3.94 – 3.84 (m, 2H), 1.37 (s, 3H), 1.32 (s, 3H), 1.12 (s, 3H), 0.97 (s, 3H); ^{13}C NMR (125 MHz, Chloroform-*d*) δ 173.7, 172.3, 148.4, 127.7, 126.1, 125.8, 82.5, 67.8, 59.1, 58.8, 24.9, 24.3, 23.9, 16.2; IR (thin film) 2998, 2967, 2924, 2852, 1748, 1277, 1162, 1123, 705 cm^{-1}; HRMS (ESI), calculated for $C_{16}H_{21}NO_5$: (M+Na$^+$) 330.1317 observed 330.1331.

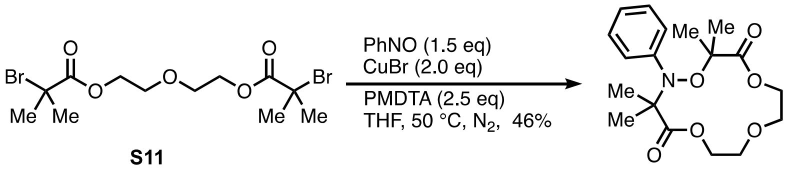

S11 **12**

3,3,13,13-tetramethyl-2-phenyl-1,5,8,11-tetraoxa-2-azacyclotridecane-4,12-dione (**12**): To a 100 mL oven dried round bottom flask is added CuBr (28 mg, 0.2 mmol, 2 eq) and PMDTA (52 µL, 0.25 mmol, 2.5 eq) in THF (anhydrous, 30 mL). This suspension is sonicated for one minutes and sparged with nitrogen for thirty minutes. A second solution of

nitrosobenzene (16 mg, 0.15 mmol, 1.5 eq) and dibromide **S11** (40 mg, 0.1 mmol, 1 eq) is prepared in THF (anhydrous, 10 mL) in a 10 mL conical flask. This solution is sparged with nitrogen for ten minutes. The copper suspension is stirred at 50 °C under nitrogen while the nitroso solution is added dropwise to the copper suspension via syringe pump over ten hours. Upon completion of addition, the reaction is allowed to continue stirring under nitrogen for an additional six hours.

The solution is concentrated *in vacuo* and reconstituted in DCM (10 mL). The DCM solution is washed with EDTA (0.5 M, pH 8, 20 mL) and extracted with DCM (3 x 15 mL). The organic layers are combined and sequentially dried with brine (20 mL) and sodium sulfate. The crude product was dry loaded onto Celite and purified via flash chromatography with an increasing gradient of EtOAc in hexane (0 —> 50%) to afford **12** (16 mg, 46 %). ^{1}H NMR (500 MHz, Chloroform-*d*) δ 7.36 – 7.23 (m, 4H), 7.15 (ddd, *J* = 8.5, 6.8, 1.8 Hz, 1H), 4.56 (ddd, *J* = 11.7, 9.1, 2.2 Hz, 1H), 4.41 (ddd, *J* = 12.0, 4.8, 2.4 Hz, 1H), 4.22 (ddd, *J* = 11.9, 8.1, 2.4 Hz, 1H), 4.12 (ddd, *J* = 12.2, 4.0, 2.4 Hz, 1H), 3.96 – 3.87 (m, 2H), 3.71 (ddd, *J* = 12.2, 4.0, 2.3 Hz, 1H), 3.65 (ddd, *J* = 11.4, 4.8, 2.4 Hz, 1H), 1.49 (s, 3H), 1.35 (s, 3H), 1.22 (s, 3H), 1.15 (s, 3H); ^{13}C NMR (125 MHz, Chloroform-*d*) δ 174.1, 173.5, 149.4, 127.7, 125.7, 125.5, 83.0, 68.6, 67.8, 67.6, 63.6, 62.5, 62.5, 26.7, 26.2, 22.3, 19.3; IR (thin film) 3060, 2985, 2945, 2865, 1732, 1466, 1381, 1281, 1138, 1091, 1027, 783, 761, 703 cm^{-1} ; HRMS (ESI), calculated for $C_{18}H_{25}NO_6$: (M+Na$^+$) 374.1580 observed 374.1594.

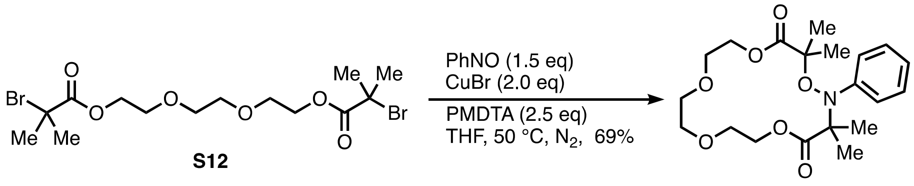

3,3,16,16-tetramethyl-2-phenyl-1,5,8,11,14-pentaoxa-2-azacyclohexadecane-4,15-dione (**13**): To a 100 mL oven dried round bottom flask is added CuBr (28 mg, 0.2 mmol, 2 eq) and PMDTA (52 µL, 0.25 mmol, 2.5 eq) in THF (anhydrous, 30 mL). This suspension is sonicated for one minutes and sparged with nitrogen for thirty minutes. A second solution of nitrosobenzene (16 mg, 0.15 mmol, 1.5 eq) and dibromide **S12** (45 mg, 0.1 mmol, 1 eq) is prepared in THF (anhydrous, 10 mL) in a 10 mL conical flask. This solution is sparged with nitrogen for ten minutes. The copper suspension is stirred at 50 °C under nitrogen while the nitroso solution is added dropwise to the copper suspension via syringe pump over ten hours. Upon completion of addition, the reaction is allowed to continue stirring under nitrogen for an additional six hours.

The solution is concentrated *in vacuo* and reconstituted in DCM (10 mL). The DCM solution is washed with EDTA (0.5 M, pH 8, 20 mL) and extracted with DCM (3 x 15 mL). The organic layers are combined and sequentially dried with brine (20 mL) and sodium sulfate. The crude product was dry loaded onto Celite and purified via flash chromatography with an increasing gradient of EtOAc in hexane (0 —> 50%) to afford **13** (27 mg, 69%). ^{1}H NMR (500 MHz, Chloroform-*d*) δ 7.37 (d, *J* = 7.9 Hz, 2H), 7.23 (t, *J* = 7.7 Hz, 2H), 7.09 (t, *J* = 7.3 Hz, 1H), 4.46 – 4.35 (m, 1H), 4.17 – 4.06 (m, 2H), 3.99 – 3.89 (m, 1H), 3.85 – 3.67 (m, 8H), 1.40 – 1.30

(m, 12H); ^{13}C NMR (125 MHz, Chloroform-*d*) δ 174.0, 173.0, 149.0, 127.6, 125.5, 125.6, 81.8, 81.8, 70.9, 70.9, 69.0, 68.9, 68.0, 64.5, 64.3, 24.3, 24.1, 24.0, 23.6; IR (thin film) 3061, 2982, 2942, 2869, 1732, 1451, 1280, 1172, 1142, 702 cm $^{-1}$; HRMS (ESI), calculated for $C_{20}H_{29}NO_7$: (M+Na$^+$) 418.1842 observed 462.1828.

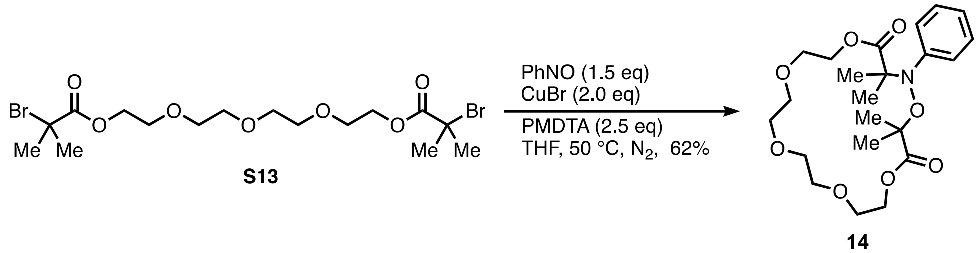

3,3,19,19-tetramethyl-2-phenyl-1,5,8,11,14,17-hexaoxa-2-azacyclononadecane-4,18-dione (**14**): To a 100 mL oven dried round bottom flask is added CuBr (28 mg, 0.2 mmol, 2 eq) and PMDTA (52 µL, 0.25 mmol, 2.5 eq) in THF (anhydrous, 30 mL). This suspension is sonicated for one minutes and sparged with nitrogen for thirty minutes. A second solution of nitrosobenzene (16 mg, 0.15 mmol, 1.5 eq) and dibromide **S13** (49 mg, 0.1 mmol, 1 eq) is prepared in THF (anhydrous, 10 mL) in a 10 mL conical flask. This solution is sparged with nitrogen for ten minutes. The copper suspension is stirred at 50 °C under nitrogen while the nitroso solution is added dropwise to the copper suspension via syringe pump over ten hours. Upon completion of addition, the reaction is allowed to continue stirring under nitrogen for an additional six hours.

The solution is concentrated *in vacuo* and reconstituted in DCM (10 mL). The DCM solution is washed with EDTA (0.5 M, pH 8, 20 mL) and extracted with DCM (3 x 15 mL). The organic layers are combined and sequentially dried with brine (20 mL) and sodium sulfate. The crude product was dry loaded onto Celite and purified via flash chromatography with an increasing gradient of EtOAc in hexane (0 —> 50%) to afford **14** as a mix of rotamers (27 mg, 62%). ^{1}H NMR (500 MHz, Chloroform-*d*) δ 7.39 – 7.31 (m, 2H), 7.23 (dd, *J* = 8.3, 7.1 Hz, 2H), 7.09 (td, *J* = 7.2, 1.3 Hz, 1H), 4.31 (dt, *J* = 9.2, 4.5 Hz, 1H), 4.20 (dd, *J* = 10.6, 5.9 Hz, 1H), 3.80 (t, *J* = 4.4 Hz, 4H), 3.78 – 3.74 (m, 2H), 3.72 (q, *J* = 4.2 Hz, 4H), 3.67 (dt, *J* = 5.6, 2.3 Hz, 2H), 3.61 – 3.44 (m, 2H), 1.47 (s, 3H), 1.36 (s, 3H), 1.33 (s, 6H); ^{13}C NMR (125 MHz, Chloroform-*d*) δ 173.7, 172.7, 148.4, 127.6, 125.7, 125.7, 80.9, 80.9, 77.2, 71.2, 71.1, 71.1, 70.9, 69.1, 68.7, 68.1, 64.6, 64.0, 24.5, 23.4, 23.3, 23.2; IR (thin film) 2983, 2943, 2872, 1733, 1467, 1281, 1172, 1137, 953, 703 cm $^{-1}$; HRMS (ESI), calculated for $C_{22}H_{33}NO_8$ (M+Na$^+$): 462.2104 observed 462.2096.

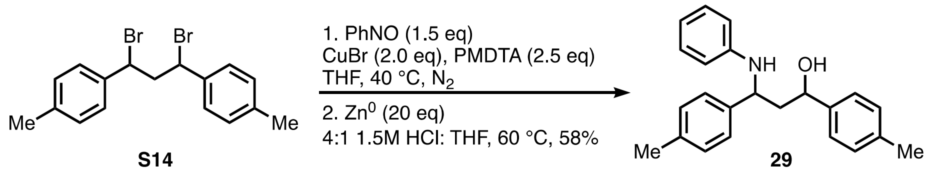

3-(phenylamino)-1,3-di-*p*-tolylpropan-1-ol (**29**): To a 25 mL oven dried round bottom flask is added CuBr (28 mg, 0.2 mmol, 2 eq) and PMDTA (52 µL, 0.25 mmol, 2.5 eq) in THF (anhydrous, 7 mL). This suspension is sonicated for one minutes and sparged with nitrogen

for seven minutes. A second solution of nitrosobenzene (16 mg, 0.15 mmol, 1.5 eq) and dibromide **S14** (38 mg, 0.1 mmol, 1 eq) is prepared in THF (anhydrous, 3 mL) in a 10 mL conical flask. This solution is sparged with nitrogen for three minutes. The copper suspension is stirred at 40 °C under nitrogen while the nitroso solution is added dropwise to the copper suspension via syringe pump over ten hours. Upon completion of addition, the reaction is allowed to continue stirring under nitrogen for an additional six hours.

The solution is concentrated *in vacuo* and reconstituted in DCM (10 mL). The DCM solution is washed with EDTA (0.5 M, pH 8, 20 mL) and extracted with DCM (3 x 15 mL). The organic layers are combined and sequentially dried with brine (20 mL) and sodium sulfate.

A 1-dram vial containing the resulting crude N–O alkylated adduct and a stir bar was charged with activated Zn^0 (130 mg, 2 mmol, 20 equiv) and 4:1 solution of 1.5 M HCl and THF (1.5 mL) and heated to 60 °C. The solution was stirred until consumption of the starting material was observed by TLC, upon which the crude reaction mixture was quenched with an aqueous solution of saturated $NaHCO_3$ (20 mL). The resulting precipitate was removed through vacuum filtration and the filtrate was extracted with EtOAc (3x 15 mL). The combined organic layers were washed with water (1x 15 mL) and once with brine (1x 15 mL) and then dried over sodium sulfate. The crude product was dry loaded onto Celite and purified via flash chromatography with an increasing gradient of DCM in hexane (0 —> 50%) to afford **29** as a 3:1 mix of diastereomers (19 mg, 58%). ^1H NMR (400 MHz, $CDCl_3$) δ 7.30 – 7.19 (m, 5H), 7.19 – 7.03 (m, 6H), 6.73 – 6.62 (m, 1H), 6.62 – 6.49 (m, 2H), 4.82 (dt, *J* = 6.9, 3.4 Hz, 1H), 4.57 (dt, *J* = 9.0, 5.2 Hz, 1H), 2.40 – 2.20 (m, 7H), 2.03 (ddd, *J* = 14.5, 5.1, 3.4 Hz, 1H); ^{13}C NMR (100 MHz, $CDCl_3$) δ 147.2, 141.5, 140.6, 137.4, 136.6, 129.4, 129.3, 129.3, 129.2, 129.1, 129.1, 126.2, 126.1, 125.7, 125.7, 117.9, 117.4, 114.3, 113.6, 73.6, 71.7, 57.7, 55.1, 47.4, 46.6, 21.1, 21.1; IR (thin film) 3366, 3011, 2912, 1596, 1499, 812, 689 cm^{-1}; HRMS (CI), calculated for $C_{23}H_{25}NO$ (M^+): 331.1936 observed 331.1935.

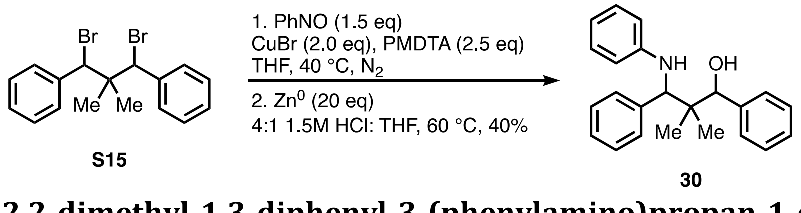

2,2-dimethyl-1,3-diphenyl-3-(phenylamino)propan-1-ol (**30**): To a 25 mL oven dried round bottom flask is added CuBr (28 mg, 0.2 mmol, 2 eq) and PMDTA (52 µL, 0.25 mmol, 2.5 eq) in THF (anhydrous, 7 mL). This suspension is sonicated for one minutes and sparged with nitrogen for seven minutes. A second solution of nitrosobenzene (16 mg, 0.15 mmol, 1.5 eq) and dibromide **S15** (38 mg, 0.1 mmol, 1 eq) is prepared in THF (anhydrous, 3 mL) in a 10 mL conical flask. This solution is sparged with nitrogen for three minutes. The copper suspension is stirred at 40 °C under nitrogen while the nitroso solution is added dropwise to the copper suspension via syringe pump over ten hours. Upon completion of addition, the reaction is allowed to continue stirring under nitrogen for an additional six hours.

The solution is concentrated *in vacuo* and reconstituted in DCM (10 mL). The DCM solution is washed with EDTA (0.5 M, pH 8, 20 mL) and extracted with DCM (3 x 15 mL). The organic layers are combined and sequentially dried with brine (20 mL) and sodium sulfate.

A 1-dram vial containing the resulting crude N–O alkylated adduct and a stir bar was charged with activated Zn^0 (130 mg, 2 mmol, 20 equiv) and 4:1 solution of 1.5 M HCl and THF (1.5 mL) and heated to 60 °C. The solution was stirred until consumption of the starting material was observed by TLC, upon which the crude reaction mixture was quenched with an aqueous solution of saturated $NaHCO_3$ (20 mL). The resulting precipitate was removed through vacuum filtration and the filtrate was extracted with EtOAc (3x 15 mL). The combined organic layers were washed with water (1x 15 mL) and once with brine (1x 15 mL) and then dried over sodium sulfate. The crude product was dry loaded onto Celite and purified via flash chromatography with an increasing gradient of DCM in hexane (0 —> 50%) to afford **30** as a 2:1 mix of diastereomers (13 mg, 40%). ^1H NMR (500 MHz, $CDCl_3$) δ 7.39 – 7.27 (m, 9H), 7.24 – 7.18 (m, 1H), 7.12 – 7.04 (m, 2H), 6.71 – 6.65 (m, 0.67 H, minor), 6.64 – 6.58 (m, 1.63H, major + minor), 6.57 – 6.53 (m, 0.7H, minor), 4.80 (s, 0.67H, major), 4.70 (s, 0.33H, minor), 4.46 (s, 0.67H, major), 4.36 (s, 0.33H, minor), 1.13 (s, 2H major), 0.96 (s, 1H minor), 0.92 (s, 1H minor), 0.62 (s, 2H major); ^{13}C NMR (125 MHz, $CDCl_3$) δ 146.8, 141.6, 140.1, 129.0, 128.9, 128.6, 128.0, 127.9, 127.8, 127.8, 127.7, 127.6, 127.5, 127.0, 126.9, 118.3, 116.9, 114.8, 113.5, 81.0, 80.4, 65.7, 65.4, 42.2, 29.7, 22.8, 22.2, 22.0, 15.8; IR (thin film) 3553, 3379, 3058, 3027, 2972, 2928, 2875, 1600, 1501, 1452, 1317, 1042, 750, 735, 703 cm^{-1}; HRMS (EI) calculated for $C_{23}H_{25}NO$ (M^+): 331.1936 observed 331.1934.

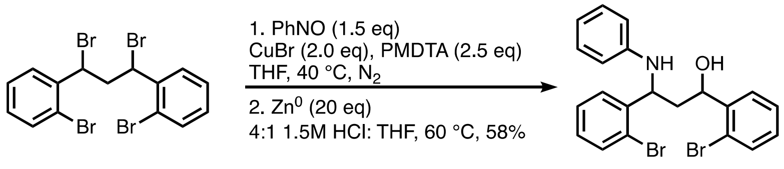

S16

31

1,3-bis(2-bromophenyl)-3-(phenylamino)propan-1-ol (**31**): To a 25 mL oven dried round bottom flask is added CuBr (28 mg, 0.2 mmol, 2 eq) and PMDTA (52 µL, 0.25 mmol, 2.5 eq) in THF (anhydrous, 7 mL). This suspension is sonicated for one minutes and sparged with nitrogen for seven minutes. A second solution of nitrosobenzene (16 mg, 0.15 mmol, 1.5 eq) and dibromide **S16** (51 mg, 0.1 mmol, 1 eq) is prepared in THF (anhydrous, 3 mL) in a 10 mL conical flask. This solution is sparged with nitrogen for three minutes. The copper suspension is stirred at 40 °C under nitrogen while the nitroso solution is added dropwise to the copper suspension via syringe pump over ten hours. Upon completion of addition, the reaction is allowed to continue stirring under nitrogen for an additional six hours.

The solution is concentrated *in vacuo* and reconstituted in DCM (10 mL). The DCM solution is washed with EDTA (0.5 M, pH 8, 20 mL) and extracted with DCM (3 x 15 mL). The organic layers are combined and sequentially dried with brine (20 mL) and sodium sulfate.

A 1-dram vial containing the resulting crude N–O alkylated adduct and a stir bar was charged with activated Zn^0 (130 mg, 2 mmol, 20 equiv) and 4:1 solution of 1.5 M HCl and THF (1.5 mL) and heated to 60 °C. The solution was stirred until consumption of the starting material

was observed by TLC, upon which the crude reaction mixture was quenched with an aqueous solution of saturated $NaHCO_3$ (20 mL). The resulting precipitate was removed through vacuum filtration and the filtrate was extracted with EtOAc (3x 15 mL). The combined organic layers were washed with water (1x 15 mL) and once with brine (1x 15 mL) and then dried over sodium sulfate. The crude product was dry loaded onto Celite and purified via flash chromatography with an increasing gradient of DCM in hexane (0 —> 50%) to afford **31** as a 2:1 mix of diastereomers (27 mg, 58%). ^{1}H NMR (400 MHz, $CDCl_3$) δ 7.80 – 6.85 (m, 10H), 6.87 – 6.47 (m, 3H), 5.44 – 5.20 (m, 1H), 5.07 (ddd, *J* = 23.6, 8.9, 3.8 Hz, 0.5H), 4.74 – 4.69 (m, 0.5H), 2.37 – 1.78 (m, 2H). ^{13}C NMR (100 MHz, $CDCl_3$) δ 147.1, 143.4, 133.1, 132.6, 129.1, 128.9, 128.7, 12, 127.8, 127.2, 127.0, 126.2, 118.2, 114.5, 114.2, 113.3, 73.2, 72.9, 58.8, 57.7, 45.8, 44.2; IR (thin film) 3377, 2913, 1597, 1500, 1020, 748 cm^{-1}; HRMS (CI), calculated for $C_{21}H_{19}NOBr_2$ (M^+): 458.9833 observed 458.9819.

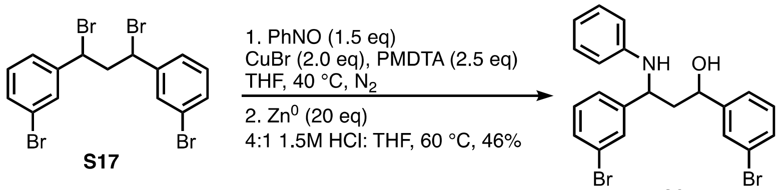

S17 → 32

1,3-bis(3-bromophenyl)-3-(phenylamino)propan-1-ol (**32**): To a 25 mL oven dried round bottom flask is added CuBr (28 mg, 0.2 mmol, 2 eq) and PMDTA (52 µL, 0.25 mmol, 2.5 eq) in THF (anhydrous, 7 mL). This suspension is sonicated for one minutes and sparged with nitrogen for seven minutes. A second solution of nitrosobenzene (16 mg, 0.15 mmol, 1.5 eq) and dibromide **S17** (51 mg, 0.1 mmol, 1 eq) is prepared in THF (anhydrous, 3 mL) in a 10 mL conical flask. This solution is sparged with nitrogen for three minutes. The copper suspension is stirred at 40 °C under nitrogen while the nitroso solution is added dropwise to the copper suspension via syringe pump over ten hours. Upon completion of addition, the reaction is allowed to continue stirring under nitrogen for an additional six hours.

The solution is concentrated *in vacuo* and reconstituted in DCM (10 mL). The DCM solution is washed with EDTA (0.5 M, pH 8, 20 mL) and extracted with DCM (3 x 15 mL). The organic layers are combined and sequentially dried with brine (20 mL) and sodium sulfate.

A 1-dram vial containing the resulting crude N–O alkylated adduct and a stir bar was charged with activated Zn^0 (130 mg, 2 mmol, 20 equiv) and 4:1 solution of 1.5 M HCl and THF (1.5 mL) and heated to 60 °C. The solution was stirred until consumption of the starting material was observed by TLC, upon which the crude reaction mixture was quenched with an aqueous solution of saturated $NaHCO_3$ (20 mL). The resulting precipitate was removed through vacuum filtration and the filtrate was extracted with EtOAc (3x 15 mL). The combined organic layers were washed with water (1x 15 mL) and once with brine (1x 15 mL) and then dried over sodium sulfate. The crude product was dry loaded onto Celite and purified via flash chromatography with an increasing gradient of DCM in hexane (0 —> 50%) to afford **32** as a 10:1 mix of diastereomers (21 mg, 46%). ^{1}H NMR (400 MHz, $CDCl_3$) δ 7.54 – 7.46 (m, 2H), 7.38 (m, 2H), 7.32 – 7.08 (m, 6H), 6.77 – 6.69 (m, 1H), 6.61 – 6.50 (m, 2H), 4.82 (dd, *J* = 9.6, 3.1 Hz, 1H), 4.62 (dd, *J* = 8.7, 3.4 Hz, 0.12H minor), 4.55 (dd, *J* = 9.1, 5.0 Hz, 0.88H major),

2.20 (dt, *J* = 14.5, 9.3 Hz, 1H), 1.98 (ddd, *J* = 14.5, 5.0, 3.1 Hz, 1H); ^{13}C NMR (100 MHz, $CDCl_3$) δ 146.6, 146.5, 146.0, 130.9, 130.4, 130.4, 130.2, 129.3, 129.2, 128.7, 124.8, 124.3, 122.9, 122.7, 118.4, 114.3, 73.1, 57.8, 47.2; IR (thin film) 3376, 3044, 2914, 1596, 1500, 1066, 780, cm^{-1}; HRMS (EI), calculated for $C_{21}H_{19}NOBr_2$ (M^+): 458.9833 observed 458.9840

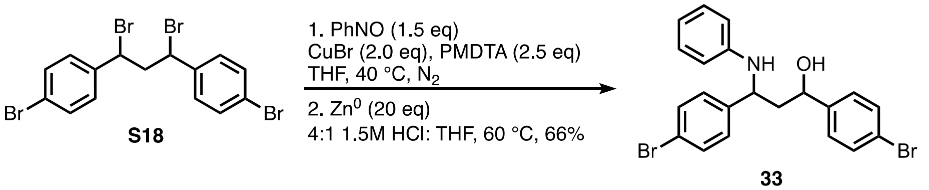

1,3-bis(4-bromophenyl)-3-(phenylamino)propan-1-ol (**33**): To a 25 mL oven dried round bottom flask is added CuBr (28 mg, 0.2 mmol, 2 eq) and PMDTA (52 µL, 0.25 mmol, 2.5 eq) in THF (anhydrous, 7 mL). This suspension is sonicated for one minutes and sparged with nitrogen for seven minutes. A second solution of nitrosobenzene (16 mg, 0.15 mmol, 1.5 eq) and dibromide **S18** (51 mg, 0.1 mmol, 1 eq) is prepared in THF (anhydrous, 3 mL) in a 10 mL conical flask. This solution is sparged with nitrogen for three minutes. The copper suspension is stirred at 40 °C under nitrogen while the nitroso solution is added dropwise to the copper suspension via syringe pump over ten hours. Upon completion of addition, the reaction is allowed to continue stirring under nitrogen for an additional six hours.

The solution is concentrated *in vacuo* and reconstituted in DCM (10 mL). The DCM solution is washed with EDTA (0.5 M, pH 8, 20 mL) and extracted with DCM (3 x 15 mL). The organic layers are combined and sequentially dried with brine (20 mL) and sodium sulfate.

A 1-dram vial containing the resulting crude N–O alkylated adduct and a stir bar was charged with activated Zn^0 (130 mg, 2 mmol, 20 equiv) and 4:1 solution of 1.5 M HCl and THF (1.5 mL) and heated to 60 °C. The solution was stirred until consumption of the starting material was observed by TLC, upon which the crude reaction mixture was quenched with an aqueous solution of saturated $NaHCO_3$ (20 mL). The resulting precipitate was removed through vacuum filtration and the filtrate was extracted with EtOAc (3x 15 mL). The combined organic layers were washed with water (1x 15 mL) and once with brine (1x 15 mL) and then dried over sodium sulfate. The crude product was dry loaded onto Celite and purified via flash chromatography with an increasing gradient of DCM in hexane (0 —> 50%) to afford **33** as a 5:1 mix of diastereomers (30 mg, 66%). ^1H NMR (400 MHz, $CDCl_3$) δ 7.52 – 7.39 (m, 4H), 7.25 – 7.16 (m, 4H), 7.15 – 7.06 (m, 2H), 6.76 – 6.66 (m, 1H), 6.52 (ddt, *J* = 16.1, 6.9, 1.1 Hz, 2H), 4.80 (dd, *J* = 9.4, 3.4 Hz, 1H), 4.53 (dd, *J* = 9.1, 5.1 Hz, 1H), 2.19 (dt, *J* = 14.5, 9.2 Hz, 1H), 1.95 (ddd, *J* = 14.4, 5.1, 3.4 Hz, 1H); ^{13}C NMR (100 MHz, $CDCl_3$) δ 146.6, 143.2, 143.0, 142.5, 131.9, 131.8, 131.7, 129.2, 129.2, 128.0, 128.0, 127.4, 121.6, 121.5, 120.9, 118.4, 117.9, 114.3, 113.7, 73.0, 71.2, 57.4, 54.8, 47.2, 46.4; IR (thin film) 3374, 3042, 2913, 1596, 1480, 1067, 1006, 822, 748 cm^{-1}; HRMS (ESI), calculated for $C_{21}H_{19}Br_2NO$ ($M+H^+$): 459.9911 observed 459.9905.

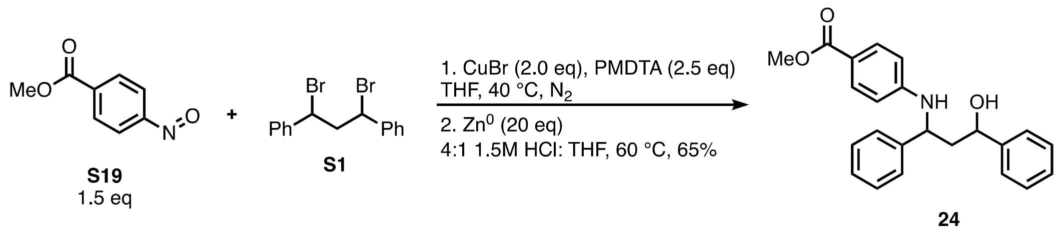

24

Methyl 4-((3-hydroxy-1,3-diphenylpropyl)amino)benzoate (**24**): To a 25 mL oven dried round bottom flask is added CuBr (28 mg, 0.2 mmol, 2 eq) and PMDTA (52 µL, 0.25 mmol, 2.5 eq) in THF (anhydrous, 7 mL). This suspension is sonicated for one minutes and sparged with nitrogen for seven minutes. A second solution of nitrosoarene **S19** (25 mg, 0.15 mmol, 1.5 eq) and dibromide **S1** (35 mg, 0.1 mmol, 1 eq, 1.1:1 mix of diastereomers) is prepared in THF (anhydrous, 3 mL) in a 10 mL conical flask. This solution is sparged with nitrogen for three minutes. The copper suspension is stirred at 40 °C under nitrogen while the nitroso solution is added dropwise to the copper suspension via syringe pump over ten hours. Upon completion of addition, the reaction is allowed to continue stirring under nitrogen for an additional six hours.

The solution is concentrated *in vacuo* and reconstituted in DCM (10 mL). The DCM solution is washed with EDTA (0.5 M, pH 8, 20 mL) and extracted with DCM (3 x 15 mL). The organic layers are combined and sequentially dried with brine (20 mL) and sodium sulfate.

A 1-dram vial containing the resulting crude N–O alkylated adduct and a stir bar was charged with activated Zn^0 (130 mg, 2 mmol, 20 equiv) and 4:1 solution of 1.5 M HCl and THF (1.5 mL) and heated to 60 °C. The solution was stirred until consumption of the starting material was observed by TLC, upon which the crude reaction mixture was quenched with an aqueous solution of saturated $NaHCO_3$ (20 mL). The resulting precipitate was removed through vacuum filtration and the filtrate was extracted with EtOAc (3x 15 mL). The combined organic layers were washed with water (1x 15 mL) and once with brine (1x 15 mL) and then dried over sodium sulfate. The crude product was dry loaded onto Celite and purified via flash chromatography with an increasing gradient of DCM in hexane (0 —> 50%) to afford **24** as a 4.6:1 mix of diastereomers (23.5 mg, 65%). ^{1}H NMR (600 MHz, $CDCl_3$) δ 7.75 (d, *J* = 8.5 Hz, 2H), 7.40 – 7.25 (m, 9H), 6.46 (d, *J* = 8.5 Hz, 2H), 4.79 (dd, *J* = 9.2, 3.3 Hz, 1H), 4.61 (dd, *J* = 8.7, 5.6 Hz, 1H), 3.79 (s, 3H), 2.30 (dt, *J* = 14.5, 9.0 Hz, 1H), 2.06 (ddd, *J* = 14.5, 5.6, 3.4 Hz, 1H); ^{13}C NMR (150 MHz, $CDCl_3$) δ 167.3, 151.1, 144.2, 142.9, 131.3, 128.8, 128.7, 128.0, 127.4, 126.1, 125.6, 118.5, 112.6, 73.4, 57.2, 51.5, 47.2; IR (thin film) 3368, 3029, 2948, 1685, 1601, 1523, 1434, 1274, 1172, 836, 771 cm^{-1}; HRMS (EI) calculated for $C_{23}H_{23}NO_3$ (M^+): 361.1670 observed 361.1674.

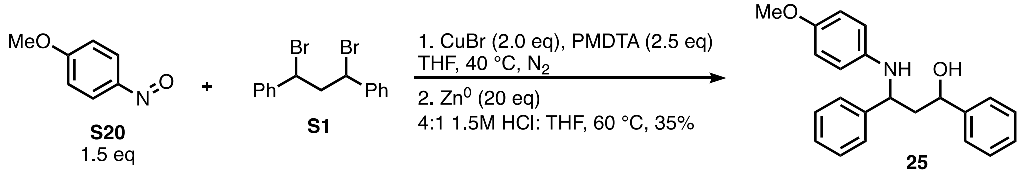

25

3-((4-methoxyphenyl)amino)-1,3-diphenylpropan-1-ol (**25**): To a 25 mL oven dried round bottom flask is added CuBr (28 mg, 0.2 mmol, 2 eq) and PMDTA (52 µL, 0.25 mmol,

2.5 eq) in THF (anhydrous, 7 mL). This suspension is sonicated for one minutes and sparged with nitrogen for seven minutes. A second solution of nitrosoarene **S20** (20 mg, 0.15 mmol, 1.5 eq) and dibromide **S1** (35 mg, 0.1 mmol, 1 eq, 1.1:1 mix of diastereomers) is prepared in THF (anhydrous, 3 mL) in a 10 mL conical flask. This solution is sparged with nitrogen for three minutes. The copper suspension is stirred at 40 °C under nitrogen while the nitroso solution is added dropwise to the copper suspension via syringe pump over ten hours. Upon completion of addition, the reaction is allowed to continue stirring under nitrogen for an additional six hours.

The solution is concentrated *in vacuo* and reconstituted in DCM (10 mL). The DCM solution is washed with EDTA (0.5 M, pH 8, 20 mL) and extracted with DCM (3 x 15 mL). The organic layers are combined and sequentially dried with brine (20 mL) and sodium sulfate.

A 1-dram vial containing the resulting crude N–O alkylated adduct and a stir bar was charged with activated Zn^0 (130 mg, 2 mmol, 20 equiv) and 4:1 solution of 1.5 M HCl and THF (1.5 mL) and heated to 60 °C. The solution was stirred until consumption of the starting material was observed by TLC, upon which the crude reaction mixture was quenched with an aqueous solution of saturated $NaHCO_3$ (20 mL). The resulting precipitate was removed through vacuum filtration and the filtrate was extracted with EtOAc (3x 15 mL). The combined organic layers were washed with water (1x 15 mL) and once with brine (1x 15 mL) and then dried over sodium sulfate. The crude product was dry loaded onto Celite and purified via flash chromatography with an increasing gradient of EtOAc in hexane (0 —> 50%) to afford **25** as a 2.4:1 mix of diastereomers (17 mg, 51%). ^1H NMR (600 MHz, $CDCl_3$) δ 7.37 – 7.25 (m, 9H), 7.19 (tt, *J* = 6.1, 2.2 Hz, 1H), 6.70 – 6.65 (m, 2H), 6.58 – 6.54 (m, 1.31H major), 6.50 – 6.46 (m, 0.65H minor), 4.91 (td, *J* = 9.3, 8.8, 3.4 Hz, 1H), 4.53 (dd, *J* = 9.5, 4.5 Hz, 0.64H major), 4.50 (dd, *J* = 8.8, 3.8 Hz, 0.32H minor), 3.68 (d, *J* = 5.4 Hz, 3H), 2.30 – 2.14 (m, 1H), 2.04 (ddd, *J* = 14.5, 4.5, 3.1 Hz, 1H); ^{13}C NMR (125 MHz, $CDCl_3$) δ 152.8, 144.5, 143.6, 140.9, 128.7, 128.6, 128.6, 128.5, 127.6, 127.6, 127.2, 127.0, 126.3, 126.2, 125.7, 125.7, 116.3, 115.4, 114.7, 114.7, 74.2, 72.0, 59.7, 56.4, 55.7, 55.7, 47.3, 46.5; IR (thin film) 3377, 3027, 2919, 2850, 1501, 1452, 1233, 1028, 818, 751 cm^{-1}; HRMS (CI) calculated for $C_{22}H_{23}NO_2$ (M^+): 333.1729; observed 333.1731.[8]

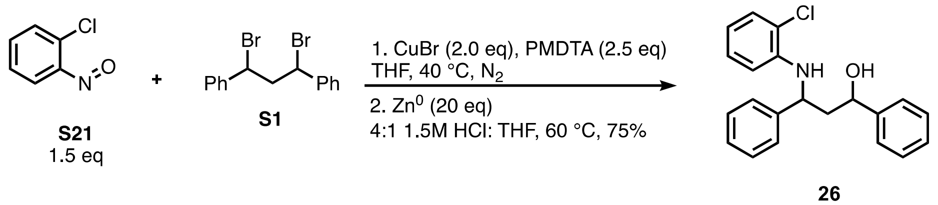

26

3-((2-chlorophenyl)amino)-1,3-diphenylpropan-1-ol (**26**): To a 25 mL oven dried round bottom flask is added CuBr (28 mg, 0.2 mmol, 2 eq) and PMDTA (52 µL, 0.25 mmol, 2.5 eq) in THF (anhydrous, 7 mL). This suspension is sonicated for one minutes and sparged with nitrogen for seven minutes. A second solution of nitrosoarene **S21** (21 mg, 0.15 mmol, 1.5 eq) and dibromide **S1** (35 mg, 0.1 mmol, 1 eq, 1.1:1 mix of diastereomers) is prepared in THF (anhydrous, 3 mL) in a 10 mL conical flask. This solution is sparged with nitrogen for three minutes. The copper suspension is stirred at 40 °C under nitrogen while the nitroso solution is added dropwise to the copper suspension via syringe pump over ten hours. Upon

completion of addition, the reaction is allowed to continue stirring under nitrogen for an additional six hours.

The solution is concentrated *in vacuo* and reconstituted in DCM (10 mL). The DCM solution is washed with EDTA (0.5 M, pH 8, 20 mL) and extracted with DCM (3 x 15 mL). The organic layers are combined and sequentially dried with brine (20 mL) and sodium sulfate.

A 1-dram vial containing the resulting crude N–O alkylated adduct and a stir bar was charged with activated Zn^0 (130 mg, 2 mmol, 20 equiv) and 4:1 solution of 1.5 M HCl and THF (1.5 mL) and heated to 60 °C. The solution was stirred until consumption of the starting material was observed by TLC, upon which the crude reaction mixture was quenched with an aqueous solution of saturated $NaHCO_3$ (20 mL). The resulting precipitate was removed through vacuum filtration and the filtrate was extracted with EtOAc (3x 15 mL). The combined organic layers were washed with water (1x 15 mL) and once with brine (1x 15 mL) and then dried over sodium sulfate. The crude product was dry loaded onto Celite and purified via flash chromatography with an increasing gradient of DCM in hexane (0 —> 50%) to afford **26** as a 2.3:1 mix of diastereomers (25 mg, 75%). *Major diastereomer*: ^1H NMR (500 MHz, $CDCl_3$) δ 7.34 – 7.20 (m, 9H), 7.16 (dt, *J* = 6.4, 2.1 Hz, 1H), 6.91 (t, *J* = 8.0 Hz, 1H), 6.55 (dd, *J* = 7.9, 2.0 Hz, 1H), 6.46 (t, *J* = 2.2 Hz, 1H), 6.32 (dd, *J* = 8.1, 2.4 Hz, 1H), 4.75 (dd, *J* = 9.2, 3.3 Hz, 1H), 4.47 (dd, *J* = 8.8, 5.3 Hz, 1H), 2.20 (dt, *J* = 14.6, 9.0 Hz, 1H), 1.98 (ddd, *J* = 14.5, 5.4, 3.3 Hz, 1H); *Minor diastereomer*: ^1H NMR (500 MHz, $CDCl_3$) δ 7.31 – 7.20 (m, 9H), 7.19 – 7.14 (m, 1H), 6.91 (t, *J* = 8.0 Hz, 1H), 6.55 (ddd, *J* = 7.9, 2.0, 0.9 Hz, 1H), 6.46 (t, *J* = 2.1 Hz, 1H), 6.32 (ddd, *J* = 8.2, 2.3, 0.9 Hz, 1H), 4.76 (dd, *J* = 9.3, 3.3 Hz, 1H), 4.47 (dd, *J* = 8.8, 5.3 Hz, 1H), 2.20 (dt, *J* = 14.5, 9.1 Hz, 1H), 1.98 (ddd, *J* = 14.5, 5.3, 3.4 Hz, 1H); ^{13}C NMR (125 MHz, $CDCl_3$) δ 144.3, 143.2, 130.0, 128.8, 128.7, 127.9, 127.3, 126.2, 125.7, 117.6, 113.8, 112.1, 105.0, 73.6, 57.7, 47.4; IR (thin film) 3382, 3028, 2921, 1595, 1482, 1323, 988, 908, 761 cm^{-1}; HRMS (EI), calculated for $C_{21}H_{20}NOCl$ (M^+): 337.1229; observed 337.1229.

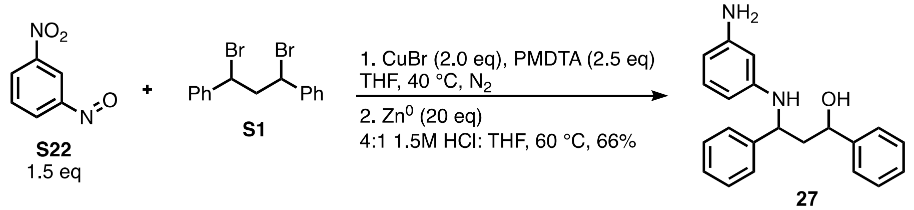

3-((3-aminophenyl)amino)-1,3-diphenylpropan-1-ol (**27**): To a 25 mL oven dried round bottom flask is added CuBr (28 mg, 0.2 mmol, 2 eq) and PMDTA (52 µL, 0.25 mmol, 2.5 eq) in THF (anhydrous, 7 mL). This suspension is sonicated for one minutes and sparged with nitrogen for seven minutes. A second solution of nitrosoarene **S22** (23 mg, 0.15 mmol, 1.5 eq) and dibromide **S1** (35 mg, 0.1 mmol, 1 eq, 1.1:1 mix of diastereomers) is prepared in THF (anhydrous, 3 mL) in a 10 mL conical flask. This solution is sparged with nitrogen for three minutes. The copper suspension is stirred at 40 °C under nitrogen while the nitroso solution is added dropwise to the copper suspension via syringe pump over ten hours. Upon completion of addition, the reaction is allowed to continue stirring under nitrogen for an additional six hours.

The solution is concentrated *in vacuo* and reconstituted in DCM (10 mL). The DCM solution is washed with EDTA (0.5 M, pH 8, 20 mL) and extracted with DCM (3 x 15 mL). The organic layers are combined and sequentially dried with brine (20 mL) and sodium sulfate.

A 1-dram vial containing the resulting crude N–O alkylated adduct and a stir bar was charged with activated Zn^0 (130 mg, 2 mmol, 20 equiv) and 4:1 solution of 1.5 M HCl and THF (1.5 mL) and heated to 60 °C. The solution was stirred until consumption of the starting material was observed by TLC, upon which the crude reaction mixture was quenched with an aqueous solution of saturated $NaHCO_3$ (20 mL). The resulting precipitate was removed through vacuum filtration and the filtrate was extracted with EtOAc (3x 15 mL). The combined organic layers were washed with water (1x 15 mL) and once with brine (1x 15 mL) and then dried over sodium sulfate. The crude product was dry loaded onto Celite and purified via flash chromatography with an increasing gradient of DCM in hexane (0 —> 50%) to afford **27** as a 3.1:1 mix of diastereomers (21 mg, 66%). ^1H NMR (500 MHz, $CDCl_3$) δ 7.29 – 7.19 (m, 9H), 7.15 – 7.11 (m, 1H), 6.84 – 6.77 (m, 1H), 6.00 – 5.91 (m, 2H), 5.84 (t, *J* = 2.2 Hz, 1H 0.84 major), 5.77 (t, *J* = 2.2 Hz, 0.16H minor), 4.77 (dt, *J* = 8.7, 3.4 Hz, 1H), 4.50 (ddd, *J* = 17.3, 8.8, 4.5 Hz, 1H), 2.26 – 2.11 (m, 1H), 2.00 – 1.94 (m, 1H); ^{13}C NMR (125 MHz, $CDCl_3$) δ 148.4, 147.3, 144.5, 143.8, 129.9, 129.9, 128.7, 128.6, 128.6, 128.6, 127.7, 127.7, 127.1, 126.9, 126.3, 126.2, 125.8, 125.7, 105.6, 105.5, 105.1, 104.9, 101.1, 100.3, 73.8, 71.8, 57.9, 55.2, 47.5, 46.8; IR (thin film) 3340, 3019, 2914, 1610, 1489, 1334, 1210, 824, 757, 698 cm^{-1}; HRMS (ESI), calculated for $C_{21}H_{22}N_2O$: $(M+H^+)$ 319.1810; observed 319.1818.

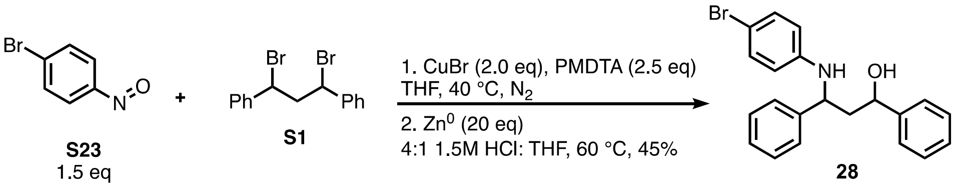

3-((4-bromophenyl)amino)-1,3-diphenylpropan-1-ol (**28**): To a 25 mL oven dried round bottom flask is added CuBr (28 mg, 0.2 mmol, 2 eq) and PMDTA (52 µL, 0.25 mmol, 2.5 eq) in THF (anhydrous, 7 mL). This suspension is sonicated for one minutes and sparged with nitrogen for seven minutes. A second solution of nitrosoarene **S22** (27.5 mg, 0.15 mmol, 1.5 eq) and dibromide **S1** (35 mg, 0.1 mmol, 1 eq, 1.1:1 mix of diastereomers) is prepared in THF (anhydrous, 3 mL) in a 10 mL conical flask. This solution is sparged with nitrogen for three minutes. The copper suspension is stirred at 40 °C under nitrogen while the nitroso solution is added dropwise to the copper suspension via syringe pump over ten hours. Upon completion of addition, the reaction is allowed to continue stirring under nitrogen for an additional six hours.

The solution is concentrated *in vacuo* and reconstituted in DCM (10 mL). The DCM solution is washed with EDTA (0.5 M, pH 8, 20 mL) and extracted with DCM (3 x 15 mL). The organic layers are combined and sequentially dried with brine (20 mL) and sodium sulfate.

A 1-dram vial containing the resulting crude N–O alkylated adduct and a stir bar was charged with activated Zn^0 (130 mg, 2 mmol, 20 equiv) and 4:1 solution of 1.5 M HCl and THF (1.5 mL) and heated to 60 °C. The solution was stirred until consumption of the starting material

was observed by TLC, upon which the crude reaction mixture was quenched with an aqueous solution of saturated $NaHCO_3$ (20 mL). The resulting precipitate was removed through vacuum filtration and the filtrate was extracted with EtOAc (3x 15 mL). The combined organic layers were washed with water (1x 15 mL) and once with brine (1x 15 mL) and then dried over sodium sulfate. The crude product was dry loaded onto Celite and purified via flash chromatography with an increasing gradient of DCM in hexane (0 —> 50%) to afford **28** as a 3.9:1 mix of diastereomers (17 mg, 45%). Major Diastereomer ^1H NMR (500 MHz, $CDCl_3$) δ 7.37 – 7.27 (m, 10H), 7.18 – 7.14 (m, 2H), 6.45 – 6.40 (m, 2H), 4.84 (dd, *J* = 9.2, 3.4 Hz, 1H), 4.53 (dd, *J* = 8.9, 5.3 Hz, 1H), 2.28 (dt, *J* = 14.4, 9.0 Hz, 1H), 2.06 (ddd, *J* = 14.5, 5.3, 3.4 Hz, 1H); ^{13}C NMR (125 MHz, $CDCl_3$) δ 146.3, 144.3, 143.2, 131.8, 128.8, 128.7, 127.9, 127.3, 126.2, 125.6, 115.9, 105.0, 73.7, 57.8, 47.4, 29.7; IR (thin film) 3372, 2924, 1594, 1491, 1454, 1072, 813, 757 cm^{-1}; HRMS (ESI), calculated for $C_{21}H_{20}BrNO$: $(M+H^+)$ 382.0807 and 384.0789; observed 382.0807 and 384.084096.

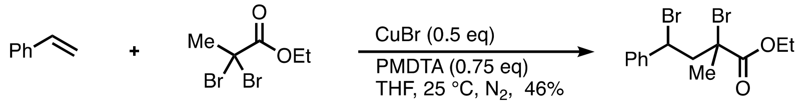

34

Ethyl 2,4-dibromo-2-methyl-4-phenylbutanoate (**34**): To a flame dried 50 mL round bottom flask is added styrene (124 µL, 1 mmol, 1 eq), ethyl 2,2-dibromopropanoate[9] (260 mg, 1 mmol, 1 eq) and CuBr (72 mg, 0.5 mmol, 0.5 eq) in THF (20 mL, anhydrous) and sparged for ten minutes through a runner septum. Next, PMDTA (129 µL, 0.75 mmol, 0.75 eq) is added through the septum to initiate the reaction. After three hours, NMR of an aliquot indicates consumption of the ester. The solution is washed with EDTA (0.5 M, pH 8) and extracted with EtOAc (3 x 15 mL) and dried sequentially with brine and sodium sulfate.
The material was dissolved in a minimal amount (~3 mL) of hexane and loaded directly onto a silica column and purified with an increasing gradient of DCM in hexane (10 —> 40 %) to yield **34** as a yellow oil (150 mg, 42%). ^1H NMR (500 MHz, Chloroform-*d*) δ 7.41 (d, *J* = 7.0 Hz, 2H), 7.33 (d, *J* = 7.2 Hz, 2H), 7.30 – 7.24 (m, 1H), 5.17 (q, *J* = 6.4, 5.9 Hz, 1H), 4.04 (dq, *J* = 10.9, 7.1 Hz, 1H), 3.93 (dq, *J* = 10.8, 7.1 Hz, 1H), 3.26 – 3.19 (m, 2H), 1.84 (s, 3H), 1.23 (t, *J* = 7.1 Hz, 3H); ^{13}C NMR (100 MHz, Chloroform-*d*) δ 170.2, 141.1, 128.7, 128.7, 127.7, 62.34, 59.2, 51.43, 49.9, 28.5, 13.7; IR (thin film) 3027, 2975, 2932, 1732, 1454, 1381, 1254, 1200, 1166, 1109, 1061, 1024, 759, 697 cm^{-1}; HRMS (EI) calculated for $C_{13}H_{16}Br_2O_2$: $(M–Br^–)^+$ 283.0334; observed 283.0323. EI is reported as –Br- due to ionization of bromine which occurs in the mass spec source.

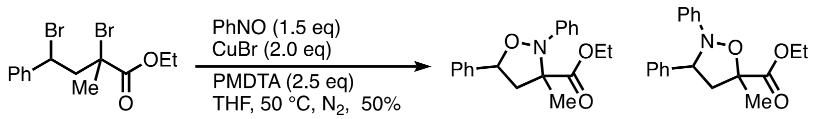

34 **35** **36**

Ethyl 5-methyl-2,3-diphenylisoxazolidine-5-carboxylate and ethyl 3-methyl-2,5-diphenylisoxazolidine-3-carboxylate (**35** and **36**): To a 25 mL oven dried round bottom flask is added CuBr (28 mg, 0.2 mmol, 2 eq) and PMDTA (52 µL, 0.25 mmol, 2.5 eq) in THF (anhydrous, 11 mL). This suspension is sonicated for one minutes and sparged with nitrogen

for ten minutes. A second solution of nitrosobenzene (16 mg, 0.15 mmol, 1.5 eq) and dibromide **34** (0.1 mmol, 1 eq) is prepared in THF (anhydrous, 4 mL) in a 10 mL conical flask. This solution is sparged with nitrogen for three minutes. The copper suspension is stirred at 50 °C under nitrogen while the nitroso solution is added dropwise to the copper suspension via syringe pump over ten hours. Upon completion of addition, the reaction is allowed to continue stirring under nitrogen for an additional six hours.

The solution is concentrated *in vacuo* and reconstituted in EtOAc (10 mL). The EtOAc solution is washed with EDTA (0.5 M, pH 8, 20 mL) and extracted with EtOAc (3 x 15 mL). The organic layers are combined and sequentially dried with brine (20 mL) and magnesium sulfate. The crude product was dry loaded onto Celite and purified via flash chromatography with an increasing gradient of DCM in hexane (0 —> 35%) to afford **35** + **36** as an inseparable mix of regioisomers and diastereomers (15.5 mg, 50%).

Major regioisomer as a mix of diastereomers: ^{1}H NMR (400 MHz, Chloroform-*d*) δ 7.57 (d, *J* = 7.2 Hz, 1H), 7.48 (d, *J* = 7.2 Hz, 1H), 7.40 (t, *J* = 7.4 Hz, 2H), 7.36 – 7.30 (m, 1H), 7.31 – 7.20 (m, 2H), 7.20 – 7.13 (m, 1H), 7.10 (d, *J* = 7.8 Hz, 1H), 7.02 (d, *J* = 7.3 Hz, 1H), 5.40 (dd, *J* = 8.8, 7.1 Hz, 1H), 5.27 (dd, *J* = 8.8, 7.0 Hz, 1H), 4.22 (d, 1H), 3.94 (q, *J* = 7.1 Hz, 1H), 3.21 (dd, *J* = 12.4, 7.1 Hz, 0.5H), 3.01 (dd, *J* = 12.2, 8.9 Hz, 0.5H), 2.62 (dd, *J* = 12.3, 7.0 Hz, 0.5H), 2.33 (dd, *J* = 12.4, 8.8 Hz, 0.5H), 1.70 (s, 1.5H), 1.46 (s, 1.5H), 1.23 (t, *J* = 7.1 Hz, 1.5H), 1.01 (t, *J* = 7.1 Hz, 1.5H); *minor regioisomer, as differentiable from the major:* ^{1}H NMR (400 MHz, Chloroform-*d*) δ 6.87 (t, *J* = 7.4 Hz, 1H), 4.77 (dd, *J* = 9.2, 7.4 Hz, 1H), 4.48 (dd, *J* = 9.1, 6.5 Hz, 2H), 4.02 (q, *J* = 7.2 Hz, 2H), 3.36 (dd, *J* = 12.4, 7.3 Hz, 0.5H), 3.09 – 3.01 (m, 0.5H), 2.68 (dd, *J* = 12.7, 9.2 Hz, 0.5H), 2.40 – 2.29 (m, 0.5H), 1.09 (t, *J* = 7.1 Hz, 3H); ^{13}C NMR (100 MHz, Chloroform-*d*) δ 172.8, 172.4, 147.0, 147.0, 139.3, 138.8, 128.8, 128.8, 128.7, 128.5, 128.5, 128.4, 128.4, 128.3, 128.1, 127.0, 126.9, 126.4, 123.2, 123.0, 117.6, 117.6, 117.0, 78.4, 78.2, 71.5, 70.9, 61.6, 61.2, 51.5, 50.7, 29.7, 23.3, 21.3, 14.0, 13.8; IR (thin film) 2927, 2850, 1726, 1598, 1448, 1259, 1158, 1096, 1027, 756, 699 cm $^{-1}$; HRMS (ESI) calculated for $C_{19}H_{21}NO_3$: (M+Na$^+$) 334.1419; observed 334.1425.

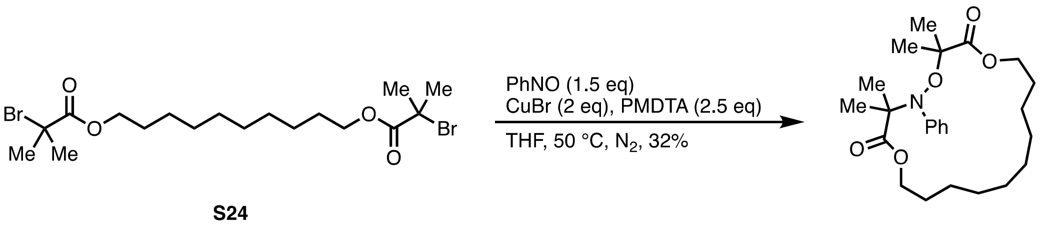

S24

S25

3,3,18,18-tetramethyl-2-phenyl-1,5,16-trioxa-2-azacyclooctadecane-4,17-dione (S25): To a 100 mL oven dried round bottom flask is added CuBr (28 mg, 0.2 mmol, 2 eq) and PMDTA (52 µL, 0.25 mmol, 2.5 eq) in THF (anhydrous, 30 mL). This suspension is sonicated for one minute and sparged with nitrogen for thirty minutes. A second solution of nitrosobenzene (16 mg, 0.15 mmol, 1.5 eq) and dibromide **S24** (47 mg, 0.1 mmol, 1 eq) is prepared in THF (anhydrous, 10 mL) in a 25 mL conical flask. This solution is sparged with nitrogen for ten minutes. The copper suspension is stirred at 50 °C under nitrogen while the nitroso solution is added dropwise to the copper suspension via syringe pump over ten

hours. Upon completion of addition, the reaction is allowed to continue stirring under nitrogen for an additional six hours.

The solution is concentrated *in vacuo* and reconstituted in DCM (10 mL). The DCM solution is washed with EDTA (0.5 M, pH 8, 20 mL) and extracted with DCM (3 x 15 mL). The organic layers are combined and sequentially dried with brine (20 mL) and sodium sulfate. The crude product was dry loaded onto Celite and purified via flash chromatography with an increasing gradient of DCM in hexane to yield **S25** as a clear oil (13.4 mg, 32%). ^{1}H NMR (400 MHz, Chloroform-*d*) δ 7.24 – 7.19 (m, 4H), 7.14 – 7.08 (m, 1H), 4.23 – 4.03 (m, 2H), 3.65 – 3.54 (m, 2H), 1.74 (p, *J* = 6.6 Hz, 2H), 1.54 – 1.20 (m, 26H); ^{13}C NMR (125 MHz, Chloroform-*d*) δ 173.5, 172.8, 148.50 , 127.5, 125.8, 125.4, 81.1, 67.9, 64.7, 64.6, 28.1, 27.5, 27.2, 27.0, 26.9, 26.7, 25.5, 24.7, 24.6, 23.5, 23.4, 23.3; IR (thin film) 2938, 2854, 1734, 1460, 1377, 1362, 1280, 1169, 1143, 767 cm $^{-1}$; HRMS (ESI) calculated for $C_{14}H_{37}NO_6$: $(M+H^+)$ 420.27; observed 420.25.

References

1 G. W. Kabalka, Z. Wu, Y. Ju and M. L. Yao, *J. Org. Chem.*, 2005, **70**, 10285–10291.
2 V. Mojr, E. Svobodová, K. Straková, T. Neveselý, J. Chudoba, H. Dvořáková and R. Cibulka, *Chem. Commun.*, 2015, **51**, 12036–12039.
3 B. Priewisch and K. Rück-Braun, *J. Org. Chem.*, 2005, **70**, 2350–2352.
4 D. J. Fisher, J. B. Shaum, C. L. Mills and J. Read De Alaniz, *Org. Lett.*, 2016, **18**, 5074–5077.
5 R. Huisgen, R. Grashey, H. Hauck and H. Seidl, *Chem. Ber.*, 1968, **101**, 2548–2558.
6 K. B. Simonsen, P. Bayón, R. G. Hazell, K. V Gothelf and K. A. Jørgensen, *J. Am. Chem. Soc.*, 1999, **121**, 3845–3853.
7 A. N. Artemov, E. V Sazonova, E. A. Mavrina and N. Y. Zarovkina, *Russ. Chem. Bull.*, 2012, **61**, 2076–2081.
8 J. Barluenga, F. J. Fananas, J. Villamana and M. Yus, *J. Org. Chem.*, 1982, **47**, 1560–1564.
9 M. Shindo, K. Masumoto and K. Shishido, *Org. Synth.*, 2007, **84**, 11.

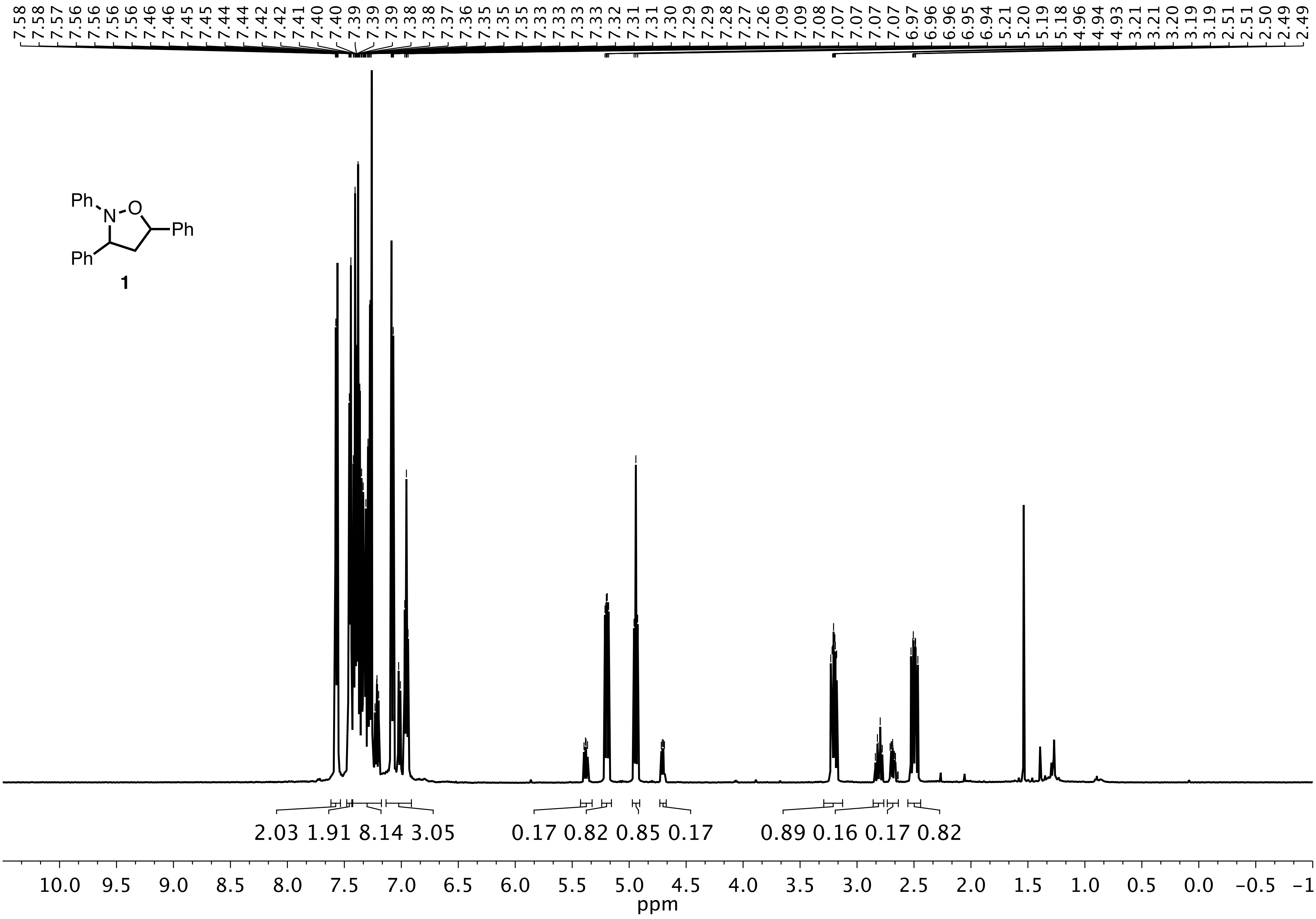

7.58 7.58 7.57 7.56 7.56 7.56 7.56 7.46 7.46 7.45 7.45 7.44 7.44 7.42 7.42 7.41 7.40 7.40 7.39 7.39 7.39 7.38 7.38 7.37 7.36 7.35 7.35 7.35 7.33 7.33 7.33 7.33 7.32 7.31 7.31 7.30 7.29 7.29 7.28 7.27 7.26 7.09 7.09 7.08 7.07 7.07 7.07 7.07 6.97 6.96 6.96 6.95 6.94 5.21 5.20 5.19 5.18 4.96 4.94 4.93 3.21 3.21 3.20 3.19 3.19 2.51 2.51 2.50 2.49 2.49
Ph
N
O
Ph
Ph
1
2.03 1.91 8.14 3.05
0.17 0.82 0.85 0.17
0.89 0.16 0.17 0.82
10.0 9.5 9.0 8.5 8.0 7.5 7.0 6.5 6.0 5.5 5.0 4.5 4.0 3.5 3.0 2.5 2.0 1.5 1.0 0.5 0.0 −0.5 −1
ppm

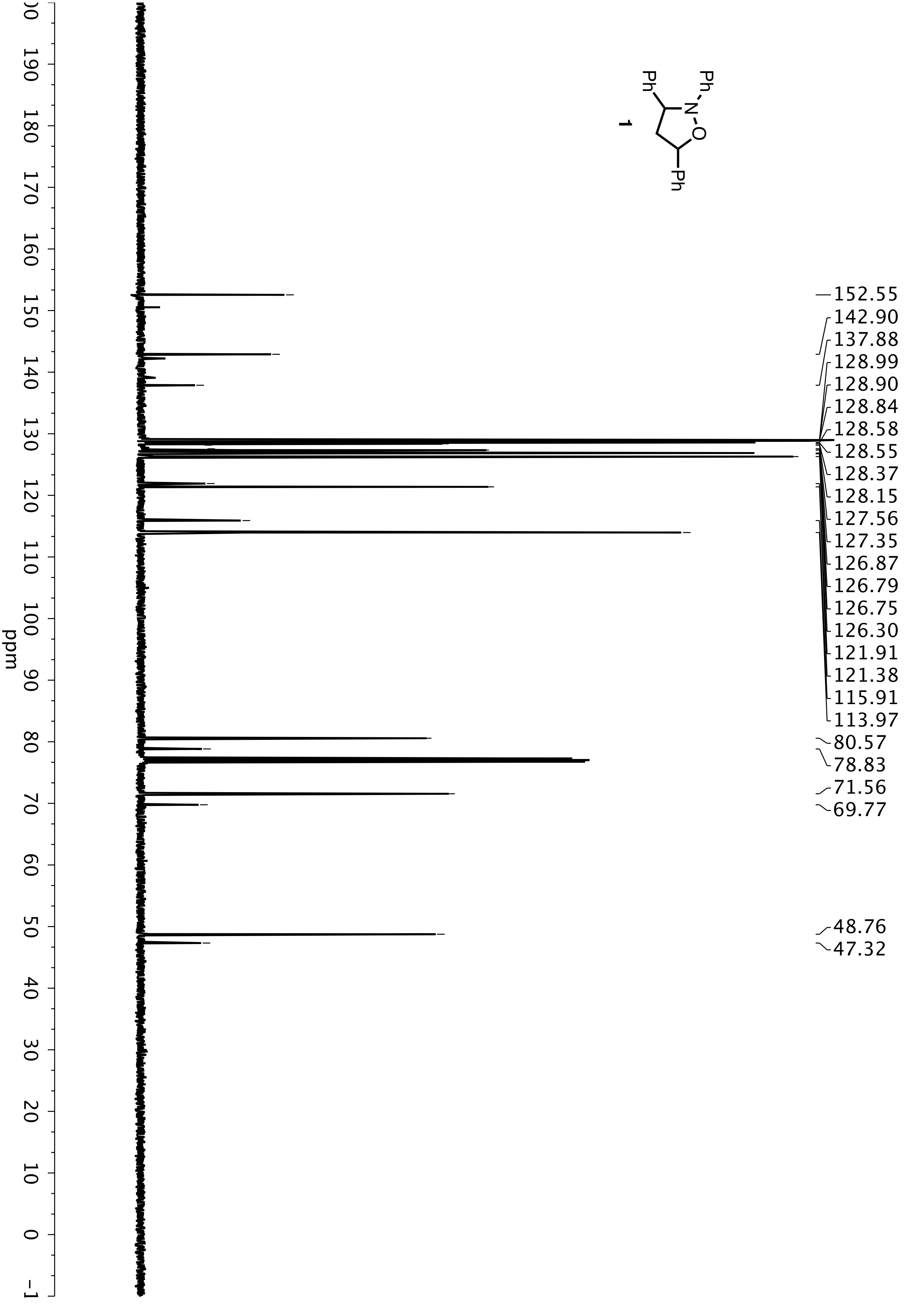

152.55
142.90
137.88
128.99
128.90
128.84
128.58
128.55
128.37
128.15
127.56
127.35
126.87
126.79
126.75
126.30
121.91
121.38
115.91
113.97
80.57
78.83
71.56
69.77
48.76
47.32
ppm
1

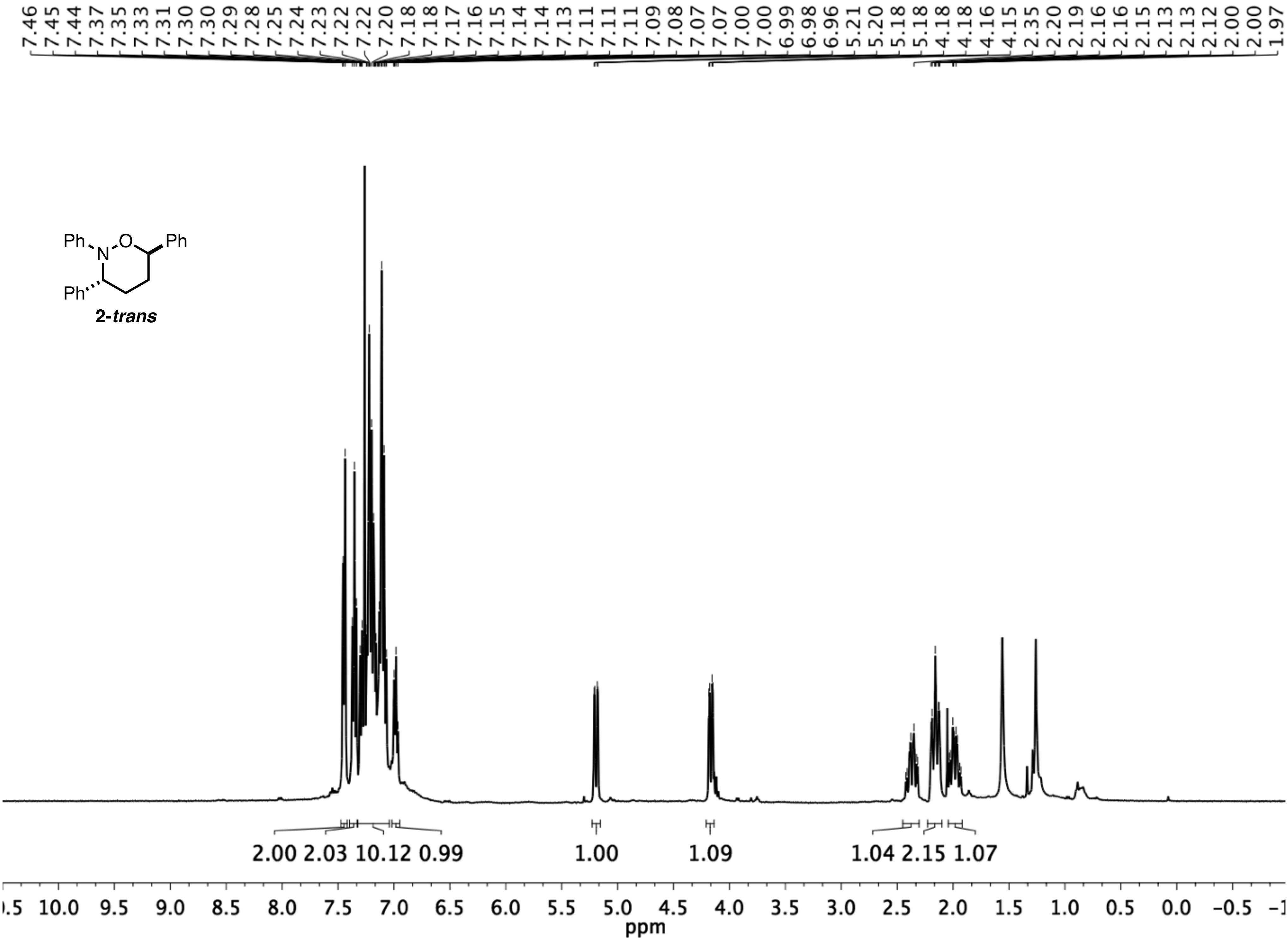

7.46 7.45 7.44 7.37 7.35 7.33 7.31 7.30 7.30 7.29 7.28 7.25 7.24 7.23 7.22 7.22 7.20 7.18 7.18 7.17 7.16 7.15 7.14 7.14 7.13 7.11 7.11 7.11 7.09 7.08 7.07 7.07 7.00 7.00 6.99 6.98 6.96 5.21 5.20 5.18 5.18 4.18 4.18 4.16 4.15 2.35 2.20 2.19 2.16 2.16 2.15 2.13 2.13 2.12 2.00 2.00 1.97
Ph
N
O
Ph
Ph
2-trans
2.00 2.03 10.12 0.99
1.00
1.09
1.04 2.15 1.07
10.0 9.5 9.0 8.5 8.0 7.5 7.0 6.5 6.0 5.5 5.0 4.5 4.0 3.5 3.0 2.5 2.0 1.5 1.0 0.5 0.0 –0.5
ppm

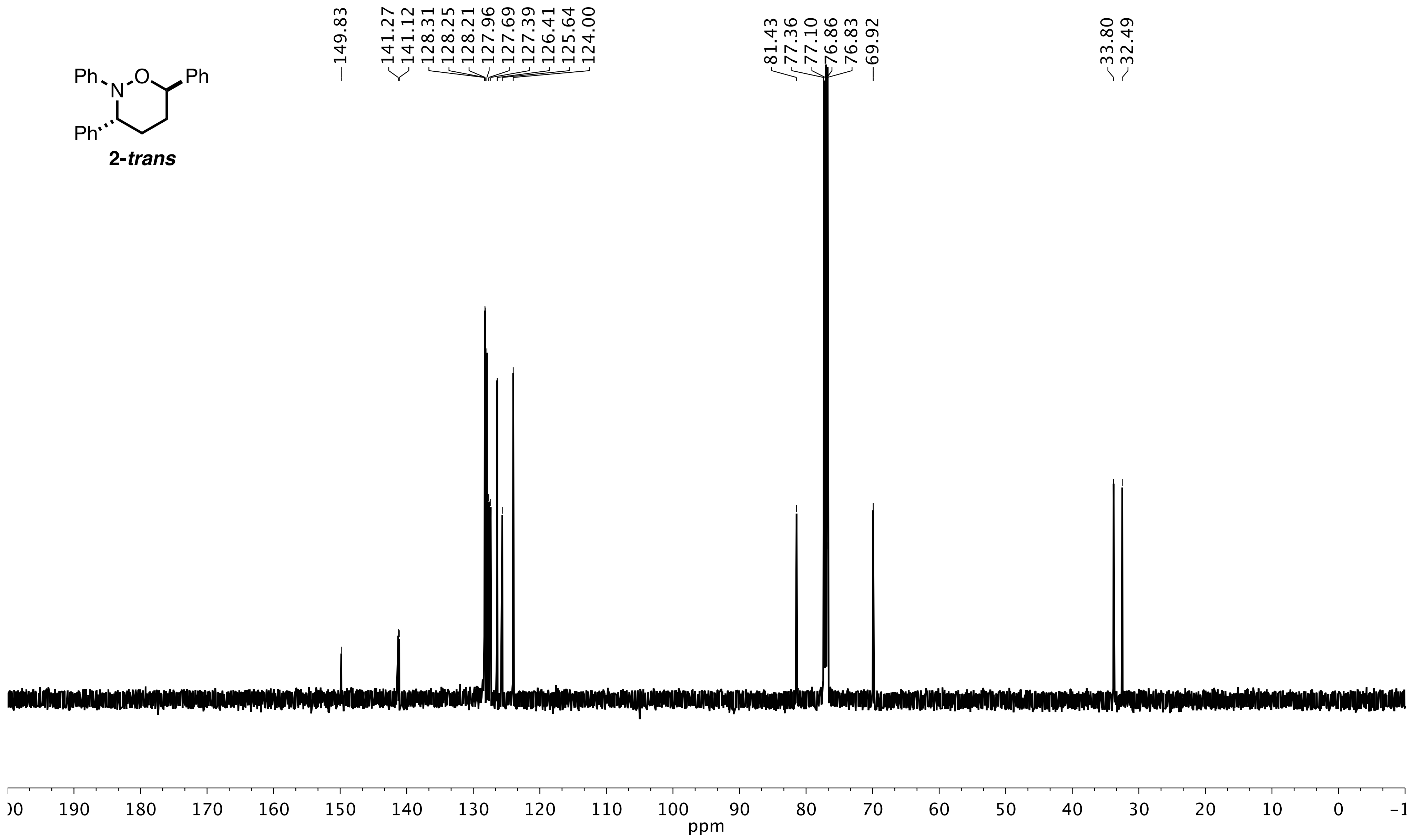
Ph
N
O
Ph
Ph
2-trans
149.83
141.27
141.12
128.31
128.25
128.21
127.96
127.69
127.39
126.41
125.64
124.00
81.43
77.36
77.10
76.86
76.83
69.92
33.80
32.49
190
180
170
160
150
140
130
120
110
100
90
80
70
60
50
40
30
20
10
0
ppm

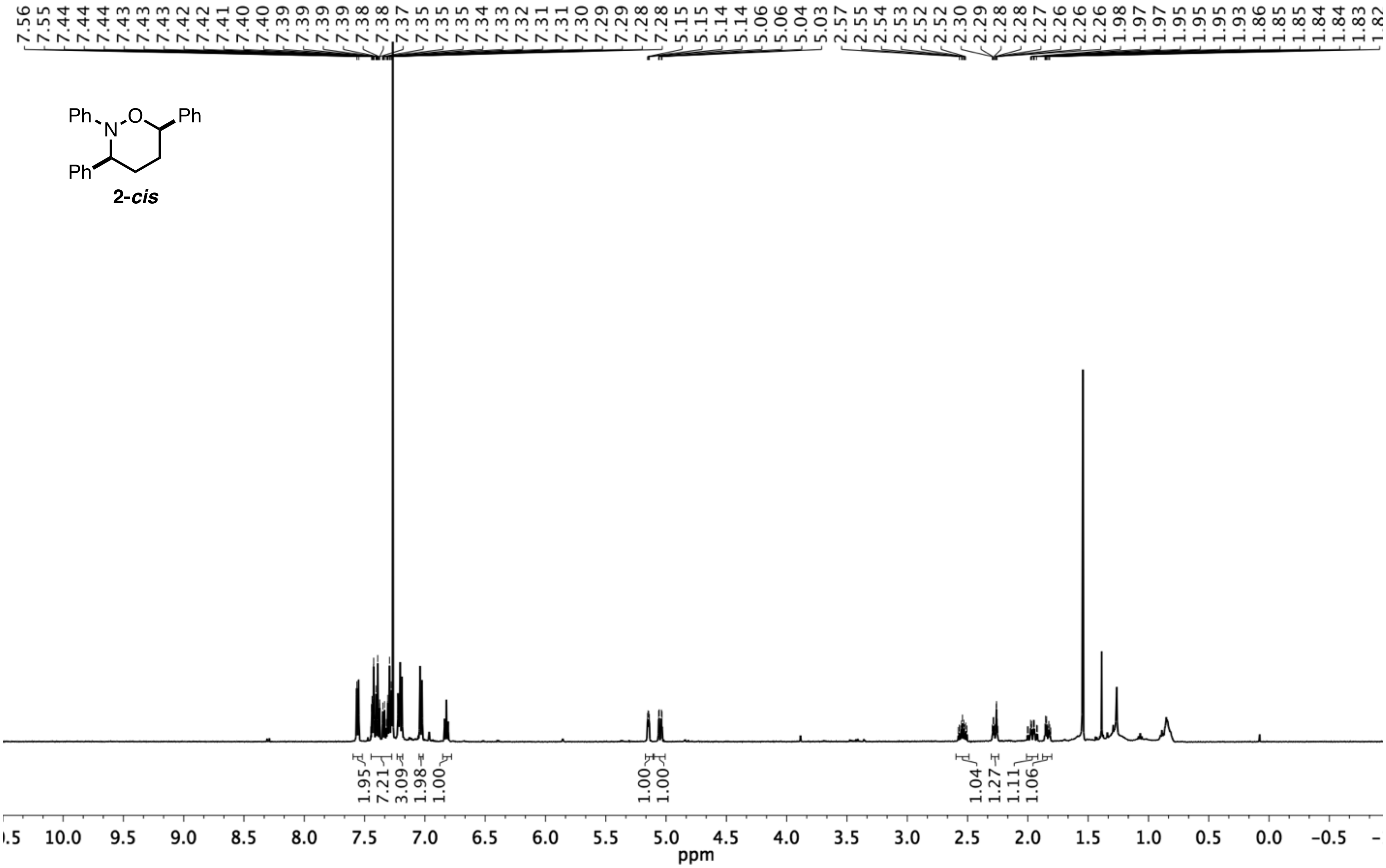
7.56
7.55
7.44
7.44
7.44
7.43
7.43
7.43
7.42
7.42
7.41
7.40
7.40
7.39
7.39
7.39
7.39
7.38
7.38
7.37
7.35
7.35
7.35
7.34
7.33
7.32
7.31
7.31
7.30
7.29
7.29
7.28
7.28
5.15
5.15
5.14
5.14
5.06
5.06
5.04
5.03
2.57
2.55
2.54
2.53
2.52
2.52
2.30
2.29
2.28
2.28
2.27
2.26
2.26
2.26
1.98
1.97
1.97
1.95
1.95
1.95
1.93
1.86
1.85
1.85
1.84
1.84
1.83
1.82
Ph
N
O
Ph
Ph
2-cis
1.95
7.21
3.09
1.98
1.00
1.00
1.00
1.04
1.27
1.11
1.06
10.0
9.5
9.0
8.5
8.0
7.5
7.0
6.5
6.0
5.5
5.0
4.5
4.0
3.5
3.0
2.5
2.0
1.5
1.0
0.5
0.0
−0.5
ppm

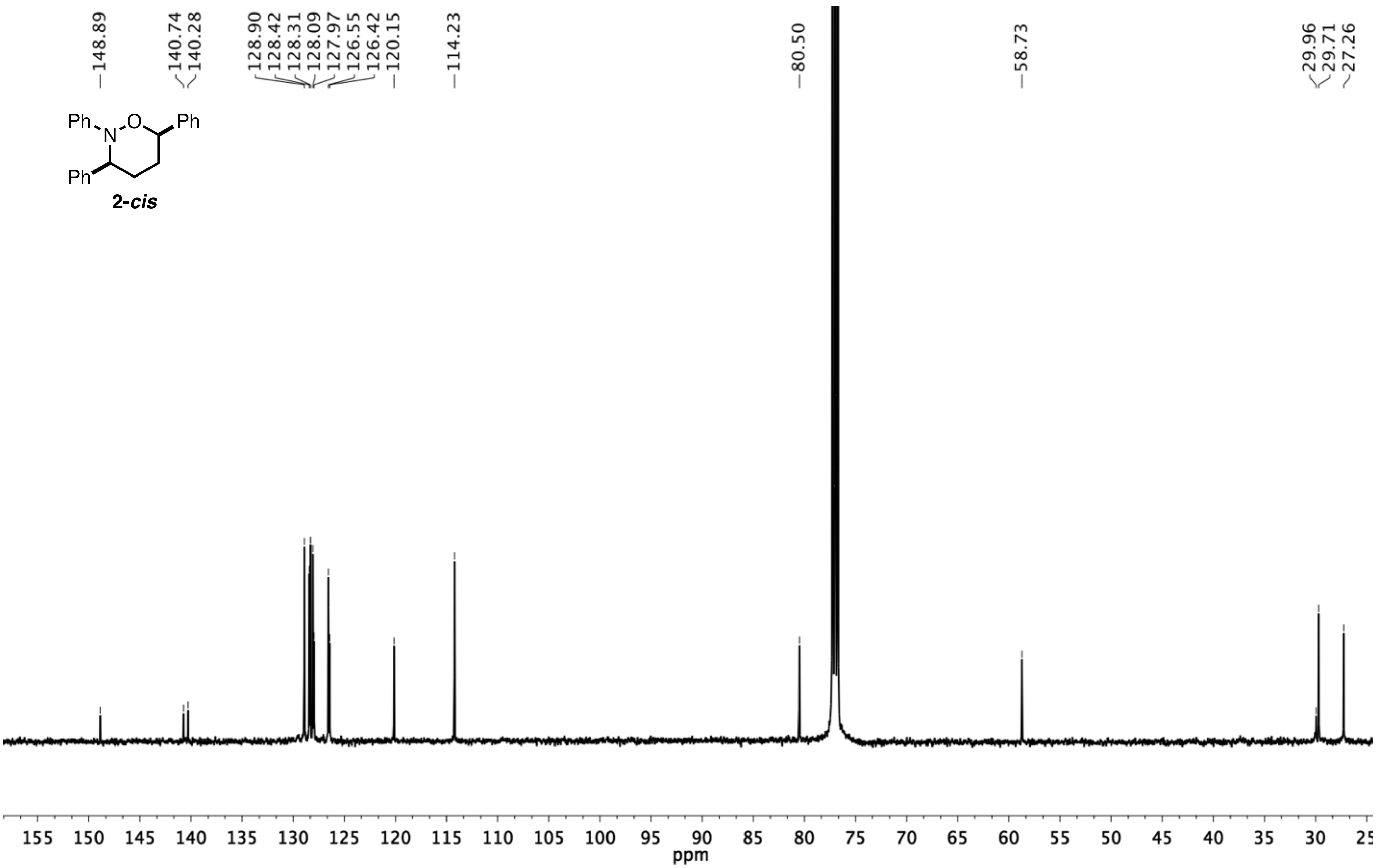
148.89
140.74
140.28
128.90
128.42
128.31
128.09
127.97
126.55
126.42
120.15
114.23
80.50
58.73
29.96
29.71
27.26
Ph
N
O
Ph
Ph
2-cis
155 150 145 140 135 130 125 120 115 110 105 100 95 90 85 80 75 70 65 60 55 50 45 40 35 30
ppm

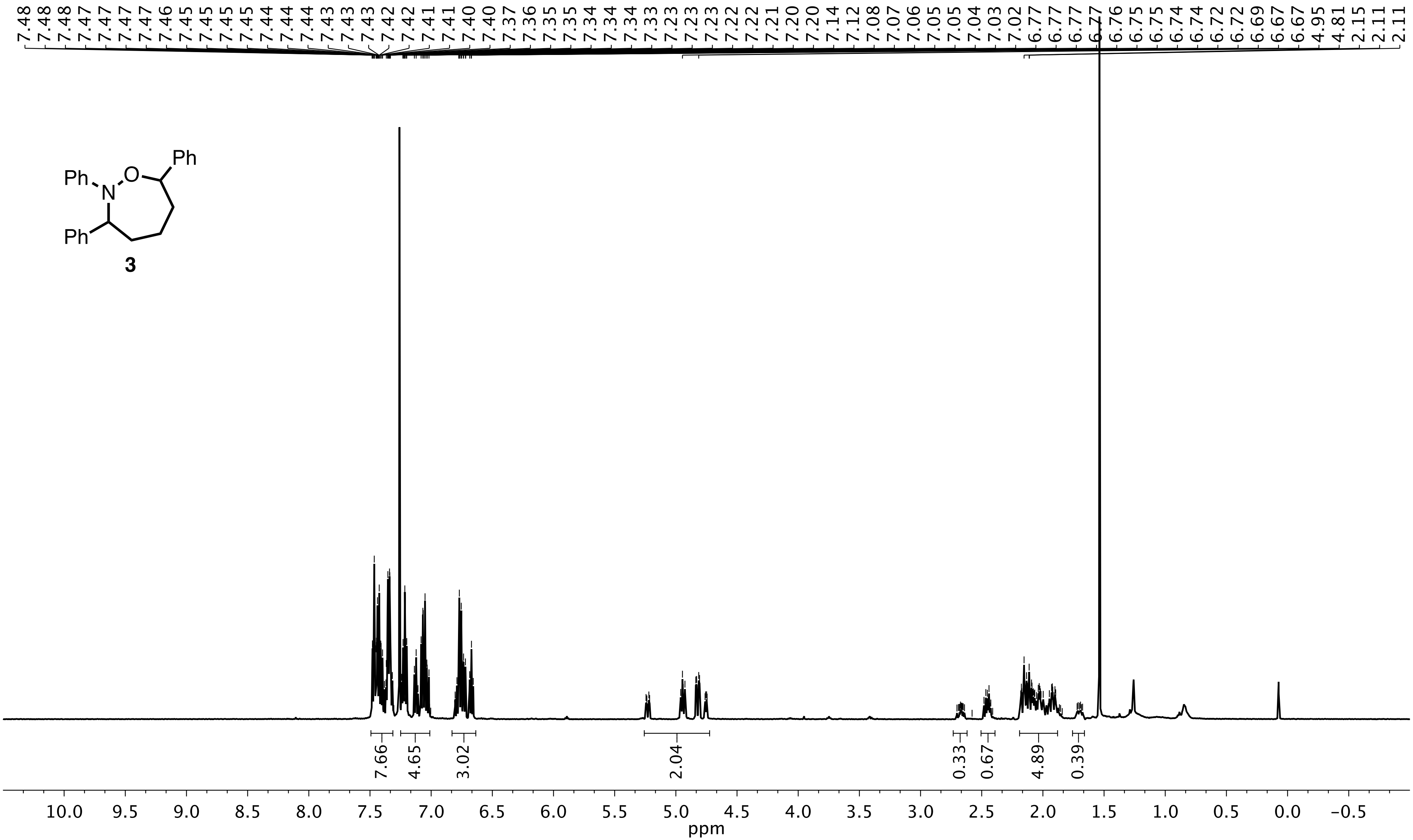
7.48 7.48 7.48 7.47 7.47 7.47 7.47 7.46 7.45 7.45 7.45 7.45 7.44 7.44 7.44 7.43 7.43 7.43 7.42 7.42 7.41 7.41 7.40 7.40 7.37 7.36 7.35 7.35 7.34 7.34 7.34 7.33 7.23 7.23 7.23 7.22 7.22 7.22 7.21 7.20 7.20 7.14 7.12 7.08 7.07 7.06 7.05 7.05 7.04 7.03 7.02 6.77 6.77 6.77 6.77 6.76 6.75 6.75 6.74 6.74 6.72 6.72 6.69 6.67 6.67 4.95 4.81 2.15 2.11 2.11
Ph
Ph
O
N
Ph
3
7.66
4.65
3.02
2.04
0.33
0.67
4.89
0.39
10.0 9.5 9.0 8.5 8.0 7.5 7.0 6.5 6.0 5.5 5.0 4.5 4.0 3.5 3.0 2.5 2.0 1.5 1.0 0.5 0.0 −0.5
ppm

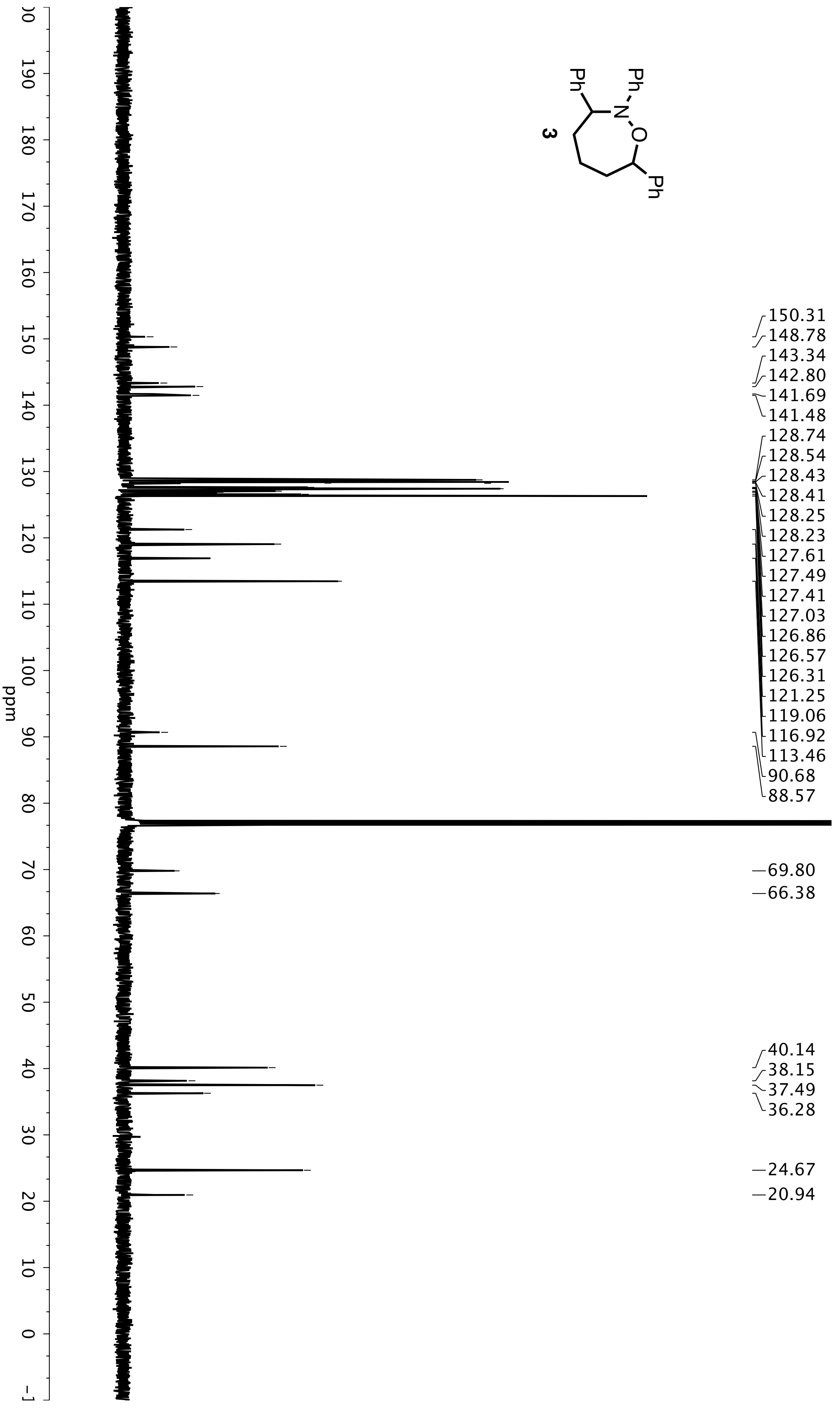
Ph
N
O
Ph
Ph
3
150.31
148.78
143.34
142.80
141.69
141.48
128.74
128.54
128.43
128.41
128.25
128.23
127.61
127.49
127.41
127.03
126.86
126.57
126.31
121.25
119.06
116.92
113.46
90.68
88.57
69.80
66.38
40.14
38.15
37.49
36.28
24.67
20.94
ppm

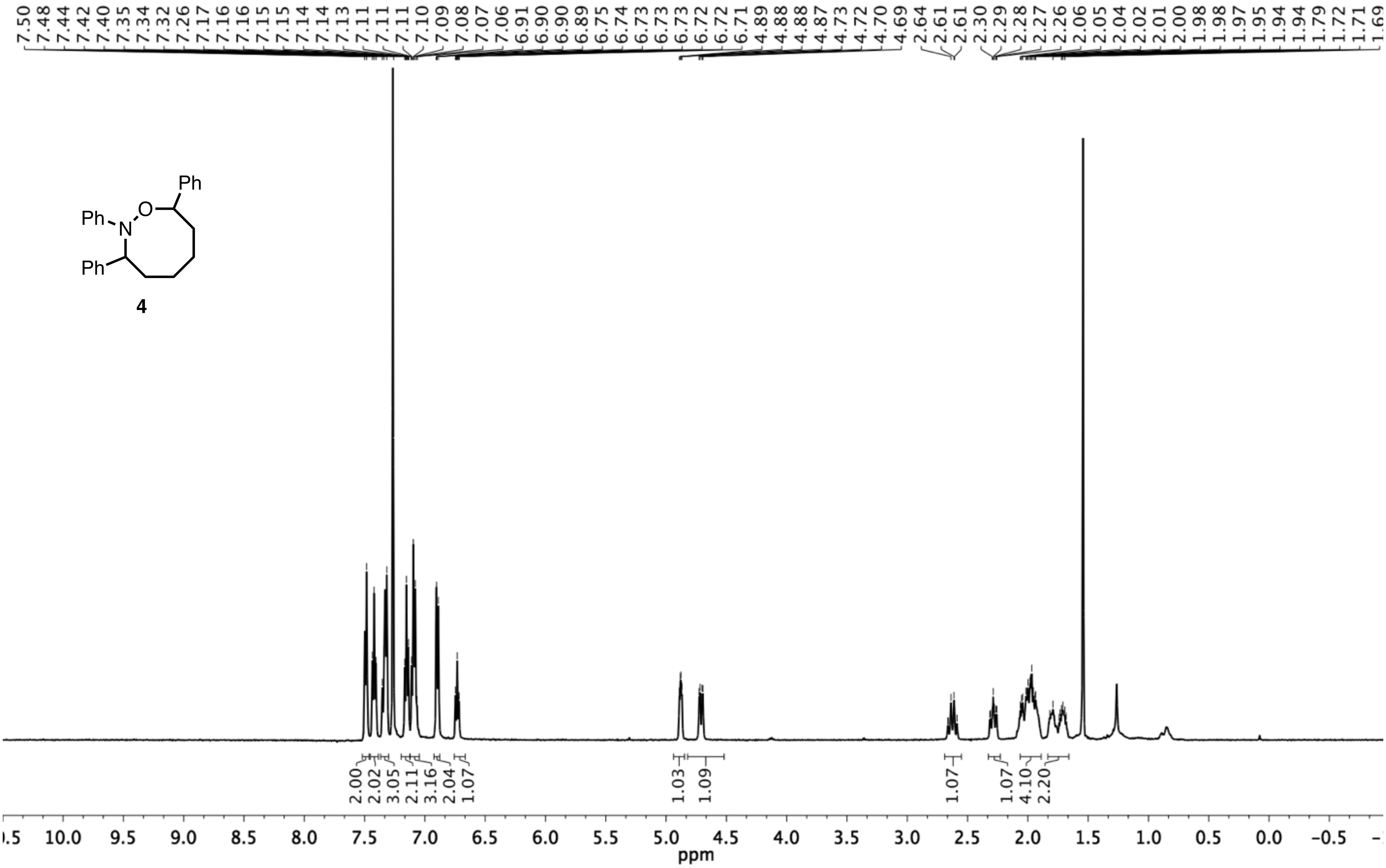
7.50
7.48
7.44
7.42
7.40
7.35
7.34
7.32
7.26
7.17
7.16
7.16
7.15
7.15
7.14
7.14
7.13
7.11
7.11
7.11
7.10
7.09
7.08
7.07
7.06
6.91
6.90
6.90
6.89
6.75
6.74
6.73
6.73
6.73
6.72
6.72
6.71
4.89
4.88
4.88
4.87
4.73
4.72
4.70
4.69
2.64
2.61
2.61
2.30
2.29
2.28
2.27
2.26
2.06
2.05
2.04
2.02
2.01
2.00
1.98
1.98
1.97
1.95
1.94
1.94
1.79
1.72
1.71
1.69
Ph
Ph
N
O
Ph
4
2.00
2.02
3.05
2.11
3.16
2.04
1.07
1.03
1.09
1.07
1.07
4.10
2.20
10.0 9.5 9.0 8.5 8.0 7.5 7.0 6.5 6.0 5.5 5.0 4.5 4.0 3.5 3.0 2.5 2.0 1.5 1.0 0.5 0.0 −0.5
ppm

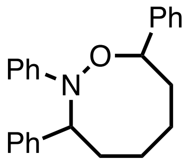
Ph
Ph
N
O
Ph

4

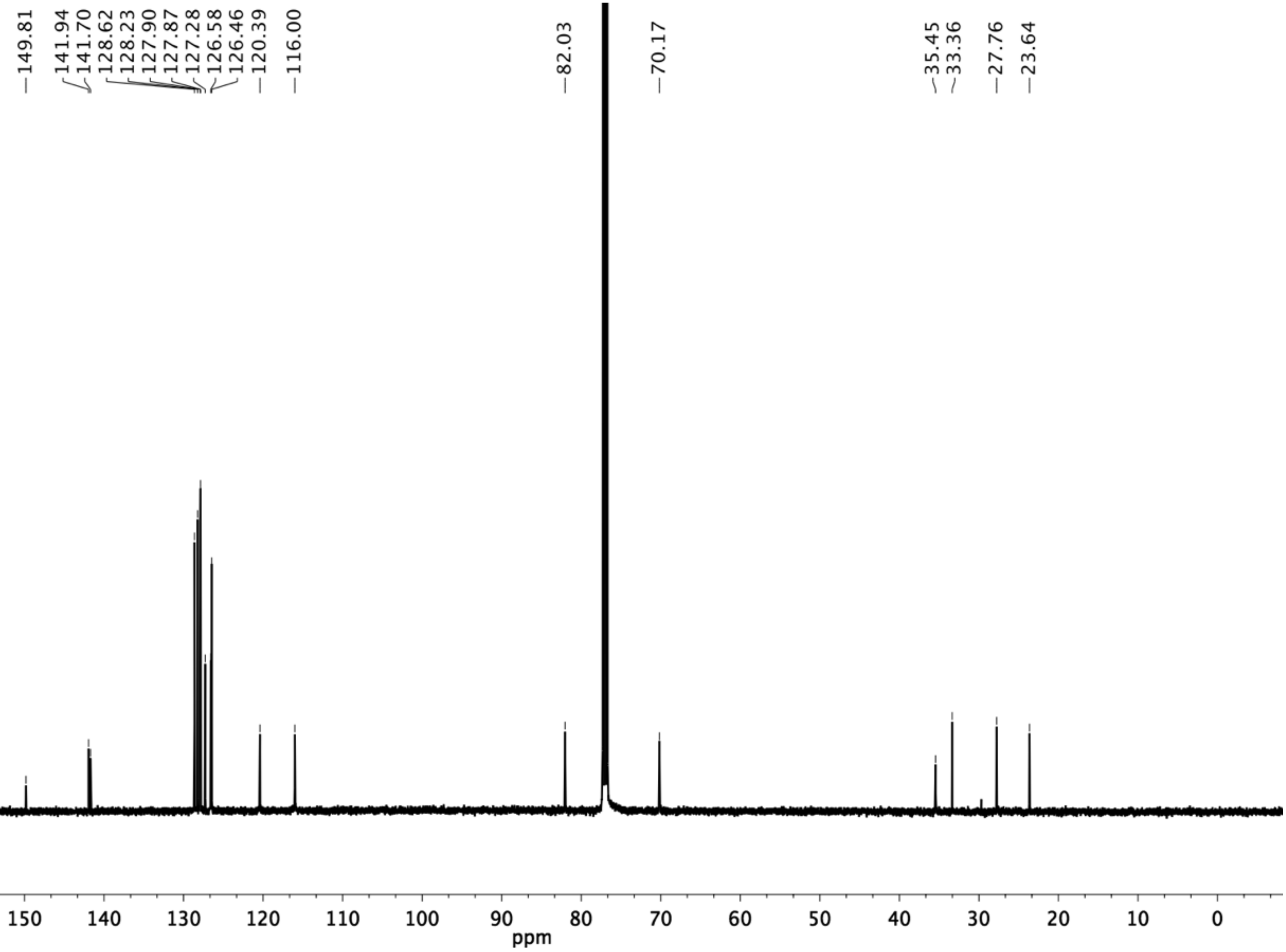
149.81
141.94
141.70
128.62
128.23
127.90
127.87
127.28
126.58
126.46
120.39
116.00
82.03
70.17
35.45
33.36
27.76
23.64
180 170 160 150 140 130 120 110 100 90 80 70 60 50 40 30 20 10 0
ppm

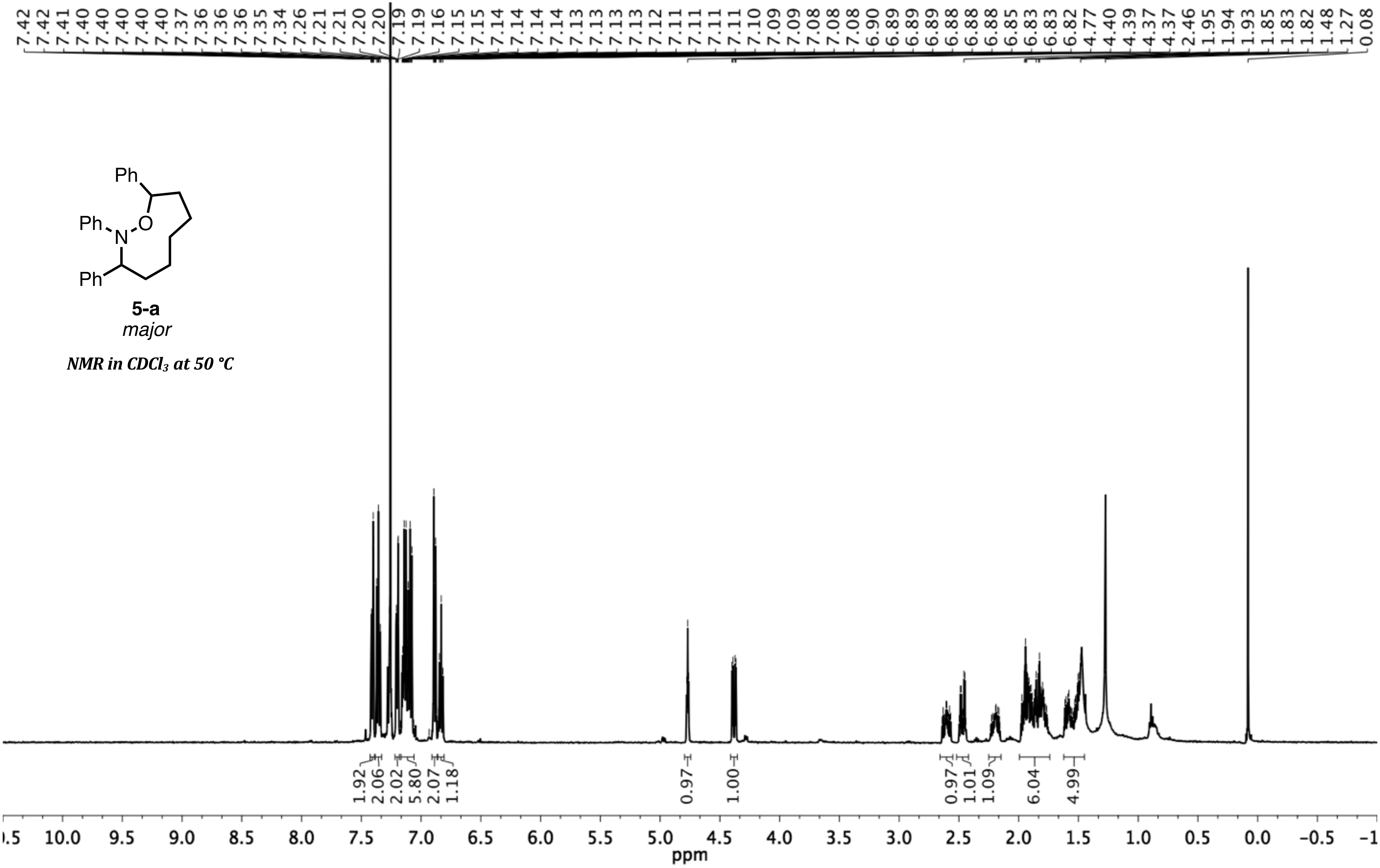

5-a
major

NMR in CDCl₃ at 50 °C

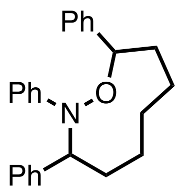

5-a
major

NMR in $CDCl_3$ at 50 °C

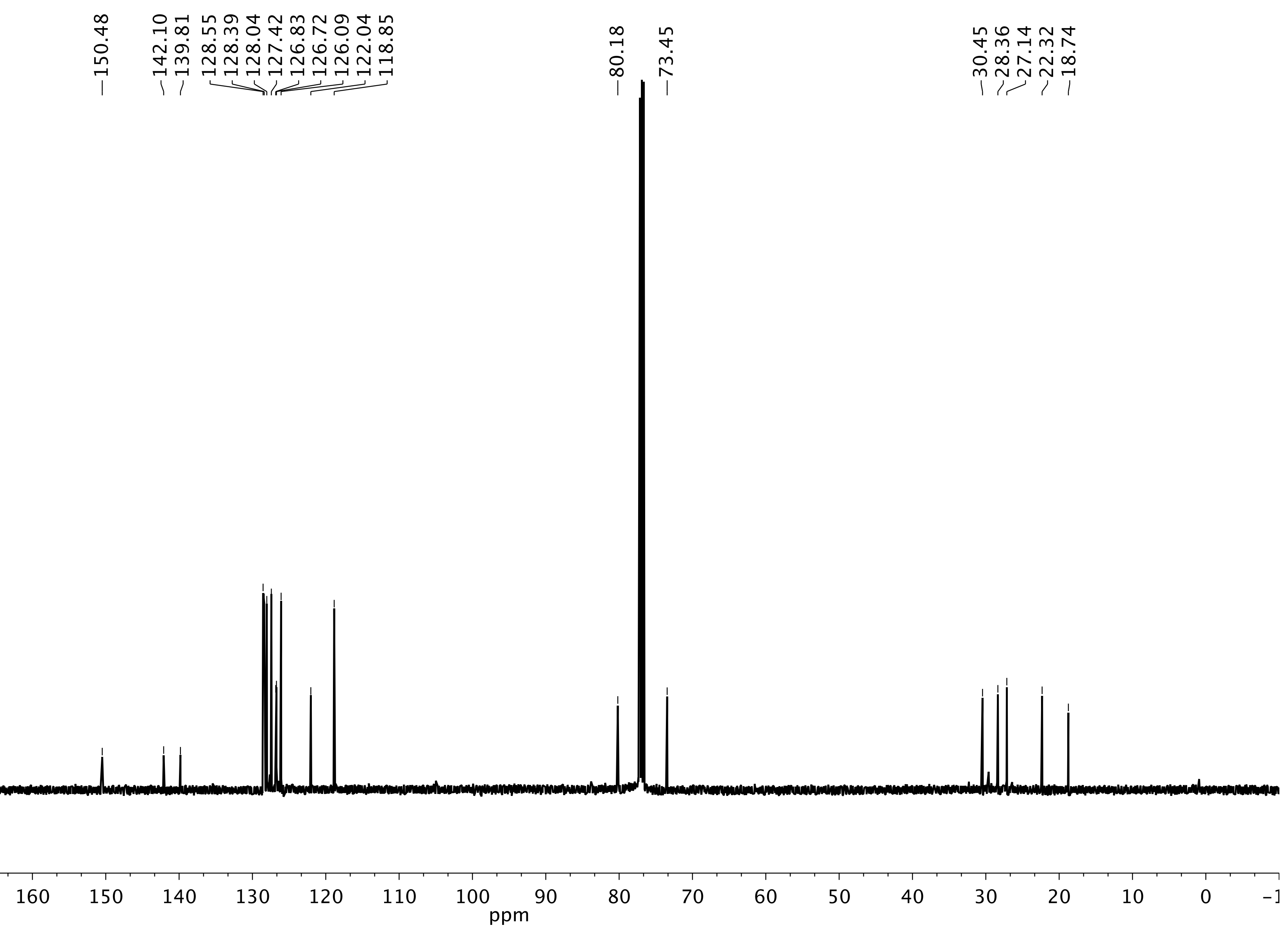

5-b
minor

NMR in $CDCl_3$ at 50 °C

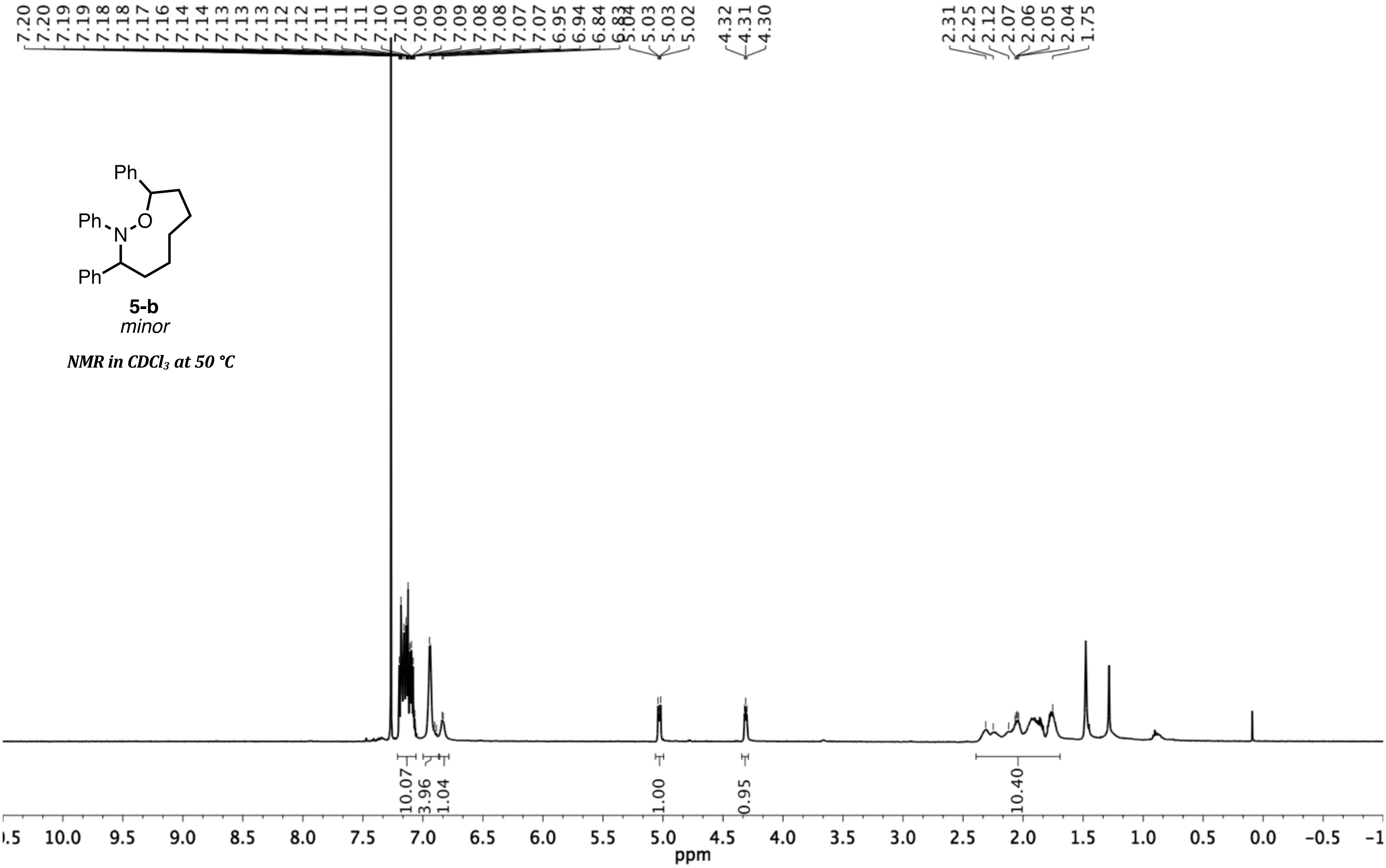

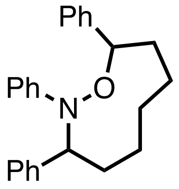

5-b
minor

NMR in $CDCl_3$ at 50 °C

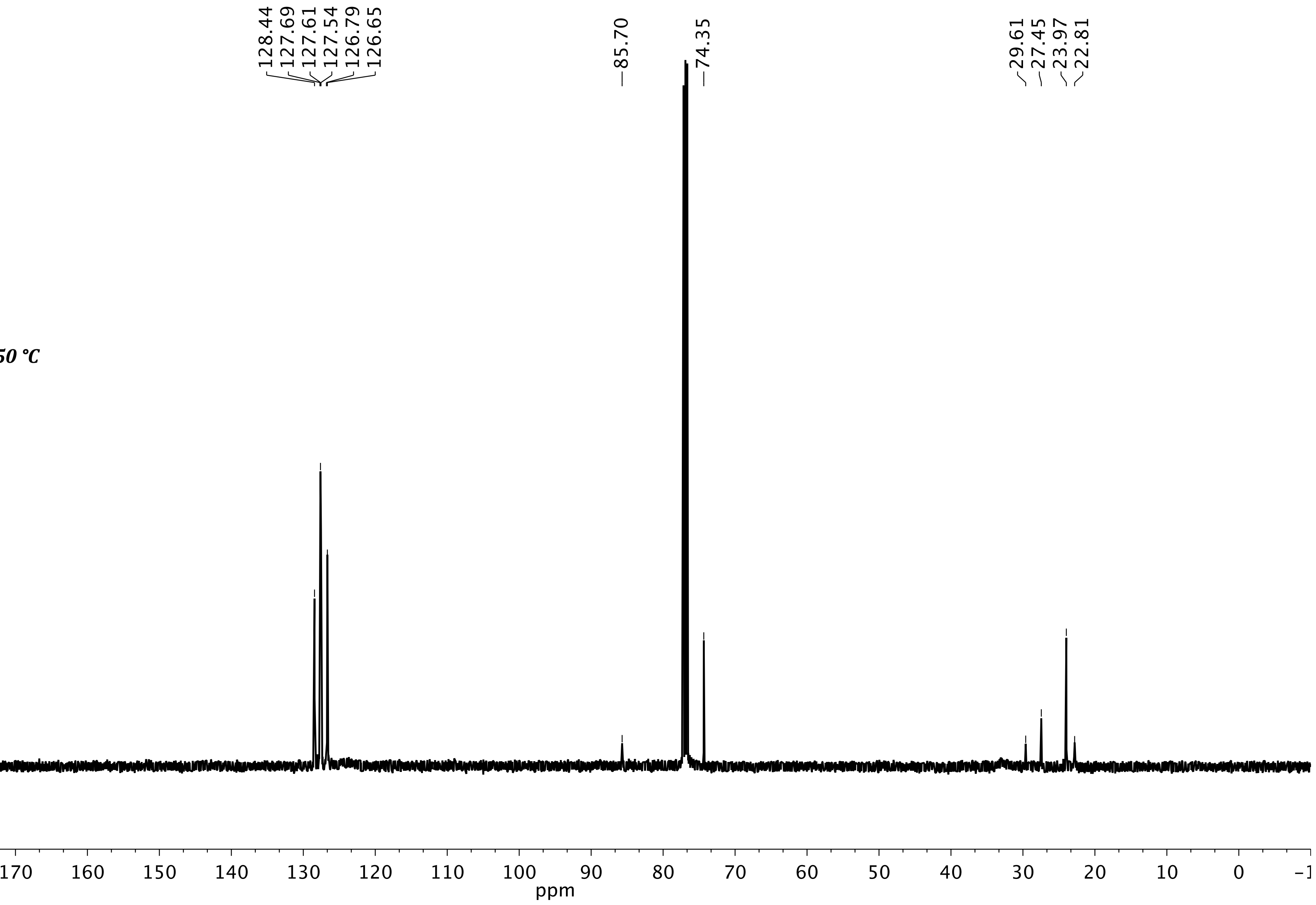

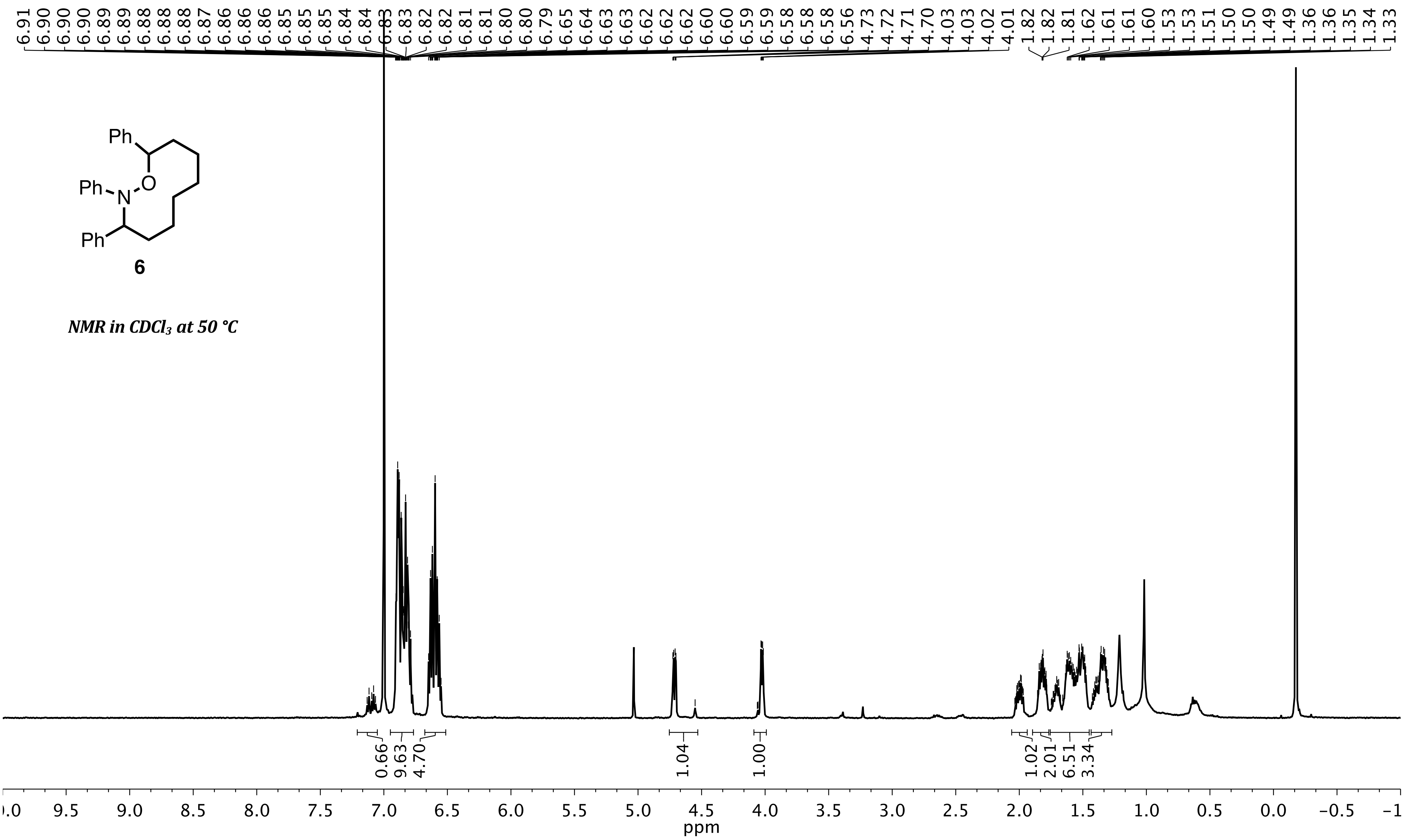
6.91 6.90 6.90 6.90 6.89 6.89 6.88 6.88 6.88 6.88 6.87 6.86 6.86 6.86 6.85 6.85 6.85 6.84 6.84 6.83 6.83 6.82 6.82 6.81 6.81 6.80 6.80 6.79 6.65 6.64 6.63 6.63 6.62 6.62 6.62 6.60 6.60 6.59 6.59 6.58 6.58 6.58 6.56 4.73 4.72 4.71 4.70 4.03 4.03 4.02 4.01 1.82 1.82 1.81 1.62 1.61 1.61 1.60 1.53 1.53 1.51 1.50 1.50 1.49 1.49 1.36 1.36 1.35 1.34 1.33
Ph
Ph
N
O
Ph
6
NMR in CDCl3 at 50 °C
0.66
9.63
4.70
1.04
1.00
1.02
2.01
6.51
3.34
10.0 9.5 9.0 8.5 8.0 7.5 7.0 6.5 6.0 5.5 5.0 4.5 4.0 3.5 3.0 2.5 2.0 1.5 1.0 0.5 0.0 −0.5 −1
ppm

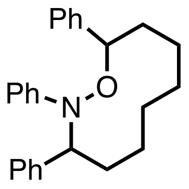

6

NMR in CDCl$_3$ at 50 °C

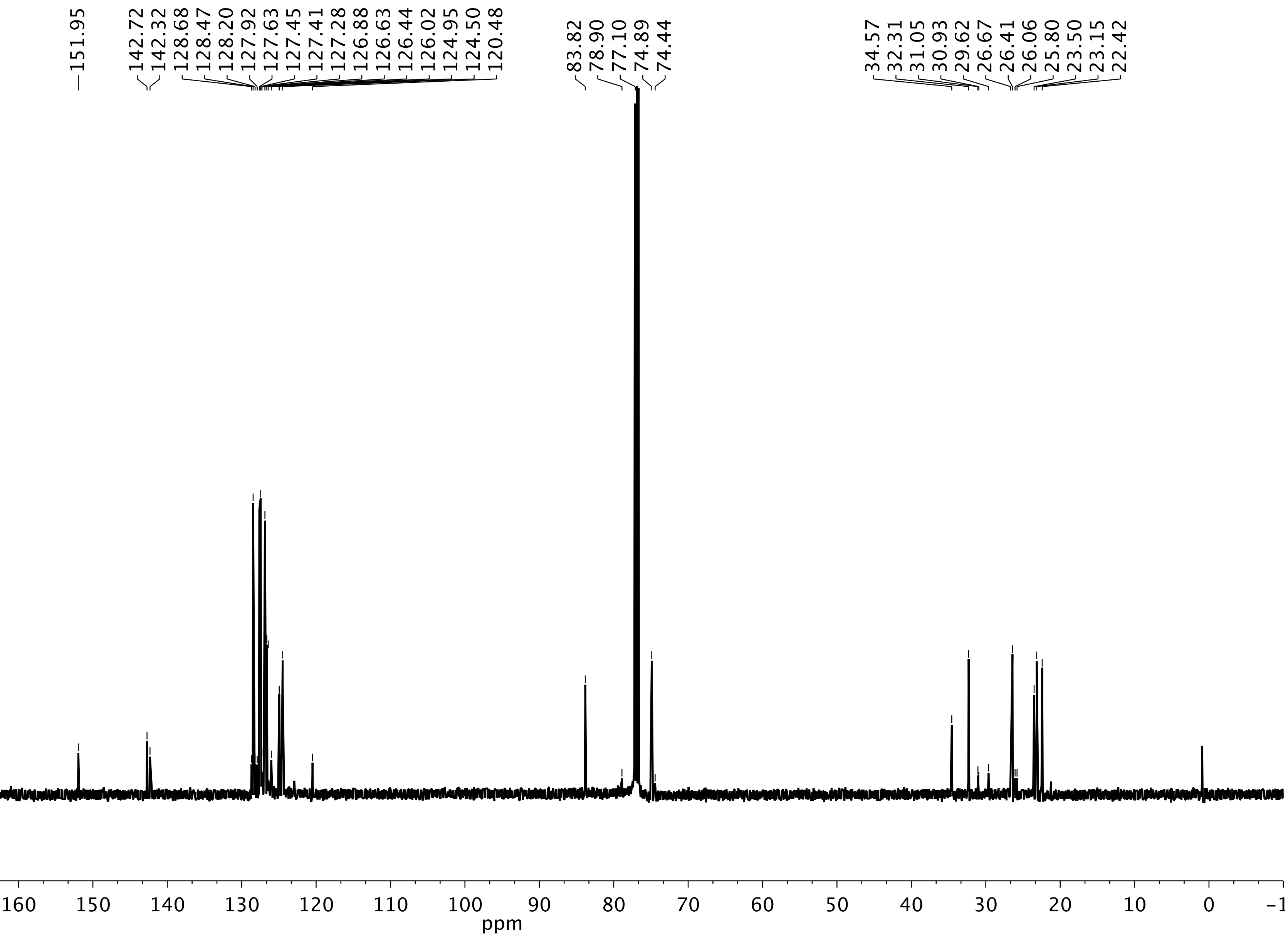

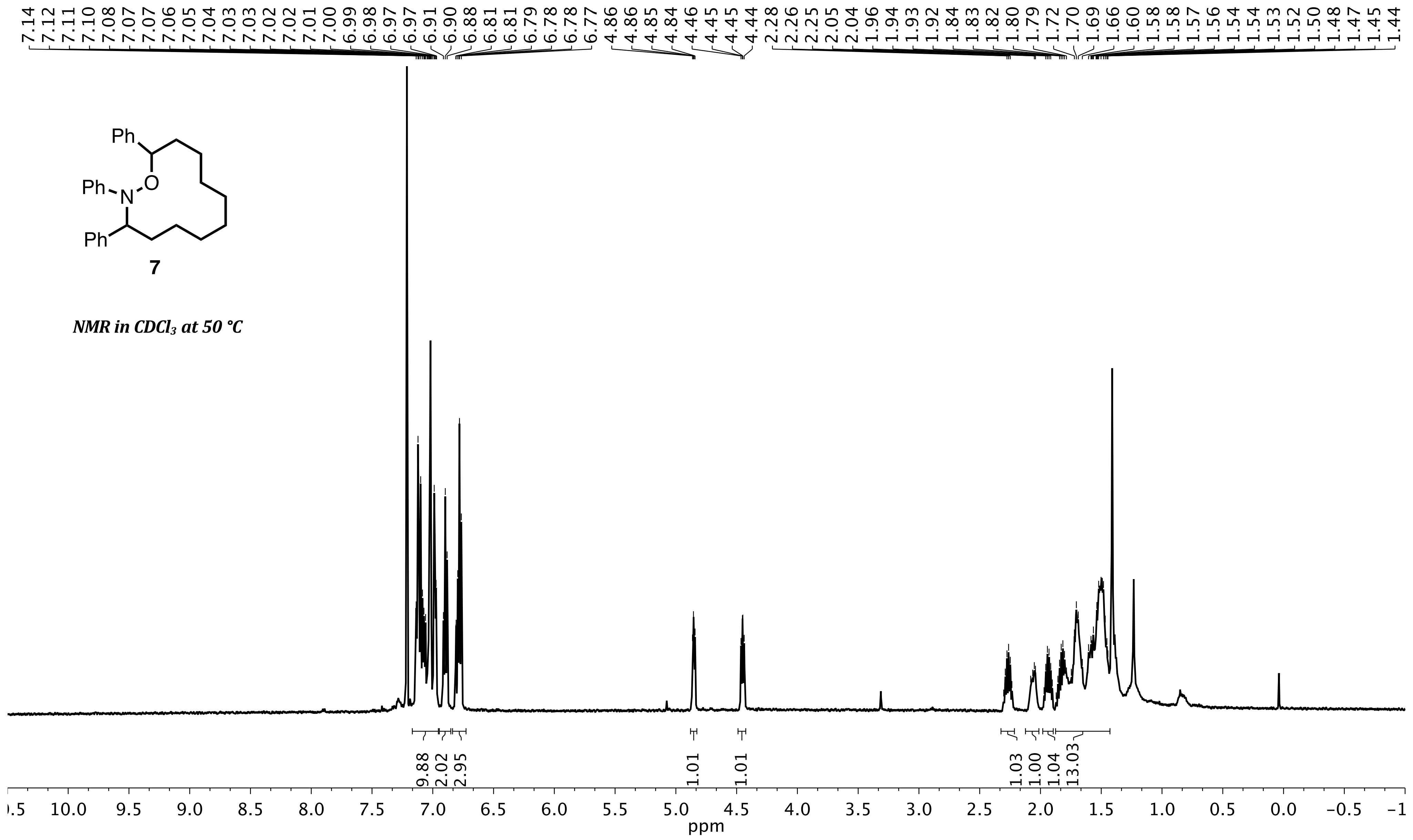
Ph
Ph
N
O
Ph
7
NMR in CDCl3 at 50 °C
7.14 7.12 7.11 7.10 7.08 7.07 7.07 7.06 7.05 7.04 7.03 7.03 7.02 7.02 7.01 7.00 6.99 6.98 6.97 6.97 6.91 6.90 6.88 6.81 6.81 6.79 6.78 6.78 6.77 4.86 4.86 4.85 4.84 4.46 4.45 4.45 4.44 2.28 2.26 2.25 2.05 2.04 1.96 1.94 1.93 1.92 1.84 1.83 1.82 1.80 1.79 1.72 1.70 1.69 1.66 1.60 1.58 1.58 1.57 1.56 1.54 1.54 1.53 1.52 1.50 1.48 1.47 1.45 1.44
9.88 2.02 2.95 1.01 1.01 1.03 1.00 1.04 13.03
10.0 9.5 9.0 8.5 8.0 7.5 7.0 6.5 6.0 5.5 5.0 4.5 4.0 3.5 3.0 2.5 2.0 1.5 1.0 0.5 0.0 −0.5 −1
ppm

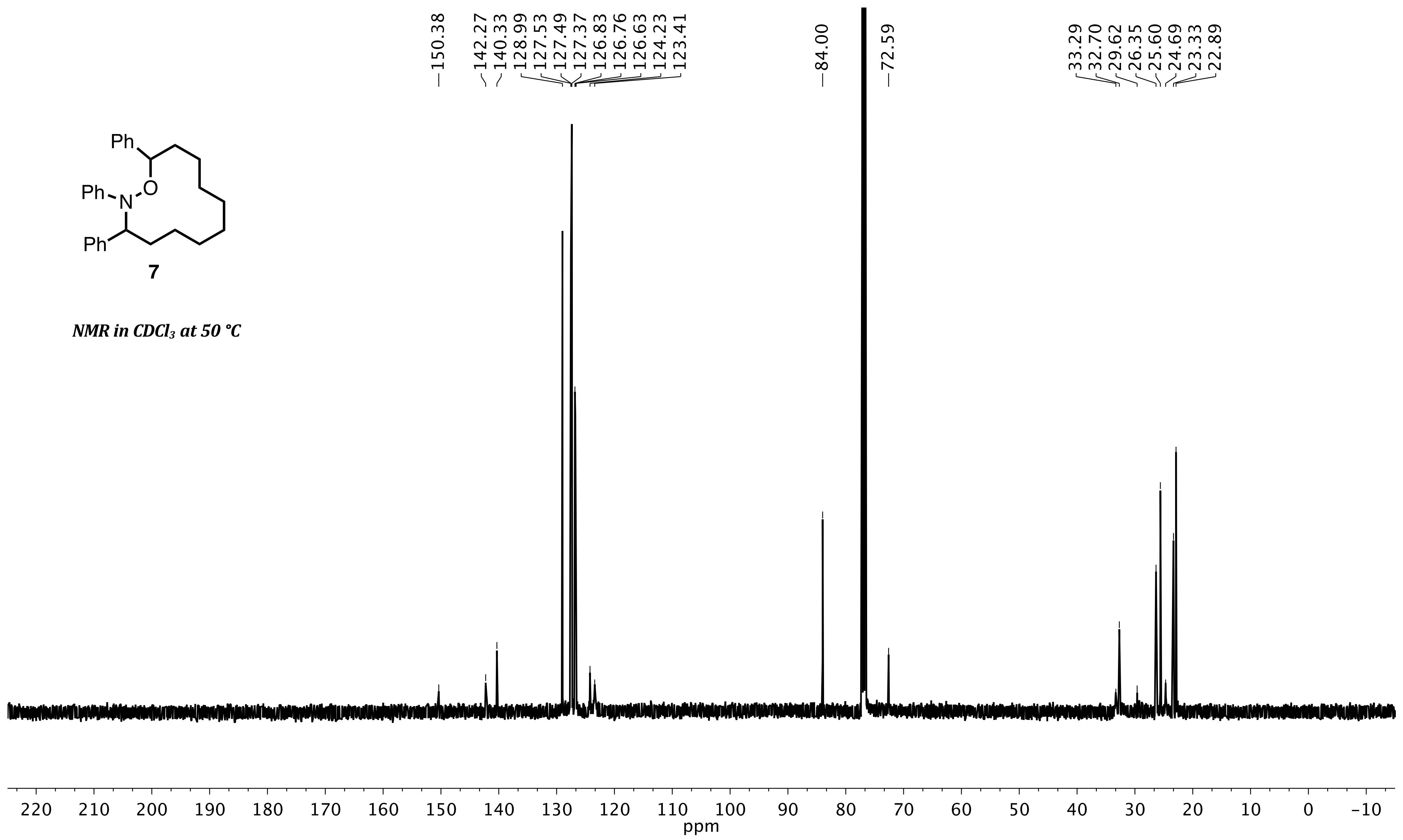

Ph
Ph
N
O
Ph
7
NMR in CDCl₃ at 50 °C
150.38
142.27
140.33
128.99
127.53
127.49
127.37
126.83
126.76
126.63
124.23
123.41
84.00
72.59
33.29
32.70
29.62
26.35
25.60
24.69
23.33
22.89
220 210 200 190 180 170 160 150 140 130 120 110 100 90 80 70 60 50 40 30 20 10 0 −10
ppm

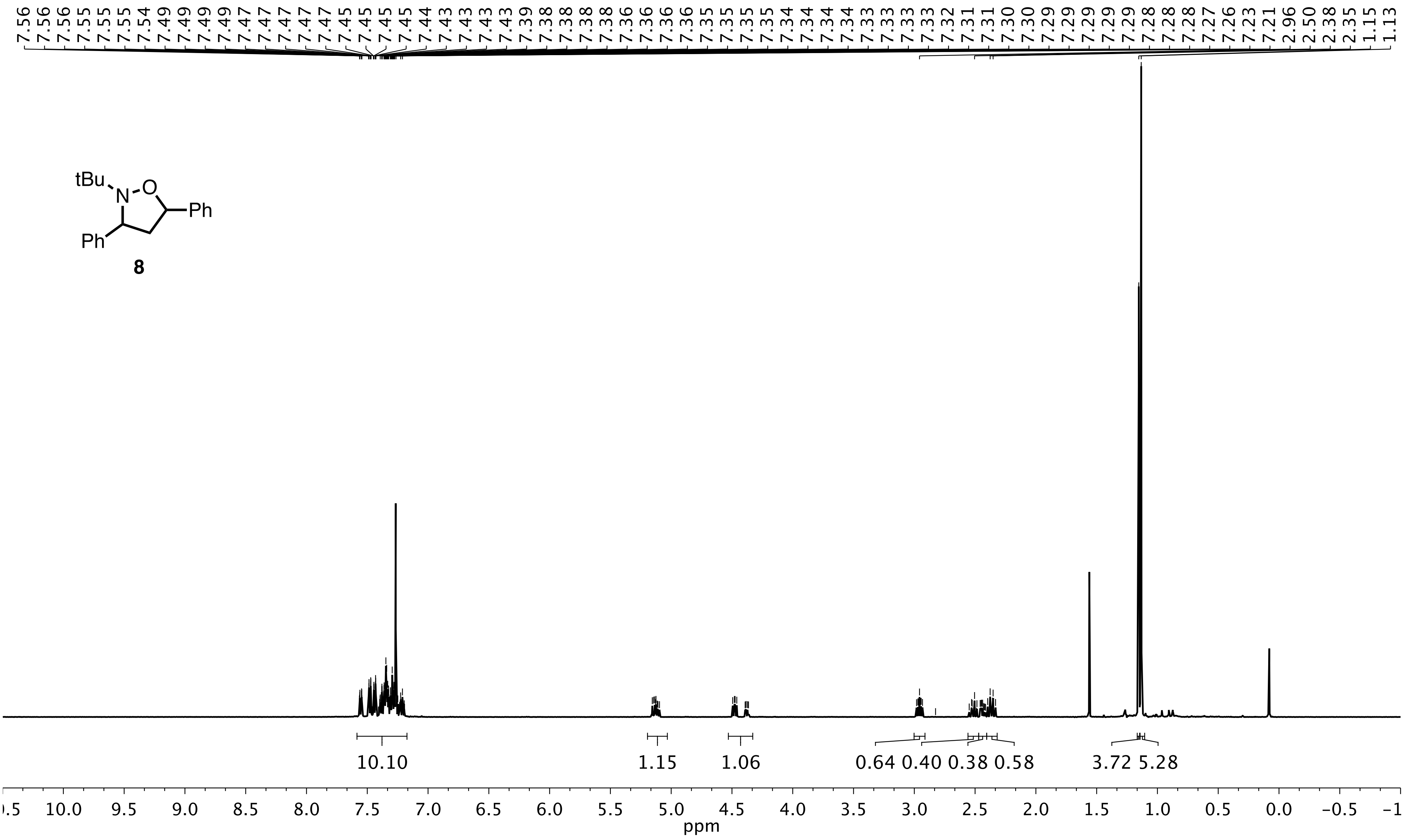
7.56
7.56
7.56
7.55
7.55
7.55
7.54
7.49
7.49
7.49
7.49
7.47
7.47
7.47
7.47
7.47
7.45
7.45
7.45
7.45
7.44
7.43
7.43
7.43
7.43
7.39
7.38
7.38
7.38
7.38
7.36
7.36
7.36
7.36
7.35
7.35
7.35
7.35
7.34
7.34
7.34
7.34
7.33
7.33
7.33
7.33
7.32
7.31
7.31
7.30
7.30
7.29
7.29
7.29
7.29
7.29
7.28
7.28
7.28
7.27
7.26
7.23
7.21
2.96
2.50
2.38
2.35
1.15
1.13
tBu
N
O
Ph
Ph
8
10.10
1.15
1.06
0.64 0.40 0.38 0.58
3.72 5.28
10.0 9.5 9.0 8.5 8.0 7.5 7.0 6.5 6.0 5.5 5.0 4.5 4.0 3.5 3.0 2.5 2.0 1.5 1.0 0.5 0.0 −0.5
ppm

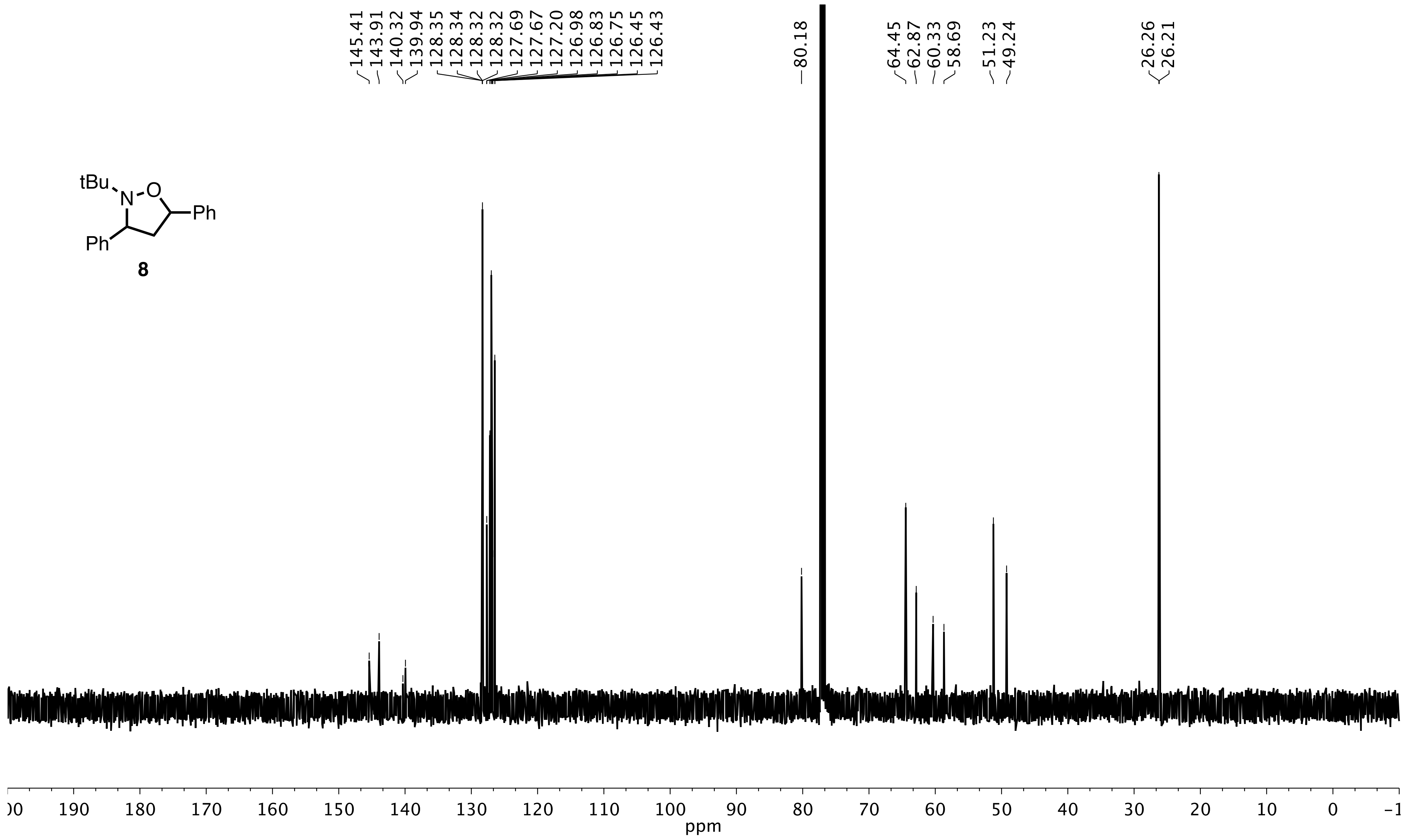

tBu
N
O
Ph
Ph
8
145.41
143.91
140.32
139.94
128.35
128.34
128.32
128.32
127.69
127.67
127.20
126.98
126.83
126.75
126.45
126.43
80.18
64.45
62.87
60.33
58.69
51.23
49.24
26.26
26.21
190
180
170
160
150
140
130
120
110
100
90
80
70
60
50
40
30
20
10
0
ppm

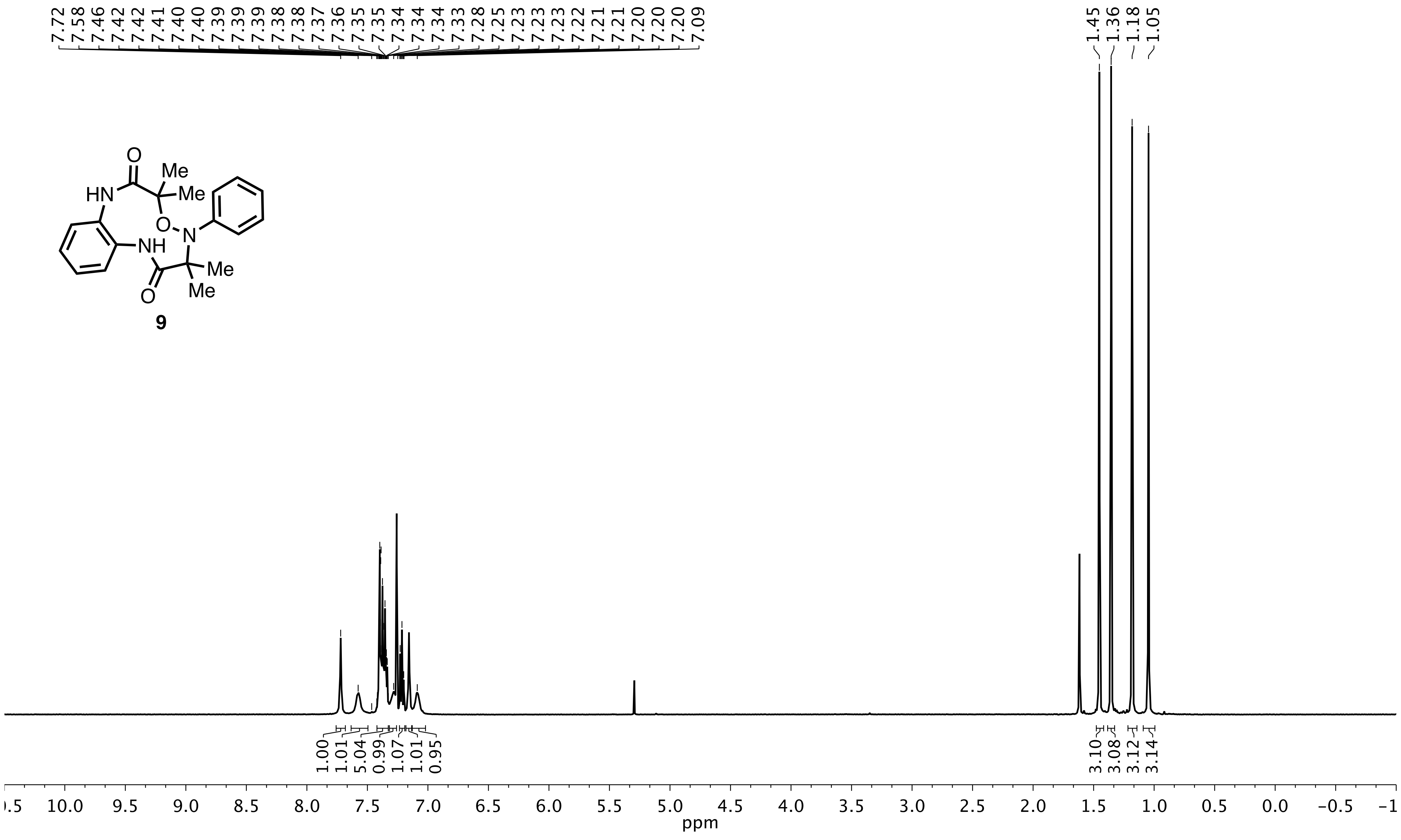

7.72
7.58
7.46
7.42
7.42
7.41
7.40
7.40
7.39
7.39
7.39
7.38
7.38
7.37
7.36
7.35
7.35
7.34
7.34
7.34
7.33
7.28
7.25
7.23
7.23
7.23
7.22
7.21
7.21
7.20
7.20
7.20
7.09
1.45
1.36
1.18
1.05
HN
NH
O
Me
O
N
9
1.00
1.01
5.04
0.99
1.07
1.01
0.95
3.10
3.08
3.12
3.14
10.0
9.5
9.0
8.5
8.0
7.5
7.0
6.5
6.0
5.5
5.0
4.5
4.0
3.5
3.0
2.5
2.0
1.5
1.0
0.5
0.0
−0.5
ppm

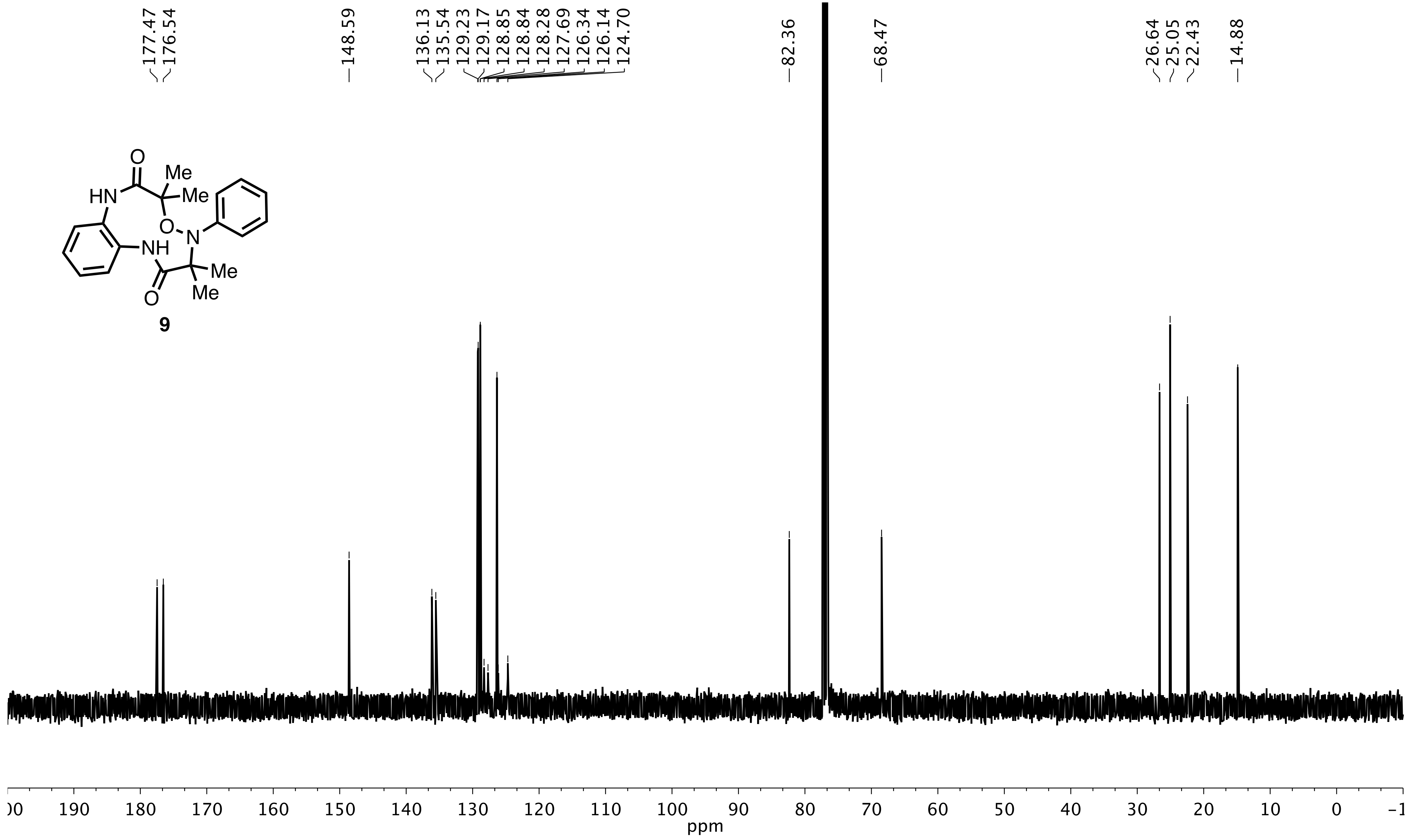

177.47
176.54
148.59
136.13
135.54
129.23
129.17
128.85
128.84
128.28
127.69
126.34
126.14
124.70
82.36
68.47
26.64
25.05
22.43
14.88
HN
NH
O
O
Me
Me
Me
Me
N
9
00
190
180
170
160
150
140
130
120
110
100
90
80
70
60
50
40
30
20
10
0
–1
ppm

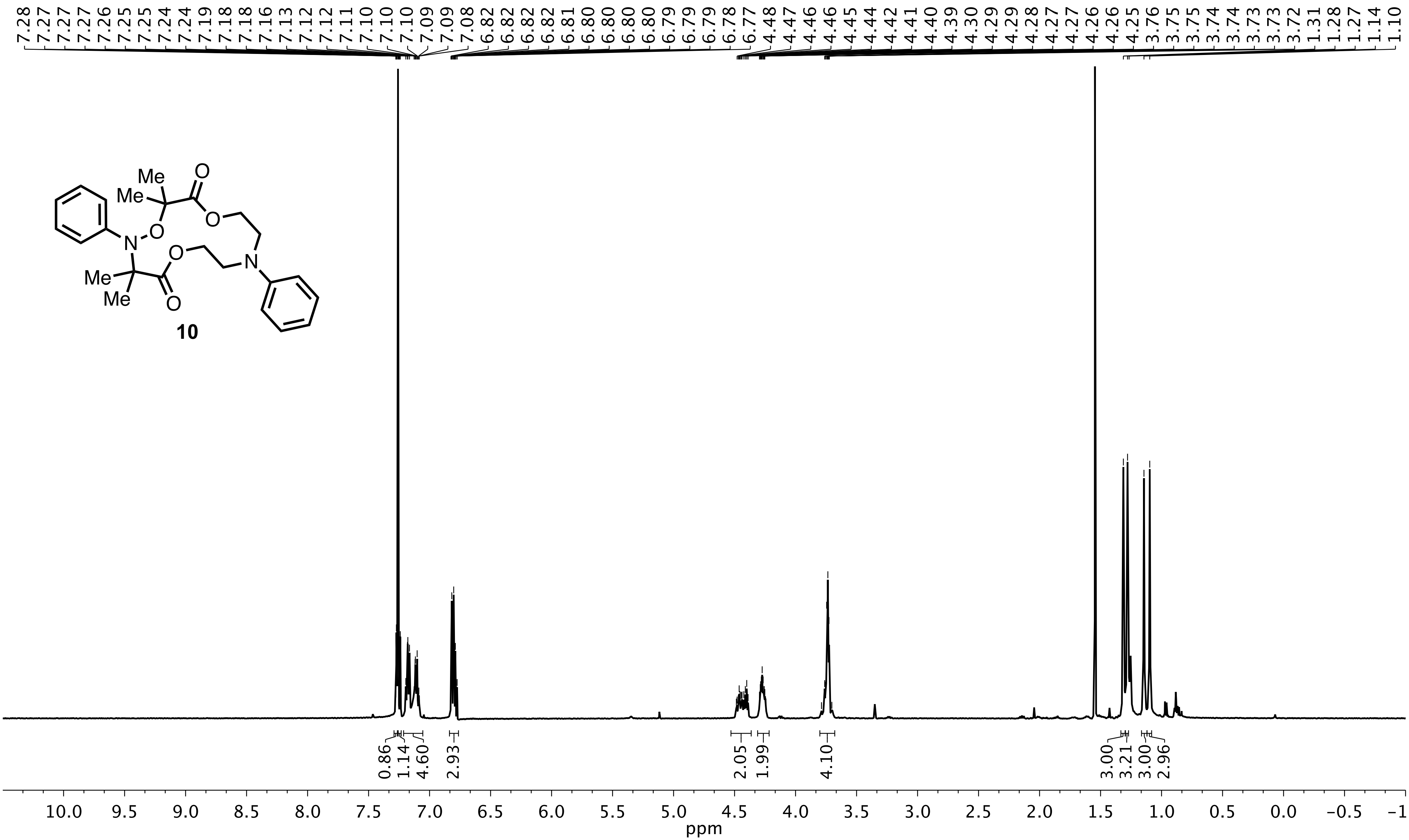

7.28 7.27 7.27 7.27 7.26 7.25 7.25 7.24 7.24 7.19 7.18 7.18 7.16 7.13 7.12 7.12 7.11 7.10 7.10 7.10 7.09 7.09 7.08 6.82 6.82 6.82 6.82 6.81 6.80 6.80 6.80 6.80 6.79 6.79 6.79 6.78 6.77 4.48 4.47 4.46 4.46 4.45 4.44 4.42 4.41 4.40 4.39 4.30 4.29 4.29 4.28 4.27 4.27 4.26 4.26 4.25 3.76 3.75 3.75 3.74 3.74 3.73 3.73 3.72 1.31 1.28 1.27 1.14 1.10
Me
Me
O
O
N
O
O
N
Me
Me
O
10
0.86
1.14
4.60
2.93
2.05
1.99
4.10
3.00
3.21
3.00
2.96
10.0 9.5 9.0 8.5 8.0 7.5 7.0 6.5 6.0 5.5 5.0 4.5 4.0 3.5 3.0 2.5 2.0 1.5 1.0 0.5 0.0 −0.5 −1
ppm

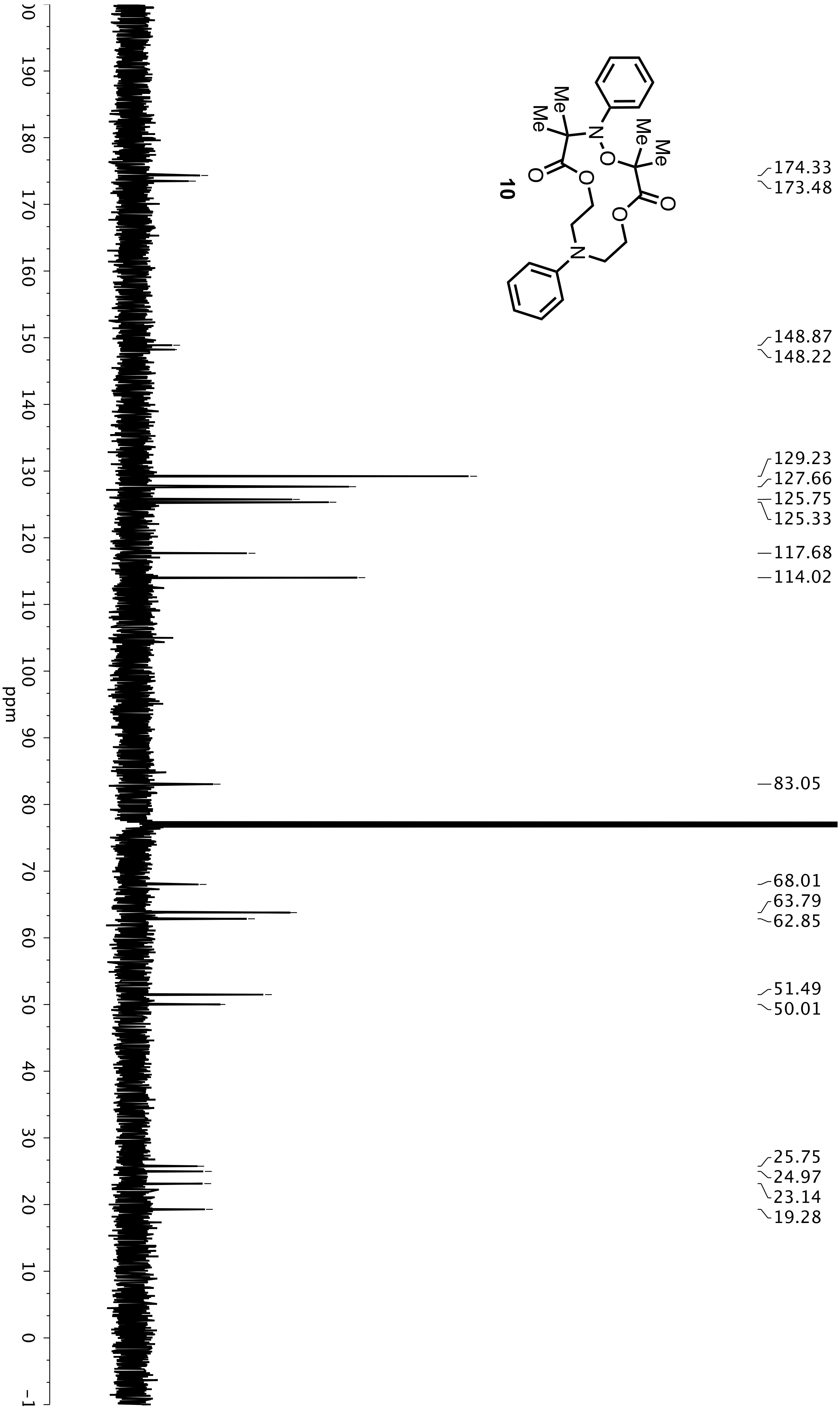
10
Me
Me
Me
Me
N
O
O
O
O
O
N
174.33
173.48
148.87
148.22
129.23
127.66
125.75
125.33
117.68
114.02
83.05
68.01
63.79
62.85
51.49
50.01
25.75
24.97
23.14
19.28
ppm

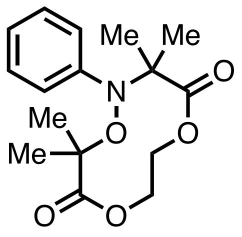

11

NMR in $CDCl_3$ at 50 °C

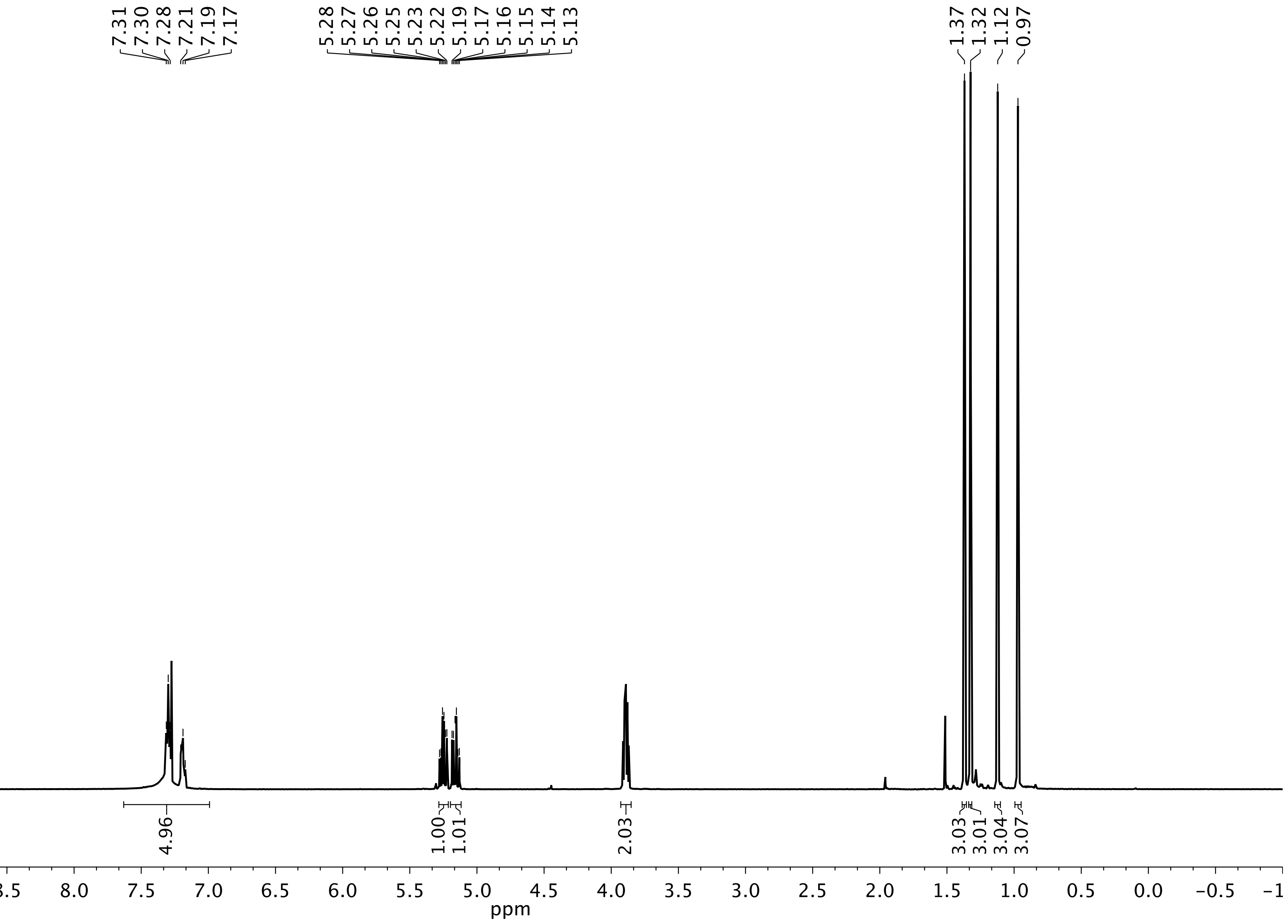

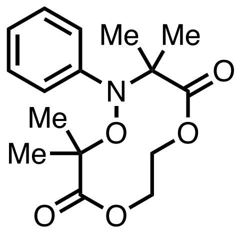

11

NMR in CDCl$_3$ at 50 °C

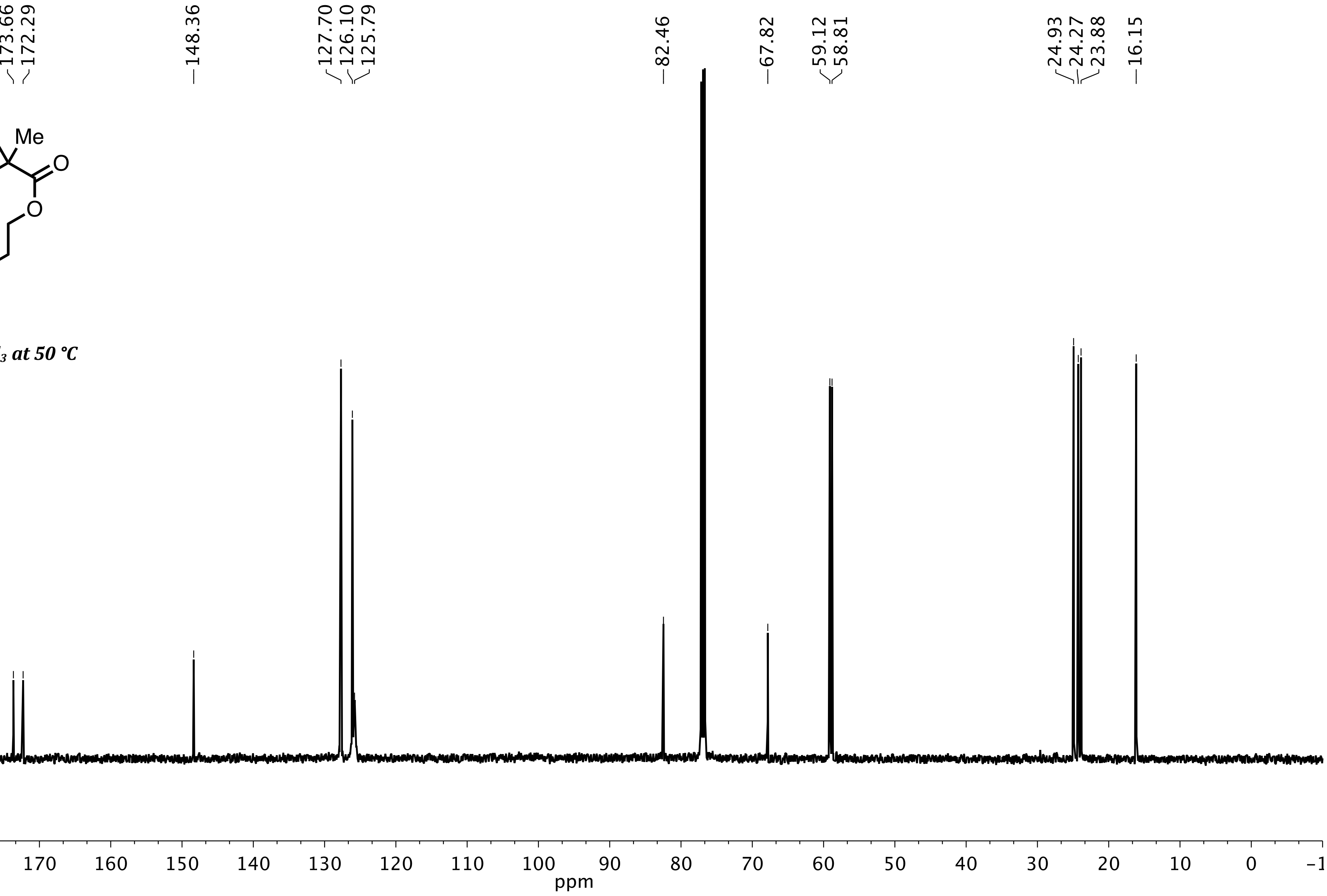

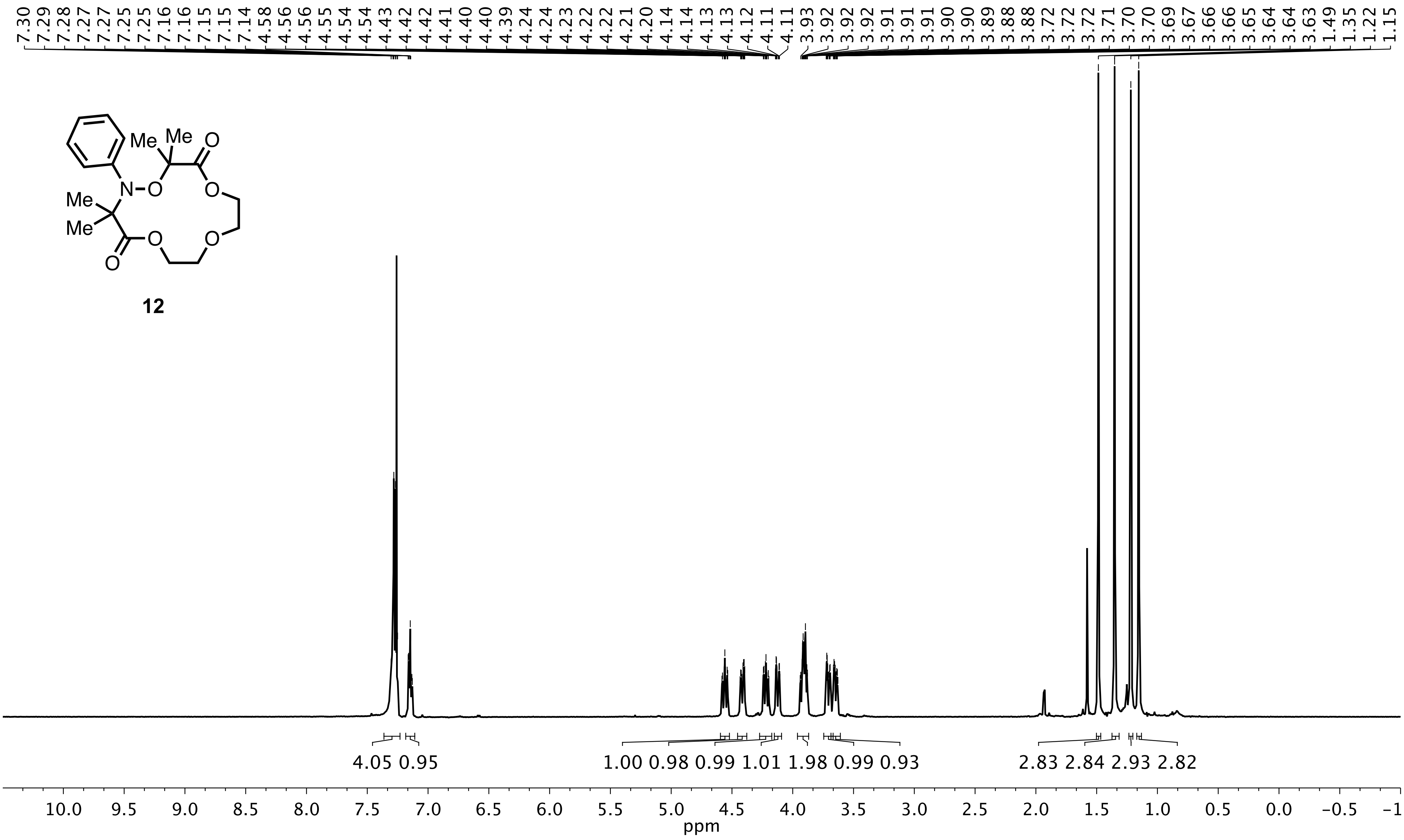
7.30 7.29 7.28 7.27 7.27 7.25 7.25 7.16 7.16 7.15 7.15 7.14 4.58 4.56 4.56 4.55 4.54 4.54 4.43 4.42 4.42 4.41 4.40 4.40 4.39 4.24 4.24 4.23 4.22 4.22 4.21 4.20 4.14 4.14 4.13 4.13 4.12 4.11 4.11 3.93 3.92 3.92 3.92 3.91 3.91 3.91 3.90 3.90 3.89 3.88 3.88 3.72 3.72 3.72 3.71 3.70 3.70 3.69 3.67 3.66 3.66 3.65 3.64 3.64 3.63 1.49 1.35 1.22 1.15
Me
Me
N
O
O
O
O
O
O
Me
Me
12
4.05 0.95
1.00 0.98 0.99 1.01 1.98 0.99 0.93
2.83 2.84 2.93 2.82
10.0 9.5 9.0 8.5 8.0 7.5 7.0 6.5 6.0 5.5 5.0 4.5 4.0 3.5 3.0 2.5 2.0 1.5 1.0 0.5 0.0 −0.5 −1
ppm

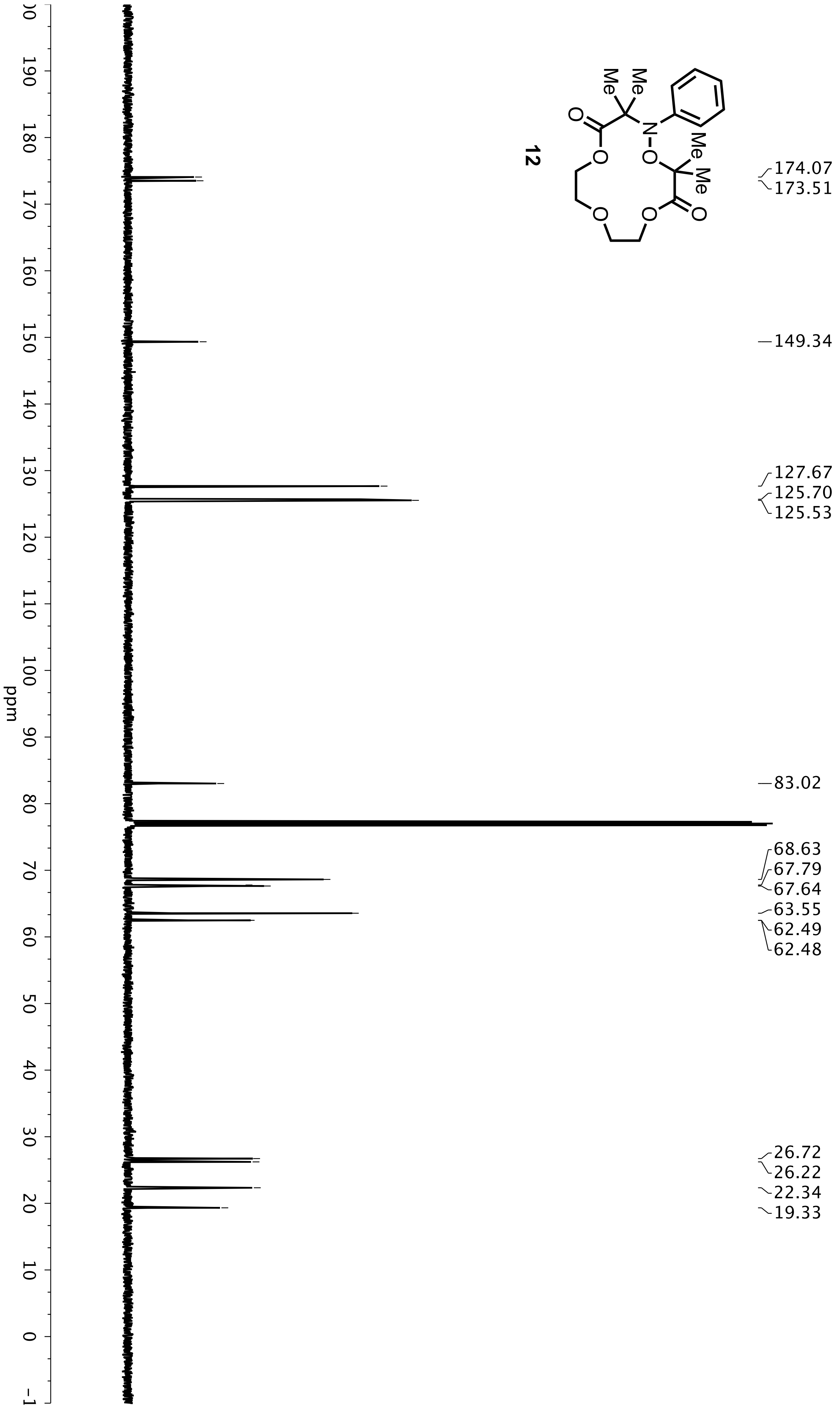
12
Me
Me
Me
Me
N
O
O
O
O
O
O
O
174.07
173.51
149.34
127.67
125.70
125.53
83.02
68.63
67.79
67.64
63.55
62.49
62.48
26.72
26.22
22.34
19.33
ppm

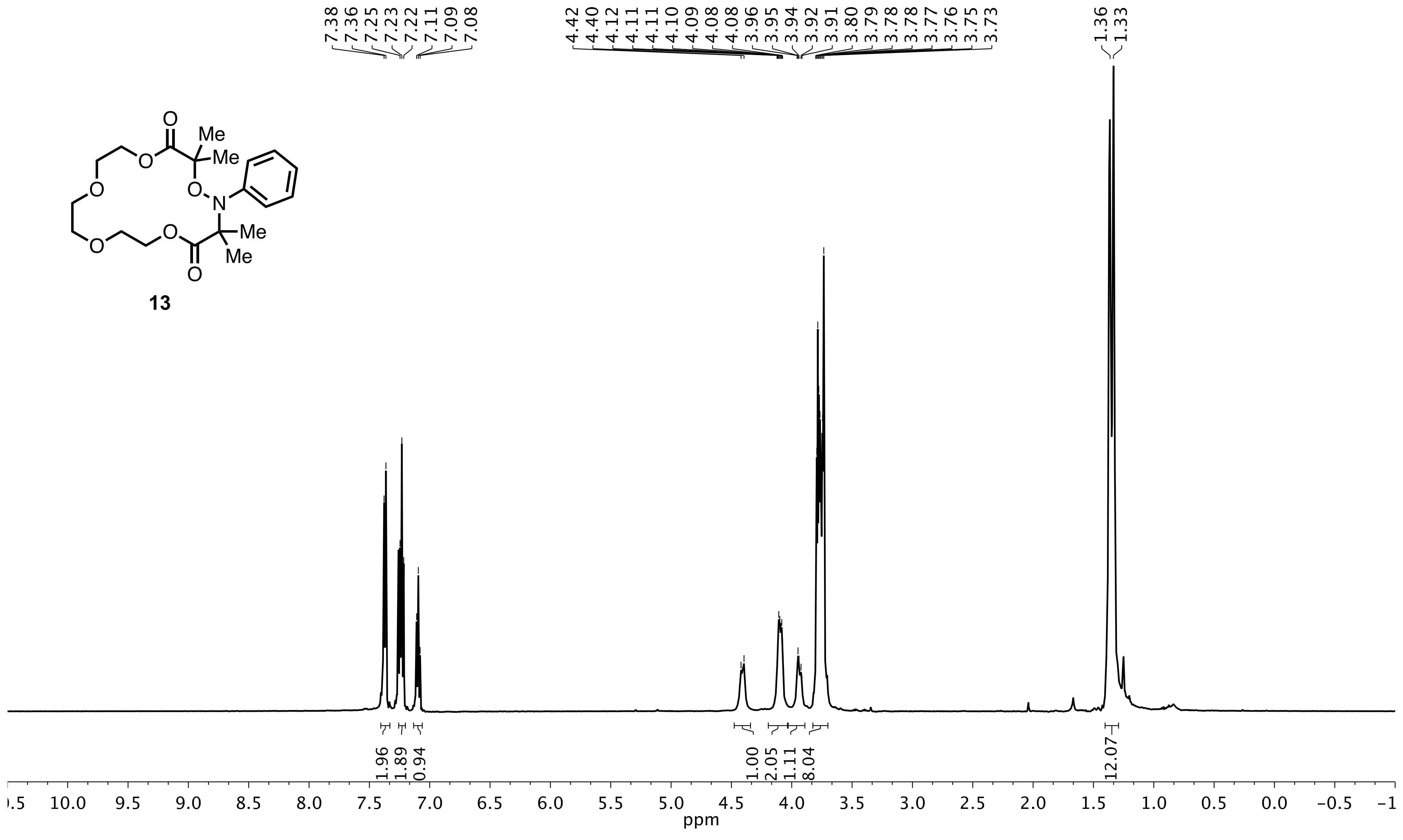
13
Me
Me
N
O
ppm
7.38 7.36 7.25 7.23 7.22 7.11 7.09 7.08
4.42 4.40 4.12 4.11 4.11 4.10 4.09 4.08 4.08 3.96 3.95 3.94 3.92 3.91 3.80 3.79 3.78 3.78 3.77 3.76 3.75 3.73
1.36 1.33
1.96 1.89 0.94 1.00 2.05 1.11 8.04 12.07

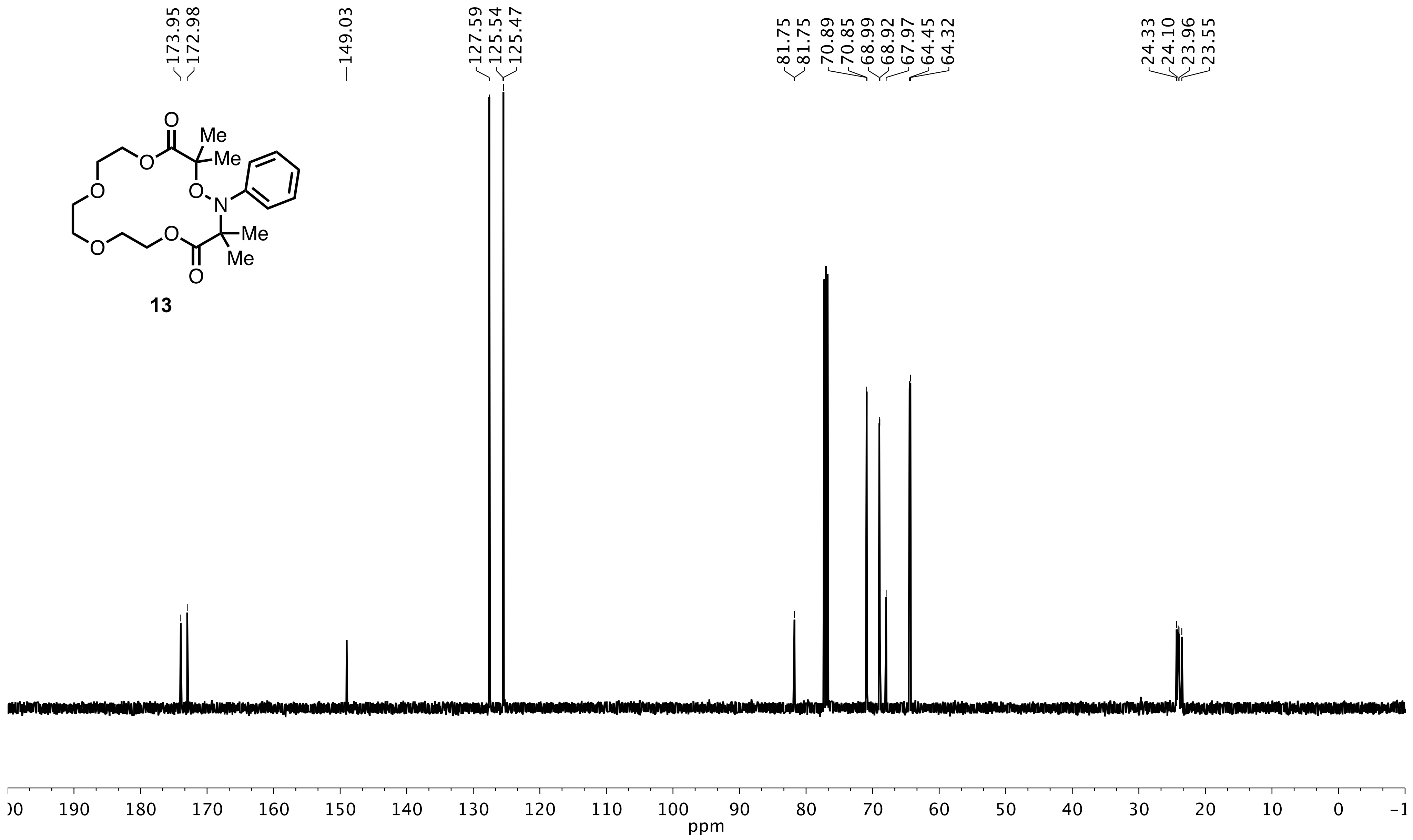
173.95
172.98
149.03
127.59
125.54
125.47
81.75
81.75
70.89
70.85
68.99
68.92
67.97
64.45
64.32
24.33
24.10
23.96
23.55
Me
Me
O
N
Me
Me
O
O
O
O
O
O
190
180
170
160
150
140
130
120
110
100
90
80
70
60
50
40
30
20
10
0
ppm

13

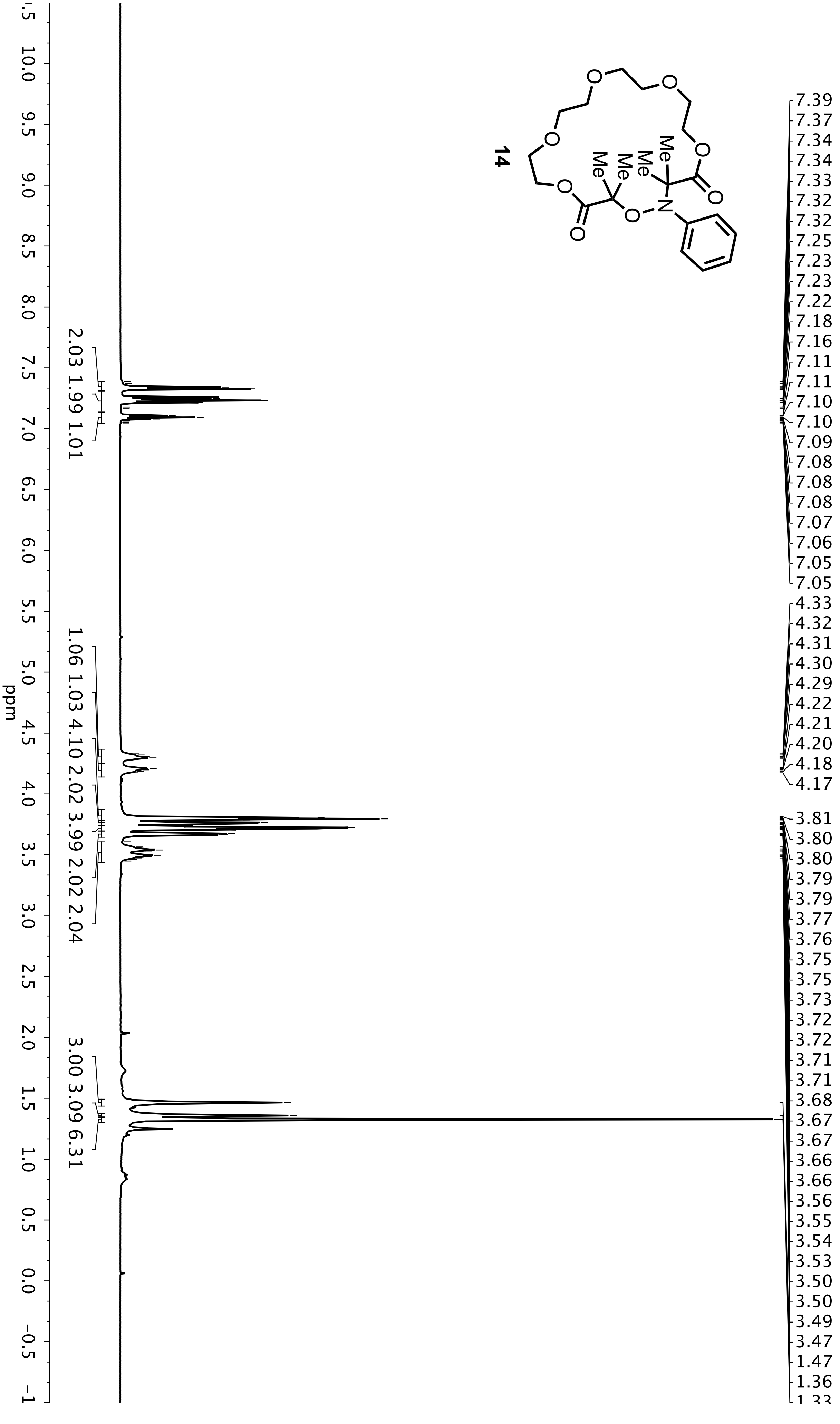
14
Me
Me
Me
Me
N
O
7.39
7.37
7.34
7.34
7.33
7.32
7.32
7.25
7.23
7.23
7.22
7.18
7.16
7.11
7.11
7.10
7.10
7.09
7.08
7.08
7.08
7.07
7.06
7.05
7.05
4.33
4.32
4.31
4.30
4.29
4.22
4.21
4.20
4.18
4.17
3.81
3.80
3.80
3.79
3.79
3.77
3.76
3.75
3.75
3.73
3.72
3.72
3.71
3.71
3.68
3.67
3.67
3.66
3.66
3.56
3.55
3.54
3.53
3.50
3.50
3.49
3.47
1.47
1.36
2.03 1.99 1.01
1.06 1.03 4.10 2.02 3.99 2.02 2.04
3.00 3.09 6.31
ppm

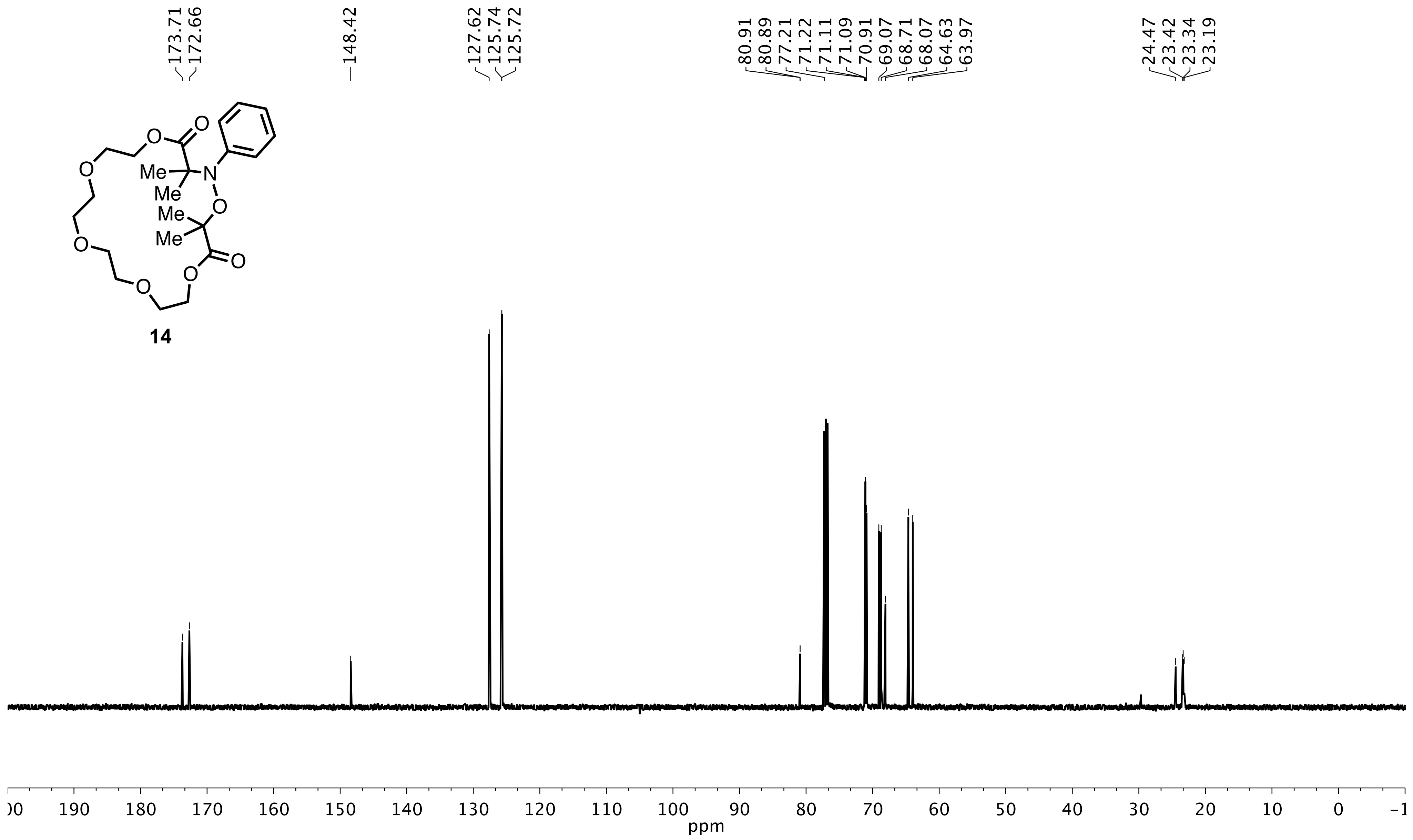
173.71
172.66
148.42
127.62
125.74
125.72
80.91
80.89
77.21
71.22
71.11
71.09
70.91
69.07
68.71
68.07
64.63
63.97
24.47
23.42
23.34
23.19
Me
Me
Me
Me
N
O
14
ppm

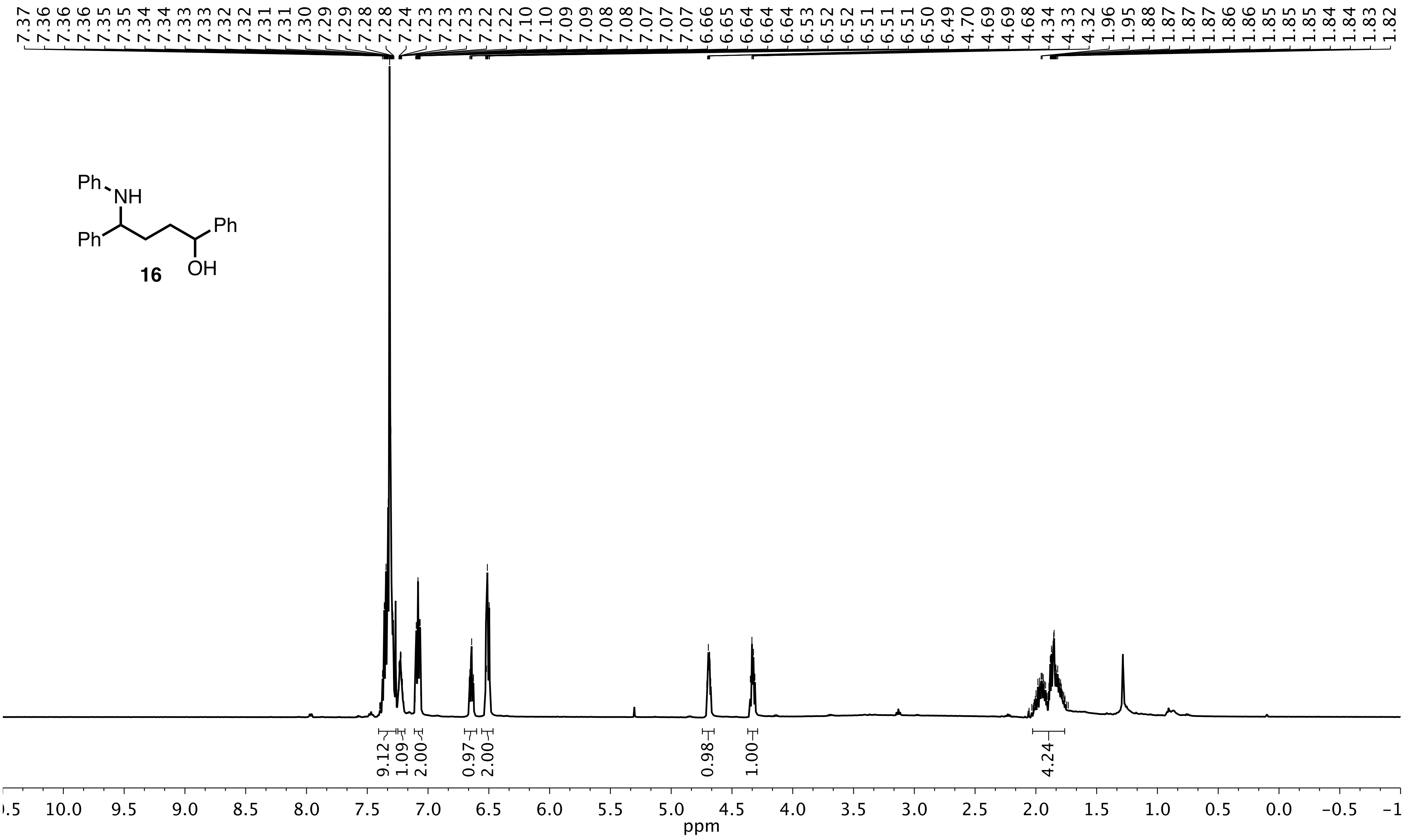
7.37 7.36 7.36 7.36 7.35 7.35 7.34 7.34 7.33 7.33 7.32 7.32 7.31 7.31 7.30 7.29 7.29 7.28 7.28 7.24 7.23 7.23 7.23 7.22 7.22 7.10 7.10 7.09 7.09 7.08 7.08 7.07 7.07 7.07 6.66 6.65 6.64 6.64 6.64 6.53 6.52 6.52 6.51 6.51 6.51 6.50 6.49 4.70 4.69 4.69 4.68 4.34 4.33 4.32 1.96 1.95 1.88 1.87 1.87 1.87 1.86 1.86 1.85 1.85 1.85 1.84 1.84 1.83 1.82
Ph
NH
Ph
Ph
OH
16
9.12
1.09
2.00
0.97
2.00
0.98
1.00
4.24
10.0 9.5 9.0 8.5 8.0 7.5 7.0 6.5 6.0 5.5 5.0 4.5 4.0 3.5 3.0 2.5 2.0 1.5 1.0 0.5 0.0 −0.5 −1
ppm

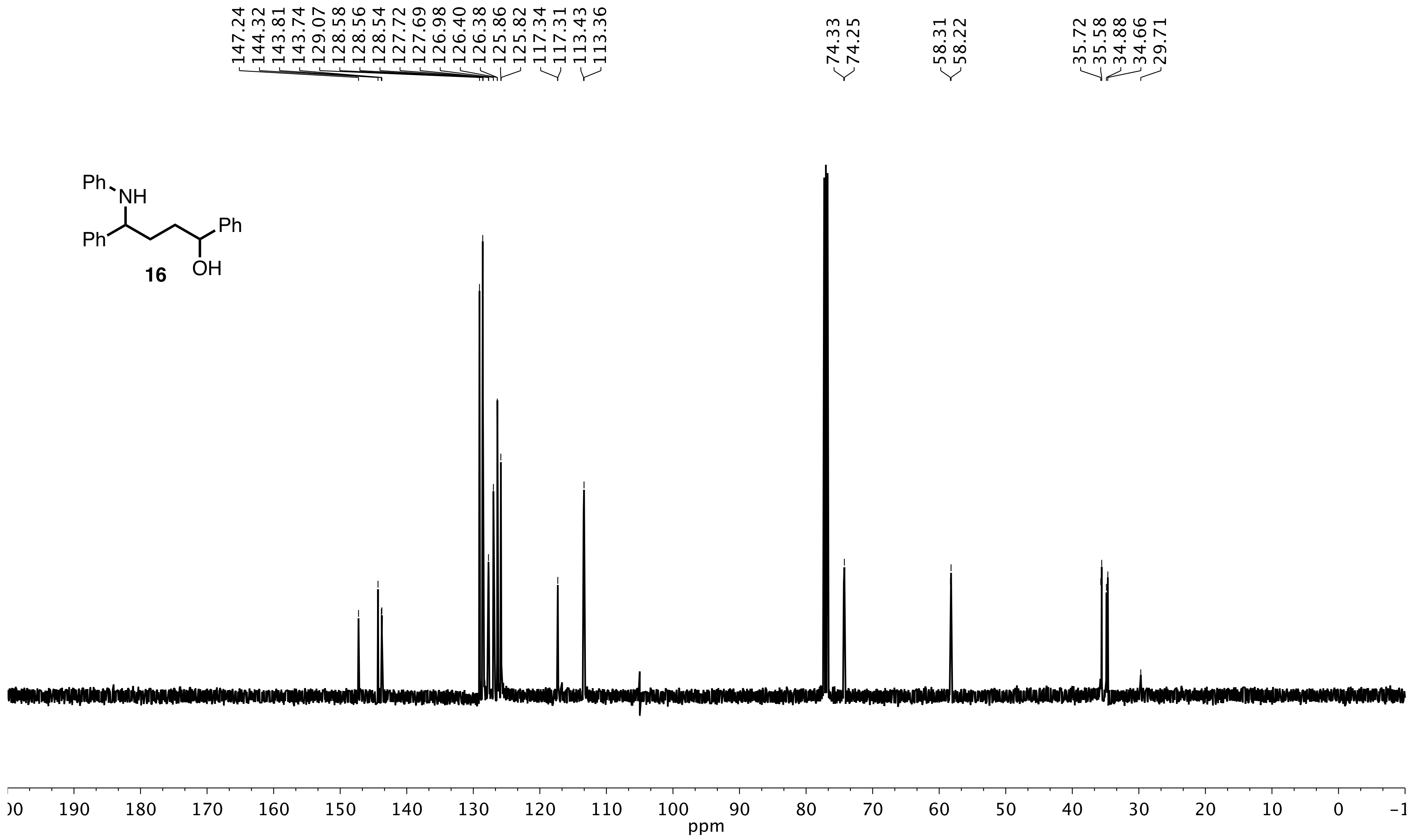

147.24
144.32
143.81
143.74
129.07
128.58
128.56
128.54
127.72
127.69
126.98
126.40
126.38
125.86
125.82
117.34
117.31
113.43
113.36
74.33
74.25
58.31
58.22
35.72
35.58
34.88
34.66
29.71
Ph
NH
Ph
Ph
OH
16
190
180
170
160
150
140
130
120
110
100
90
80
70
60
50
40
30
20
10
0
ppm

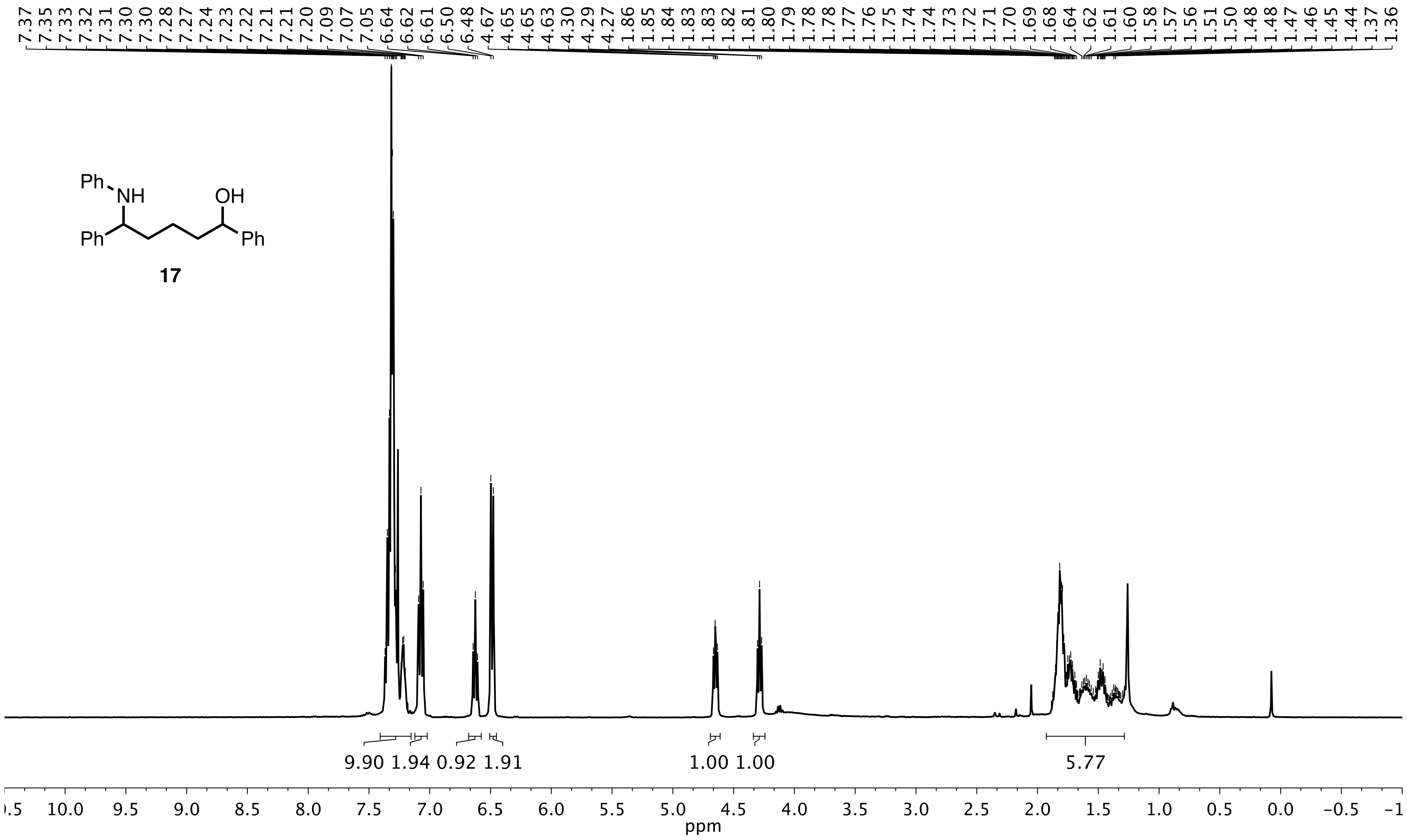
7.37 7.35 7.33 7.32 7.31 7.30 7.30 7.28 7.27 7.24 7.23 7.22 7.21 7.21 7.20 7.09 7.07 7.05 6.64 6.62 6.61 6.50 6.48 4.67 4.65 4.65 4.63 4.30 4.29 4.27 1.86 1.85 1.84 1.83 1.83 1.82 1.81 1.80 1.79 1.78 1.78 1.77 1.76 1.75 1.74 1.74 1.73 1.72 1.71 1.70 1.69 1.68 1.64 1.62 1.61 1.60 1.58 1.57 1.56 1.51 1.50 1.48 1.48 1.47 1.46 1.45 1.44 1.37 1.36
Ph
NH
OH
Ph
Ph
17
9.90 1.94 0.92 1.91
1.00 1.00
5.77
10.0 9.5 9.0 8.5 8.0 7.5 7.0 6.5 6.0 5.5 5.0 4.5 4.0 3.5 3.0 2.5 2.0 1.5 1.0 0.5 0.0 −0.5
ppm

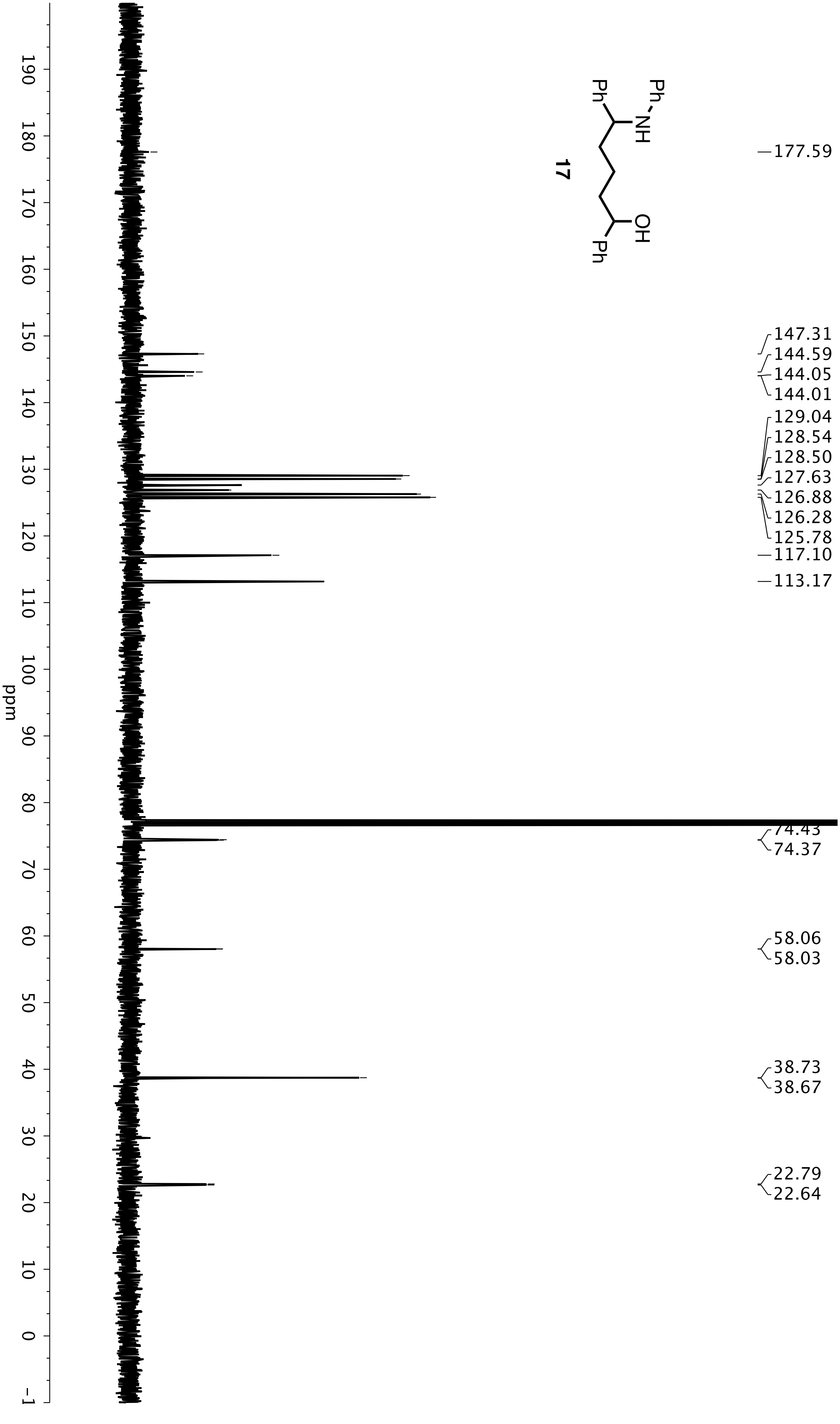

Ph
NH
OH
Ph
Ph
17
177.59
147.31
144.59
144.05
144.01
129.04
128.54
128.50
127.63
126.88
126.28
125.78
117.10
113.17
74.43
74.37
58.06
58.03
38.73
38.67
22.79
22.64
ppm

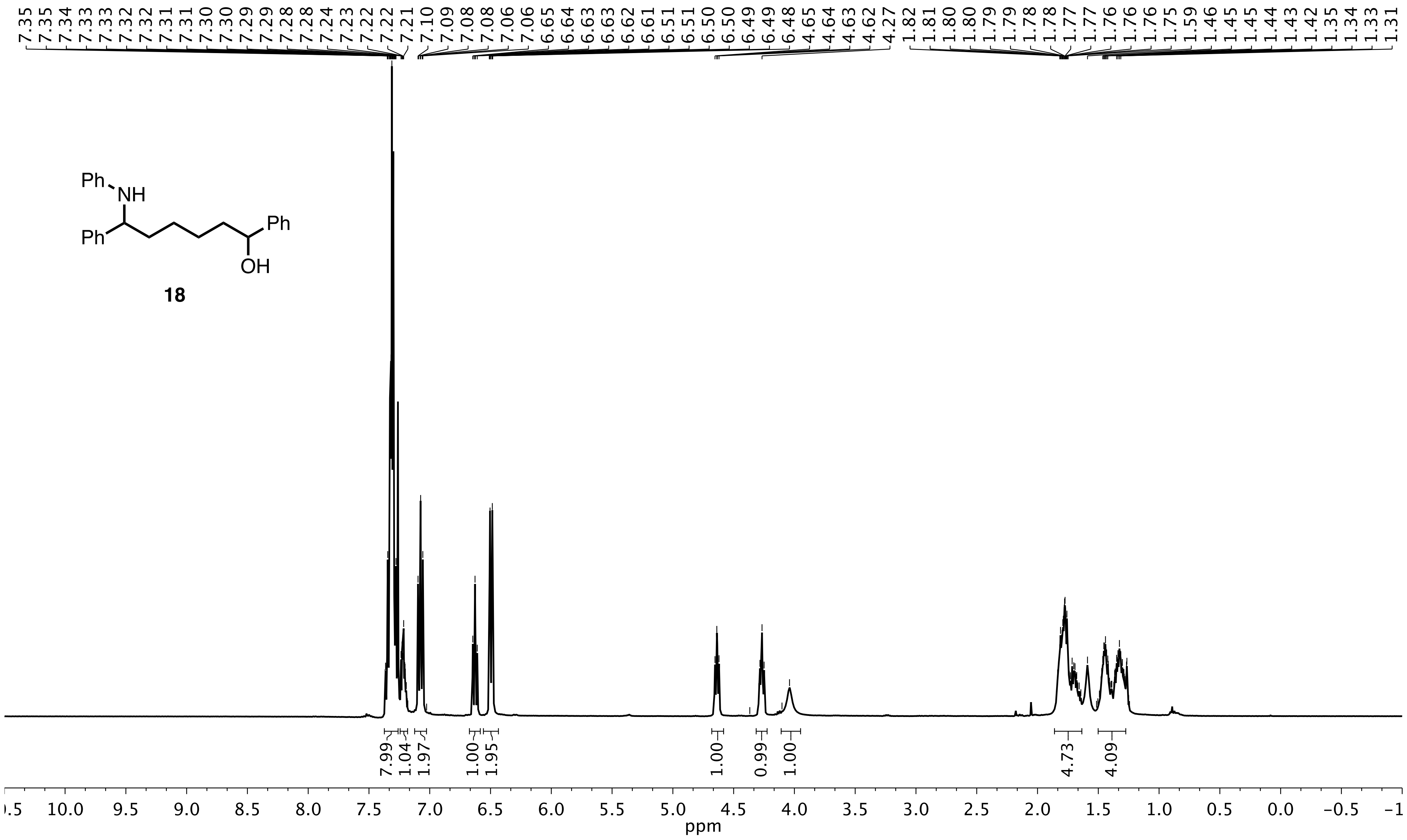

Ph
NH
Ph
Ph
OH
18
7.35 7.35 7.34 7.33 7.33 7.32 7.32 7.31 7.31 7.30 7.30 7.29 7.29 7.28 7.28 7.24 7.23 7.22 7.22 7.21 7.10 7.09 7.08 7.08 7.06 7.06 6.65 6.64 6.63 6.63 6.62 6.61 6.51 6.51 6.50 6.50 6.49 6.49 6.48 4.65 4.64 4.63 4.62 4.27
1.82 1.81 1.80 1.80 1.79 1.79 1.78 1.78 1.77 1.77 1.76 1.76 1.76 1.75 1.59 1.46 1.45 1.45 1.44 1.43 1.42 1.35 1.34 1.33 1.31
7.99 1.04 1.97 1.00 1.95 1.00 0.99 1.00 4.73 4.09
10.0 9.5 9.0 8.5 8.0 7.5 7.0 6.5 6.0 5.5 5.0 4.5 4.0 3.5 3.0 2.5 2.0 1.5 1.0 0.5 0.0 −0.5
ppm

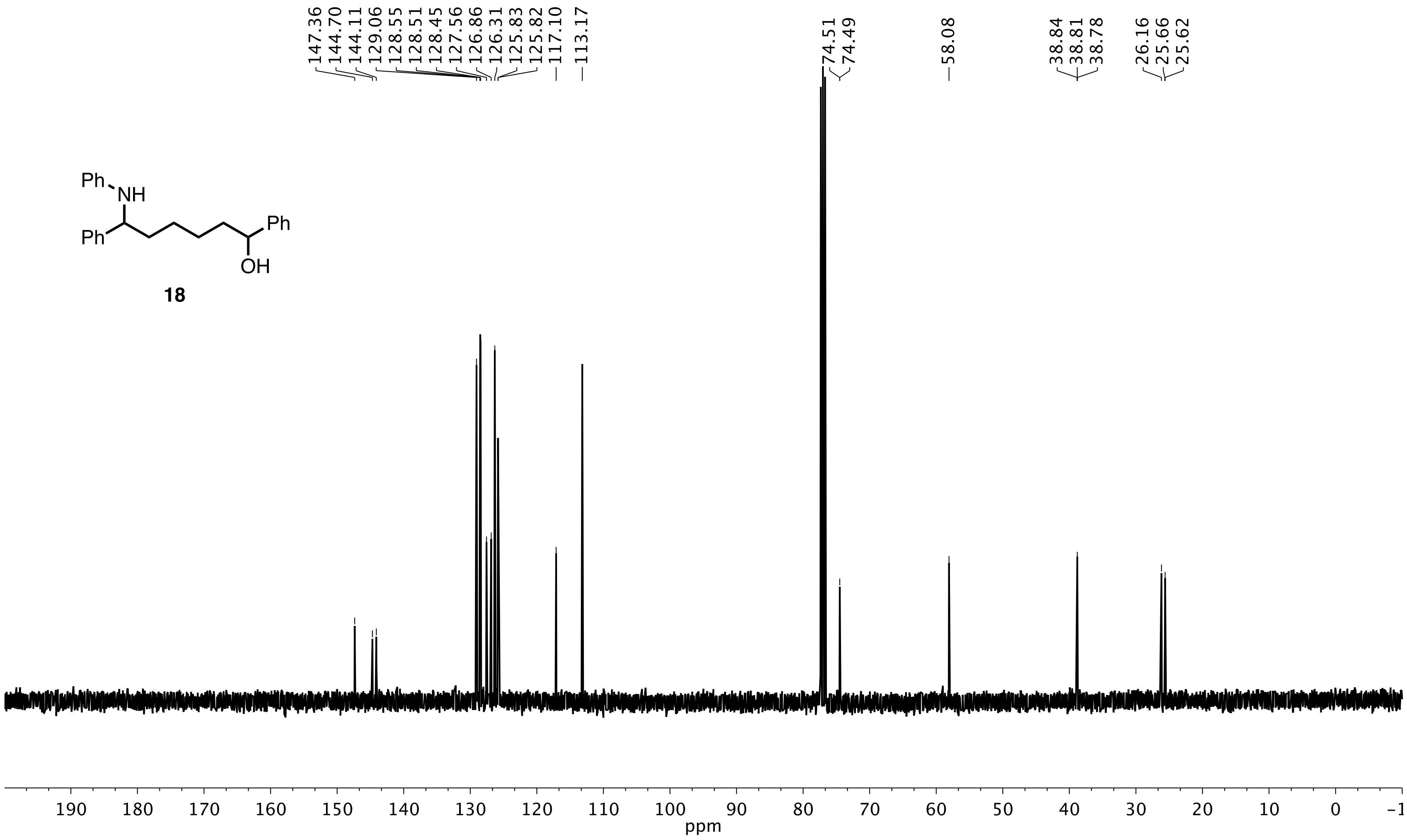

Ph
NH
Ph
Ph
OH
147.36
144.70
144.11
129.06
128.55
128.51
128.45
127.56
126.86
126.31
125.83
125.82
117.10
113.17
74.51
74.49
58.08
38.84
38.81
38.78
26.16
25.66
25.62
190
180
170
160
150
140
130
120
110
100
90
80
70
60
50
40
30
20
10
0
–1
ppm

18

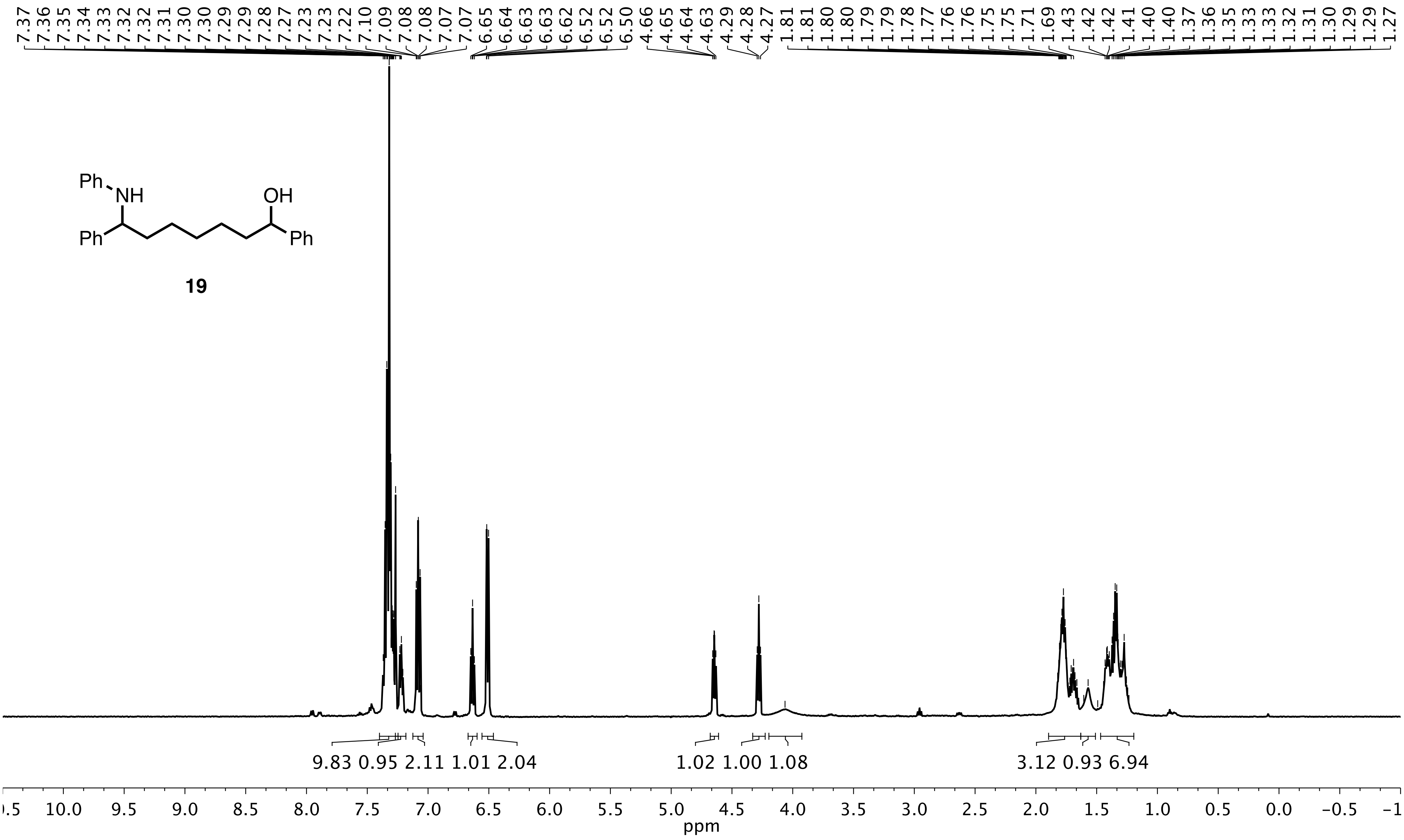
7.37 7.36 7.35 7.34 7.33 7.32 7.32 7.31 7.30 7.30 7.29 7.29 7.28 7.27 7.23 7.23 7.22 7.10 7.09 7.08 7.08 7.07 7.07 6.65 6.64 6.63 6.63 6.62 6.52 6.52 6.50 4.66 4.65 4.64 4.63 4.29 4.28 4.27 1.81 1.81 1.80 1.80 1.79 1.79 1.78 1.77 1.76 1.76 1.75 1.75 1.71 1.69 1.43 1.42 1.42 1.41 1.40 1.40 1.37 1.36 1.35 1.33 1.33 1.32 1.31 1.30 1.29 1.29 1.27
Ph
NH
OH
Ph
Ph
19
9.83 0.95 2.11 1.01 2.04
1.02 1.00 1.08
3.12 0.93 6.94
10.0 9.5 9.0 8.5 8.0 7.5 7.0 6.5 6.0 5.5 5.0 4.5 4.0 3.5 3.0 2.5 2.0 1.5 1.0 0.5 0.0 −0.5
ppm

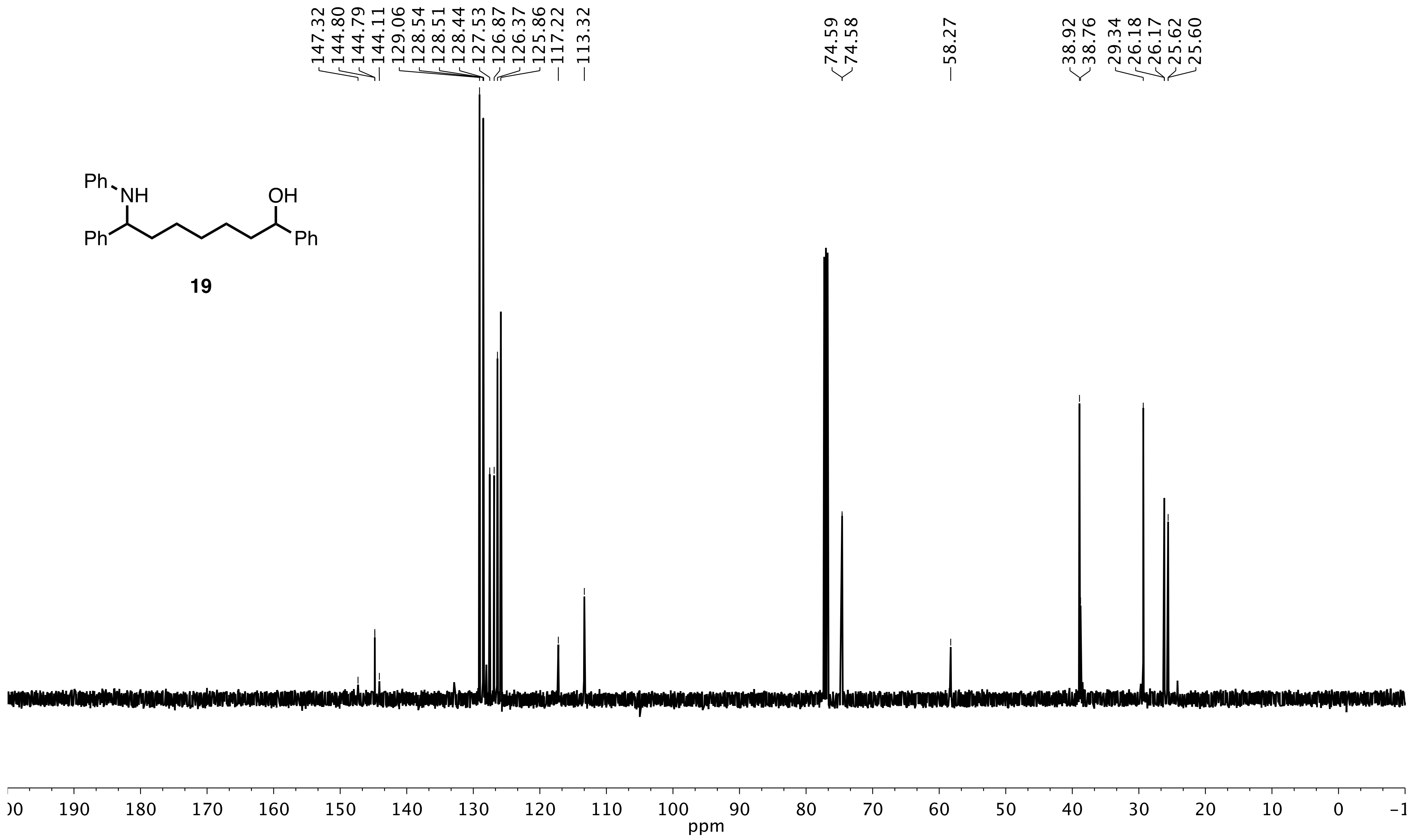
147.32
144.80
144.79
144.11
129.06
128.54
128.51
128.44
127.53
126.87
126.37
125.86
117.22
113.32
74.59
74.58
58.27
38.92
38.76
29.34
26.18
26.17
25.62
25.60
Ph
NH
OH
Ph
Ph
19
ppm

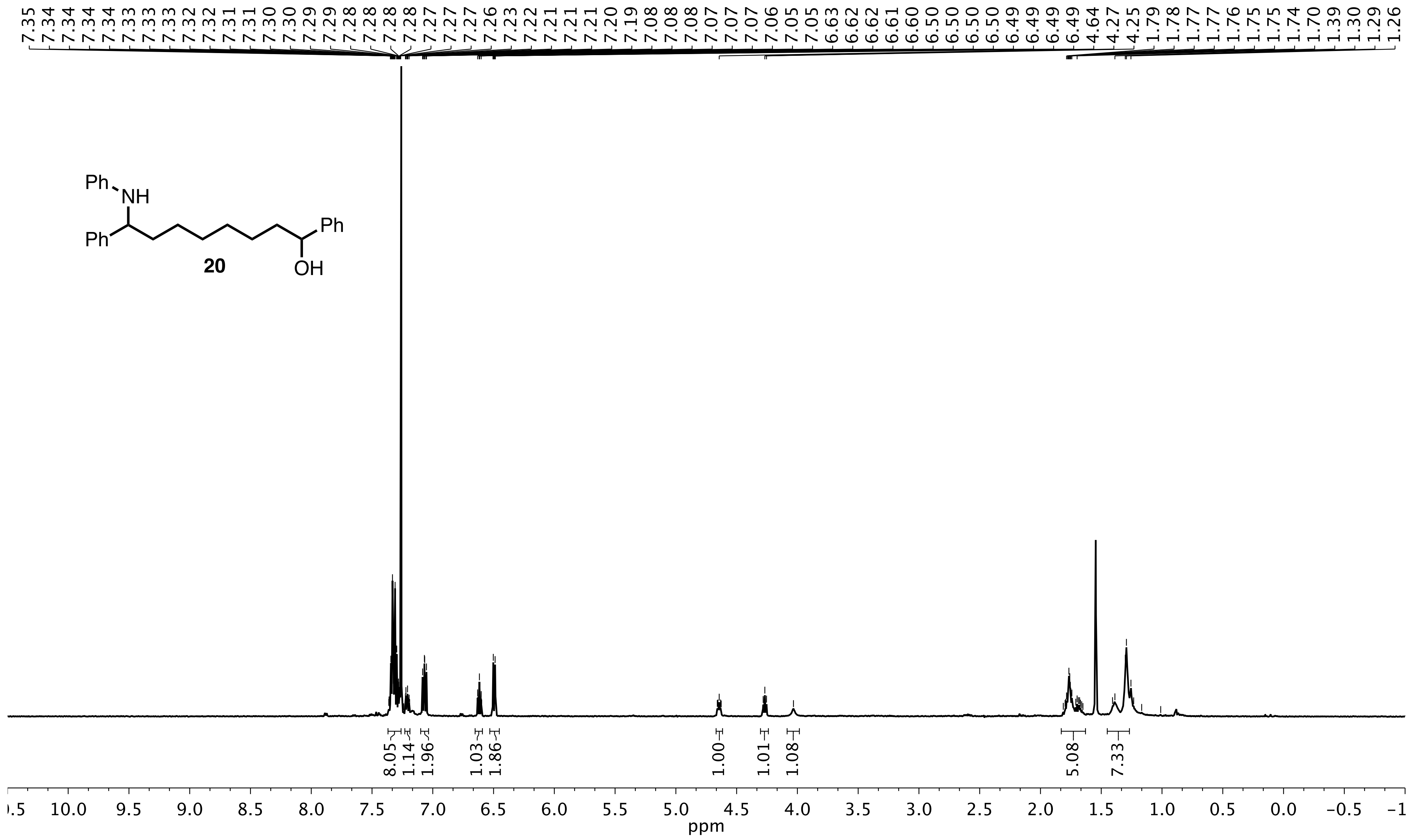

7.35 7.34 7.34 7.34 7.34 7.33 7.33 7.33 7.32 7.32 7.31 7.31 7.30 7.30 7.29 7.29 7.28 7.28 7.28 7.28 7.27 7.27 7.27 7.26 7.23 7.22 7.21 7.21 7.21 7.20 7.19 7.08 7.08 7.08 7.07 7.07 7.07 7.06 7.05 7.05 6.63 6.62 6.62 6.61 6.60 6.50 6.50 6.50 6.50 6.49 6.49 6.49 6.49 4.64 4.27 4.25 1.79 1.78 1.77 1.77 1.76 1.75 1.75 1.74 1.70 1.39 1.30 1.29 1.26
Ph
NH
Ph
Ph
OH
20
8.05
1.14
1.96
1.03
1.86
1.00
1.01
1.08
5.08
7.33
ppm

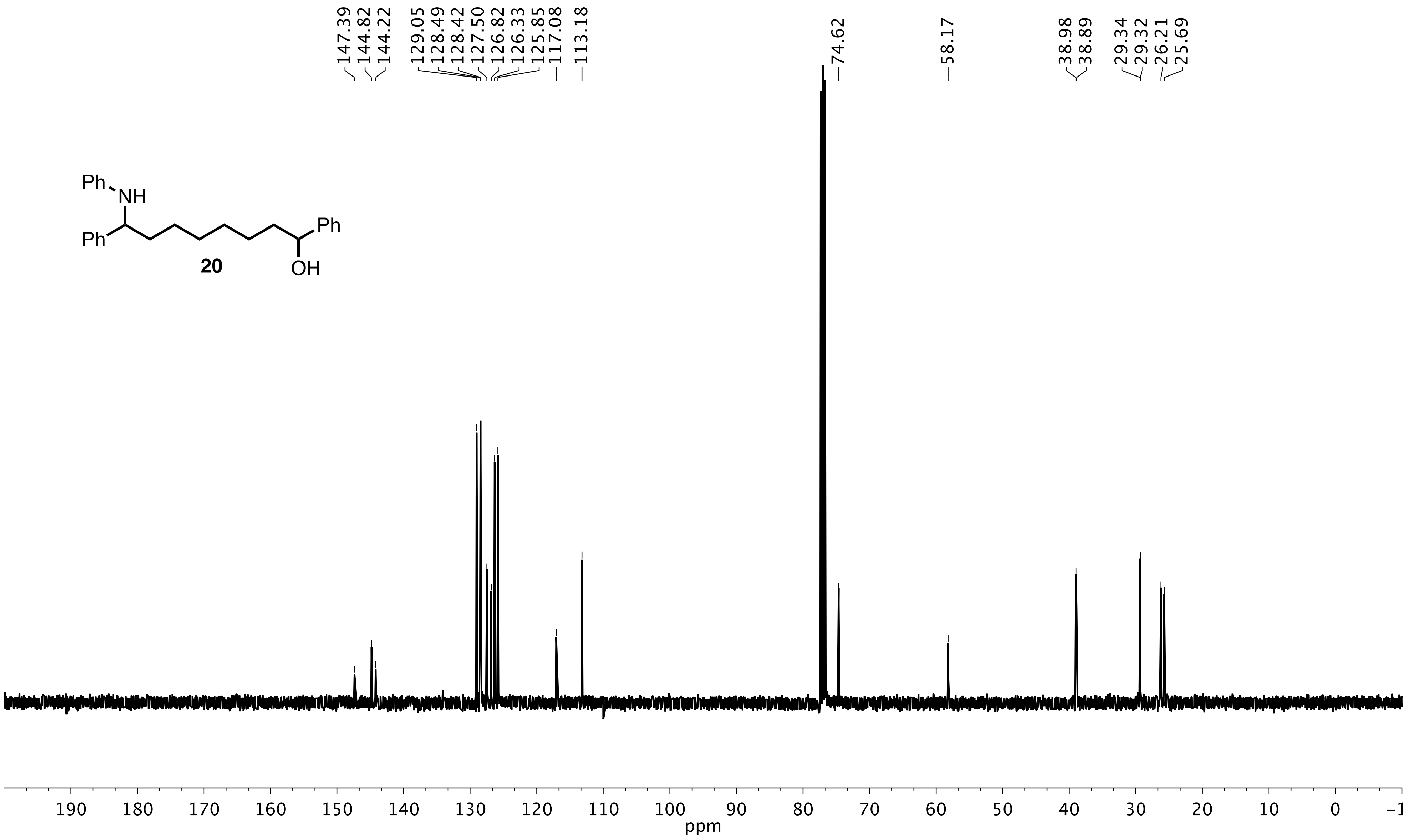
Ph
NH
Ph
Ph
OH
20
147.39
144.82
144.22
129.05
128.49
128.42
127.50
126.82
126.33
125.85
117.08
113.18
74.62
58.17
38.98
38.89
29.34
29.32
26.21
25.69
190
180
170
160
150
140
130
120
110
100
90
80
70
60
50
40
30
20
10
0
–1
ppm

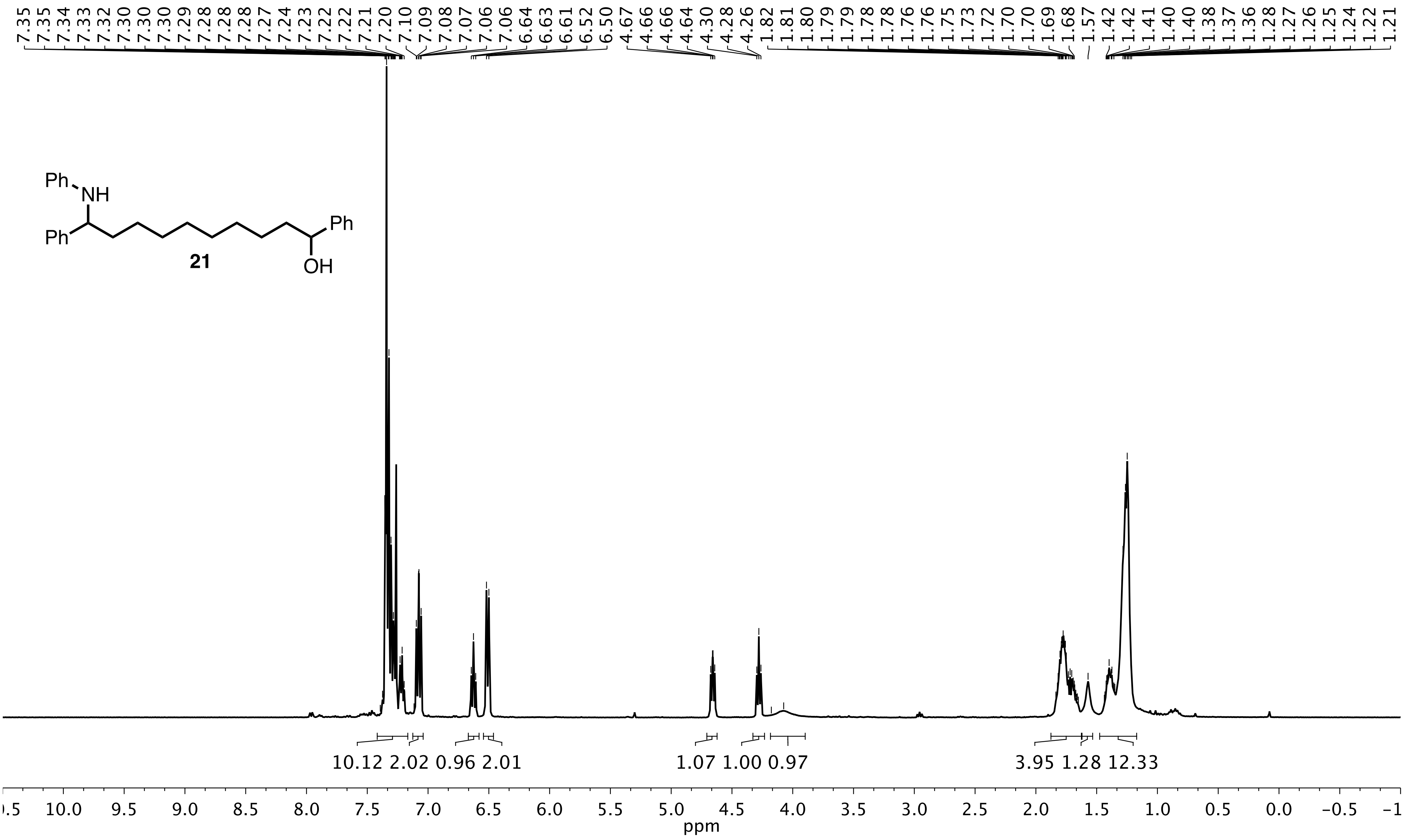
7.35
7.35
7.34
7.33
7.32
7.30
7.30
7.30
7.29
7.28
7.28
7.28
7.27
7.24
7.23
7.22
7.22
7.21
7.20
7.10
7.09
7.08
7.07
7.06
7.06
6.64
6.63
6.61
6.52
6.50
4.67
4.66
4.66
4.64
4.30
4.28
4.26
1.82
1.81
1.80
1.79
1.79
1.78
1.78
1.76
1.76
1.75
1.73
1.72
1.70
1.70
1.69
1.68
1.57
1.42
1.42
1.41
1.40
1.40
1.38
1.37
1.36
1.28
1.27
1.26
1.25
1.24
1.22
1.21
Ph
NH
Ph
Ph
OH
21
10.12 2.02 0.96 2.01
1.07 1.00 0.97
3.95 1.28 12.33
10.0 9.5 9.0 8.5 8.0 7.5 7.0 6.5 6.0 5.5 5.0 4.5 4.0 3.5 3.0 2.5 2.0 1.5 1.0 0.5 0.0 −0.5
ppm

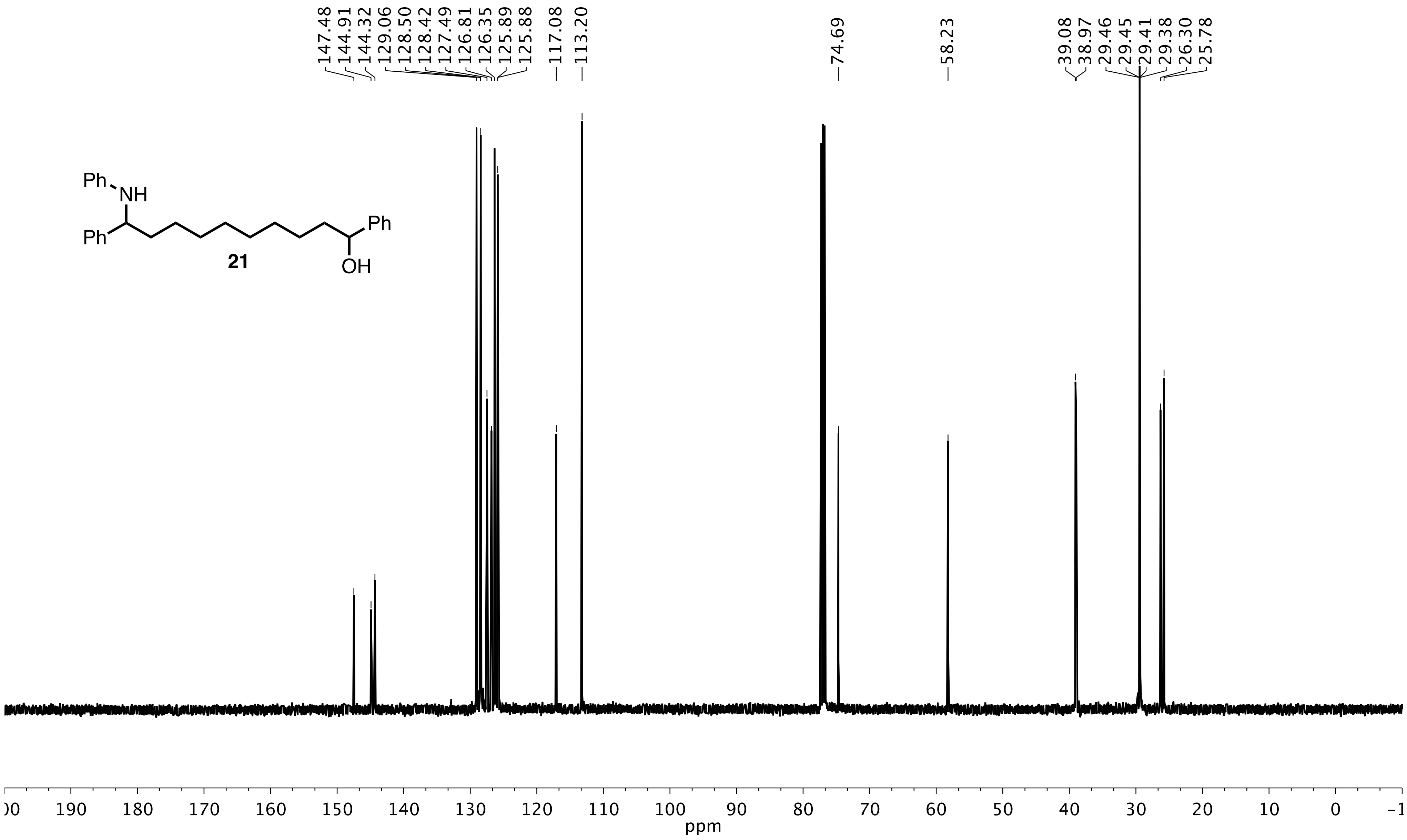
147.48
144.91
144.32
129.06
128.50
128.42
127.49
126.81
126.35
125.89
125.88
117.08
113.20
74.69
58.23
39.08
38.97
29.46
29.45
29.41
29.38
26.30
25.78
Ph
NH
Ph
21
Ph
OH
190
180
170
160
150
140
130
120
110
100
90
80
70
60
50
40
30
20
10
0
ppm

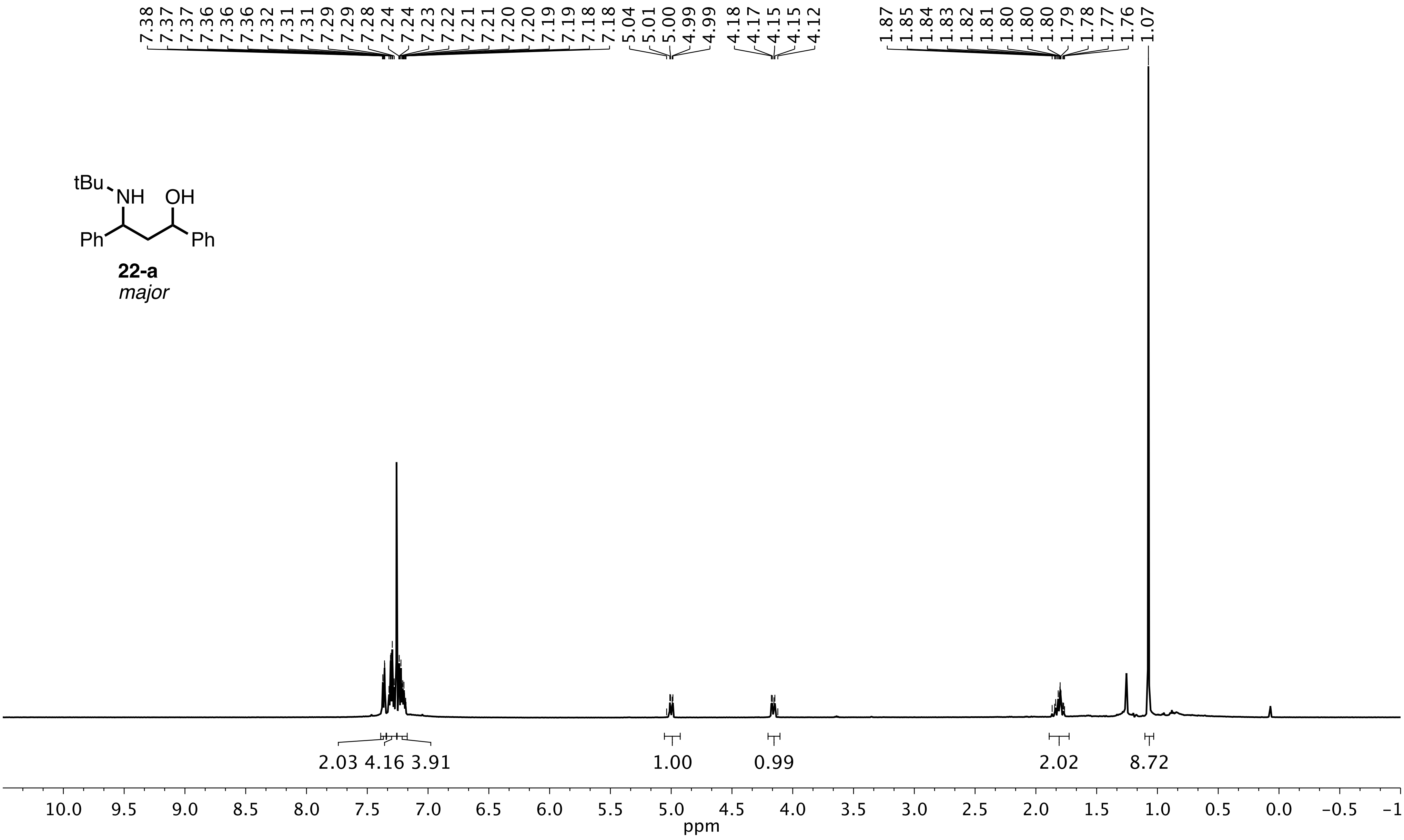

tBu
NH
OH
Ph
Ph
22-a
major
7.38
7.37
7.37
7.36
7.36
7.36
7.32
7.31
7.31
7.29
7.29
7.28
7.24
7.24
7.23
7.22
7.21
7.21
7.20
7.20
7.19
7.19
7.18
7.18
5.04
5.01
5.00
4.99
4.99
4.18
4.17
4.15
4.15
4.12
1.87
1.85
1.84
1.83
1.82
1.81
1.80
1.80
1.80
1.79
1.78
1.77
1.76
1.07
2.03 4.16 3.91
1.00
0.99
2.02
8.72
10.0 9.5 9.0 8.5 8.0 7.5 7.0 6.5 6.0 5.5 5.0 4.5 4.0 3.5 3.0 2.5 2.0 1.5 1.0 0.5 0.0 −0.5 −1
ppm

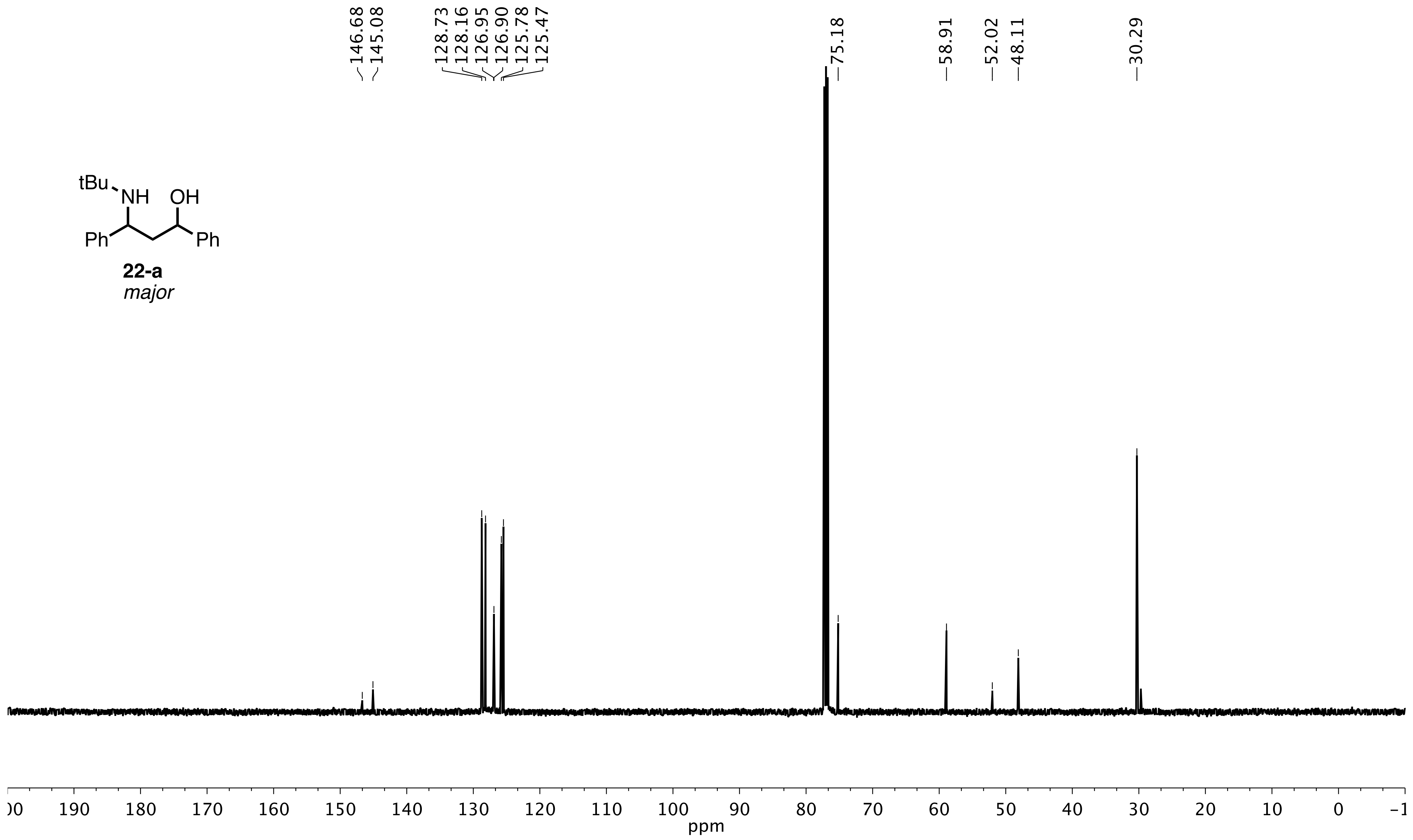

tBu
NH
OH
Ph
Ph
22-a
major
146.68
145.08
128.73
128.16
126.95
126.90
125.78
125.47
75.18
58.91
52.02
48.11
30.29
190
180
170
160
150
140
130
120
110
100
90
80
70
60
50
40
30
20
10
0
ppm

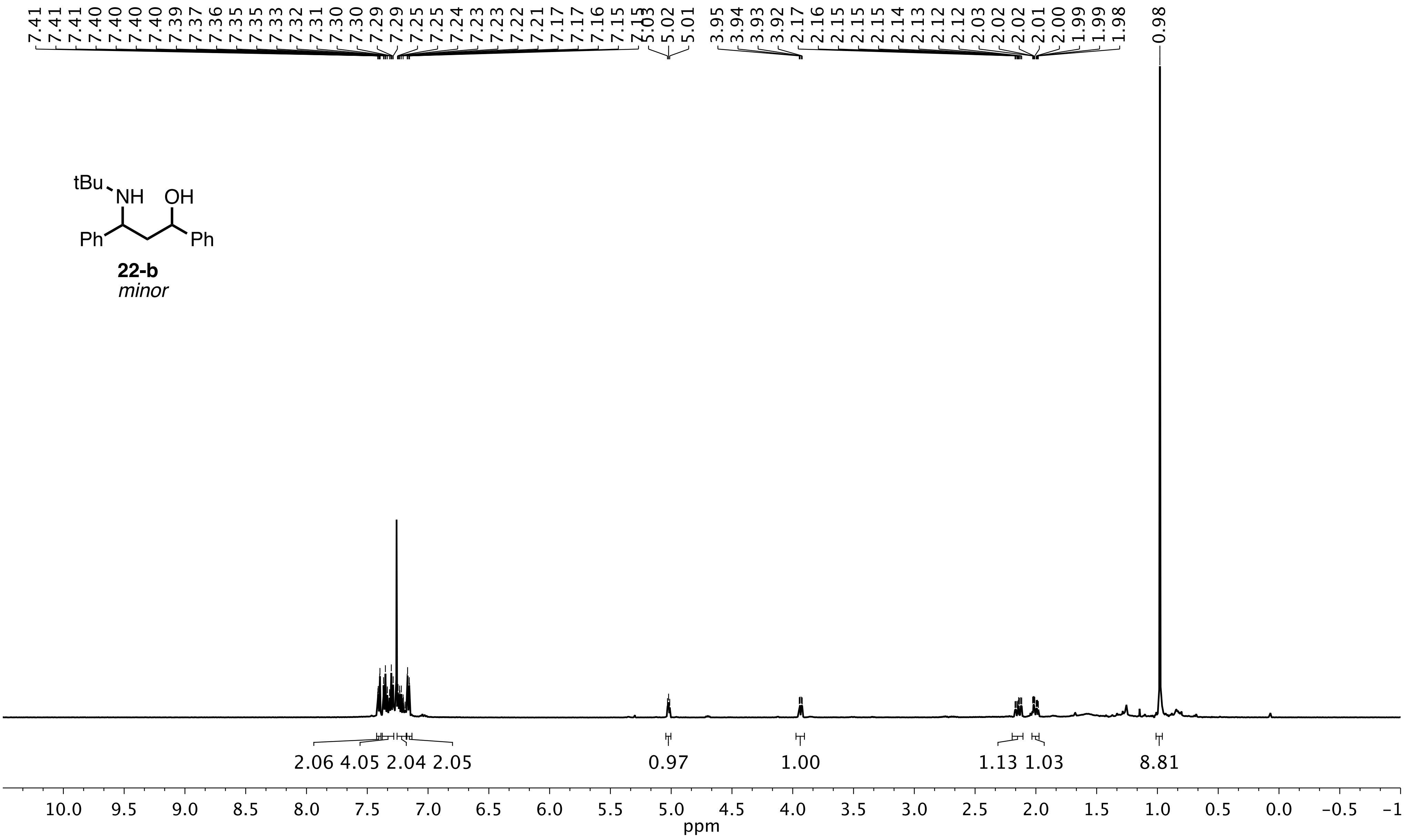

7.41 7.41 7.41 7.40 7.40 7.40 7.40 7.39 7.37 7.36 7.35 7.35 7.33 7.32 7.31 7.30 7.30 7.29 7.29 7.25 7.25 7.24 7.23 7.23 7.22 7.21 7.17 7.17 7.16 7.15 7.15
5.03 5.02 5.01
3.95 3.94 3.93 3.92 2.17 2.16 2.15 2.15 2.15 2.14 2.13 2.12 2.12 2.03 2.02 2.02 2.01 2.00 1.99 1.99 1.98
0.98
tBu
NH
OH
Ph
Ph
22-b
minor
2.06 4.05 2.04 2.05
0.97
1.00
1.13 1.03
8.81
10.0 9.5 9.0 8.5 8.0 7.5 7.0 6.5 6.0 5.5 5.0 4.5 4.0 3.5 3.0 2.5 2.0 1.5 1.0 0.5 0.0 −0.5 −1
ppm

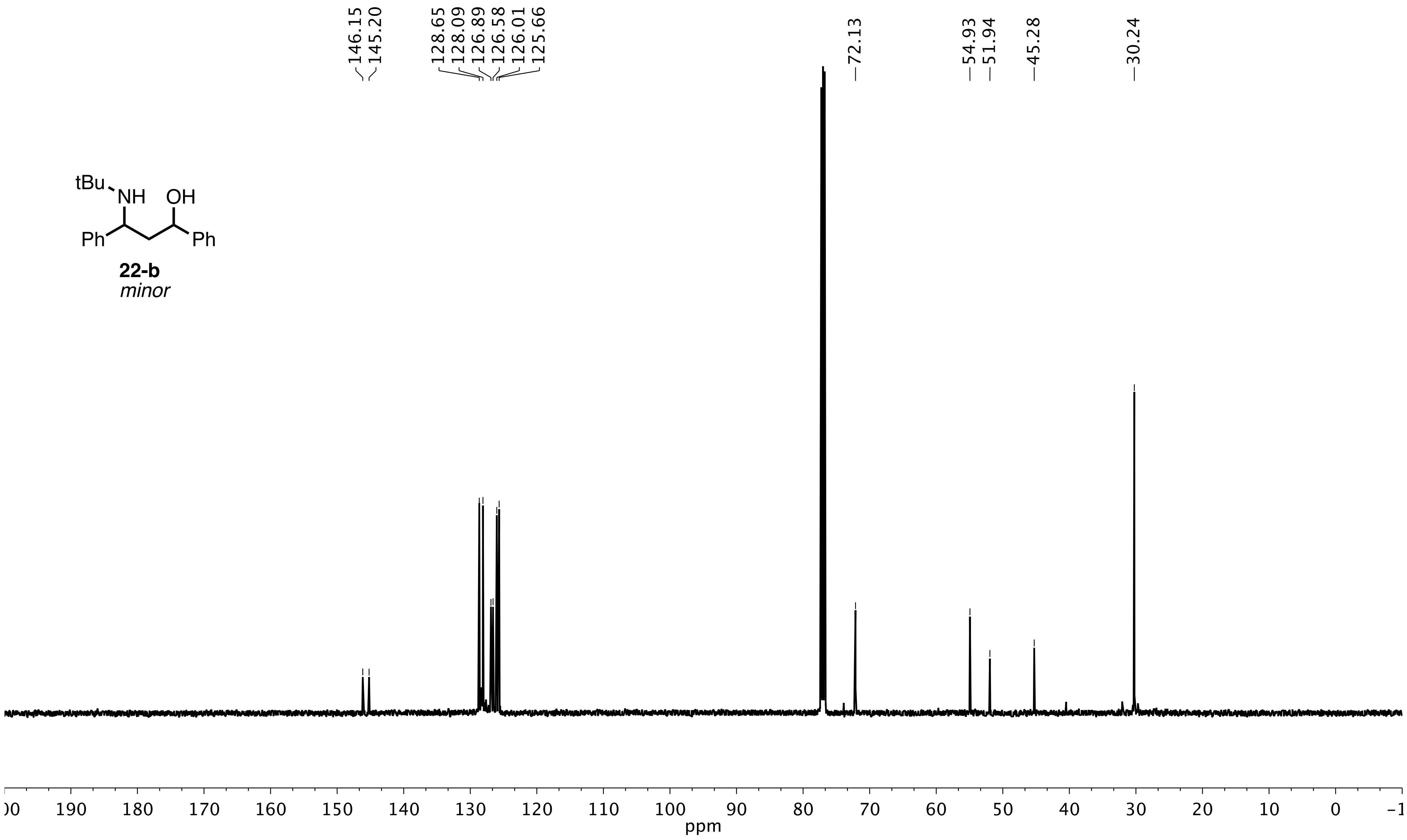
146.15
145.20
128.65
128.09
126.89
126.58
126.01
125.66
72.13
54.93
51.94
45.28
30.24
ppm

22-b
minor

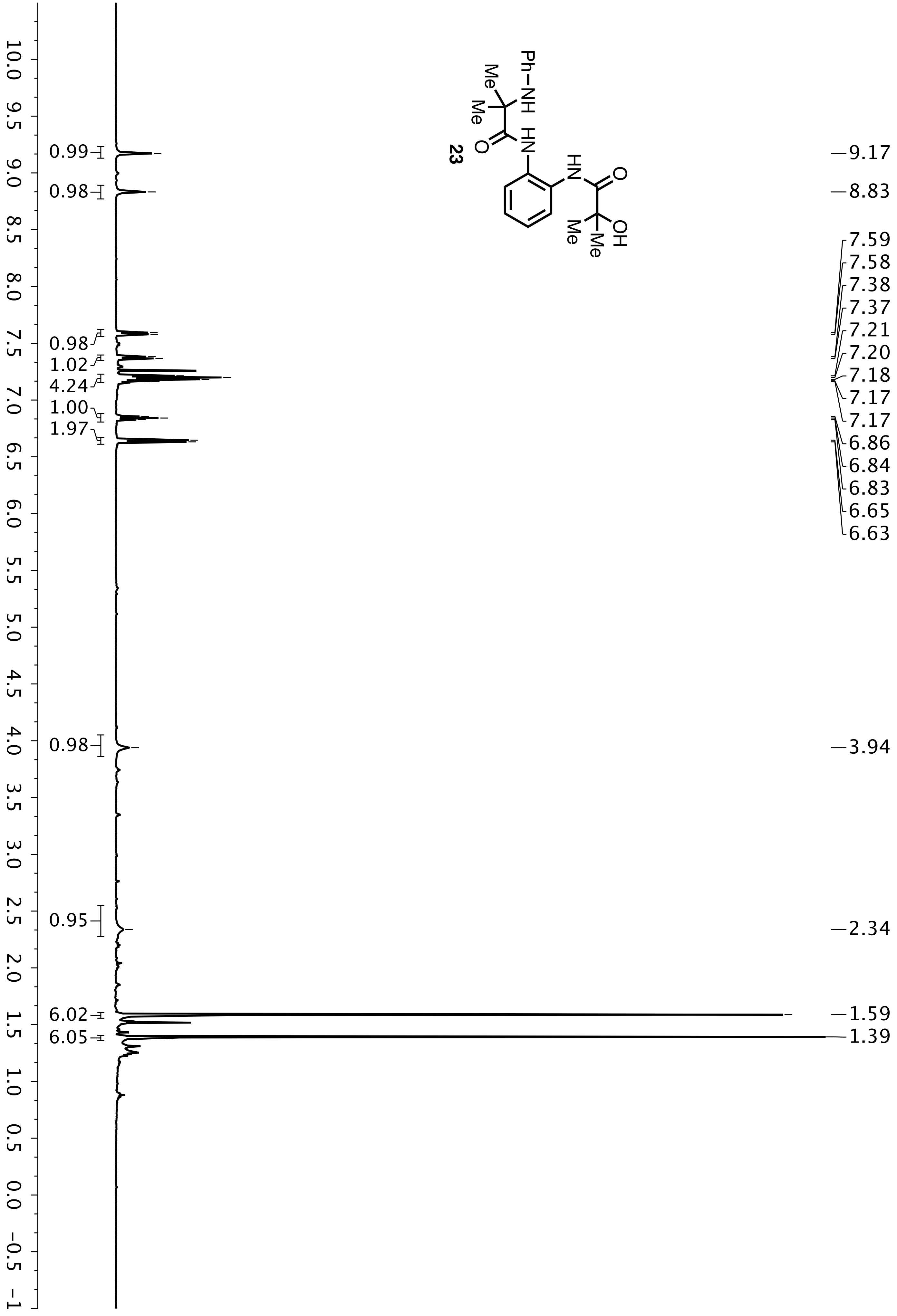

Ph–NH
HN
Me
Me
O
HN
O
OH
Me
Me
23
0.99
0.98
0.98
1.02
4.24
1.00
1.97
0.98
0.95
6.02
6.05
9.17
8.83
7.59
7.58
7.38
7.37
7.21
7.20
7.18
7.17
7.17
6.86
6.84
6.83
6.65
6.63
3.94
2.34
1.59
1.39
10.0 9.5 9.0 8.5 8.0 7.5 7.0 6.5 6.0 5.5 5.0 4.5 4.0 3.5 3.0 2.5 2.0 1.5 1.0 0.5 0.0 −0.5 −1
ppm

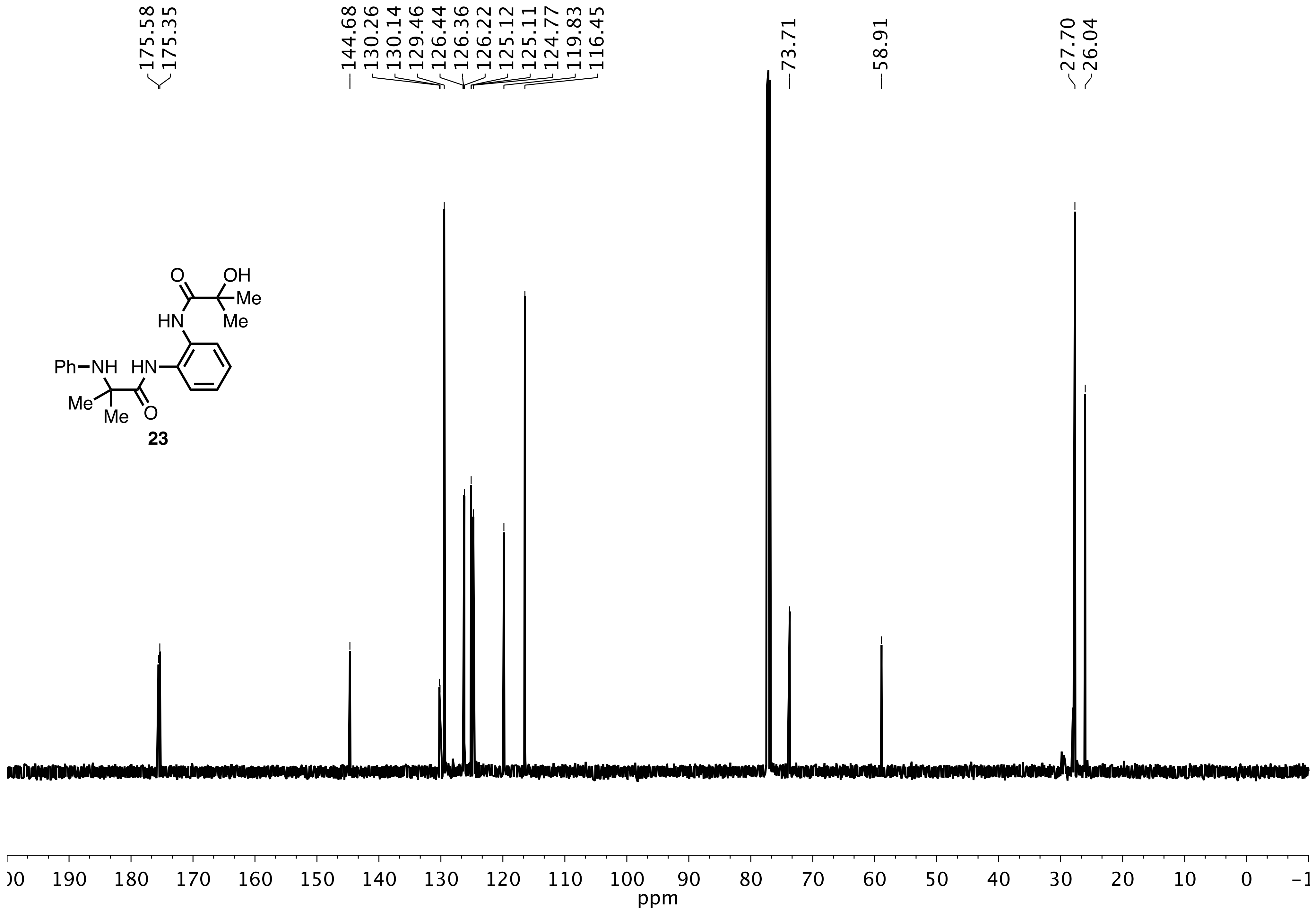
175.58
175.35
144.68
130.26
130.14
129.46
126.44
126.36
126.22
125.12
125.11
124.77
119.83
116.45
73.71
58.91
27.70
26.04
Ph−NH HN
Me Me O
HN O OH Me Me
23
00 190 180 170 160 150 140 130 120 110 100 90 80 70 60 50 40 30 20 10 0 −1
ppm

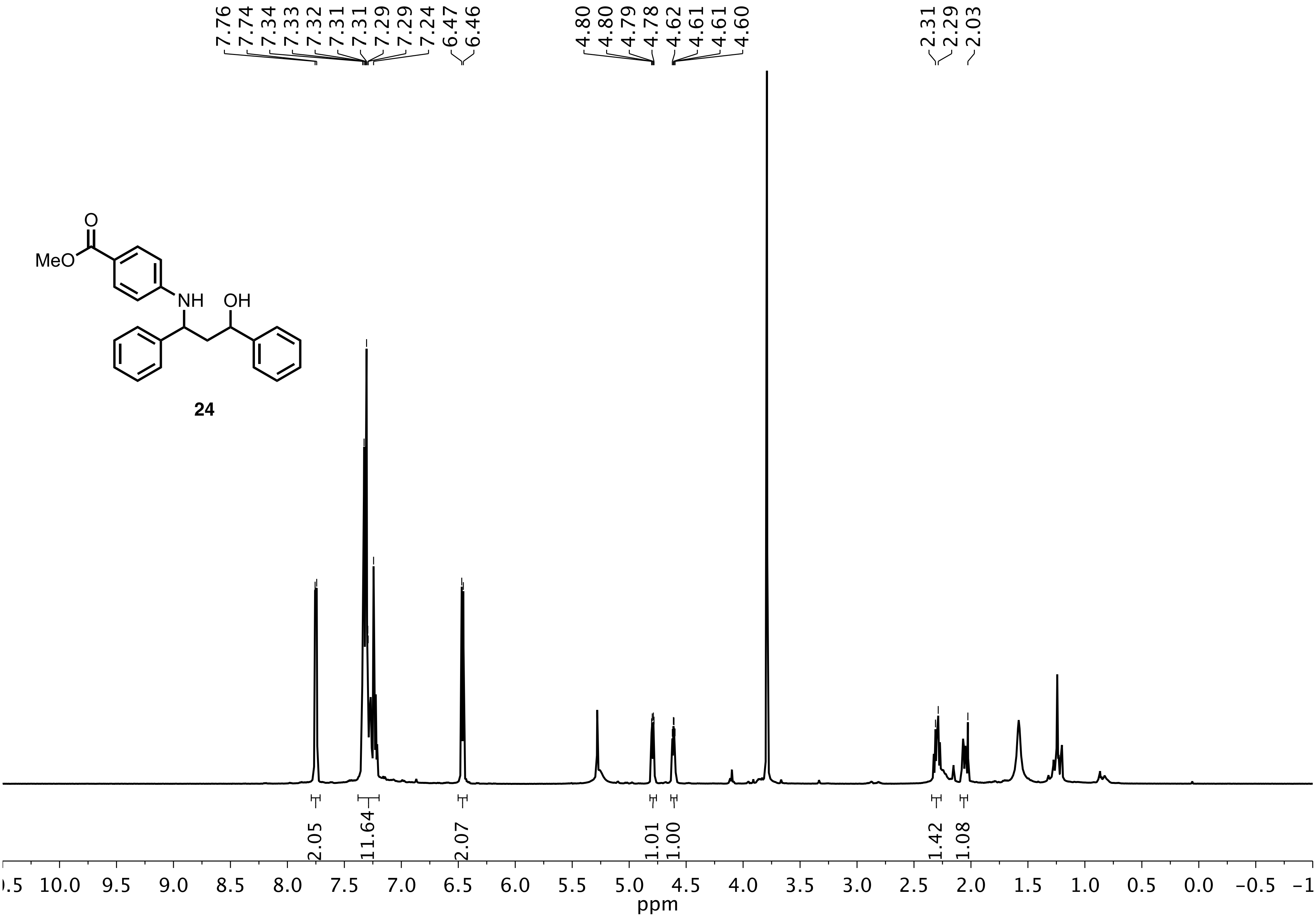
MeO
O
NH
OH
7.76
7.74
7.34
7.33
7.32
7.31
7.31
7.29
7.29
7.24
6.47
6.46
4.80
4.80
4.79
4.78
4.62
4.61
4.61
4.60
2.31
2.29
2.03
2.05
11.64
2.07
1.01
1.00
1.42
1.08
10.0
9.5
9.0
8.5
8.0
7.5
7.0
6.5
6.0
5.5
5.0
4.5
4.0
3.5
3.0
2.5
2.0
1.5
1.0
0.5
0.0
−0.5
ppm

24

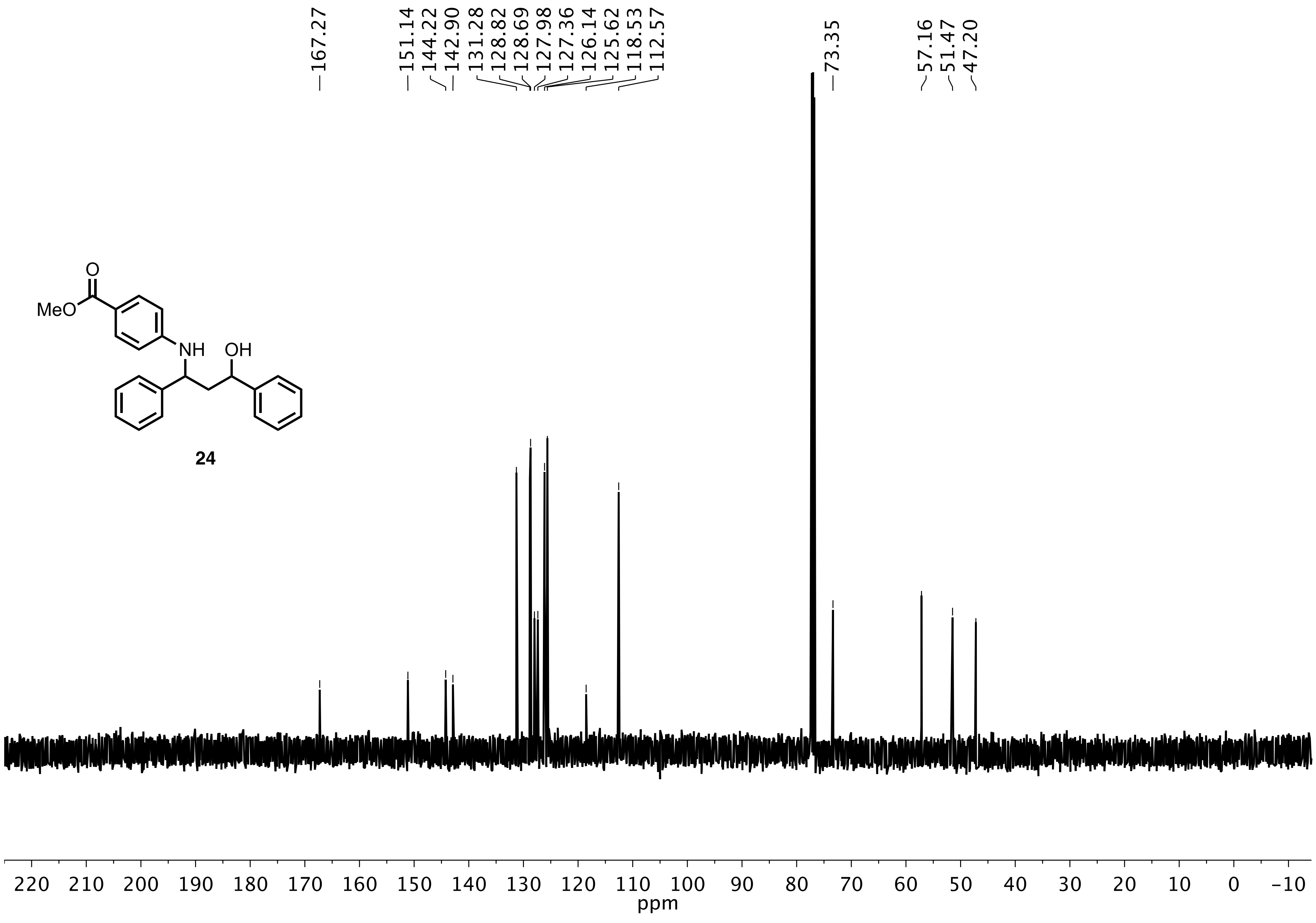
167.27
151.14
144.22
142.90
131.28
128.82
128.69
127.98
127.36
126.14
125.62
118.53
112.57
73.35
57.16
51.47
47.20
O
MeO
NH
OH
24
220 210 200 190 180 170 160 150 140 130 120 110 100 90 80 70 60 50 40 30 20 10 0 −10
ppm

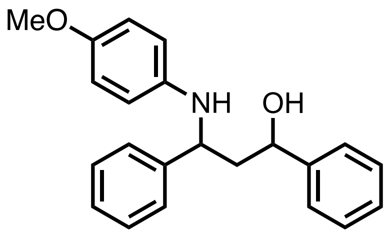
MeO
NH
OH

25

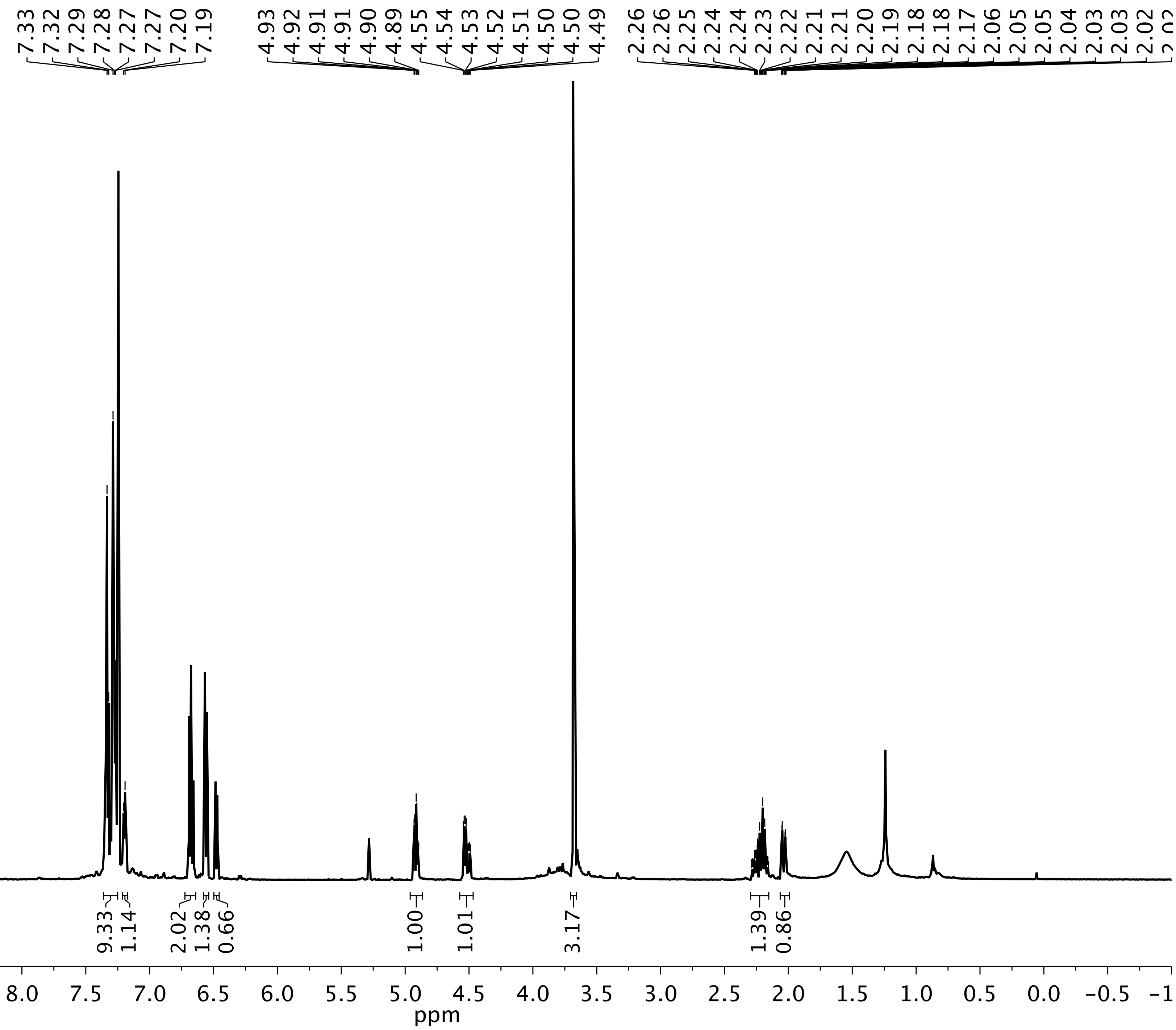
7.33
7.32
7.29
7.28
7.27
7.27
7.20
7.19
4.93
4.92
4.91
4.91
4.90
4.89
4.55
4.54
4.53
4.52
4.51
4.50
4.50
4.49
2.26
2.26
2.25
2.24
2.24
2.23
2.22
2.21
2.21
2.20
2.19
2.18
2.18
2.17
2.06
2.05
2.05
2.04
2.03
2.03
2.02
2.0
9.33
1.14
2.02
1.38
0.66
1.00
1.01
3.17
1.39
0.86
).5
10.0
9.5
9.0
8.5
8.0
7.5
7.0
6.5
6.0
5.5
5.0
4.5
4.0
3.5
3.0
2.5
2.0
1.5
1.0
0.5
0.0
−0.5
−1
ppm

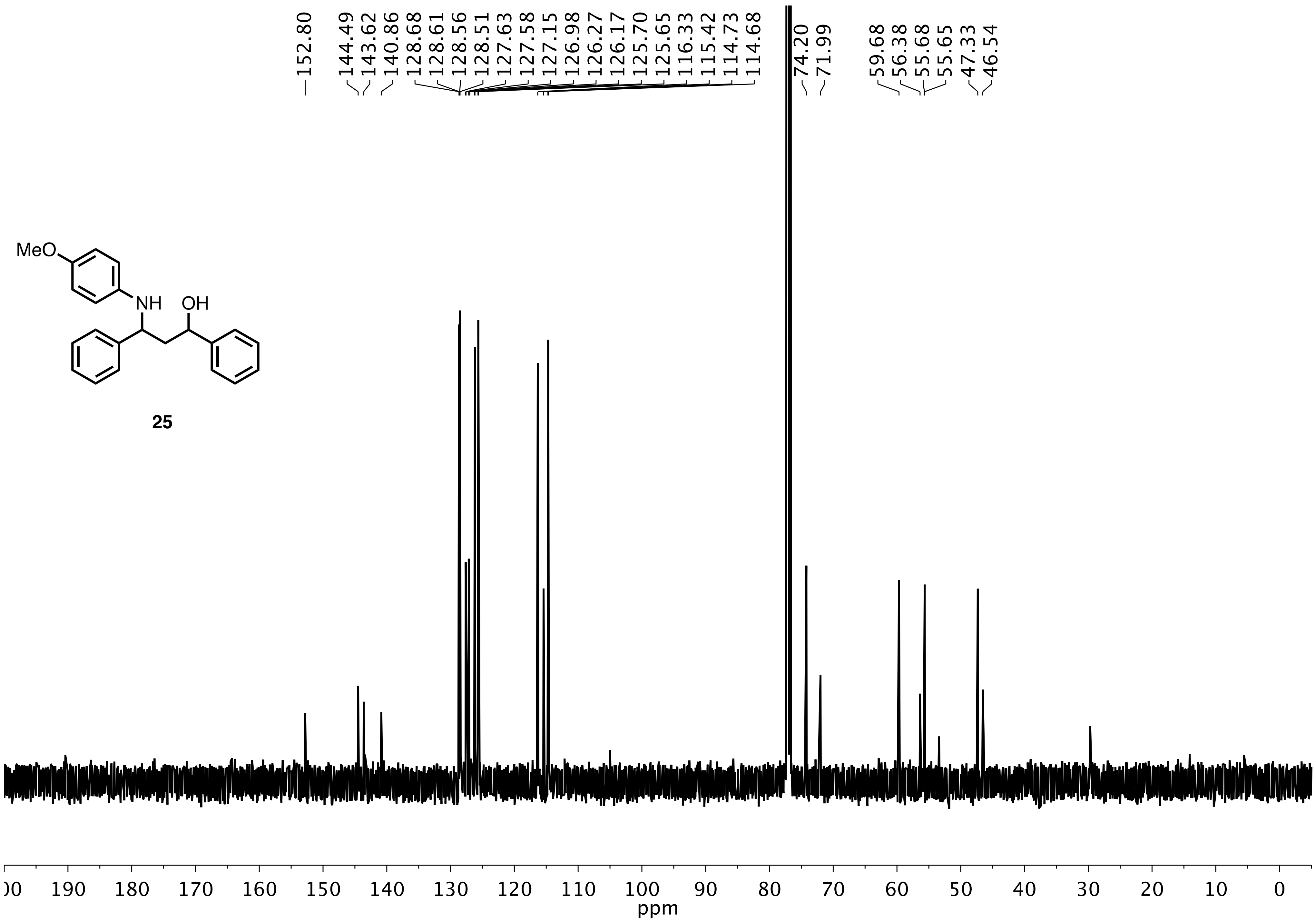

152.80
144.49
143.62
140.86
128.68
128.61
128.56
128.51
127.63
127.58
127.15
126.98
126.27
126.17
125.70
125.65
116.33
115.42
114.73
114.68
74.20
71.99
59.68
56.38
55.68
55.65
47.33
46.54
MeO
NH
OH
25
190
180
170
160
150
140
130
120
110
100
90
80
70
60
50
40
30
20
10
0
ppm

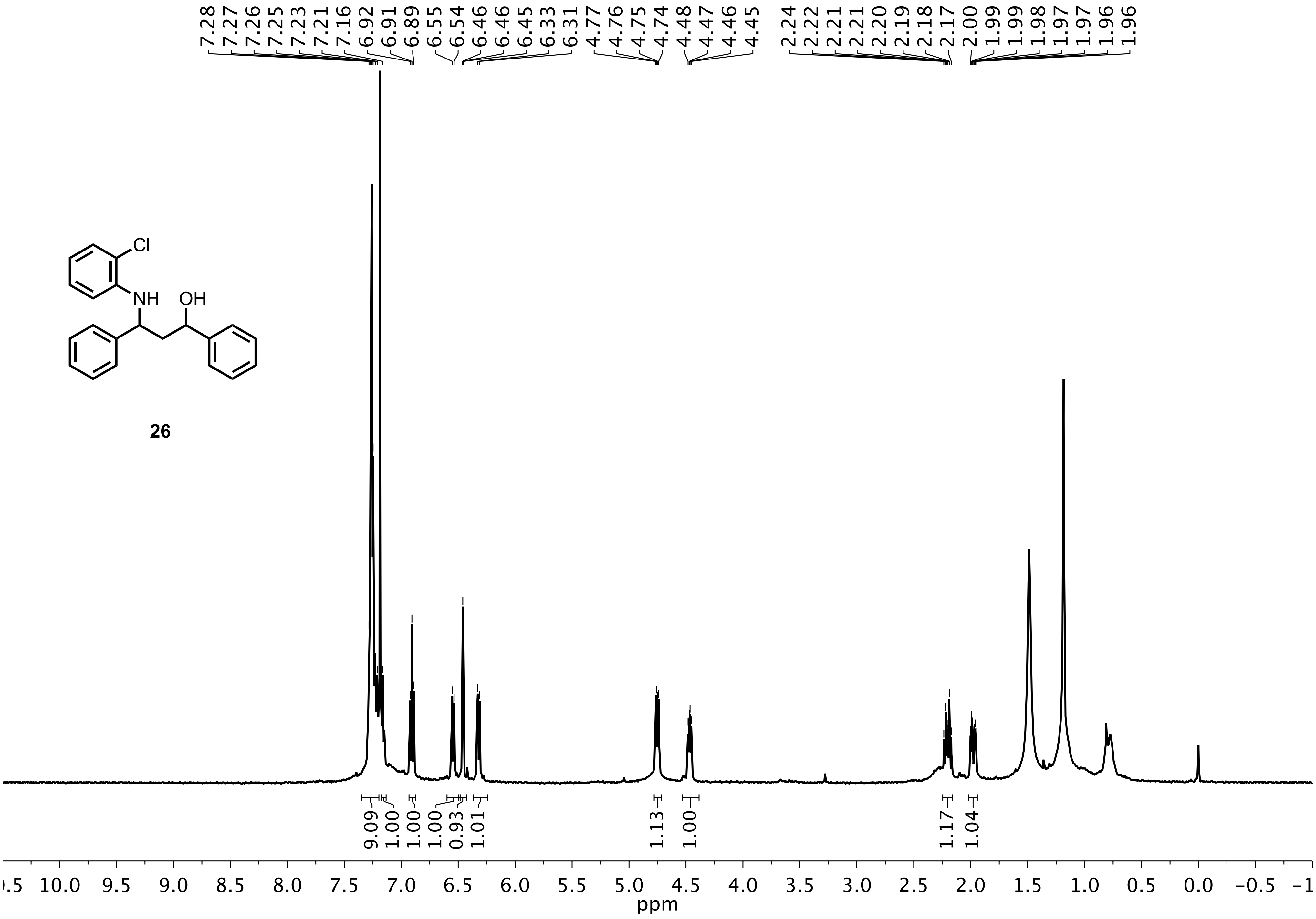
Cl
NH
OH
7.28 7.27 7.26 7.25 7.23 7.21 7.16 6.92 6.91 6.89 6.55 6.54 6.46 6.46 6.45 6.33 6.31 4.77 4.76 4.75 4.74 4.48 4.47 4.46 4.45 2.24 2.22 2.21 2.21 2.20 2.19 2.18 2.17 2.00 1.99 1.99 1.98 1.97 1.97 1.96 1.96
9.09 1.00 1.00 1.00 0.93 1.01 1.13 1.00 1.17 1.04
10.0 9.5 9.0 8.5 8.0 7.5 7.0 6.5 6.0 5.5 5.0 4.5 4.0 3.5 3.0 2.5 2.0 1.5 1.0 0.5 0.0 −0.5
ppm

26

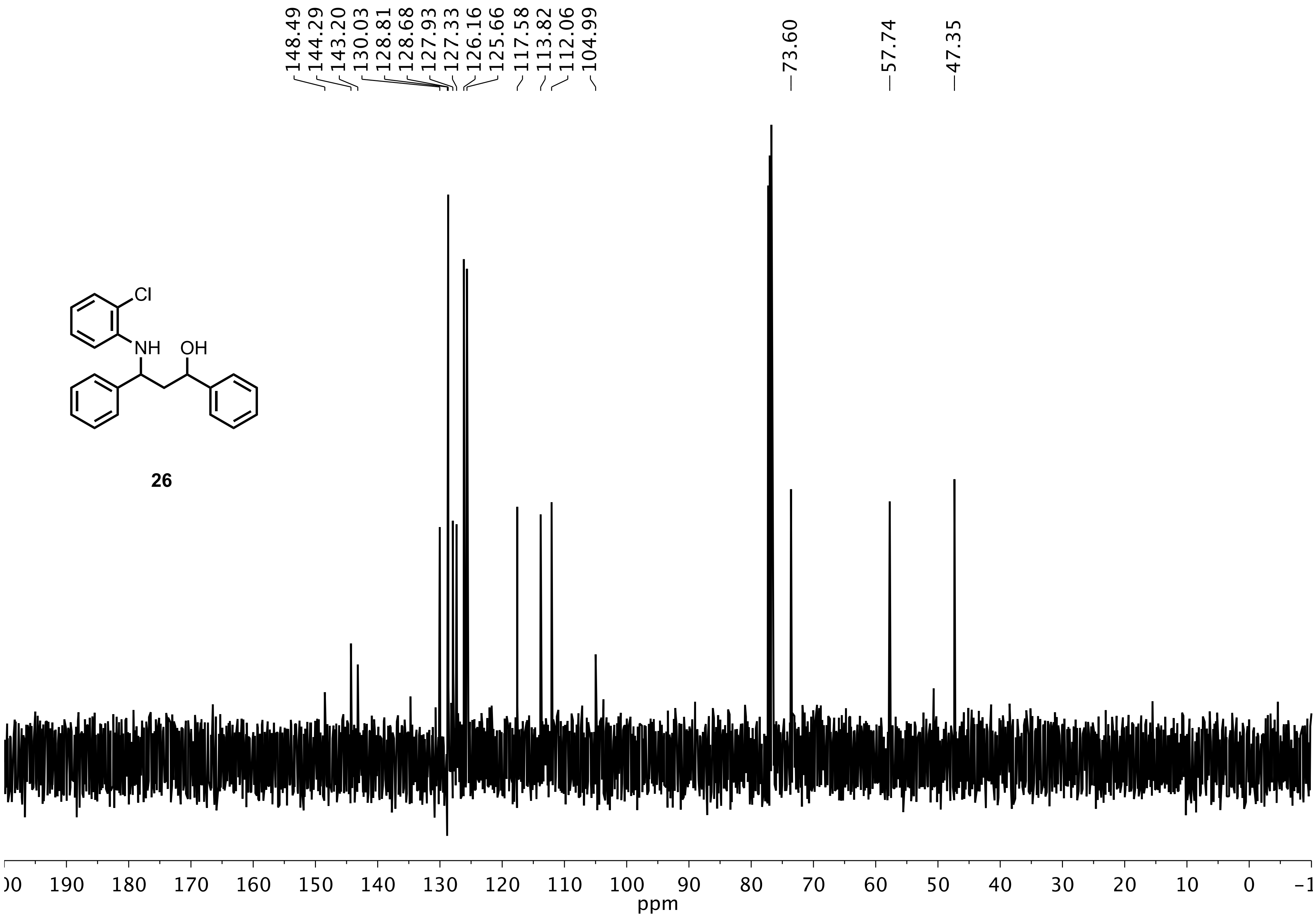
Cl
NH
OH
148.49
144.29
143.20
130.03
128.81
128.68
127.93
127.33
126.16
125.66
117.58
113.82
112.06
104.99
73.60
57.74
47.35
190
180
170
160
150
140
130
120
110
100
90
80
70
60
50
40
30
20
10
0
ppm

26

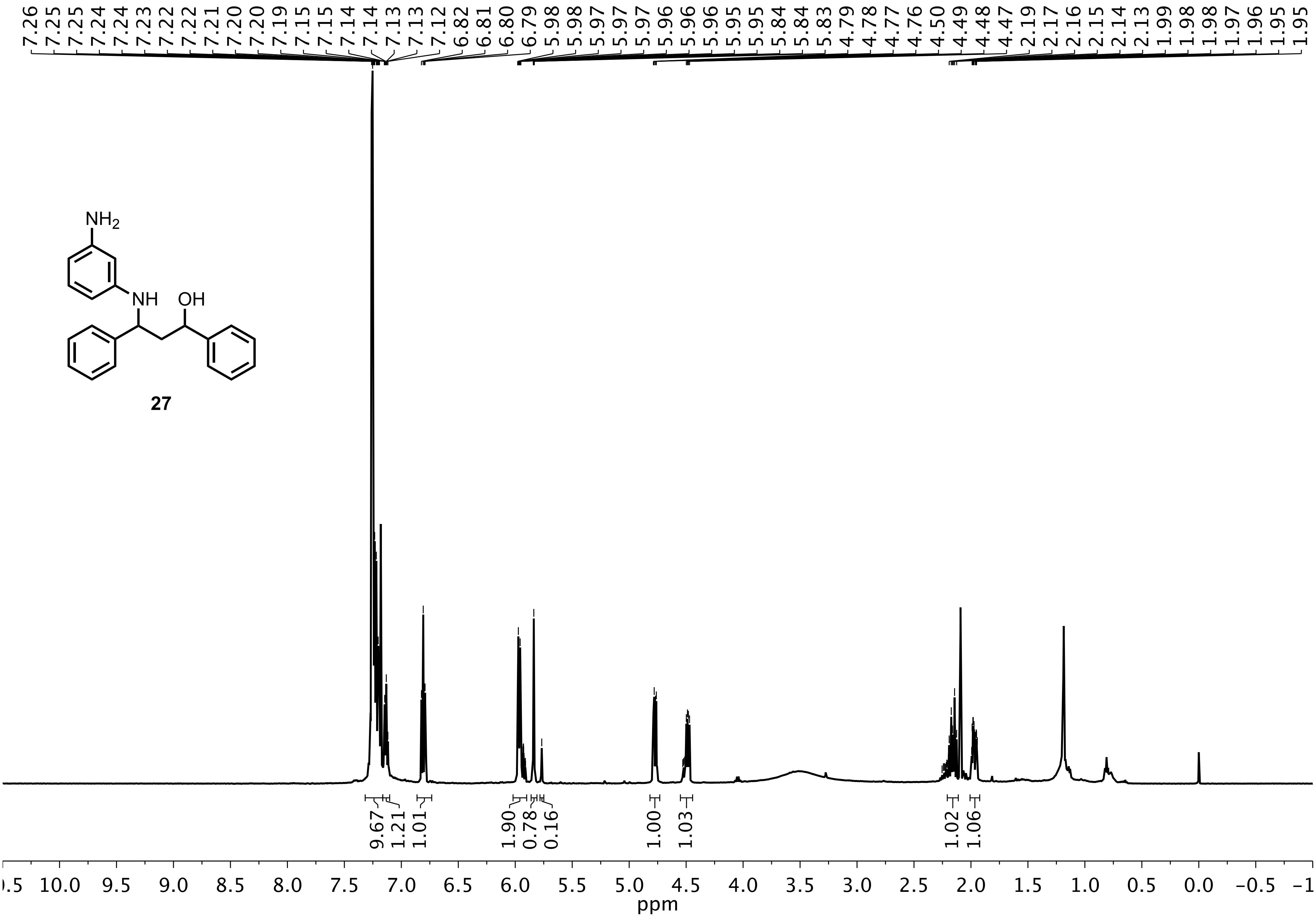
7.26
7.25
7.25
7.24
7.24
7.23
7.22
7.22
7.21
7.20
7.20
7.19
7.15
7.15
7.14
7.14
7.13
7.13
7.12
6.82
6.81
6.80
6.79
5.98
5.98
5.97
5.97
5.97
5.96
5.96
5.96
5.95
5.95
5.84
5.84
5.83
4.79
4.78
4.77
4.76
4.50
4.49
4.48
4.47
2.19
2.17
2.16
2.15
2.14
2.13
1.99
1.98
1.98
1.97
1.96
1.95
1.95
NH2
NH
OH
27
9.67
1.21
1.01
1.90
0.78
0.16
1.00
1.03
1.02
1.06
.5
10.0
9.5
9.0
8.5
8.0
7.5
7.0
6.5
6.0
5.5
5.0
4.5
4.0
3.5
3.0
2.5
2.0
1.5
1.0
0.5
0.0
−0.5
−1
ppm

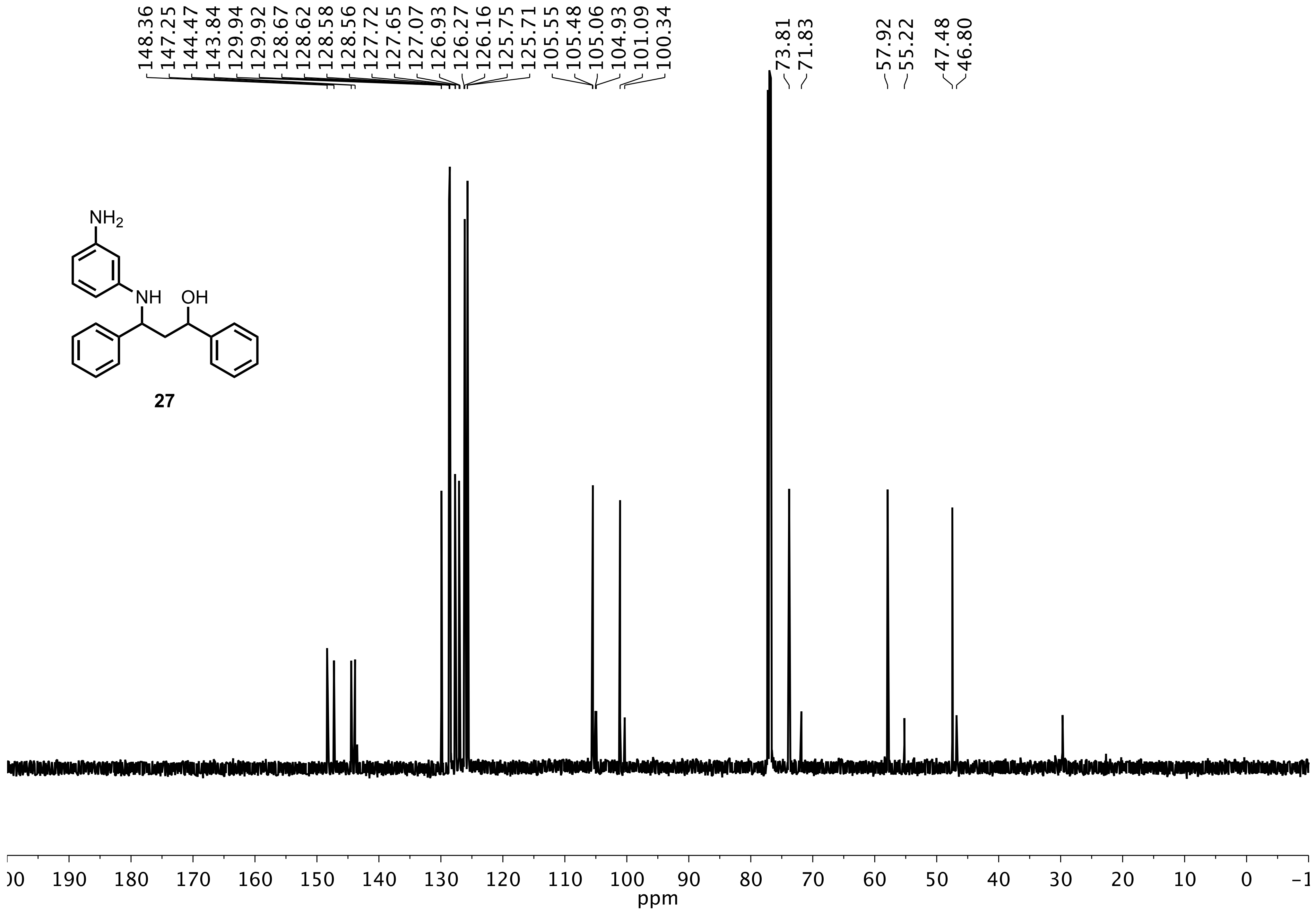
148.36
147.25
144.47
143.84
129.94
129.92
128.67
128.62
128.58
128.56
127.72
127.65
127.07
126.93
126.27
126.16
125.75
125.71
105.55
105.48
105.06
104.93
101.09
100.34
73.81
71.83
57.92
55.22
47.48
46.80
NH2
NH
OH
27
190 180 170 160 150 140 130 120 110 100 90 80 70 60 50 40 30 20 10 0
ppm

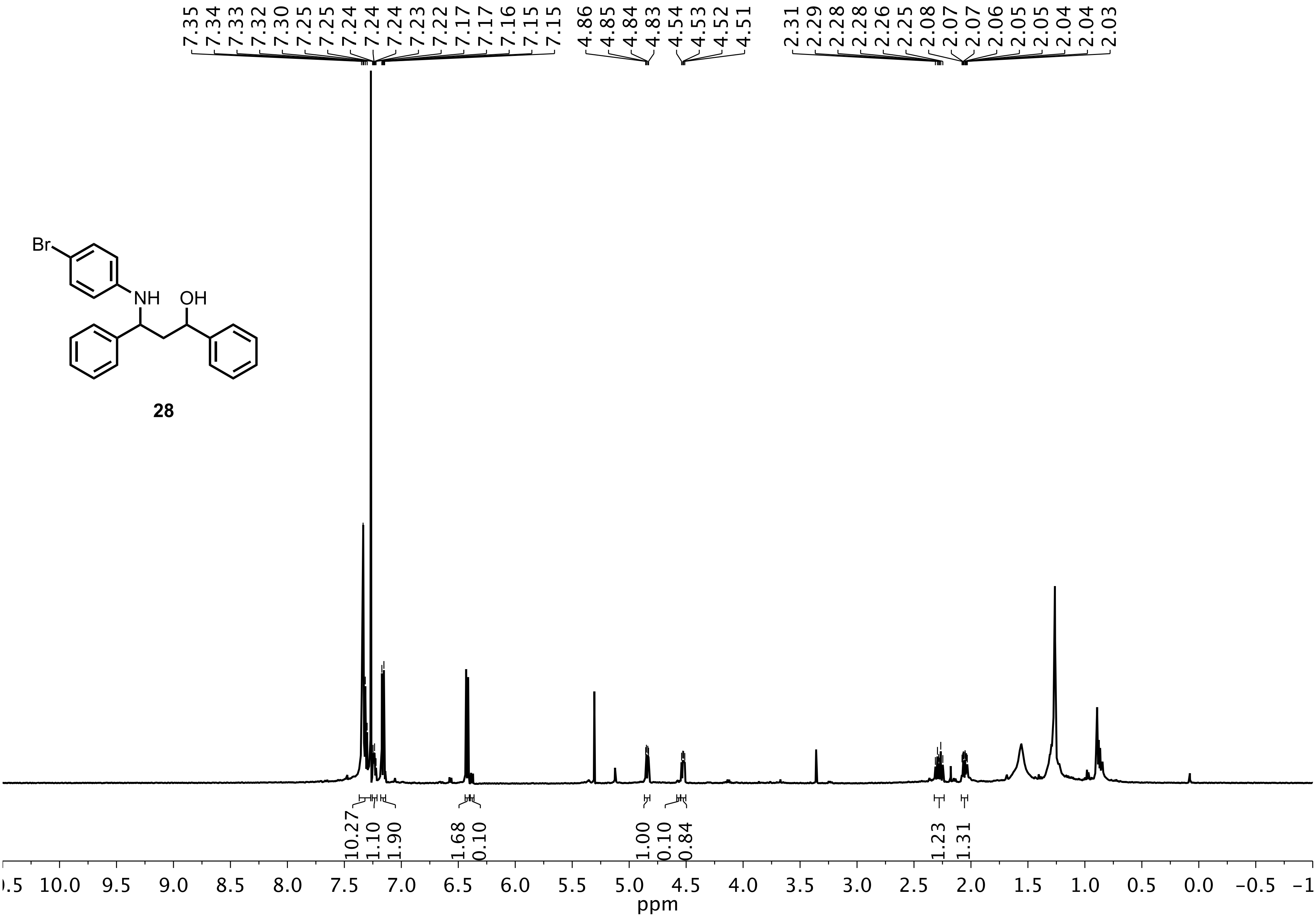
7.35
7.34
7.33
7.32
7.30
7.25
7.25
7.24
7.24
7.24
7.23
7.22
7.17
7.17
7.16
7.15
7.15
4.86
4.85
4.84
4.83
4.54
4.53
4.52
4.51
2.31
2.29
2.28
2.28
2.26
2.25
2.08
2.07
2.07
2.06
2.05
2.05
2.04
2.04
2.03
Br
NH
OH
10.27
1.10
1.90
1.68
0.10
1.00
0.10
0.84
1.23
1.31
10.0
9.5
9.0
8.5
8.0
7.5
7.0
6.5
6.0
5.5
5.0
4.5
4.0
3.5
3.0
2.5
2.0
1.5
1.0
0.5
0.0
−0.5
ppm

28

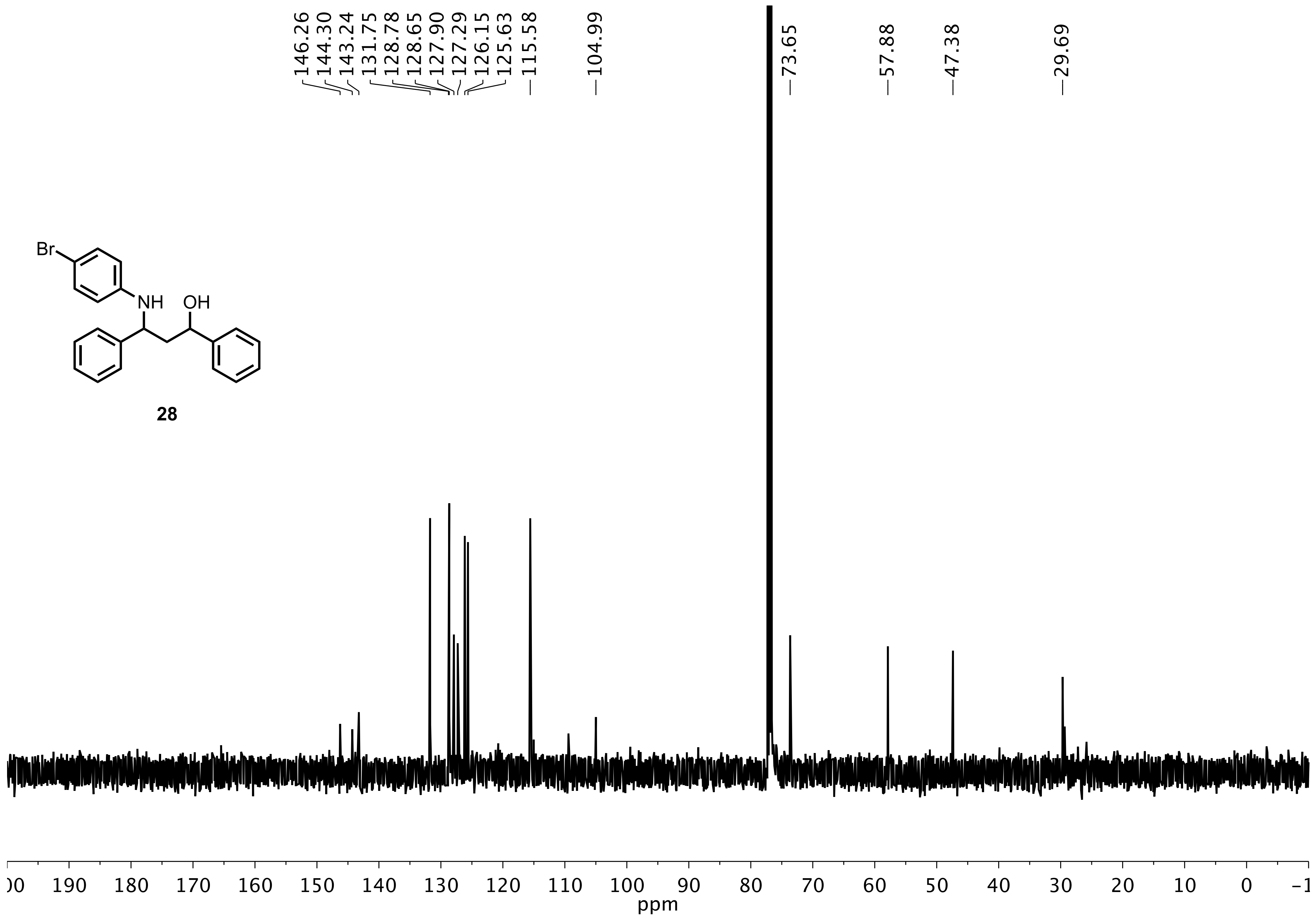

Br
NH
OH
28
146.26
144.30
143.24
131.75
128.78
128.65
127.90
127.29
126.15
125.63
115.58
104.99
73.65
57.88
47.38
29.69
190
180
170
160
150
140
130
120
110
100
90
80
70
60
50
40
30
20
10
0
ppm

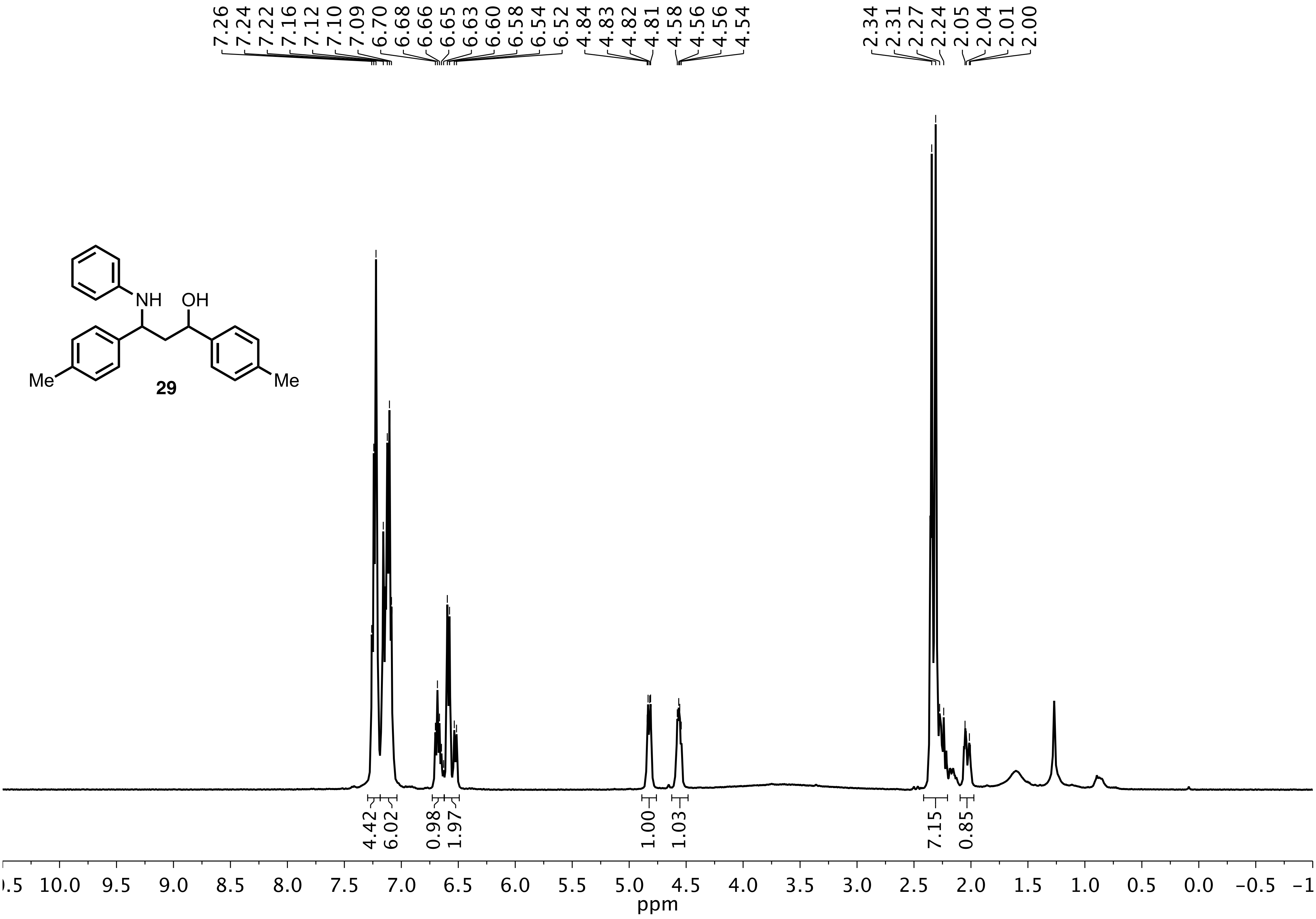
7.26
7.24
7.22
7.16
7.12
7.10
7.09
6.70
6.68
6.66
6.65
6.63
6.60
6.58
6.54
6.52
4.84
4.83
4.82
4.81
4.58
4.56
4.56
4.54
2.34
2.31
2.27
2.24
2.05
2.04
2.01
2.00
NH
OH
Me
Me
29
4.42
6.02
0.98
1.97
1.00
1.03
7.15
0.85
10.0
9.5
9.0
8.5
8.0
7.5
7.0
6.5
6.0
5.5
5.0
4.5
4.0
3.5
3.0
2.5
2.0
1.5
1.0
0.5
0.0
−0.5
ppm

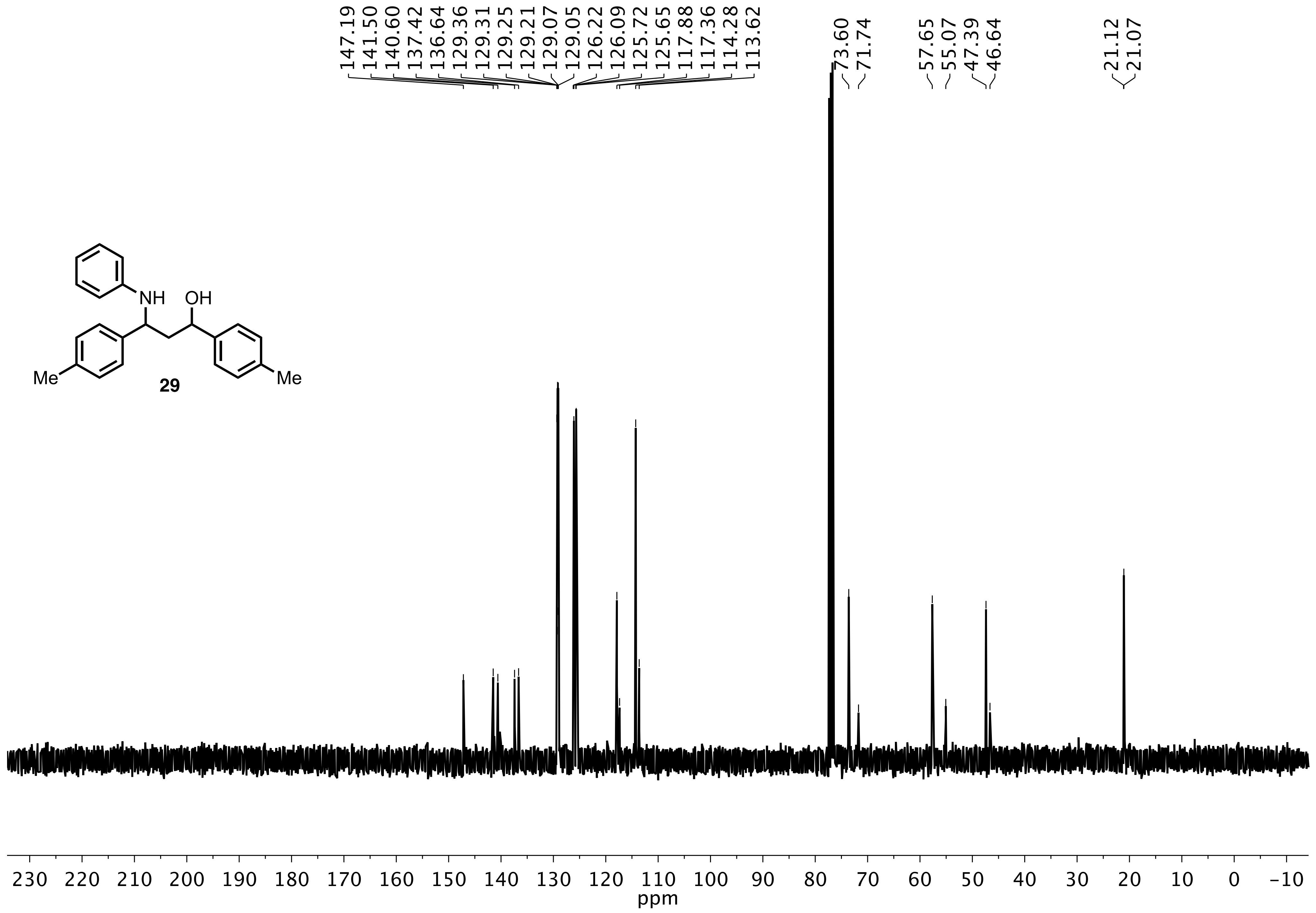
147.19
141.50
140.60
137.42
136.64
129.36
129.31
129.25
129.21
129.07
129.05
126.22
126.09
125.72
125.65
117.88
117.36
114.28
113.62
73.60
71.74
57.65
55.07
47.39
46.64
21.12
21.07
NH
OH
Me
Me
29
230 220 210 200 190 180 170 160 150 140 130 120 110 100 90 80 70 60 50 40 30 20 10 0 −10
ppm

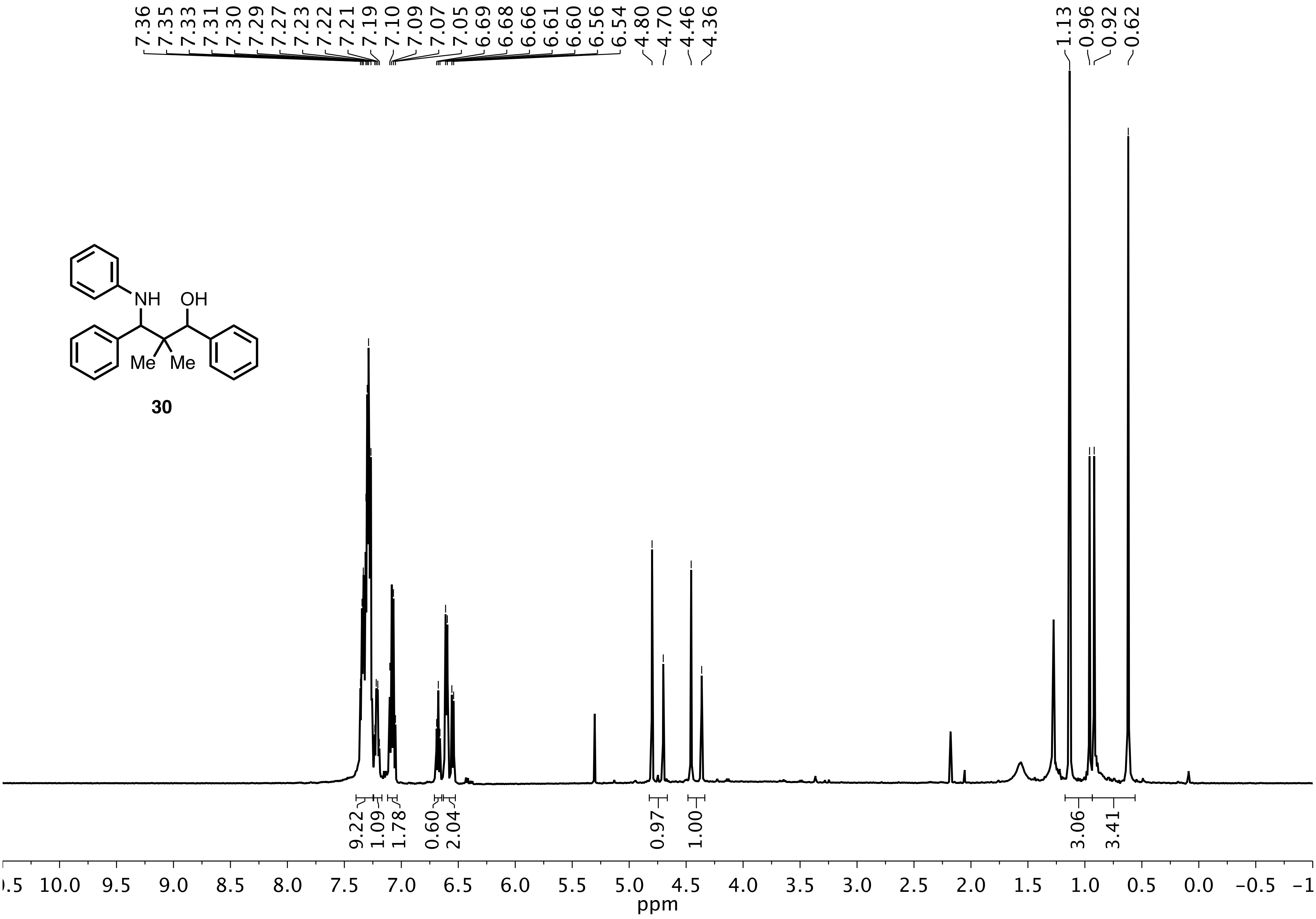
7.36
7.35
7.33
7.31
7.30
7.29
7.27
7.23
7.22
7.21
7.19
7.10
7.09
7.07
7.05
6.69
6.68
6.66
6.61
6.60
6.56
6.54
4.80
4.70
4.46
4.36
1.13
0.96
0.92
0.62
NH
OH
Me
Me
30
9.22
1.09
1.78
0.60
2.04
0.97
1.00
3.06
3.41
10.0
9.5
9.0
8.5
8.0
7.5
7.0
6.5
6.0
5.5
5.0
4.5
4.0
3.5
3.0
2.5
2.0
1.5
1.0
0.5
0.0
−0.5
ppm

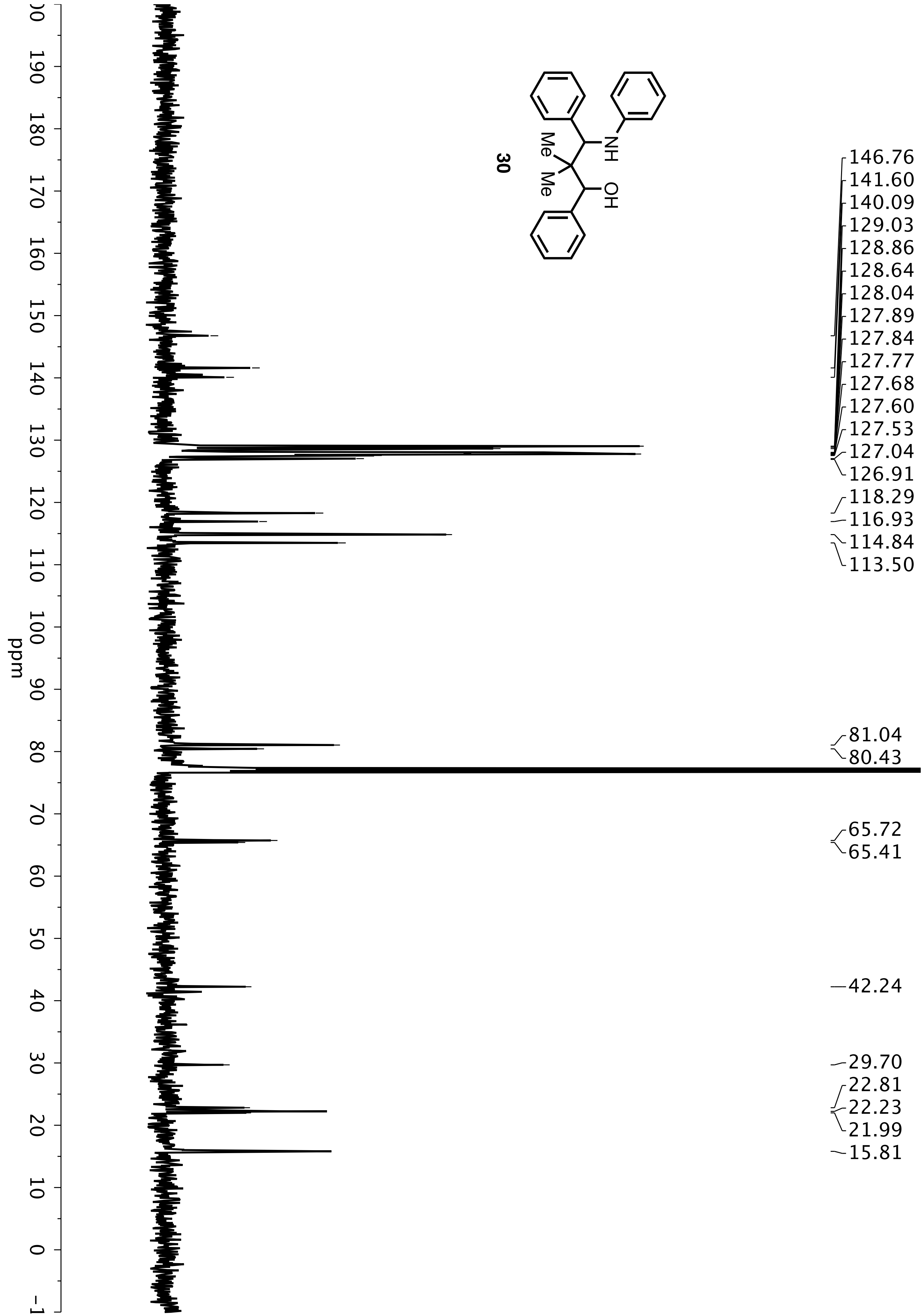

NH
OH
Me
Me
30
146.76
141.60
140.09
129.03
128.86
128.64
128.04
127.89
127.84
127.77
127.68
127.60
127.53
127.04
126.91
118.29
116.93
114.84
113.50
81.04
80.43
65.72
65.41
42.24
29.70
22.81
22.23
21.99
15.81
ppm

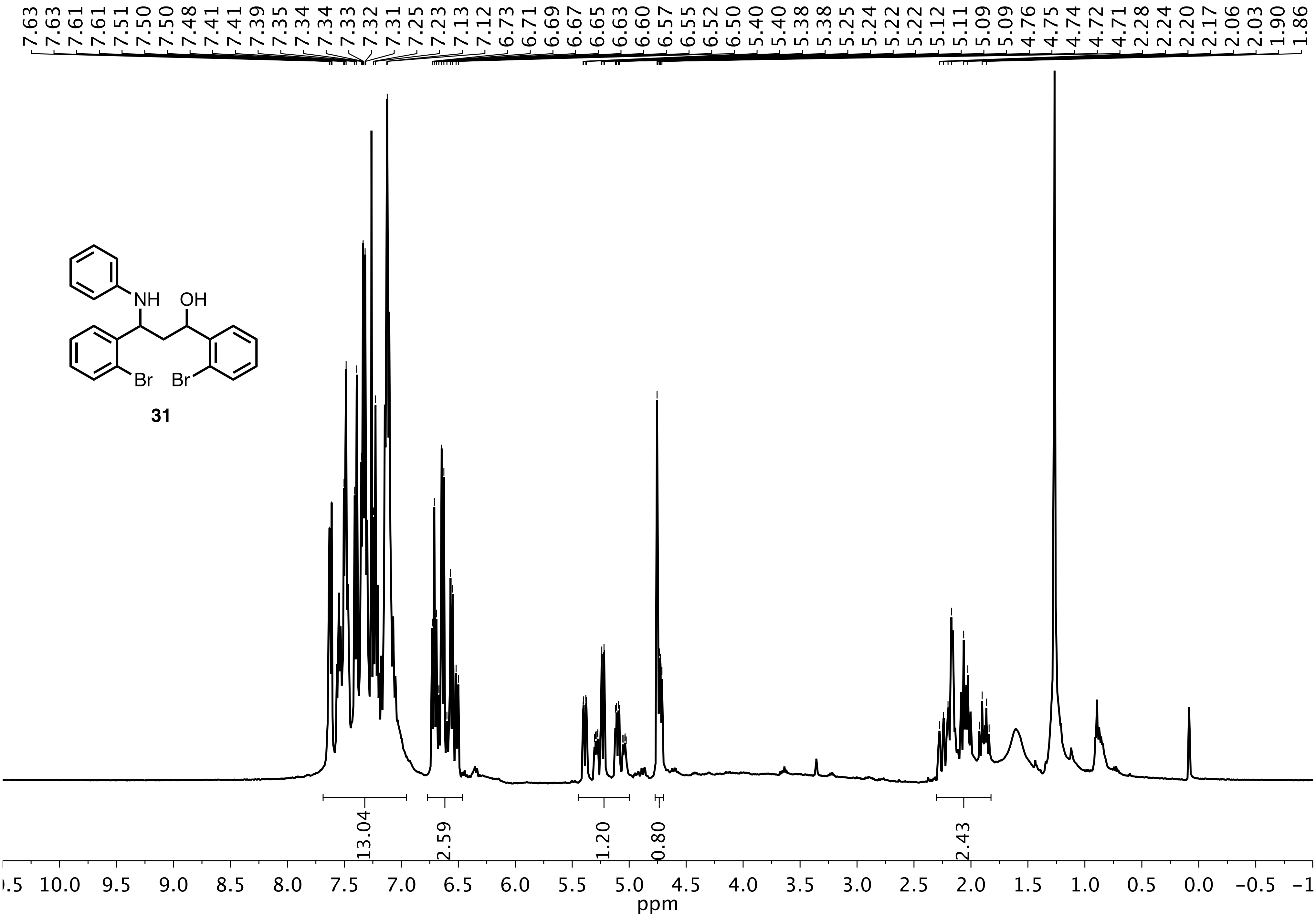
7.63
7.63
7.61
7.61
7.51
7.50
7.50
7.48
7.41
7.41
7.39
7.35
7.34
7.34
7.33
7.32
7.31
7.25
7.23
7.13
7.12
6.73
6.71
6.69
6.67
6.65
6.63
6.60
6.57
6.55
6.52
6.50
5.40
5.40
5.38
5.38
5.25
5.24
5.22
5.22
5.12
5.11
5.09
5.09
4.76
4.75
4.74
4.72
4.71
2.28
2.24
2.20
2.17
2.06
2.03
1.90
1.86
NH
OH
Br
Br
31
13.04
2.59
1.20
0.80
2.43
10.0
9.5
9.0
8.5
8.0
7.5
7.0
6.5
6.0
5.5
5.0
4.5
4.0
3.5
3.0
2.5
2.0
1.5
1.0
0.5
0.0
−0.5
ppm

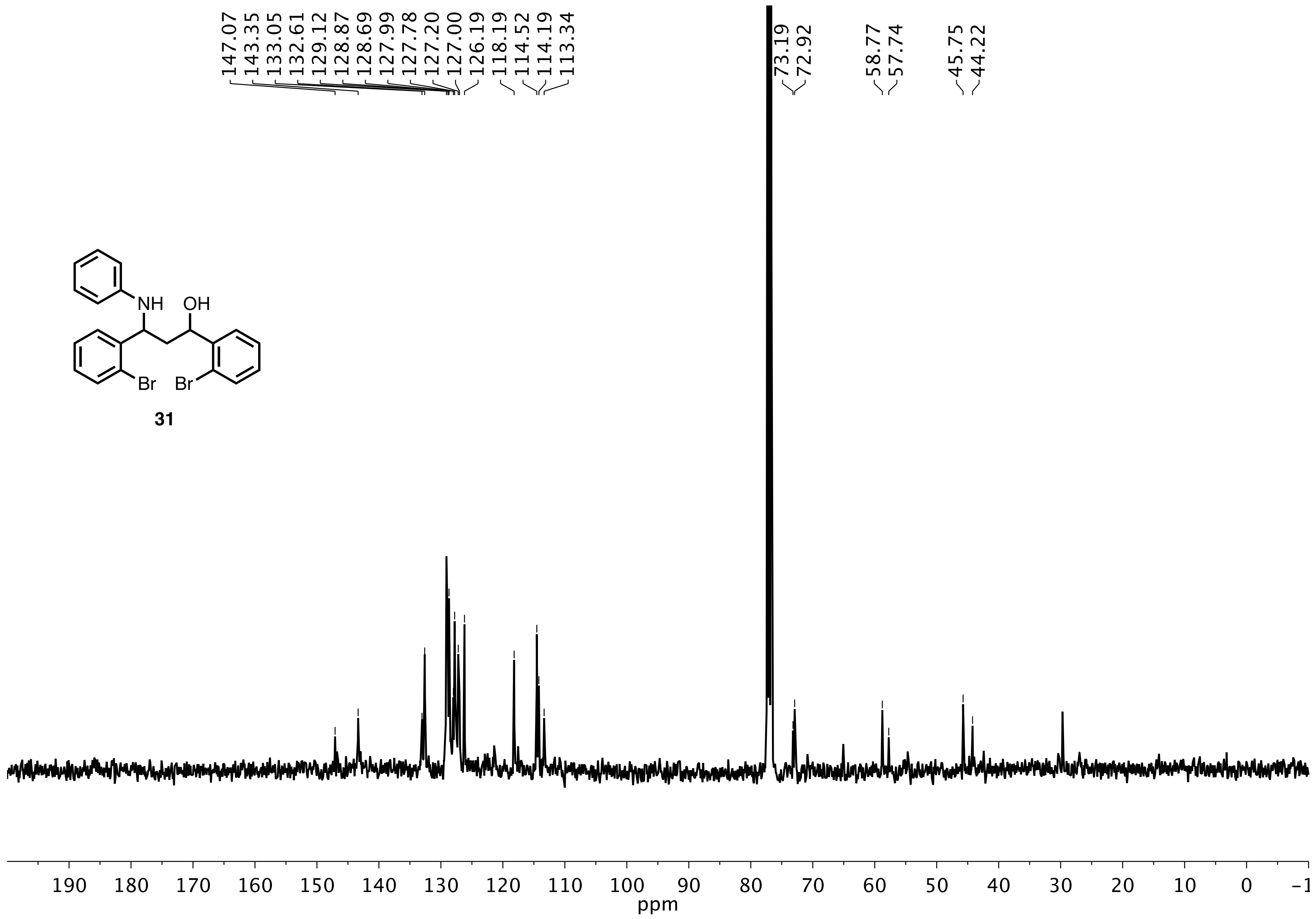
147.07
143.35
133.05
132.61
129.12
128.87
128.69
127.99
127.78
127.20
127.00
126.19
118.19
114.52
114.19
113.34
73.19
72.92
58.77
57.74
45.75
44.22
NH
OH
Br
Br
31
190 180 170 160 150 140 130 120 110 100 90 80 70 60 50 40 30 20 10 0 −1
ppm

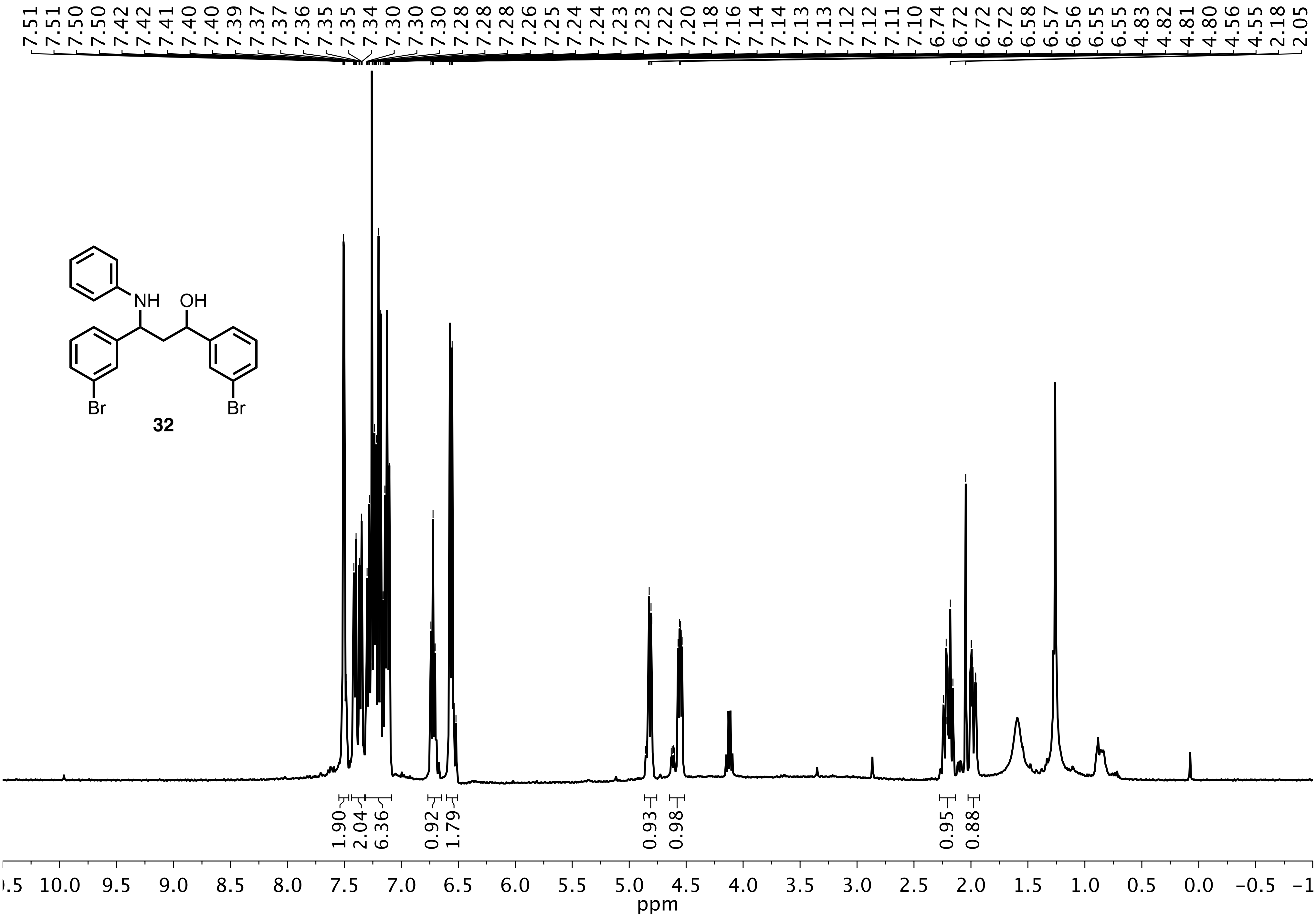
7.51 7.51 7.50 7.50 7.42 7.42 7.41 7.40 7.40 7.39 7.37 7.37 7.36 7.35 7.35 7.34 7.30 7.30 7.30 7.28 7.28 7.28 7.26 7.25 7.24 7.24 7.23 7.23 7.22 7.20 7.18 7.16 7.14 7.14 7.13 7.13 7.12 7.12 7.11 7.10 6.74 6.72 6.72 6.72 6.58 6.57 6.56 6.55 6.55 4.83 4.82 4.81 4.80 4.56 4.55 2.18 2.05
NH
OH
Br
Br
32
1.90
2.04
6.36
0.92
1.79
0.93
0.98
0.95
0.88
10.0 9.5 9.0 8.5 8.0 7.5 7.0 6.5 6.0 5.5 5.0 4.5 4.0 3.5 3.0 2.5 2.0 1.5 1.0 0.5 0.0 −0.5
ppm

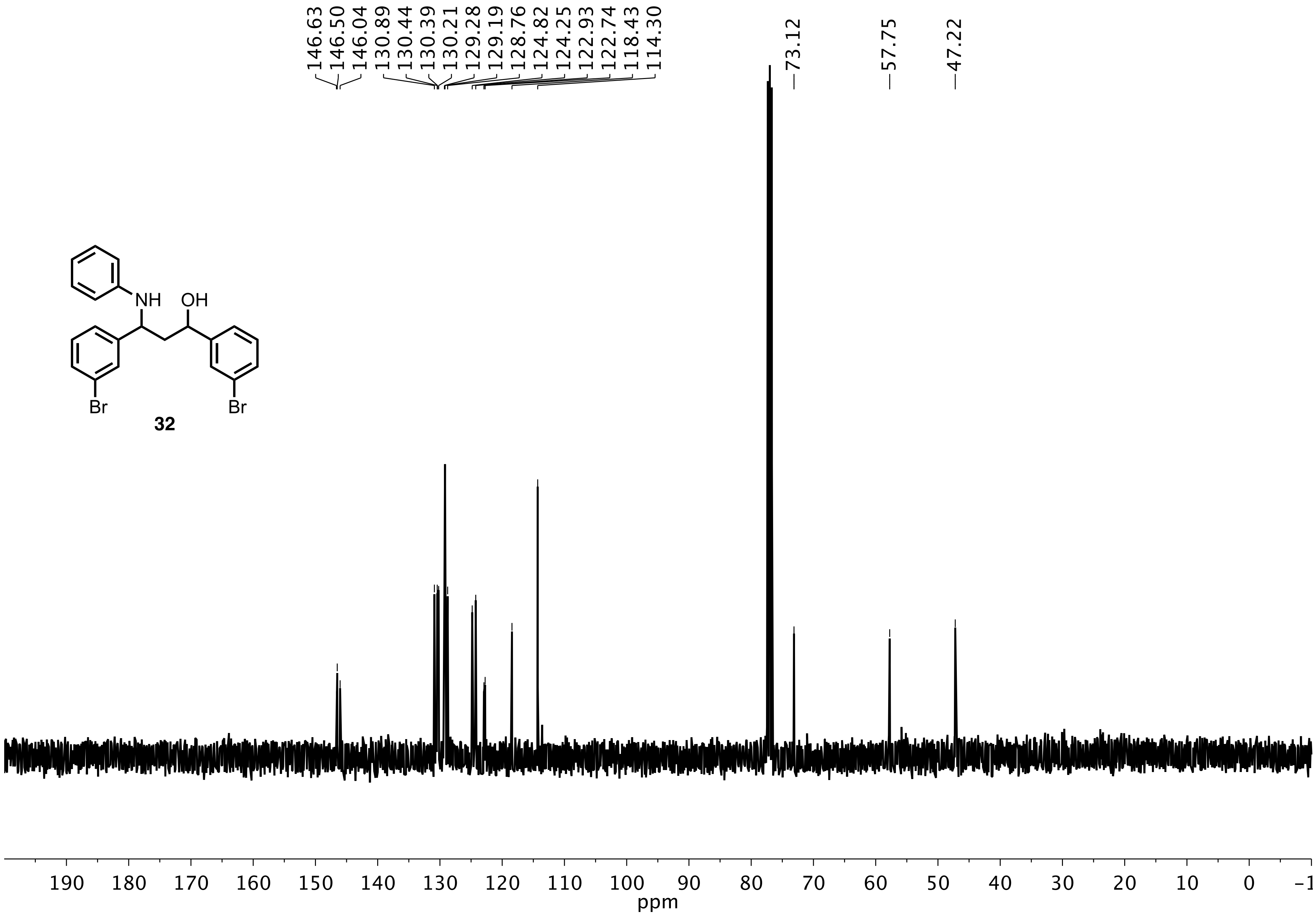
146.63
146.50
146.04
130.89
130.44
130.39
130.21
129.28
129.19
128.76
124.82
124.25
122.93
122.74
118.43
114.30
73.12
57.75
47.22
NH
OH
Br
Br
32
190
180
170
160
150
140
130
120
110
100
90
80
70
60
50
40
30
20
10
0
–1
ppm

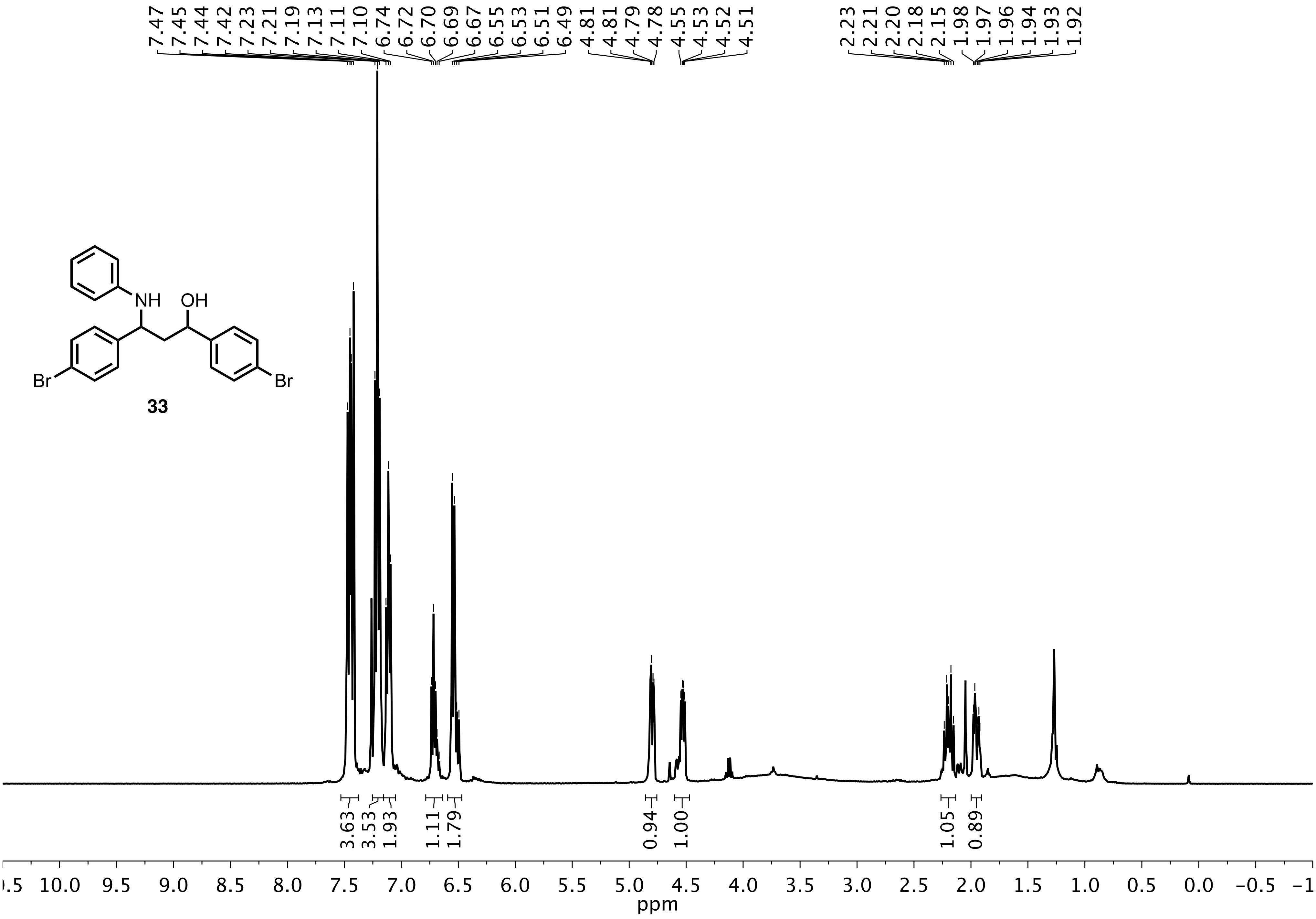

7.47
7.45
7.44
7.42
7.23
7.21
7.19
7.13
7.11
7.10
6.74
6.72
6.70
6.69
6.67
6.55
6.53
6.51
6.49
4.81
4.81
4.79
4.78
4.55
4.53
4.52
4.51
2.23
2.21
2.20
2.18
2.15
1.98
1.97
1.96
1.94
1.93
1.92
NH
OH
Br
Br
33
3.63
3.53
1.93
1.11
1.79
0.94
1.00
1.05
0.89
10.0 9.5 9.0 8.5 8.0 7.5 7.0 6.5 6.0 5.5 5.0 4.5 4.0 3.5 3.0 2.5 2.0 1.5 1.0 0.5 0.0 −0.5
ppm

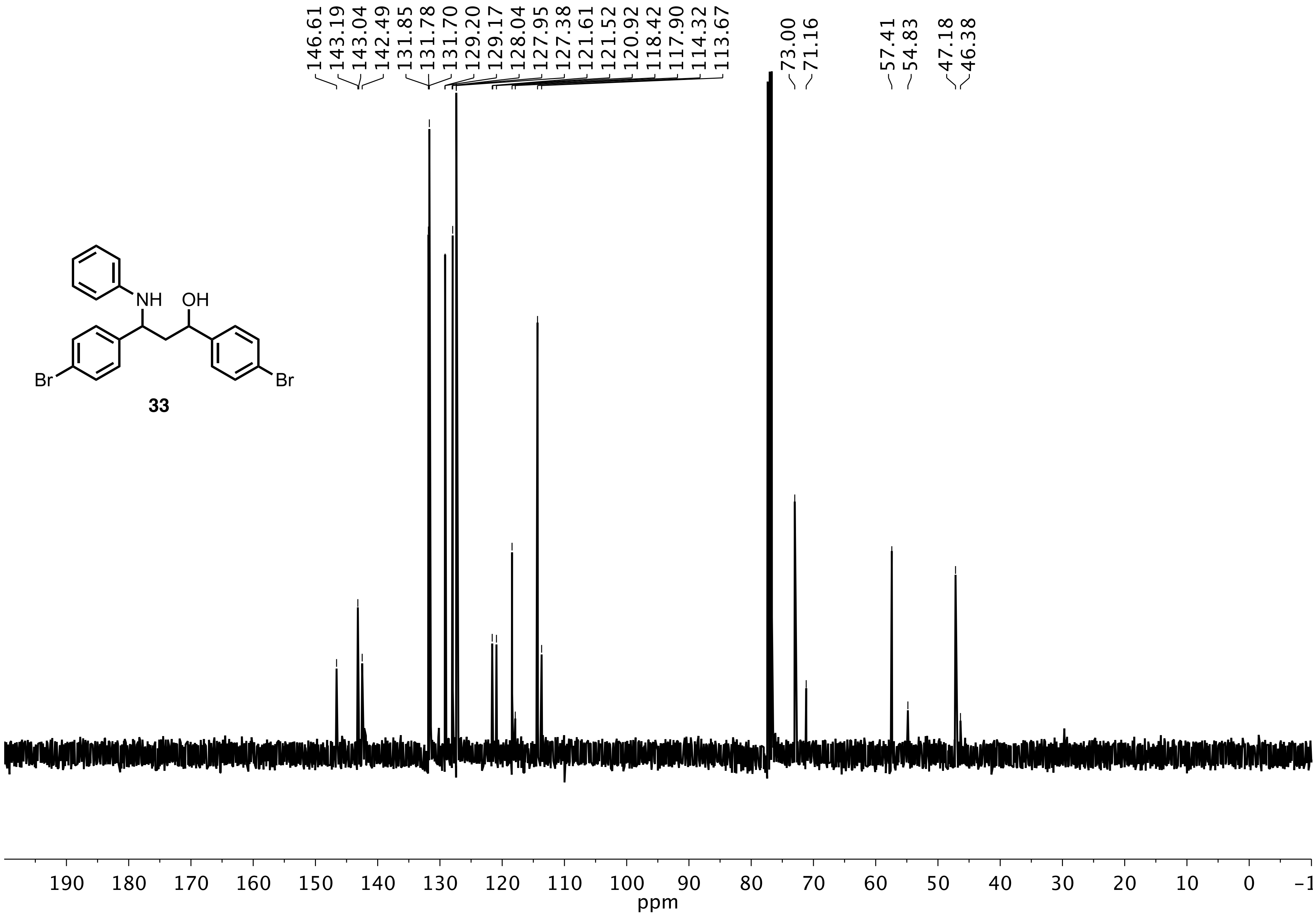

146.61
143.19
143.04
142.49
131.85
131.78
131.70
129.20
129.17
128.04
127.95
127.38
121.61
121.52
120.92
118.42
117.90
114.32
113.67
73.00
71.16
57.41
54.83
47.18
46.38
NH
OH
Br
Br
33
ppm

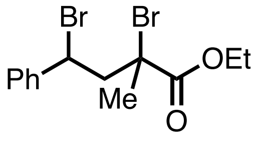

34

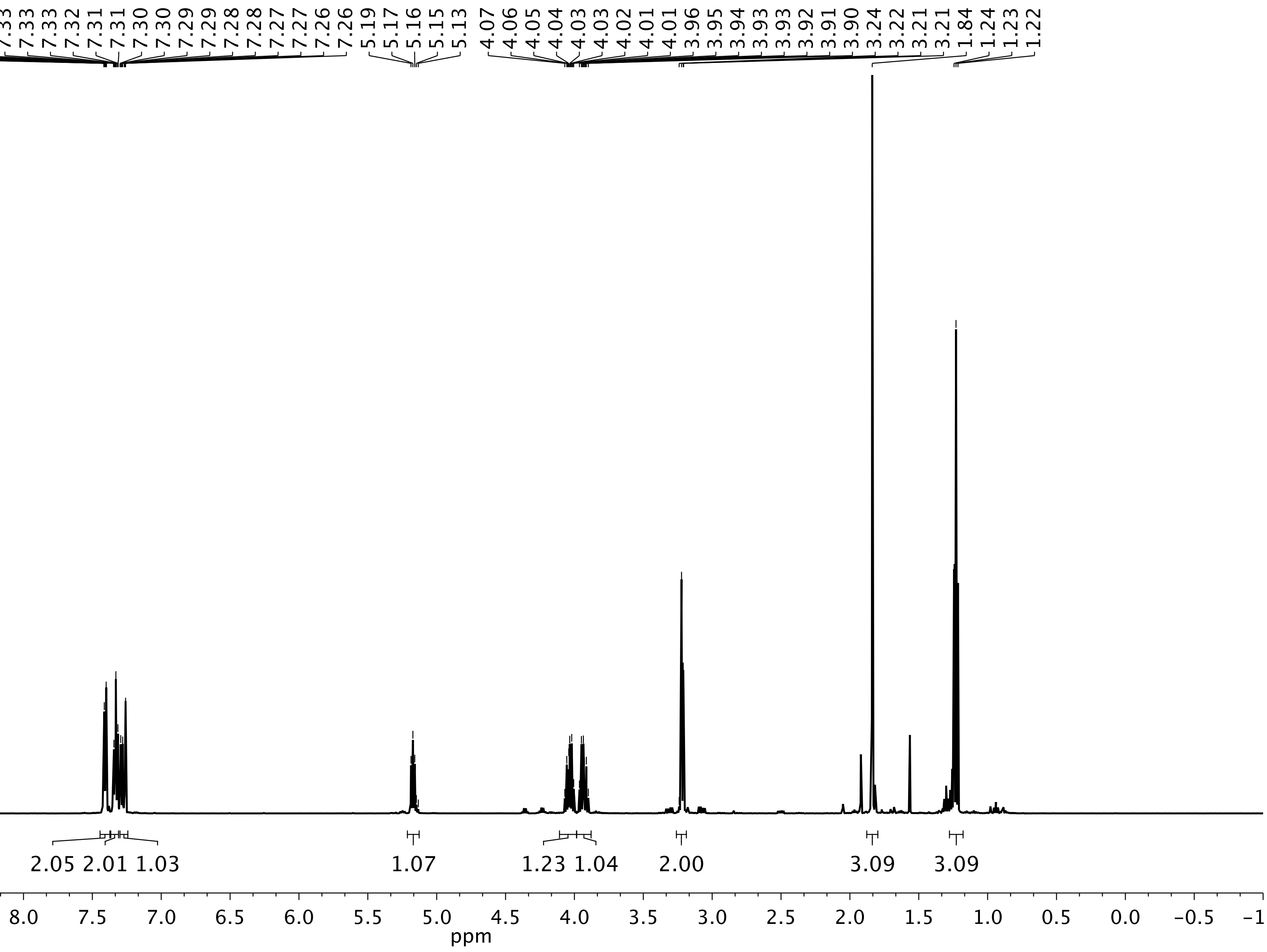
7.42 7.41 7.41 7.40 7.40 7.40 7.35 7.34 7.34 7.33 7.33 7.33 7.33 7.32 7.31 7.31 7.30 7.30 7.29 7.29 7.28 7.28 7.27 7.27 7.26 7.26
5.19 5.17 5.16 5.15 5.13
4.07 4.06 4.05 4.04 4.03 4.03 4.02 4.01 4.01 3.96 3.95 3.94 3.93 3.93 3.92 3.91 3.90
3.24 3.22 3.21 3.21
1.84
1.24 1.23 1.22
2.05 2.01 1.03
1.07
1.23 1.04
2.00
3.09
3.09
10.0 9.5 9.0 8.5 8.0 7.5 7.0 6.5 6.0 5.5 5.0 4.5 4.0 3.5 3.0 2.5 2.0 1.5 1.0 0.5 0.0 −0.5 −1
ppm

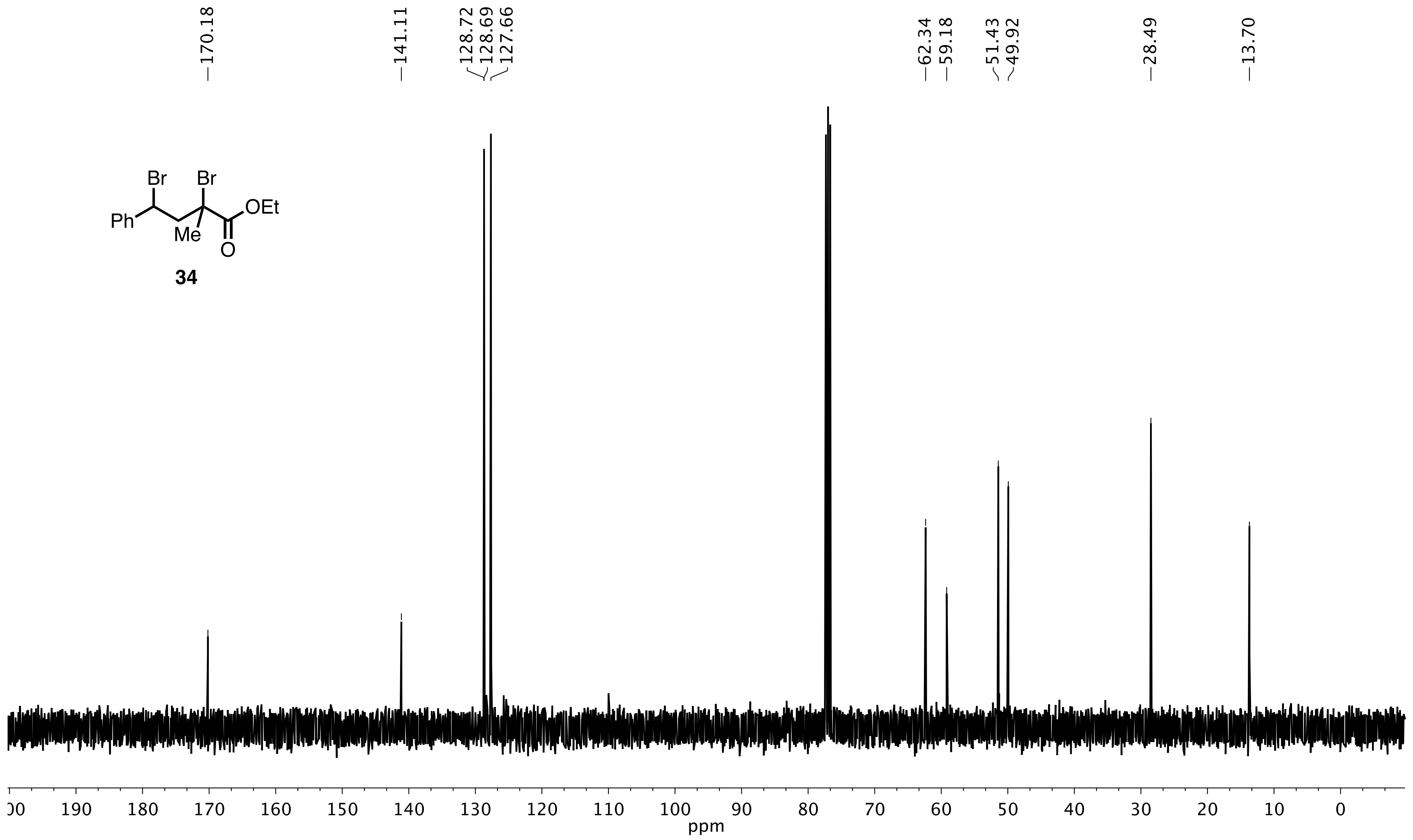
170.18
141.11
128.72
128.69
127.66
62.34
59.18
51.43
49.92
28.49
13.70
Br
Br
Ph
OEt
Me
O
34
190
180
170
160
150
140
130
120
110
100
90
80
70
60
50
40
30
20
10
0
ppm

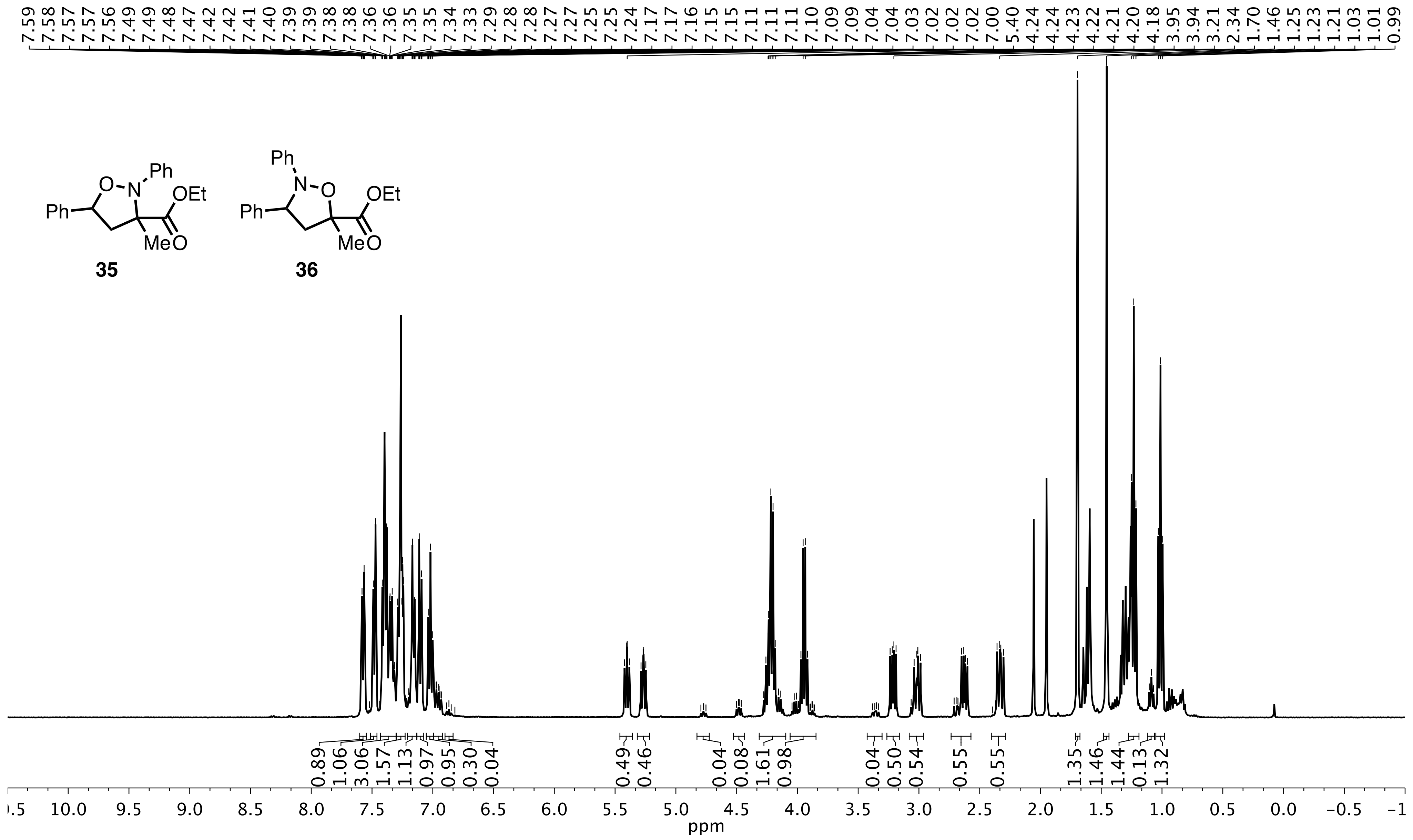
7.59 7.58 7.57 7.57 7.56 7.49 7.49 7.48 7.47 7.42 7.42 7.41 7.40 7.39 7.39 7.38 7.38 7.36 7.36 7.35 7.35 7.34 7.33 7.29 7.28 7.28 7.27 7.27 7.25 7.25 7.24 7.17 7.17 7.16 7.15 7.15 7.11 7.11 7.11 7.10 7.09 7.09 7.04 7.04 7.03 7.02 7.02 7.02 7.00 5.40 4.24 4.24 4.23 4.22 4.21 4.20 4.18 3.95 3.94 3.21 2.34 1.70 1.46 1.25 1.23 1.21 1.03 1.01 0.99
Ph
O
N
OEt
Ph
MeO
35
Ph
N
O
OEt
Ph
MeO
36
0.89 1.06 3.06 1.57 1.13 0.97 0.95 0.30 0.04 0.49 0.46 0.04 0.08 1.61 0.98 0.04 0.50 0.54 0.55 0.55 1.35 1.46 1.44 0.13 1.32
10.0 9.5 9.0 8.5 8.0 7.5 7.0 6.5 6.0 5.5 5.0 4.5 4.0 3.5 3.0 2.5 2.0 1.5 1.0 0.5 0.0 −0.5
ppm

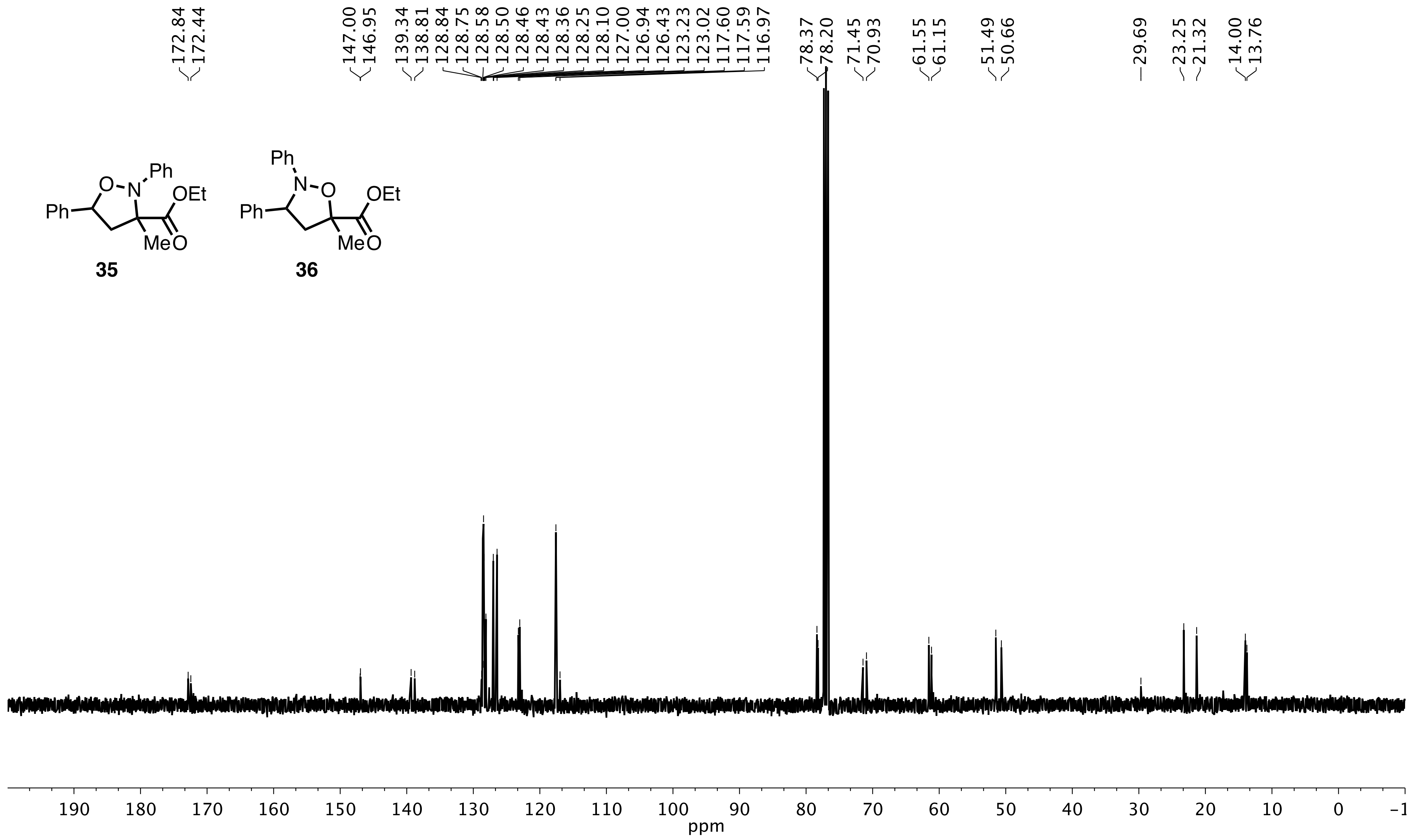
172.84
172.44
147.00
146.95
139.34
138.81
128.84
128.75
128.58
128.50
128.46
128.43
128.36
128.25
128.10
127.00
126.94
126.43
123.23
123.02
117.60
117.59
116.97
78.37
78.20
71.45
70.93
61.55
61.15
51.49
50.66
29.69
23.25
21.32
14.00
13.76
Ph
O
N
Ph
OEt
MeO
35
Ph
N
O
Ph
OEt
MeO
36
190
180
170
160
150
140
130
120
110
100
90
80
70
60
50
40
30
20
10
0
–1
ppm

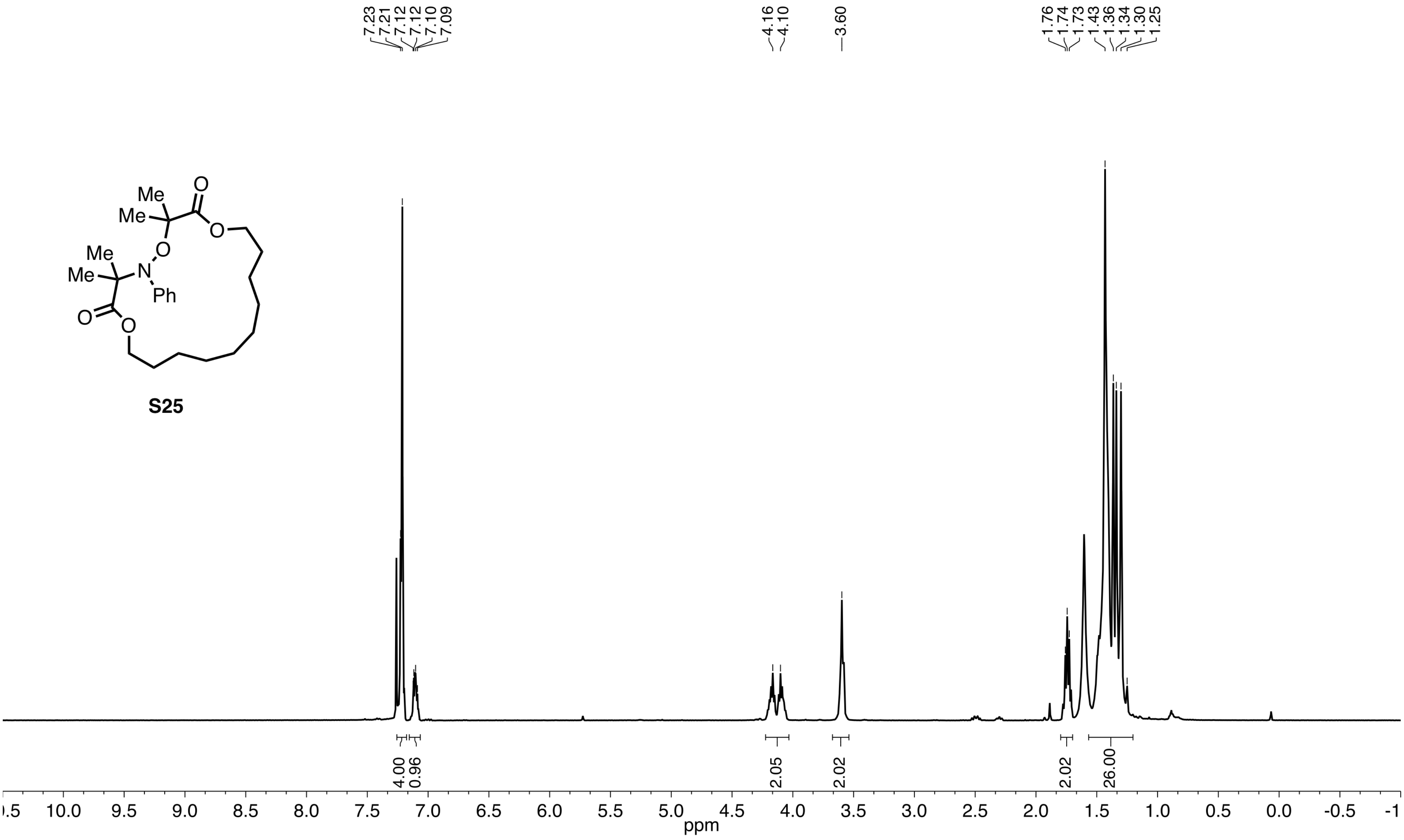
7.23
7.21
7.12
7.12
7.10
7.09
4.16
4.10
3.60
1.76
1.74
1.73
1.43
1.36
1.34
1.30
1.25
Me
Me
O
O
Me
Me
N
O
Ph
O
O
4.00
0.96
2.05
2.02
2.02
26.00
10.0
9.5
9.0
8.5
8.0
7.5
7.0
6.5
6.0
5.5
5.0
4.5
4.0
3.5
3.0
2.5
2.0
1.5
1.0
0.5
0.0
-0.5
-1
ppm

S25

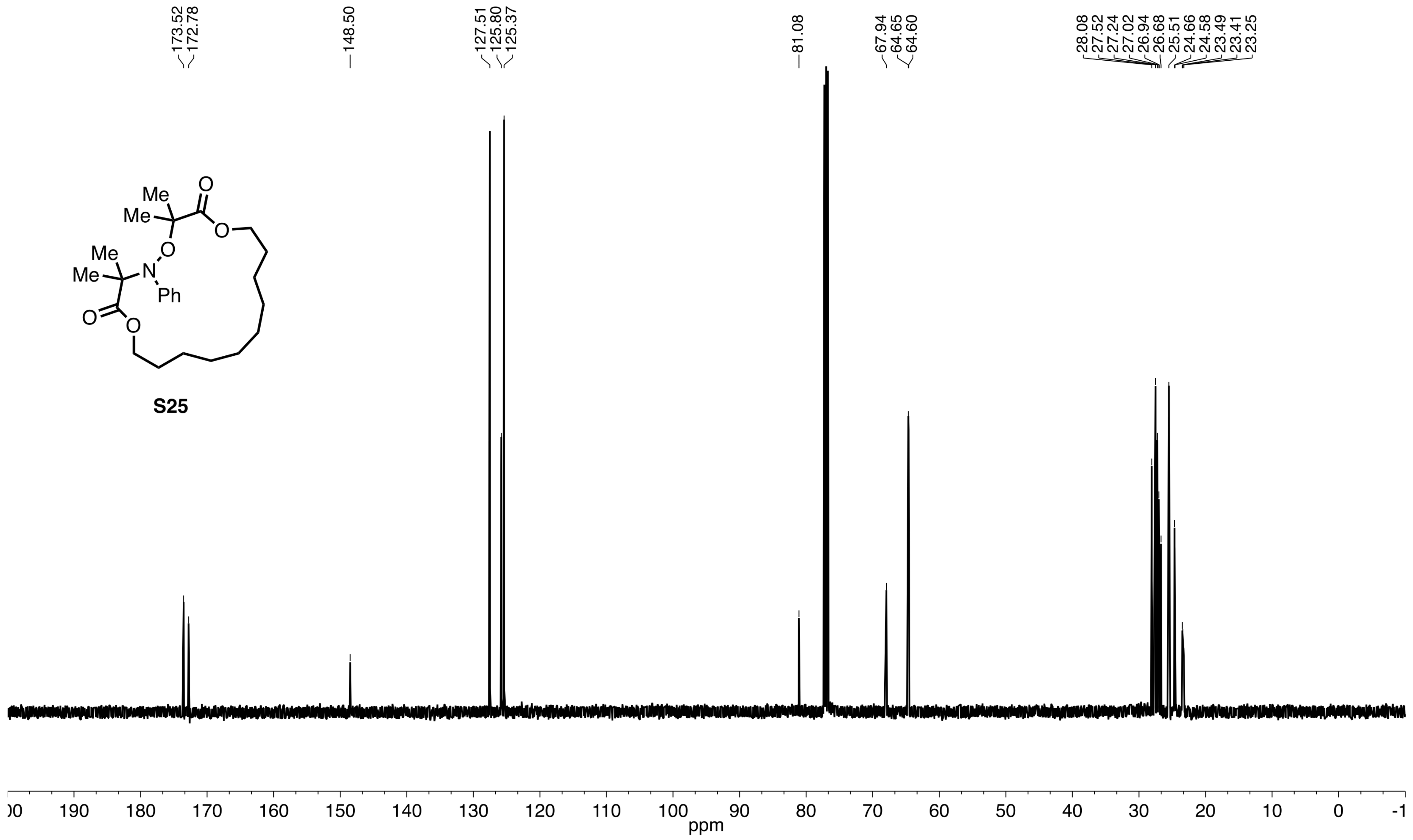

173.52
172.78
148.50
127.51
125.80
125.37
81.08
67.94
64.65
64.60
28.08
27.52
27.24
27.02
26.94
26.68
25.51
24.66
24.58
23.49
23.41
23.25
Me
Me
O
O
N
Ph
S25
ppm
